# Clinical Outcomes of Adjunctive Corticosteroid Therapy Versus Standard Treatment Alone in Patients With Bacterial Facial Infections: A Systematic Review and Meta‐Analysis

**DOI:** 10.1002/cre2.70377

**Published:** 2026-05-15

**Authors:** Umer Hussain, Wajiha Abbas, Inayat Ur Rahman, Imad Ali, Umer Ullah, Muhammad Yousaf Ali, Nadia Mansoor, Nuha Abdalla Osman Mustafa, Riyadh Alroomy, Roqayah Ibrahim Aljuailan, Asif Rehman, Badi Alotaibi

**Affiliations:** ^1^ Ministry of Health Saudi Arabia Riyadh Saudi Arabia; ^2^ Balochistan Institute of Child Health Services Quetta Pakistan; ^3^ Saidu College of Dentistry Swat Pakistan; ^4^ Saidu College of Dentistry, Oral and Maxillofacial Surgery Swat Pakistan; ^5^ Oral and Maxillofacial Surgery, Rehman College of Dentistry Peshawar Pakistan; ^6^ Fatima Jinnah Institute of Dental Sciences Karachi Pakistan; ^7^ Oral Medicine and Diagnostics, Bacha Khan Dental College Mardan Pakistan; ^8^ Surgical and Diagnostic Science Department Dar AlUloom University Riyadh Saudi Arabia; ^9^ Department of Conservative Dental Science, College of Dentistry Qassim University Buraydah Saudi Arabia; ^10^ Department of Conservative Dental Sciences, College of Dentistry Qassim University Al Mulayda Buraydah Saudi Arabia; ^11^ Bath Spa University Bath UK; ^12^ Department of Conservative Dental Sciences, College of Dentistry Qassim University Buraydah Saudi Arabia

**Keywords:** corticosteroid, facial infection, hospital stay, meta‐analysis, number of surgeries, odontogenic infection, orbital cellulitis, steroid, systematic review

## Abstract

**Background:**

**Objectives:** To evaluate the clinical effectiveness and safety of adjunctive corticosteroid versus standard treatment alone in facial infections.

**Methods:**

**Eligibility criteria:** Included clinical comparative studies (randomized or non‐randomized) evaluating adjunctive corticosteroids versus standard care in patients with bacterial facial infections such as odontogenic/facial space infections, dental abscesses, orbital cellulitis, periorbital cellulitis, and Ludwig's angina.

**Information sources:** An unrestricted literature search of five databases was conducted up to December 15, 2025.

**Risk of bias:** The quality assessment of the studies was conducted using the Cochrane Risk of Bias Tool (ROBINS‐I) for non‐randomized and a new risk of bias tool (RoB‐2) for randomized studies.

**Synthesis of results:** Random effects meta‐analyses using mean difference (MD) or standardized mean differences (SMDs) were performed, followed by sensitivity analyses, and assessment of the quality of evidence using GRADE.

**Results:**

**Included studies:** Fifteen comparative studies (three randomized and 12 non‐randomized) including 13,905 patients with bacterial facial infections (61.5% male) were included.

**Synthesis of results:** Low‐certainty evidence showed that the use of adjunctive steroids significantly reduced hospital stay compared with standard treatment alone (10 studies; MD = −1.61 [−3.17, −0.05] days; *p* = 0.04; *I* ^2^ = 99.3%). Low‐certainty evidence also showed that pre‐treatment CRP was higher in the steroid groups (3 studies; SMD = 0.33 [0.09, 0.58]; *p* = 0.03; *I* ^2^ = 0%). Low‐certainty evidence indicated no significant difference in the number of surgeries (*p* = 0.13), while very low‐certainty evidence showed no significant differences in intensive care unit admissions (*p* = 0.07) or airway compromise (*p* = 0.18). Sensitivity and subgroup analyses showed no significant differences by study design, infection type, or risk of bias, confirming robust results; only ethnicity had a significant effect on hospital stay (*p* = 0.0027).

**Discussion:**

**Limitations of evidence:** Certainty of evidence was low to very low for all outcomes, with hospital stay rated low due to methodological limitations, high risk of bias, and the predominance of non‐randomized studies.

**Interpretation**: The low level of evidence suggests that adjunctive corticosteroids may provide a reduction in hospital length of stay for patients with bacterial facial infections. Further well‐designed, randomized, and prospective comparative studies are needed to confirm these findings.

**PROPSERO Registration Number:** (CRD420261329331).

**Abbreviations**
CI: confidence interval
CRP: C‐reactive protein
GRADE: Grading of Recommendations, Assessment, Development, and Evaluation
ICU: intense care unit
MD: mean difference
NRS: non‐randomized studies
NSAIDs: non‐steroidal anti‐inflammatory drugs
OR: odds ratio
PICOS: population, intervention, comparator, outcomes, study design
PRISMA: Preferred Reporting Items for Systematic Reviews and Meta‐Analyses
PROSPERO: International Prospective Register of Systematic Reviews
RCTs: randomized controlled trials
ROBINS‐I: Risk of Bias in Nonrandomized Studies
RoB‐2: risk of bias 2 tool
SMD: standardized mean difference

## 1 Introduction

### 1.1 Background

Facial infections are a diverse group of conditions affecting the skin, subcutaneous tissues, facial spaces, and odontogenic structures in the maxillofacial region (Bali et al. 2015). These infections may be classified according to the causative pathogen as bacterial, viral, fungal, or mixed microbial infections. Bacterial infections are the most common and include odontogenic infections, cellulitis, and deep fascial space infections, whereas fungal infections are less common and usually occur in immunocompromised patients, while viral infections more often involve superficial soft tissues or mucocutaneous structures (Bali et al. 2015). These infections may originate from pathology of odontogenic structures, trauma, sinus infections, or dermatologic causes (Branstetter and Weissman 2003). They may present as localized cellulitis or can progress to more severe forms, including deep neck space infections and/or mediastinitis (Shareef et al. 2024). In case of inadequate and delayed treatment, these infections may result in severe complications such as airway compromise, cavernous sinus thrombosis, orbital cellulitis, sepsis, and even mortality (Wang et al. 2025). An incidence of 9.8 cases per 100,000 has been reported for these infections, with mortality reaching 1.4% (Velhonoja et al. 2020).

Standard management of bacterial facial infections included the prompt administration of appropriate antimicrobial therapy, empirical and based on culture sensitivity, surgical drainage when indicated, removal of the source of infection, and supportive care (Caruso et al. 2022; Lou et al. 2024). Although antibiotics remain the mainstay of treatment, targeting common causative organisms such as *Staphylococcus aureus*, streptococcal species, and anaerobic bacteria, inflammation and tissue edema often contribute significantly to pain, trismus, dysphagia, and airway compromise, thereby increasing morbidity and prolonging hospital stay (Delbet‐Dupas et al. 2020).

Adjunctive corticosteroids have been proposed in various facial and other infections as a potential strategy to reduce inflammatory edema, improve symptoms, and accelerate recovery (Chen et al. 2018). Corticosteroids exert potent anti‐inflammatory effects by suppressing cytokine production, reducing vascular permeability, and limiting leukocyte migration (Shukla et al. 2026). Their clinical efficacy has been demonstrated in other infectious and inflammatory conditions such as severe community‐acquired pneumonia and bacterial meningitis (Cheema et al. 2024). Nevertheless, concerns persist regarding their immunosuppressive properties, which may theoretically impair host defense mechanisms and exacerbate infection (Chastain et al. 2024).

The role of adjunctive corticosteroids in facial infections remains a subject of considerable debate due to their potential benefits and associated risks. Several studies have reported favorable outcomes, including reduced pain, accelerated resolution of swelling, and shorter hospitalization (Chen et al. 2018; Pushker et al. 2013; Davies et al. 2015; Suleman et al. 2025; Low et al. 2017). Delbet‐Dupas et al. (2020) reported fewer intensive care admissions and surgical interventions with corticosteroid use, although the length of stay was paradoxically longer. Conversely, other investigations have shown minimal or no benefit. They reported no significant reductions in hospitalization duration, surgical intervention, or complication rates, increased operative episodes, or elevated inflammatory markers in patients receiving corticosteroids (Yen and Yen 2005; Foster et al. 2018; Gill et al. 2022).

Some systematic reviews have previously been conducted to evaluate the role of systemic corticosteroids in head and neck infections, including deep neck space and orbital cellulitis. Sajdlowska et al. (2023) conducted a systematic review to assess the effect of steroids on hospital length of stay in head and neck infections. Their search identified only three eligible studies, and data were insufficient to perform a meta‐analysis, highlighting the limited quality and quantity of available evidence. Similarly, Mahalingam et al. (2021) reviewed the role of adjuvant corticosteroids in periorbital cellulitis and subperiosteal abscesses, identifying four studies with heterogeneous designs, small sample sizes, and very low certainty of evidence, which precluded definitive conclusions regarding efficacy. Kim and Bae (2022) performed a meta‐analysis on orbital cellulitis and reported reduced hospitalization with corticosteroids, but their study did not include other facial space infections.

### 1.2 Rationale

Despite increasing clinical interest in adjunctive corticosteroid therapy for facial infections, there is no clear consensus regarding its efficacy and safety. Existing studies vary in design, patient populations, infection severity, type and dose of corticosteroid administered, and outcome measures assessed. Furthermore, evidence remains scattered across different clinical settings, including odontogenic infections, cellulitis, and deep space infections. To date, no comprehensive synthesis has systematically evaluated the impact of adjunctive corticosteroid therapy on clinically relevant outcomes such as pain reduction, duration of swelling, trismus improvement, length of hospital stay, need for surgical intervention, and complication rates. A systematic review and meta‐analysis are therefore warranted to consolidate available evidence, assess the magnitude of treatment effects, evaluate heterogeneity across studies, and provide evidence‐based guidance for clinical practice.

### 1.3 Objective

The research question of this study was: in patients with bacterial facial infections, does adjunctive corticosteroid therapy in addition to standard treatment improve clinical outcomes compared with standard treatment alone? The objective of this systematic review and meta‐analysis was to evaluate the clinical effectiveness and safety of adjunctive corticosteroid therapy compared with standard treatment alone in patients with bacterial facial infections.

## 2 Methodology

### 2.1 Registration and Protocol

This systematic review and meta‐analysis were conducted in accordance with the methodological recommendations of the Cochrane Handbook for Systematic Reviews of Interventions (Higgins et al. 2022) and reported following the Preferred Reporting Items for Systematic Reviews and Meta‐Analyses (PRISMA) 2020 guidelines (Liberati et al. 2009). The review protocol was prospectively registered in the International Prospective Register of Systematic Reviews (PROSPERO) under the registration number CRD420261329331.

### 2.2 Eligibility Criteria

Our eligibility criteria were based on the PICOS format (Participants, Interventions, Comparator, Outcomes, and Study Design) principle. P: Patients with facial infections like odontogenic infections, facial space infections, dental abscesses, Ludwig's angina, or orbital cellulitis. I: Adjunctive oral or intravenous corticosteroids (e.g., dexamethasone, prednisolone, methylprednisolone) administered at any reported time point during treatment (e.g., on admission, within 6 h, within 48 h, or later during hospitalization), with no restriction on dose or course duration C: No steroid treatment or standard treatment of facial infection (antibiotics and NSAIDs). O: Reduction in swelling, pain relief, hospital stay, number of surgeries needed, levels of inflammatory markers, airway compromise, and other clinical outcomes. S: Clinical comparative studies, including randomized controlled trials and prospective or retrospective cohort/non‐randomized studies. Case reports, case series involving fewer than five patients, animal studies, studies on fungal, viral, or other non‐bacterial infections, and non‐clinical studies were excluded.

The primary outcomes of the review were hospital stay, number of surgeries required, and the occurrence of airway compromise. Secondary outcomes included reduction in swelling, pain relief, change in inflammatory markers, need for additional interventions, and other reported clinical outcomes associated with the management of facial infections.

### 2.3 Information Sources and Search Strategy

An unrestricted literature search was conducted across five electronic databases (PubMed, Scopus, Cochrane CENTRAL, LILACS, and Google Scholar [first 100 hits only]) using a predefined search strategy (Table S1). The search covered publications from inception up to December 15, 2025, without restrictions on publication date, language, type, or status. Reference lists of eligible articles and prior systematic reviews were also manually screened for additional relevant studies.

### 2.4 Selection Process

All records retrieved from the databases were exported into a Microsoft Excel 2016 spreadsheet. Titles were organized in ascending order, and duplicate entries were highlighted using a red font via an Excel function. A separate adjacent column was used to facilitate the identification and removal of these duplicates. Screening was conducted in two stages: initially, the titles and abstracts of all retrieved studies were evaluated against the predefined eligibility criteria, followed by full‐text review of potentially relevant articles. Study selection was performed independently by two authors (U.H. and W.A.), with any disagreements resolved through consultation with a third author (I.R.).

### 2.5 Data Collection Process and Items

Data extraction from the included studies was conducted using pre‐defined and piloted forms to ensure a consistent and thorough approach. Information was collected on study characteristics (such as country, design, and sample size), participant demographics (age and sex), type and severity of facial infection, corticosteroid regimen (including type, dose, route, and duration), comparator interventions (such as antibiotics and NSAIDs alone), reported adverse effects of steroids, primary outcomes (hospital stay, number of surgeries, and airway compromise), secondary outcomes (pain relief, reduction in swelling, inflammatory marker levels, and other clinical outcomes), and key findings. Extraction was carried out independently by two authors (I.A. and U.U.), and any disagreements were resolved through discussion with a third author (N.M.) to ensure accuracy and reduce the risk of bias.

### 2.6 Study Risk of Bias

The risk of bias was assessed using the Risk of Bias in Nonrandomized Studies of Interventions (ROBINS‐I) tool (Sterne et al. 2016) for non‐randomized studies new risk of bias tool (RoB‐2) (Higgins et al. 2019) for randomized controlled trial. The assessment was performed independently by two authors (M.Y.A. and N.A.O.M.), and any discrepancies were resolved through consultation with a third author (R.A.).

#### 2.6.1 Effect Measures and Synthesis Measures

Data analyses were carried out using R statistical software version 4.3.3. Quantitative synthesis was performed for outcomes reported in at least two studies. Due to clinical heterogeneity, such as differences in ethnicity, type and severity of facial infection, variation in dose and route of corticosteroid, and age of patients, a random‐effects model was used to estimate the average of the distribution of true effects (Papageorgiou 2014). Estimation of heterogeneity (tau^2^) was performed using the restricted maximum likelihood approach due to its greater reliability (Langan et al. 2019). The studies reported the effect sizes of outcome variables in the same unit, like hospital stay in days, and the mean difference (MD) was used. Those studies that reported outcomes as median with IQR were converted into mean and SD using an online calculator based on Luo et al. (2018) (https://www.math.hkbu.edu.hk/~tongt/papers/median2mean.html). Days to improvement reported in the studies were pooled with hospital stay, as both variables reflect the duration of clinical recovery. The standard deviation was computed from the reported 95% confidence intervals using the formula recommended in the Cochrane Handbook for Systematic Reviews of Interventions: SD = √n × (Upper CI − Lower CI)/3.92. To account for potential small‐study effects, pooled estimates were adjusted using the Hartung–Knapp method. Study heterogeneity was evaluated through visual inspection of forest plots, estimation of tau^2^ for absolute heterogeneity, and calculation of the *I* ^2^ statistic to quantify relative heterogeneity (Higgins et al. 2003). For meta‐analyses with three or more studies, 95% prediction intervals were calculated to account for heterogeneity and estimate the range of outcomes in future similar trials (Page et al. 2021). No unit‐of‐analysis issues were observed, as all studies employed independent groups without within‐person comparisons, and data were extracted from post‐treatment (discharge) measurements.

Sensitivity and subgroup analyses were conducted to evaluate the robustness of the findings with respect to study design, type of infection, and ethnicity. A *p*‐value ≤ 0.05 was considered statistically significant, except for tests of between‐subgroup heterogeneity, where a threshold of 0.1 was applied.

### 2.7 Publication Bias

Publication bias was assessed visually using funnel plots and quantitatively with Egger's regression test for outcomes reported in eight or more studies.

### 2.8 Certainty Assessment

The confidence in the meta‐analysis findings was evaluated using the GRADE (Grading of Recommendations, Assessment, Development, and Evaluation) framework (Guyatt et al. 2011). The results were then presented in updated summary of findings tables (Carrasco‐Labra et al. 2016).

## 3 Results

### 3.1 Study Search

Table S1 shows the search strategies and the number of records identified from four electronic databases for studies assessing the use of steroids in facial or odontogenic infections. The last search was performed on December 15, 2025. The search strategy used both MeSH terms and free‐text keywords related to corticosteroids (such as steroids, glucocorticoids, dexamethasone, prednisolone, and methylprednisolone) and infections of the facial or odontogenic region (including cellulitis, abscess, facial space infection, maxillofacial infection, and dental abscess). A total of 1562 records were retrieved from PubMed using a combination of MeSH terms and title keywords without applying restrictive filters. The Cochrane Library retrieved 679 trials, while Scopus identified 565 records through searches in titles, abstracts, and keywords. The search in Google Scholar produced 1300 records. Due to the large number of results, only the first 100 records were screened, from which three additional relevant articles were identified manually.

### 3.2 Study Selection

**Figure 1:**
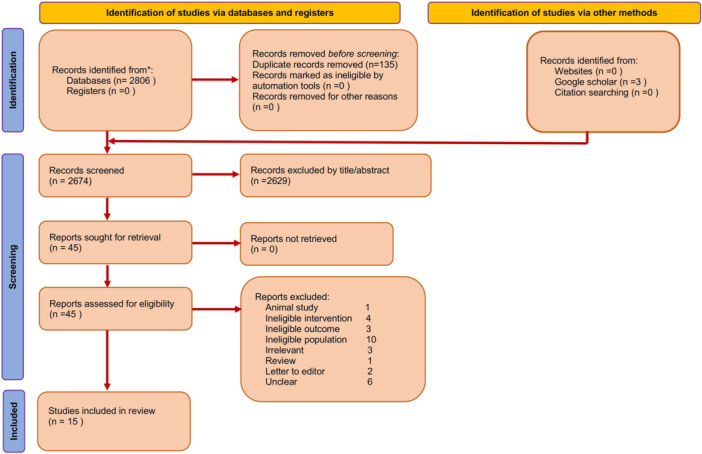
PRISMA flowchart of the study selection process.

A total of 2809 records were identified through the search process, including 2806 records from electronic databases and 3 from Google Scholar, while no records were obtained from websites or citation searching. After the removal of 135 duplicate records, 2674 records remained for screening. During the title and abstract screening stage, 2629 records were excluded as they did not meet the inclusion criteria. The full texts of 45 articles were subsequently retrieved and assessed for eligibility. Among these, 30 reports were excluded for the following reasons: animal study (*n* = 1), ineligible intervention (*n* = 4), ineligible outcomes (*n* = 3), ineligible population (*n* = 10), irrelevant studies (*n* = 3), review article (*n* = 1), letters to the editor (*n* = 2), and unclear reports (*n* = 6). Following the eligibility assessment, 15 studies met the inclusion criteria and were included in the systematic review (Figure 1).

### 3.3 Study Characteristics

The 15 included studies were conducted in nine countries: France, India, Israel, Malaysia, Nigeria, Pakistan, the UK, and the USA. Three studies were randomized controlled trials (RCTs), and the remaining 12 were non‐randomized studies (NRS), of which eight were prospective, and four were retrospective. A total of 13,905 participants were included across all studies. Fourteen studies reported gender distribution, with males representing 61.5% of participants (*n* = 8559). Twelve studies provided age data, with a median age of 27.05 years. Orbital cellulitis was the most frequently studied infection, reported in 8 out of 15 studies (53.3%). Odontogenic infections, including cervicofacial and facial space infections, were reported in 5 studies (33.3%), while periapical abscess and Ludwig's angina were each reported in 1 study (6.7%). Dexamethasone was the most commonly used steroid, appearing in nine studies, followed by prednisone, prednisolone, methylprednisolone, and general corticosteroids, with some studies using a combination of steroids. Steroids were generally administered alongside standard antibiotic therapy, with comparators being antibiotics alone, standard care without steroids, or, in one case, NSAIDs. Adverse effects of steroids were rare and mild, including occasional hyperactivity, insomnia, or hyperglycemia, while most studies reported no significant complications. The most frequently measured outcome was length of hospital stay, followed by the need for surgical intervention, CRP levels, severity of disease, ICU admission, readmission, and duration of antibiotics (Table 1).

**Table 1:** Characteristics of included studies.

Author (year)	Country; study design	Participants (M/F); mean age	Type of facial infection	Intervention (steroid regimen)	Comparator	Adverse effects of steroids	Outcome measured	Key findings
Chen et al. (2018)	USA; pRNS	43 (19/24); mean age 8.9 years	Orbital cellulitis	IV dexamethasone 0.3 mg/kg every 6 h × 3 days on admission (*n* = 28)	IV antibiotics alone (*n* = 15)	Hyperactivity (11%), insomnia (4%)	Length of hospital stay	Steroid group had significantly shorter hospital stay (3.8 ± 0.2 vs. 6.7 ± 0.3 days; *p* < 0.001).
Leszczynska et al. (2021)	USA; rRNS	5645 (3639/2006); median age 6 years	Orbital cellulitis	Systemic dexamethasone (in 88.8%) and methyl prednisolone (8.2%) within 2 days of admission (*n* = 1347)	No corticosteroids (*n* = 4298)	No adverse events	LOS; operative episodes; ICU admission; 30‐day readmission	Multivariate analysis found that corticosteroid use does not affect LOS but significantly increases the operative episode (OR = 2.5 [1.5–3.9]) and re‐admission (OR = 2.5 [1.5–3.78]).
Baumann et al. (2021)	USA; pRCT	73 (34/39); mean age 33.9 years	Periapical abscess	Single oral dexamethasone 10 mg (*n* = 37)	Placebo (*n* = 36)	No adverse events	Pain score up at baseline, discharge 12 h, 24 h, 48 h, 72 h	Significant pain reduction at 12 h (*p* = 0.029); no difference beyond 12 h.
Brameli et al. (2018)	Israel; rRNS	35 (22/13); mean age ~6 years	Orbital cellulitis	Prednisone (*n* = 6) or methylprednisolone ≤ 1 mg/kg/day or > 1 mg/kg/day (1–15 days) (*n* = 8)	Antibiotics alone (*n* = 21)	No adverse events	Severity of disease on basis of C‐reactive protein, need of combination antibiotics	Faster improvement with steroids (1.50 vs. 3.72 days adjusted); higher doses associated with faster recovery.
Kent et al. (2021)	UK; pNRS (multicentre)	1002 (546/456); mean age 37.5 years	Cervicofacial infections (mostly odontogenic 822/1002)	Systemic steroids (commonly dexamethasone; also hydrocortisone, methylprednisolone); (*n* = 357)	No steroids (standard treatment) (*n* = 645)	3 patients (0.8%) had hyperglycemia	LOS, CRP and airway compromise	Steroids group has higher CRP, airway compromise, Longer LOS
Christensen and Park (2025)	USA; pNRS	240 (138/102); mean age 40.3 years	Facial space odontogenic infection	Corticosteroids (primarily dexamethasone; dose varied) (*n* = 114)	Standard care without steroids (*n* = 126)	No significant steroid‐related complications	LOS, need for surgical intervention and CRP	No significant reduction in LOS and need for surgical intervention but higher CRP level in steroid group
Yen and Yen (2005)	USA; rRNS	23 pediatric patients (NR); age range:11 days–15 years)	Orbital cellulitis with SPA	IV corticosteroids (mainly dexamethasone 0.33–1 mg/kg; single dose every 12 h up to 7 days) (*n* = 12)	Standard care without steroids (*n* = 11)	No adverse reactions reported	Length of hospital stay; need for surgical drainage	No significant difference in LOS (*p* = 0.26) or need of surgical intervention (*p* = 0.20)
Gill et al. (2022)	USA; rNRS	5832 children (3546/2286); median age 5 years	Orbital cellulitis	Systemic corticosteroids (dexamethasone, prednisone, prednisolone, or methylprednisolone) (*n* = 330)	Standard care without steroids (*n* = 5502)	No adverse reactions reported	Length of hospital stay; surgical intervention; ICU admission; readmission; hospital costs	Early corticosteroid use was not associated with reduced length of stay, surgical intervention, ICU admission, readmission, or costs
Delbet‐Dupas et al. (2020)	France; pRNS	653 (386/267); mean age 37 years	Severe odontogenic infection	E1: Corticosteroids on admission (*n* = 50) E2: NASIDs with corticosteroids (*n* = 37)	C1: Standard care without steroids and NSAIDs (*n* = 263) C2: NSAIDs (*n* = 242)	No adverse reactions reported	ICU admission, number of surgeries, LOS, need for tracheotomy	CS group has significantly less ICU admission, less number of surgeries and longer LOS
Foster et al. (2018)	USA; rNRS	85 (52/33); median age: periorbital 6.0 years, orbital 1.2 years	Periorbital cellulitis (*n* = 58), orbital cellulitis (*n* = 27)	IV steroids for orbital cellulitis (*n* = 12)	IV antibiotics alone in orbital cellulitis (*n* = 15)	No adverse reactions reported	LOS, duration of antibiotics	Steroid treatment did not affect either the length of hospital ization or duration of antibiotic treatment.
Pushker et al. (2013)	India; pRCT	21 (15/6); mean age: ~22years	Orbital cellulitis	Oral prednisolone started on day 4–7 after initial IV antibiotics; 1.5 mg/kg/day (*n* = 14)	IV antibiotics alone (*n* = 7)	No adverse reactions reported	Pain, duration of IV antibiotics, LOS	The mean duration of IV antibiotics was longer than with steroids (11.6 ± 4.6 vs. 8.6 ± 1.3 days; *p* = 0.013), and hospital stay was also shorter with steroids (14.1 ± 3.7 vs. 18.4 ± 5.9 days; *p* = 0.02).
Low et al. (2017)	Malayesia; rNRS	30 (22/8); mean age 32.1 years	Odontogenic infections	IV dexamethasone 8 mg every 8 h, with antibiotics	Antibiotics alone	No adverse reactions reported	LOS, pain, mouth opening	Steroid group has less pain, edema, mouth opening, and hospital stay
Suleman et al. (2025)	Pakistan; pRCT	130 (79/51); mean age 44.1 years	Odontogenic infections	IV dexamethasone 8 mg every 8 h × 3 days + IV antibiotics (*n* = 65)	IV antibiotics alone (*n* = 65)	No adverse reactions reported	Change in swelling size, change in mouth opening, need for surgery, length of hospital stay	Steroid group had significantly greater reduction in swelling (3.26 ± 0.67 vs. 2.93 ± 0.62 cm; *p* = 0.004), greater increase in mouth opening (1.24 ± 0.56 vs. 0.93 ± 0.74 cm; *p* = 0.003), and shorter hospital stay (3.89 ± 1.00 vs. 4.46 ± 1.10 days; *p* = 0.010)
Ekaniyere and Dauda (2020)	Nigeria; rNRS	62 (36/26); mean age 40.6 years	Ludwig's angina	IV dexamethasone stat 10 mg first 24 h, then 5 mg q6h for 48 h, plus IV antibiotics (*n* = 29)	Surgical decompression + IV antibiotics (*n* = 33)	No adverse reactions reported	Airway compromise, length of hospital stay (LOS)	Airway compromise was 28.0% vs. 8.1% (pharmacological vs surgical; *p* = 0.47), and LOS was 13.8 ± 5.84 vs. 8.05 ± 3.87 days, both not significant.
Davies et al. (2015)	USA; pNRS	31 (25/6); mean age ~8.5 years	Pediatric orbital cellulitis	Oral prednisone 1 mg/kg/day × 7 days, started plus IV antibiotics (*n* = 24)	IV antibiotics and/or surgery (*n* = 7)	Hyperactivity (*n* = 2)	LOS	Steroid group had shorter hospitalization (1.1 vs. 4.9 days; *p* < 0.01).

Abbreviations: CRP, C‐reactive protein; CS, corticosteroid; h, hours; LOS, length of hospital stay (days); NR, not reported; NSAIDs, nonsteroidal anti‐inflammatory drugs; pRCT, prospective randomized controlled trial; pRNS, prospective non‐randomized study; rNRS, retrospective non‐randomized study; SPA, subperiosteal abscess.

### 3.4 Risk of Bias in Studies

**Figure 2:**
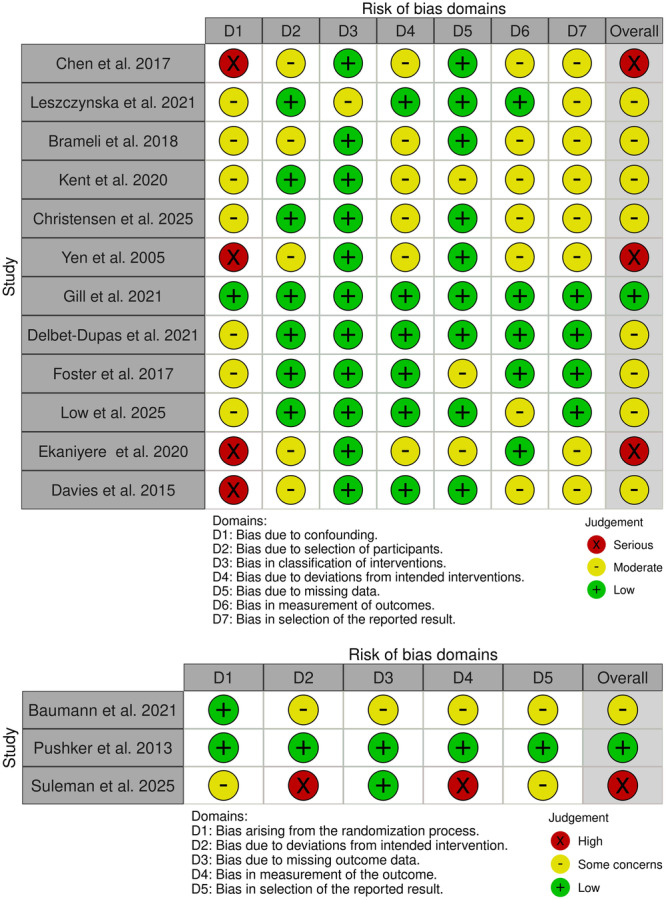
Risk of bias across the included studies.

The risk of bias across the included studies is summarized in Figure 2. Among the 12 non‐randomized studies, three were at serious risk of bias, mainly due to confounding (D1), while eight had moderate risk, largely related to participant selection (D2) and selective reporting (D7). One study showed low risk across all domains. Of the three randomized controlled trials, one was low risk, one had some concerns, and one was at high risk, with deviations from intended interventions (D2) and outcome measurement (D4) contributing most.

### 3.5 Results of Individual Studies and Data Syntheses

The complete results extracted from all included studies can be available from the corresponding author on reasonable request. Results of meta‐analyses with at least two studies can be seen in Table 2, while outcomes/comparisons assessed only from single studies can be seen in Table S2.

**Table 2:** Results of meta‐analyses (≥ 2 studies) comparing hospital stay, CRP, number of surgeries, ICU admissions, and airway compromises in patients having facial infection receiving adjunctive steroids versus standard treatment.

Outcome	*n*	Statistical metrics	Value	*p*	*τ* ^2^ [95% CI]	*I* ^2^ [95% CI]	95% Prediction
Hospital stay in days	10	MD [95% CI]	−1.61 [−3.17; −0.05]	0.04	3.95 [1.42; 14.89]	99.3% [99.1%; 99.4%]	−6.38; 3.16
CRP	3	SMD [95% CI]	0.33 [0.09; 0.58]	0.03	0.0001 [0.0; 4.37]	0.0% [0.0%; 89.6%]	0.08; 0.58
Number of surgeries	7	OR [95% CI]	0.48 [0.17; 1.34]	0.13	0.89 [0.25; 5.52]	91.7% [85.5%; 95.3%]	0.038; 6.06
ICU admissions	2	OR [95% CI]	0.56 [0.25; 1.27]	0.07	0 [‐,‐]	0.0% [‐,‐]	0.073; 4.32
Airway compromise	2	OR [95% CI]	0.17 [0.03; 101.01]	0.1786	0.0278 [‐,‐]	5.5% [‐,‐]	0.002; 142.48

Abbreviations: CI, confidence interval; CRP, C‐reactive protein; ICU, intensive care unit; MD, mean difference; *n*, number of studies; OR, odds ratio; *p*, *p*‐value; SMD, standardized mean difference.

### 3.6 Effect of Adjunctive Steroids on Clinical Outcomes in Patients With Facial Infections

**Figure 3:**
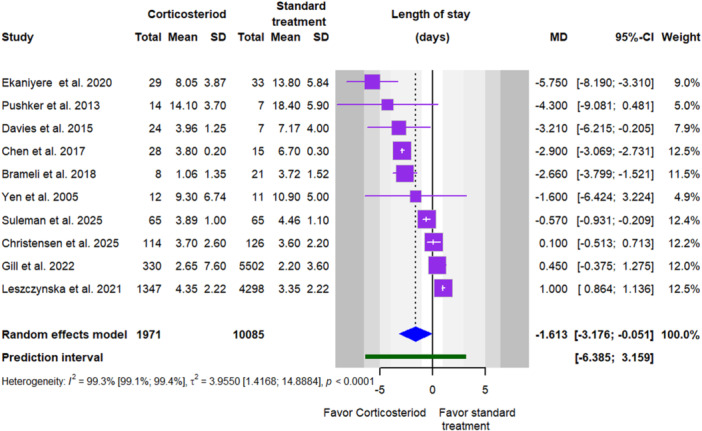
Forest plot comparing hospital stay in corticosteroid versus standard treatment.

**Figure 4:**
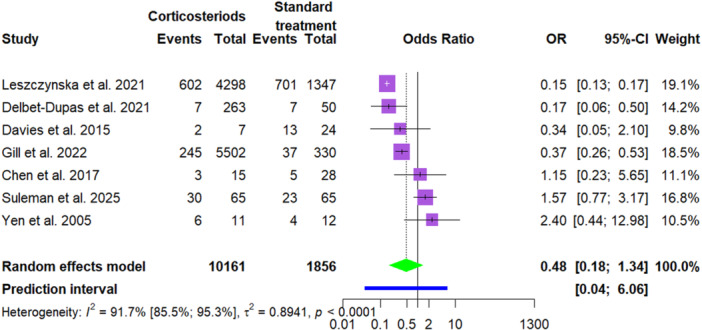
Forest plot comparing the number of surgeries in patients having facial infection receiving adjunctive steroids versus standard treatment.

Data from 10 studies showed that adjunctive steroids significantly reduced hospital stay (MD = −1.61 days, 95% CI: −3.17 to −0.05, *p* = 0.04), though heterogeneity was very high (*I* ^2^ = 99.3%, 95% CI 99.1% to 99.4%) and the prediction interval ranged from −6.38 to 3.16 days (Table 2 and Figure 3). Pre‐treatment CRP levels were reported in three studies and were significantly higher in patients receiving steroids (SMD = 0.33, 95% CI: 0.09 to 0.58, *p* = 0.03), with low heterogeneity (*I* ^2^ = 0%, 95% CI: 0.0%–89.6%) and a prediction interval from 0.08 to 0.58 (Table 2 and Figure 4). The number of surgeries, assessed in seven studies, was lower but not statistically significant (OR = 0.48, 95% CI: 0.17–1.34, *p* = 0.13), with high heterogeneity (*I* ^2^ = 91.7%, 95% CI: 85.5%–95.3%) and a prediction interval of 0.038–6.06. ICU admissions, evaluated in two studies, showed a non‐significant reduction (OR = 0.56, 95% CI: 0.25–1.27, *p* = 0.07), with no heterogeneity (*I* ^2^ = 0%) and a prediction interval of 0.073–4.32. Airway compromise, reported in two studies, also showed no significant effect (OR = 0.17, 95% CI: 0.03–101.01, *p* = 0.18), with low heterogeneity (*I* ^2^ = 5.5%) and a wide prediction interval from 0.002 to 142.48 (Table 2).

### 3.7 Source of Heterogeneity—Sensitivity/Subgroup Analyses

Sensitivity and subgroup analyses were conducted for hospital stay in patients with facial infections receiving adjunctive steroids versus standard treatment. There were no significant differences based on study design (non‐randomized studies vs. RCTs), type of infection (orbital cellulitis vs. odontogenic facial space infection), or risk of bias (serious, moderate, low), indicating the results were generally robust. The only notable variation was by ethnicity, with effect sizes differing across groups (American: −0.77 [−2.68; 1.15], Asian: −1.87 [−5.90; 2.16], African: −5.75 [−8.19; −3.31]), and this subgroup difference was statistically significant (*p* = 0.0027). Other subgroup comparisons did not show significant differences in mean hospital stay (Table 3).

**Table 3:** Sensitivity/subgroup analyses for meta‐analyses with at least eight included studies comparing hospital stay in patients having facial infection receiving adjunctive steroids versus standard treatment.

Outcome	Predictor	Group/Subgroup	*n*	MD [95% CI]	*p* *
Hospital study in days	Study design	Original analysis	10	−1.61 [−3.17; −0.05]	0.99
		Non‐randomized studies	8	−1.63 [−3.55; 0.28]	
		RCT	2	−1.65 [−23.09; 19.81]	
	Type of infection	Original analysis	10	−1.61 [−3.17; −0.05]	0.85
		Orbital cellulitis	7	−1.53 [−3.38; 0.31]	
		Odontogenic facial space infection	3	−1.88 [−9.62; 5.84]	
	Ethnicity	Original analysis	10	−1.61 [−3.17; −0.05]	0.0027
		American	6	−0.77 [−2.68; 1.15]	
		Asian	3	−1.87 [−5.90; 2.16]	
		African	1	−5.75 [−8.19; −3.31]	
	Risk of bias	Original analysis	10	−1.61 [−3.17; −0.05]	0.52
		Serious	4	−2.65 [−6.23; 0.91]	
		Moderate	4	−0.93 [−4.14; 2.27]	
		Low	2	−1.32 [−30.48; 27.85]	

Abbreviations: CI, confidence interval; MD, mean difference; *n*, number of studies; RCT, randomized controlled trials.

* *p*‐value for subgroup differences.

### 3.8 Publication Bias

**Figure 5:**
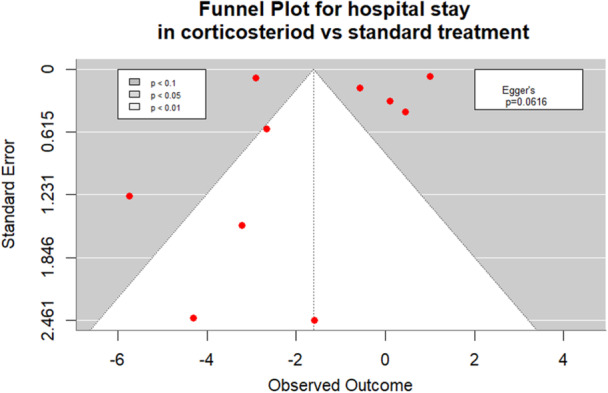
Funnel plot for hospital stay in corticosteroid vs. standard treatment.

Publication bias for hospital stay in patients receiving corticosteroids versus standard treatment was evaluated using a funnel plot and Egger's test. The funnel plot appeared approximately symmetrical, and the Egger's test was not statistically significant (*p* = 0.0616) thus indicating no evidence of publication bias (Figure 5).

### 3.9 Certainty of Evidence

Our certainty in the results of this analysis, assessed using the GRADE approach, ranged from low to very low across all outcomes. This was mainly due to the inclusion of non‐randomized studies, small sample sizes, inconsistency across studies, and wide confidence intervals. Adjunctive steroids slightly reduced hospital stay compared with standard treatment (MD = −1.61 days; 95% CI: −3.17 to −0.05) across 10 studies (2 RCTs and 8 NRS; *n* = 13,058), with low certainty. Pre‐treatment CRP levels were slightly higher in the steroid group (SMD = 0.33; 95% CI: 0.09–0.58) in 3 NRS (*n* = 1271), also with low certainty. The number of surgeries showed a trend toward reduction with steroids (OR = 0.48; 95% CI: 0.17–1.34) across 7 studies (1 RCT, 6 NRS; *n* = 10,951), but this was not statistically significant, with low certainty. ICU admissions (OR = 0.56; 95% CI: 0.25–1.27; 2 NRS, *n* = 179) and airway compromise (OR = 0.17; 95% CI: 0.03–101.01; 2 NRS, *n* = 27) showed little to no difference between groups, with very low certainty due to very few studies, imprecision, and extremely wide confidence intervals (Table 4).

**Table 4:** Summary of findings table according to the GRADE approach.

**Efficacy of adjunctive steroids versus standard treatment in facial infections**				
**Patient or population:** Patients with facial infections				
**Setting:** Multiple countries (USA, UK, India, Pakistan, Nigeria, Malaysia, France)				
**Intervention:** Adjunctive steroids				
**Comparison:** Standard treatment				
**Outcome**	**No. of participants (studies)**	**Certainty of the evidence (GRADE)**	**Effect size (difference with experimental group)**	**Interpretation/What happens with experimental treatment**
Hospital stay (days)	13,058 (10 studies: 2 RCTs, 8 NRS)	⊕⊕◯◯ Low^b^	MD = −1.61 [−3.17, −0.05]	Slight reduction in hospital stay with adjunctive steroids
CRP	1271 (3 NRS)	⊕⊕◯◯ Low^b^	SMD = 0.33 [0.09, 0.58]	Slight high CRP pre‐treatment levels in the steroid group
Number of surgeries	10,951 (7 studies: 1 RCT and 6 NRS)	⊕⊕◯◯ Low^b^	OR = 0.48 [0.17, 1.34]	Trend toward fewer surgeries, but not statistically significant
ICU admissions	179 (2 NRS)	⊕◯◯◯ Very low^c^	OR = 0.56 [0.25, 1.27]	Little to no difference in ICU admissions
Airway compromise	27 (2 NRS)	⊕◯◯◯ Very low^c^	OR = 0.17 [0.03, 101.01]	No clear effect; extremely wide confidence interval

Abbreviations: CRP, C‐reactive protein; MD, mean difference; NRS, non‐randomized study; OR, odds ratio; RCT, randomized controlled trial; SMD, standardized mean difference.

^a^ Low certainty: downgraded due to inconsistency and serious risk of bias in the studies.

^b^ Low certainty: downgraded due to a small number of studies and risk of bias.

^c^ Very low certainty: downgraded due to very few studies, imprecision, and a wide confidence interval.

## 4 Discussion

This systematic review and meta‐analysis synthesizes evidence from 15 clinical studies, including 3 randomized controlled trials and 12 non‐randomized studies, comprising a total of 13,905 patients with facial infections. To our knowledge, this is the first study to evaluate the effectiveness and safety of adjunctive corticosteroids across a broad range of facial infections, including orbital cellulitis, odontogenic infections, and deep cervicofacial infections.

In this study, the use of adjunctive steroids was associated with a reduction in the length of hospital stay compared to the standard treatment (10 studies, MD = −1.61 [− 3.17, −0.05] days; *p* = 0.04). Patients who received steroids also had slightly higher pre‐treatment CRP levels (SMD = 0.33[0.09, 0.58]; *p* = 0.03). Pre‐treatment CRP was generally higher in patients who received steroids, suggesting that most studies administered steroids to those with more severe facial infections. Despite this, the adjunctive steroid group still experienced shorter hospital stays, indicating that corticosteroids may be particularly effective in reducing the duration of hospitalization when used appropriately. These findings help clarify observations from earlier studies, which often reported no significant difference in length of stay between groups (Pushker et al. 2013; Suleman et al. 2025; Gill et al. 2022; Leszczynska et al. 2021; Baumann et al. 2021; Brameli et al. 2018). These studies noted that patients receiving steroids tended to have more severe infections, which could mask the potential benefit of steroids on hospital stay (Baumann et al. 2021; Brameli et al. 2018). This finding may also reflect confounding by indication, where clinicians preferentially selected steroids for patients with greater inflammatory burden; therefore, the observed benefit may represent a conservative estimate rather than an overestimation of effect.

Our results support this interpretation and suggest that if corticosteroids were administered in a randomized manner, the reduction in hospital stay might be even more pronounced. One important point to note is that while most individual studies reported non‐significant differences in hospital stay, the pooled analysis showed a statistically significant reduction. This is likely because combining multiple studies increased the overall sample size and statistical power, allowing a meaningful effect to emerge (Kent et al. 2021). The reduction in hospital stay seen with adjunctive corticosteroids can be explained by their ability to control inflammation. Steroids reduce the release of inflammatory substances and decrease swelling in the affected tissues, which helps relieve pain and discomfort (Pushker et al. 2013). As a result, patients recover faster, can eat and move more easily, and are able to be discharged earlier than those who receive standard treatment alone. From a biological perspective, suppression of pro‐inflammatory mediators and reduction of tissue edema may be particularly relevant in confined facial spaces where swelling can substantially impair function.

For other clinical outcomes—such as the need for the number of surgeries, ICU admissions, and airway compromise—the effects of corticosteroids were not significant. Although the number of surgeries was lower in the steroid group (Christensen and Park 2025; Sakkas 2023) but was not statistically different (7 studies, OR = 0.48 [0.17, 1.34], *p* = 0.13), and high variability between studies (*I* ^2^ = 91.7%). Similarly, ICU admissions and airway compromise showed no significant differences, with wide prediction intervals reflecting variation in patient characteristics and clinical practices. These outcomes are influenced by multiple factors beyond inflammation, including the severity and location of the infection as well as differences in institutional protocols. (Exley 2022; Quintana 2023) Because corticosteroids primarily reduce inflammation rather than directly addressing structural complications, their impact on these outcomes appears limited and variable (Ekaniyere and Dauda 2020). These outcomes were reported in less number of studies, so this may be due to less power; the results are not significant (Pandis 2013). The requirement for surgery is often determined by abscess formation, anatomical spread, or failure of source control, factors that may not be substantially altered by anti‐inflammatory therapy alone.

Previous reviews have provided important preliminary evidence on this topic. Sajdlowska et al. (2023) systematically reviewed head and neck infections and reported that two of three included studies showed reduced hospital stay with corticosteroid use, although insufficient data prevented meta‐analysis. Mahalingam et al. (2021) performed a review and meta‐analysis of periorbital cellulitis secondary to sinusitis and found significantly shorter inpatient stay with steroids, without significant differences in surgical intervention, while emphasizing heterogeneity and risk of bias. Kim et al. (2022) conducted a meta‐analysis of orbital cellulitis and reported shorter hospitalization with adjunctive corticosteroids, with no significant increase in surgical drainage. However, these reviews were limited by small study numbers or a narrower clinical scope. The current study expands upon these findings by including a broader spectrum of facial infections and updated comparative evidence.

The results of our study were heterogeneous with high *I* ^2^ values. Differences in the type of infection, ethnic variability of the participants, steroid doses, study sizes, and inclusion of non‐randomized studies could all contribute to variability. To address this, subgroup analyses were performed based on infection type and study design, considering ethnic differences and risk of bias. The subgroup analyses showed the reduction in hospital stay with steroids was generally robust. Only ethnicity was a significant confounder for length of hospital stay (*p* = 0.0027). This may reflect differences in healthcare systems, admission thresholds, discharge practices, or underlying population characteristics rather than ethnicity itself. However, due to a lack of available data, subgroup analyses were not possible for other outcomes.

The certainty of evidence for each outcome was evaluated using the GRADE approach, considering several domains like the number of studies and participants, study design, risk of bias, precision of estimates, directness of evidence, consistency across studies, and the magnitude of effect. Overall, the certainty of evidence ranged from “very low” to “low” for all outcomes. For the primary outcome of hospital length of stay, the evidence was graded as low certainty. Although the pooled analysis suggested that adjunctive corticosteroids may slightly reduce the duration of hospitalization, the certainty was downgraded due to methodological limitations and the inclusion of a large proportion of non‐randomized studies. In addition, variations in infection type, patient characteristics, and treatment protocols across studies contributed to inconsistency in the evidence. Similarly, the evidence for CRP levels was rated as low certainty. This outcome was reported in a limited number of studies, all of which were non‐randomized. Differences in baseline inflammatory status and the timing of CRP measurement may have influenced the observed results, reducing confidence in the overall estimate. For surgical intervention, the certainty of evidence was also low. Although the pooled estimate suggested a possible reduction in the number of surgeries among patients receiving adjunctive steroids, the confidence interval was wide and included the null value, indicating uncertainty regarding the true effect. The predominance of observational studies further limited the strength of the evidence. The certainty of evidence for ICU admissions and airway compromise was graded as very low. These outcomes were reported in only a small number of studies with limited sample sizes, resulting in substantial imprecision and wide confidence intervals. In particular, the extremely wide confidence interval observed for airway compromise reflects considerable uncertainty in the estimated effect. Accordingly, these findings should be interpreted cautiously and should not be viewed as definitive evidence for routine corticosteroid use in all facial infections.

Several limitations of the available evidence should be acknowledged. First, a large proportion of the included studies were non‐randomized, which increases the risk of selection bias and confounding. Second, insufficient and inconsistent reporting of data limited the ability to perform pooled analyses for several outcomes. Third, many studies had a high risk of bias, mainly due to retrospective designs and limited control for confounding factors. In addition, subgroup analyses were not possible for most outcomes because of the limited number of studies reporting comparable data, except for hospital length of stay. Severity of infection was also poorly reported across studies, which prevented subgroup analysis based on disease severity. We were also unable to perform subgroup analyses according to the timing and duration of corticosteroid administration because these data were inconsistently reported across the included studies. Finally, the inclusion of different types of facial infections, such as odontogenic infections and orbital cellulitis, may have introduced clinical heterogeneity that could influence the pooled estimates. Adverse effects related to corticosteroid therapy were also inconsistently reported, limiting conclusions regarding safety. Future well‐designed studies should use standardized severity classifications and outcome reporting to allow more meaningful subgroup analyses.

From a clinical perspective, adjunctive corticosteroids may be considered selectively after appropriate antimicrobial therapy and source control, particularly in patients where edema, pain, or functional limitation are prominent features. However, routine universal administration cannot be recommended on the basis of the current low‐certainty evidence.

This review has several key strengths. It is the first study to comprehensively address this research question by including 15 studies and performing a meta‐analysis. A systematic search was conducted across major databases to ensure thorough coverage of the literature. Where necessary, data reported as medians and interquartile ranges were converted to means and standard deviations to enable quantitative synthesis. The review was prospectively registered to ensure transparency. Additionally, robust statistical methods were applied, including random‐effects modeling, Hartung–Knapp adjustment, subgroup analyses where feasible, and assessment of evidence certainty using the GRADE approach, enhancing the reliability and interpretability of the findings.

## 5 Conclusion

The low level of evidence suggests that adjunctive corticosteroids may provide a modest reduction in hospital length of stay for patients with facial infections. However, their impact on other clinical outcomes like the need for surgery, ICU admissions, and airway compromise appears limited and uncertain. The overall certainty of evidence is low to very low. Further well‐designed randomized controlled trials with standardized protocols are needed to confirm their efficacy and safety and to guide clinical practice.

## Author Contributions

**Umer Hussain:** conceptualization (equal), investigation (equal), methodology (equal), project administration (equal), writing – original draft (equal), software (equal), formal analysis (equal). **Wajiha Abbas:** conceptualization (equal), investigation (equal), methodology (equal). **Inayat Ur Rahman:** data curation (equal), risk of bias (equal), investigation (equal), writing – review and editing (equal). **Imad Ali:** conceptualization (equal), data extraction (equal), writing – review and editing (equal). **Umer Ullah:** conceptualization (equal), investigation (equal), methodology (equal), writing – review and editing (equal). **Muhammad Yousaf Ali:** data curation (equal), risk of bias (equal), investigation (equal), writing – review and editing (equal). **Nadia Mansoor:** data extraction (equal), risk of bias (equal), investigation (equal), writing – review and editing (equal). **Nuha Abdalla Osman Mustafa:** conceptualization (equal), investigation (equal), methodology (equal), writing – review and editing (equal). **Riyadh Alroomy:** data curation (equal), risk of bias (equal), investigation (equal), writing – review and editing (equal). **Roqayah Ibrahim Aljuailan:** conceptualization (equal), investigation (equal), methodology (equal), writing – review and editing (equal). **Asif Rehman:** conceptualization (equal), investigation (equal), methodology (equal), data analysis, writing – review and editing (equal). **Badi Alotaibi:** writing curation (equal), risk of bias (equal), investigation (equal), writing – review and editing (equal).

## Funding

The authors have nothing to report.

## Ethics Statement

This study is a systematic review and does not involve human participants, patient data, or animal subjects.

## Consent

As this meta‐analysis is based solely on previously published data and does not involve human participants, consent was not applicable.

## Conflicts of Interest

The authors whose names are listed in title page certify that they have NO affiliations with or involvement in any organization or entity with any financial interest (such as honoraria; educational grants; participation in speakers' bureaus; membership, employment, consultancies, stock ownership, or other equity interest; and expert testimony or patent‐licensing arrangements), or non‐financial interest (such as personal or professional relationships, affiliations, knowledge or beliefs) in the subject matter or materials discussed in this manuscript. The authors declare no conflicts of interest.

## Supporting information


**Table S1:** Searches from four databases (last date of search: December 15, 2025).
**Table S2:** Individual studies meta‐analysis for various outcomes in patients with facial infection receiving adjunctive corticosteroid vs standard treatment.
**Table S3:** List of included and excluded studies.

## Data Availability

The data that support the findings of this study are available on request from the corresponding author. The data are not publicly available due to privacy or ethical restrictions.
