# Supplementary material for: Clinical Outcomes of Adjunctive Corticosteroid Therapy Versus Standard Treatment Alone in Patients With Bacterial Facial Infections: A Systematic Review and Meta‐Analysis
Source: Clin Exp Dent Res. 2026 May 15;12(3):e70377. doi: 10.1002/cre2.70377 (PMC13178280; doi:10.1002/cre2.70377)
Supplement: Supplementary file 1 — Table S1: Searches from four databases (last date of search: December 15, 2025). Table S2: Individual studies meta‐analysis for various outcomes in patients with facial infection receiving adjunctive corticosteroid vs standard treatment. Table S3: List of included and excluded studies. [file CRE2-12-e70377-s001.docx]

**Supplemental file**

**Clinical Outcomes of Adjunctive Corticosteroid Therapy versus Standard Treatment Alone in Patients with Facial Infections: A Systematic Review and Meta-Analysis**

Table S1. Searches from four databases (last date of search: December 15, 2025)

| Database | Keywords | Filter Applied | Hits |
| --- | --- | --- | --- |
| PubMed | ( ( ("Steroids"[Mesh] OR "Glucocorticoids"[Mesh]) OR (steroid*[Title] OR corticosteroid*[Title] OR dexamethasone[Title] OR prednisolone[Title] OR methylprednisolone[Title]) ) AND ( ("Cellulitis"[Mesh] OR "Abscess"[Mesh] OR "Tooth Diseases"[Mesh:NoExp] OR "Fasciitis"[Mesh]) OR ("facial space infection*"[Title] OR "maxillofacial infection*"[Title] OR "dental abscess*"[Title] OR "orbital cellulitis"[Title] OR "facial cellulitis"[Title] OR "facial infection"[Title] OR "odontogenic infection*"[Title]) )) | All Fields | 1562 |
| Scopus | TITLE-ABS-KEY ( (steroid* OR corticosteroid* OR glucocorticoid* OR dexamethason* OR prednisolon*) AND ("facial space infection*" OR "maxillofacial infection*" OR "odontogenic infection*" OR "dental abscess*" OR "orbital cellulit*" OR "periapical abscess*" OR "peri-apical abscess*")) | Article title, abstract, keywords | 565 |
| Cochrane Library | ( ("Steroids"[Mesh] OR "Glucocorticoids"[Mesh]) OR (steroid* OR corticosteroid* OR dexamethasone OR prednisolone OR methylprednisolone) ) AND ( ("Cellulitis"[Mesh] OR "Abscess"[Mesh] OR "Tooth Diseases"[Mesh:NoExp] OR "Fasciitis"[Mesh]) OR ("facial space infection*" OR "maxillofacial infection*" OR "dental abscess*" OR "orbital cellulitis" OR "facial cellulitis" OR "facial infection" OR "odontogenic infection*") ) in Title Abstract Keyword | Title, Abstract, Keywords | 679 trials |
| Google Scholar | (steroid OR corticosteroids) AND ("facial space infection" OR "odontogenic infection") | No | 1300(first 100 hits screened and three articles selected manually) |

Table S2. Individual studies meta-analysis for various outcomes in patients with facial infection receiving adjunctive corticosteroid vs standard treatment

| Study | Outcome | Statistics | Value | p value |
| --- | --- | --- | --- | --- |
| Chen et al., 2017 | Hospital stay | MD (95% CI) | −2.90 (−3.07 to −2.73) | <0.001 |
| Leszczynska et al., 2021 | Hospital stay | MD (95% CI) | 1.00 (0.86 to 1.14) | <0.001 |
| Brameli et al., 2018 | Hospital stay | MD (95% CI) | −2.66 (−3.80 to −1.52) | <0.001 |
| Christensen et al., 2025 | Hospital stay | MD (95% CI) | 0.10 (−0.51 to 0.71) | 0.75 |
| Yen et al., 2005 | Hospital stay | MD (95% CI) | −1.60 (−6.42 to 3.22) | 0.52 |
| Gill et al., 2022 | Hospital stay | MD (95% CI) | 0.45 (−0.38 to 1.28) | 0.29 |
| Pushker et al., 2013 | Hospital stay | MD (95% CI) | −4.30 (−9.08 to 0.48) | 0.08 |
| Suleman et al., 2025 | Hospital stay | MD (95% CI) | −0.57 (−0.93 to −0.21) | <0.001 |
| Ekaniyere et al., 2020 | Hospital stay | MD (95% CI) | −5.75 (−8.19 to −3.31) | <0.001 |
| Davies et al., 2015 | Hospital stay | MD (95% CI) | −3.21 (−6.22 to −0.20) | 0.04 |
| Brameli et al. 2018 | Pretreatment CRP level | SMD (95% CI) | 0.89 (0.04 to 1.74) | 0.04 |
| Kent et al. 2020 | Pretreatment CRP level | SMD (95% CI) | 0.34 (0.21 to 0.47) | <0.001 |
| Christensen et al. 2025 | Pretreatment CRP level | SMD (95% CI) | 0.27 (0.01 to 0.52) | 0.04 |

Table S3. List of included and excluded studies

| sno | Author | Title | | | | Publication Year | | database | by | reason for exclusion |
| --- | --- | --- | --- | --- | --- | --- | --- | --- | --- | --- |
| 1 | Galioto NJ | Peritonsillar Abscess | | | | 2017 | | pubmed | title/abstract | irrelevant |
| 2 | Voelker R | What Is Plantar Fasciitis? | | | | 2024 | | pubmed | title/abstract | irrelevant |
| 3 | Niklas K | [Eosinophilic fasciitis] | | | | 2015 | | pubmed | title/abstract | irrelevant |
| 4 | Lamback EB | Eosinophilic fasciitis | | | | 2016 | | pubmed | title/abstract | irrelevant |
| 5 | Kornelsen E | Corticosteroids for periorbital and orbital cellulitis | | | | 2021 | | pubmed | title/abstract | review |
| 6 | Lu JE | The Role of Steroids for Pediatric Orbital Cellulitis - Review of the Controversy | | | | 2023 | | pubmed | title/abstract | review |
| 7 | Mertens JS | Morphea and Eosinophilic Fasciitis: An Update | | | | 2017 | | pubmed | fulltext | review |
| 8 | Leszczynska MA | Corticosteroids for Acute Orbital Cellulitis | | | | 2021 | | pubmed | fulltext | irrelevant |
| 9 | Simjian T | Dexamethasone Administration and Mortality in Patients with Brain Abscess: A Systematic Review and Meta-Analysis | | | | 2018 | | pubmed | title/abstract | irrelevant |
| 10 | Kim BY | Role of systemic corticosteroids in orbital cellulitis: a meta-analysis and literature review | | | | 2022 | | pubmed | duplicate | duplicate |
| 11 | Peccerillo F | Eosinophilic cellulitis | | | | 2015 | | pubmed | title/abstract | irrelevant |
| 12 | Tsoi KL | Eosinophilic fasciitis | | | | 2012 | | pubmed | duplicate | duplicate |
| 13 | Tami A | Ludwig's angina and steroid use: A narrative review | | | | 2020 | | pubmed | duplicate | duplicate |
| 14 | Ito H | Ludwig's angina and steroid therapy | | | | 2021 | | pubmed | fulltext | letter to editor |
| 15 | Santos JC | Pediatric preseptal and orbital cellulitis: A 10-year experience | | | | 2019 | | pubmed | duplicate | duplicate |
| 16 | Dandeniya C | Eosinophilic fasciitis: experience with a patient and review of the potential mimics | | | | 2021 | | pubmed | title/abstract | review |
| 17 | Gill PJ | Association Between Corticosteroids and Outcomes in Children Hospitalized With Orbital Cellulitis | | | | 2022 | | pubmed | duplicate | duplicate |
| 18 | Chen L | Intravenous Steroids With Antibiotics on Admission for Children With Orbital Cellulitis | | | | 2018 | | pubmed | duplicate | duplicate |
| 19 | Richards DP | Iliotibial band Z-lengthening | | | | 2003 | | pubmed | title/abstract | irrelevant |
| 20 | Mahindra P | Chronic Plantar Fasciitis: Effect of Platelet-Rich Plasma, Corticosteroid, and Placebo | | | | 2016 | | pubmed | duplicate | duplicate |
| 21 | Hur K | Adjunct steroids in the treatment of peritonsillar abscess: A systematic review | | | | 2018 | | pubmed | title/abstract | review |
| 22 | Piekoszewska-Ziętek P | Developmental Abnormalities of Teeth in Children With Nephrotic Syndrome | | | | 2022 | | pubmed | title/abstract | irrelevant |
| 23 | Karakılıç GD | Prolotherapy Versus Phonophoresis and Corticosteroid Injections for the Treatment of Plantar Fasciitis: A Randomized, Double-Blind Clinical Trial | | | | 2023 | | pubmed | title/abstract | irrelevant |
| 24 | Rao MK | Fungal abscess after intra-orbital steroid injection: a case report | | | | 2022 | | pubmed | title/abstract | case report |
| 25 | Baumann GP | The Effects of Dexamethasone on the Time to Pain Resolution in Dental Periapical Abscess | | | | 2021 | | pubmed | fulltext | included |
| 26 | Singh P | A systematic review and meta-analysis of platelet-rich plasma versus corticosteroid injections for plantar fasciopathy | | | | 2017 | | pubmed | title/abstract | review |
| 27 | Fett N | Eosinophilic fasciitis: Current concepts | | | | 2018 | | pubmed | title/abstract | irrelevant |
| 28 | Espinoza GM | Orbital inflammatory pseudotumors: etiology, differential diagnosis, and management | | | | 2010 | | pubmed | title/abstract | irrelevant |
| 29 | Whittaker GA | Corticosteroid injection for plantar heel pain: a systematic review and meta-analysis | | | | 2019 | | pubmed | title/abstract | irrelevant |
| 30 | Afsahi V | Wells syndrome | | | | 2003 | | pubmed | title/abstract | irrelevant |
| 31 | Maheshwari R | Acute dacryocystitis causing orbital cellulitis and abscess | | | | 2009 | | pubmed | duplicate | duplicate |
| 32 | Gardner B | Plantar Fasciitis | | | | 2015 | | pubmed | duplicate | duplicate |
| 33 | Papavasileiou E | Ipilimumab-induced Ocular and Orbital Inflammation--A Case Series and Review of the Literature | | | | 2016 | | pubmed | title/abstract | irrelevant |
| 34 | Fu R | Progression of Subperiosteal Orbital Abscess after Clinical Resolution on Intravenous Antibiotics and Steroids | | | | 2023 | | pubmed | duplicate | duplicate |
| 35 | Sène D | [Eosinophilic fasciitis (Shulman's disease): Diagnostic and therapeutic review] | | | | 2015 | | pubmed | title/abstract | irrelevant |
| 36 | Tseng WC | The Comparative Effectiveness of Autologous Blood-derived Products Versus Steroid Injections in Plantar Fasciitis: A Systematic Review and Meta-analysis of Randomized Controlled Trials | | | | 2021 | | pubmed | title/abstract | irrelevant |
| 37 | Thomas J | Approach to treatment of refractory dissecting cellulitis of the scalp: a systematic review | | | | 2021 | | pubmed | title/abstract | irrelevant |
| 38 | Tansey JB | Dexamethasone Use in the Treatment of Pediatric Deep Neck Space Infections | | | | 2020 | | pubmed | title/abstract | irrelevant |
| 39 | Demirdover C | Necrotising fasciitis or pyoderma gangrenosum: A fatal dilemma | | | | 2019 | | pubmed | title/abstract | irrelevant |
| 40 | Fei X | Platelet-rich plasma has better mid-term clinical results than traditional steroid injection for plantar fasciitis: A systematic review and meta-analysis | | | | 2021 | | pubmed | title/abstract | irrelevant |
| 41 | López F | Cavernous sinus thrombosis during pregnancy | | | | 2017 | | pubmed | title/abstract | irrelevant |
| 42 | Page C | Parapharyngeal abscess: diagnosis and treatment | | | | 2008 | | pubmed | title/abstract | irrelevant |
| 43 | Bielsa Marsol I | Update on the classification and treatment of localized scleroderma | | | | 2013 | | pubmed | title/abstract | irrelevant |
| 44 | Dey R | Ciprofloxacin and dexamethasone in combination attenuate S. aureus induced brain abscess via neuroendocrine-immune interaction of TLR-2 and glucocorticoid receptor leading to behavioral improvement | | | | 2021 | | pubmed | title/abstract | irrelevant |
| 45 | Watanabe Y | A case of eosinophilic fasciitis and generalized morphea overlap | | | | 2020 | | pubmed | title/abstract | irrelevant |
| 46 | Moreno-Arquieta IA | Mycophenolate mofetil and mycophenolic acid for the treatment of eosinophilic fasciitis: report of two cases and literature review | | | | 2022 | | pubmed | title/abstract | irrelevant |
| 47 | Davies BW | C-Reactive Protein As a Marker for Initiating Steroid Treatment in Children With Orbital Cellulitis | | | | 2015 | | pubmed | duplicate | duplicate |
| 48 | Alfakeekh K | Immunosuppressive burden and risk factors of infection in primary childhood nephrotic syndrome | | | | 2019 | | pubmed | title/abstract | irrelevant |
| 49 | Lee DO | Comparing effectiveness of polydeoxyribonucleotide injection and corticosteroid injection in plantar fasciitis treatment: A prospective randomized clinical study | | | | 2020 | | pubmed | duplicate | duplicate |
| 50 | Yamagishi A | Prednisolone Treatment Is Effective for an Idiopathic Penile Abscess: A Case Report and Review | | | | 2021 | | pubmed | title/abstract | irrelevant |
| 51 | Zuelgaray E | [Sarcoid-like granulomatosis associated with eosinophilic fasciitis] | | | | 2018 | | pubmed | title/abstract | irrelevant |
| 52 | Lee AYS | A case of rash and arthralgias | | | | 2019 | | pubmed | title/abstract | case report |
| 53 | Chan KK | Eosinophilic Fasciitis Following Checkpoint Inhibitor Therapy: Four Cases and a Review of Literature | | | | 2020 | | pubmed | title/abstract | irrelevant |
| 54 | Suharwardy J | Periorbital necrotising fasciitis | | | | 1994 | | pubmed | title/abstract | irrelevant |
| 55 | Hooten WM | Epidural abscess and meningitis after epidural corticosteroid injection | | | | 2004 | | pubmed | title/abstract | irrelevant |
| 56 | Rastegar S | Comparison of dry needling and steroid injection in the treatment of plantar fasciitis: a single-blind randomized clinical trial | | | | 2018 | | pubmed | duplicate | duplicate |
| 57 | Koçak HE | Is corticosteroid a treatment choice for the management of peritonsillar abscess? | | | | 2018 | | pubmed | title/abstract | irrelevant outcome |
| 58 | Fonseca MES | Eosinophilic fasciitis during pregnancy: case report and review of literature | | | | 2018 | | pubmed | title/abstract | irrelevant |
| 59 | Lin HW | Ludwig's angina in the pediatric population | | | | 2009 | | pubmed | title/abstract | review |
| 60 | LaPonsie SA | When an Orbital Infection Isn't Infectious at All: A Review of Orbital Inflammatory Syndrome | | | | 2017 | | pubmed | title/abstract | case report |
| 61 | Jain K | Platelet rich plasma versus corticosteroid injection for plantar fasciitis: A comparative study | | | | 2015 | | pubmed | duplicate | duplicate |
| 62 | Sandini M | Intraoperative Dexamethasone Decreases Infectious Complications After Pancreaticoduodenectomy and is Associated with Long-Term Survival in Pancreatic Cancer | | | | 2018 | | pubmed | title/abstract | irrelevant |
| 63 | Peña-Martínez VM | Effect of corticosteroids over plantar fascia thickness in plantar fasciitis: a systematic review and meta-analysis | | | | 2024 | | pubmed | title/abstract | irrelevant |
| 64 | Hocaoglu S | Comparative Effectiveness of Radial Extracorporeal Shockwave Therapy and Ultrasound-Guided Local Corticosteroid Injection Treatment for Plantar Fasciitis | | | | 2017 | | pubmed | duplicate | duplicate |
| 65 | Ozawa H | Clinical Course of Eosinophilic Cellulitis | | | | 2017 | | pubmed | title/abstract | irrelevant |
| 66 | Aytekin K | Necrotizing Fasciitis After Total Hip Arthroplasty: A Rare Case Presentation | | | | 2022 | | pubmed | title/abstract | irrelevant |
| 67 | Thordarson DB | Highlight article: January-June 2013 | | | | 2013 | | pubmed | title/abstract | irrelevant |
| 68 | Williams SK | Heel pain-plantar fasciitis and Achilles enthesopathy | | | | 2004 | | pubmed | title/abstract | irrelevant |
| 69 | Romano N | Plantar pain is not always fasciitis | | | | 2017 | | pubmed | title/abstract | irrelevant |
| 70 | Bischoff L | Eosinophilic fasciitis: demographics, disease pattern and response to treatment: report of 12 cases and review of the literature | | | | 2008 | | pubmed | title/abstract | irrelevant |
| 71 | Liau MM | Pemetrexed-induced lower limb pseudocellulitis | | | | 2017 | | pubmed | title/abstract | case report |
| 72 | Murray M | Cervicofacial necrotizing fasciitis and steroids: case report and literature review | | | | 2012 | | pubmed | title/abstract | irrelevant |
| 73 | Choquet-Kastylevsky G | Eosinophilic fasciitis and simvastatin | | | | 2001 | | pubmed | title/abstract | case report |
| 74 | Yen MT | Effect of corticosteroids in the acute management of pediatric orbital cellulitis with subperiosteal abscess | | | | 2005 | | pubmed | fulltext | included |
| 75 | Schroeder KA | Effect of dexamethasone on experimental brain abscess | | | | 1987 | | pubmed | title/abstract | irrelevant |
| 76 | Au Eong DTM | Ultrasound in the diagnosis and monitoring of eosinophilic fasciitis | | | | 2021 | | pubmed | title/abstract | irrelevant |
| 77 | do Nascimento IV | Chronic systemic corticosteroid therapy influences the development of pulp necrosis and experimental apical periodontitis, exacerbating the inflammatory process and bone resorption in rats | | | | 2022 | | pubmed | title/abstract | Aimal study |
| 78 | Pushker N | Role of oral corticosteroids in orbital cellulitis | | | | 2013 | | pubmed | duplicate | duplicate |
| 79 | Thom C | Point-of-Care Ultrasound Identifies Pyomyositis Secondary to Intramuscular Testosterone Injection: Report of Two Cases | | | | 2022 | | pubmed | title/abstract | irrelevant |
| 80 | Canan H | Periocular Paederus dermatitis mimicking preseptal cellulitis | | | | 2013 | | pubmed | title/abstract | irrelevant |
| 81 | Cullen NP | Plantar fasciitis: a review | | | | 2006 | | pubmed | title/abstract | irrelevant |
| 82 | Ou Yang O | Eosinophilic Fasciitis - Beware of the Rare Form of Hand Contracture | | | | 2020 | | pubmed | title/abstract | irrelevant |
| 83 | Neuwelt EA | Effect of gentamicin and dexamethasone on the natural history of the rat Escherichia coli brain abscess model with histopathological correlation | | | | 1984 | | pubmed | title/abstract | irrelevant |
| 84 | Vandamme E | Eosinophilic Fasciitis Unmasking a Lung Cancer | | | | 2022 | | pubmed | title/abstract | irrelevant |
| 85 | Jain SK | Comparison of Plantar Fasciitis Injected With Platelet-Rich Plasma vs Corticosteroids | | | | 2018 | | pubmed | duplicate | duplicate |
| 86 | Roos DE | Randomized Comparison Radiation Therapy and Steroids for Plantar Fasciitis: In Regard to Canyilmaz et al | | | | 2016 | | pubmed | duplicate | duplicate |
| 87 | Miller JJ 3rd | The fasciitis-morphea complex in children | | | | 1992 | | pubmed | title/abstract | irrelevant |
| 88 | Albert-Fort M | A case report of orbital Langerhans cell histiocytosis presenting as a orbital cellulitis | | | | 2018 | | pubmed | title/abstract | case report |
| 89 | Elessa D | TNF-α antagonist infliximab for aseptic abscess syndrome | | | | 2019 | | pubmed | title/abstract | irrelevant |
| 90 | Hanselman AE | Cryopreserved human amniotic membrane injection for plantar fasciitis: a randomized, controlled, double-blind pilot study | | | | 2015 | | pubmed | duplicate | duplicate |
| 91 | Elizondo-Rodriguez J | A comparison of botulinum toxin a and intralesional steroids for the treatment of plantar fasciitis: a randomized, double-blinded study | | | | 2013 | | pubmed | duplicate | duplicate |
| 92 | Irie K | [A case of localized fasciitis with ulcerative colitis] | | | | 2022 | | pubmed | title/abstract | irrelevant |
| 93 | Rozenblat M | Wells' Syndrome Induced by Ustekinumab | | | | 2019 | | pubmed | title/abstract | irrelevant |
| 94 | Muller T | [Well's cellulitis: A case report] | | | | 2017 | | pubmed | title/abstract | irrelevant |
| 95 | Grady J | Extracorporeal Pulse-Activated Therapy versus Injection: Treatment of Recalcitrant Plantar Fasciitis | | | | 2019 | | pubmed | title/abstract | irrelevant |
| 96 | Han Y | Nocardiosis in glomerular disease patients with immunosuppressive therapy | | | | 2020 | | pubmed | title/abstract | irrelevant |
| 97 | Jinnin M | Diagnostic criteria, severity classification and guidelines of eosinophilic fasciitis | | | | 2018 | | pubmed | title/abstract | irrelevant |
| 98 | MacInnes A | Long-Term Outcome of Open Plantar Fascia Release | | | | 2016 | | pubmed | title/abstract | irrelevant |
| 99 | Brameli A | Systemic corticosteroids may be beneficial for managing severe or refractory orbital cellulitis in children | | | | 2018 | | pubmed | fulltext | included |
| 100 | Garelli V | Dissecting cellulitis: responding to topical steroid and oral clindamycin | | | | 2017 | | pubmed | title/abstract | irrelevant |
| 101 | Mardani-Kivi M | Treatment Outcomes of Corticosteroid Injection and Extracorporeal Shock Wave Therapy as Two Primary Therapeutic Methods for Acute Plantar Fasciitis: A Prospective Randomized Clinical Trial | | | | 2015 | | pubmed | duplicate | duplicate |
| 102 | Kim DS | Idiopathic orbital myositis mimicking orbital cellulitis | | | | 2010 | | pubmed | title/abstract | irrelevant |
| 103 | McMillan AM | Ultrasound guided corticosteroid injection for plantar fasciitis: randomised controlled trial | | | | 2012 | | pubmed | duplicate | duplicate |
| 104 | Mahalingam S | The management of periorbital cellulitis secondary to sinonasal infection: a multicenter prospective study in the United Kingdom | | | | 2020 | | pubmed | title/abstract | irrelevant |
| 105 | Yu AM | Pediatric Wells syndrome (eosinophilic cellulitis) after vaccination: A case report and review of the literature | | | | 2018 | | pubmed | title/abstract | irrelevant |
| 106 | Crespo R | Facial cellulitis induced in chickens by Mycoplasma gallisepticum bacterin and its treatment | | | | 2008 | | pubmed | fulltext | Aimal study |
| 107 | Matsuhisa Y | Cellulitis caused by Roseomonas mucosa in a child: a case report | | | | 2023 | | pubmed | title/abstract | irrelevant |
| 108 | Chaigne B | Cluster analysis reveals eosinophilia and fibrosis as poor prognostic markers in 128 patients with eosinophilic fasciitis | | | | 2022 | | pubmed | title/abstract | irrelevant |
| 109 | Bae KH | Orbital Cellulitis from an Orbital Compressed Air and Diesel Explosion Injury | | | | 2018 | | pubmed | fulltext | letter to editor |
| 110 | Kirkland P | Use of primary corticosteroid injection in the management of plantar fasciopathy: is it time to challenge existing practice? | | | | 2013 | | pubmed | title/abstract | irrelevant |
| 111 | Osborne HR | Treatment of plantar fasciitis by LowDye taping and iontophoresis: short term results of a double blinded, randomised, placebo controlled clinical trial of dexamethasone and acetic acid | | | | 2006 | | pubmed | duplicate | duplicate |
| 112 | Norman T | Lucio phenomenon with concomitant necrotizing fasciitis and acute kidney injury | | | | 2022 | | pubmed | title/abstract | irrelevant |
| 113 | Wallace RJ Jr | Skin, soft tissue, and bone infections due to Mycobacterium chelonae chelonae: importance of prior corticosteroid therapy, frequency of disseminated infections, and resistance to oral antimicrobials other than clarithromycin | | | | 1992 | | pubmed | title/abstract | irrelevant |
| 114 | Manzini CU | D-penicillamine in the treatment of eosinophilic fasciitis: case reports and review of the literature | | | | 2012 | | pubmed | title/abstract | irrelevant |
| 115 | Riel H | Corticosteroid injection plus exercise versus exercise, beyond advice and a heel cup for patients with plantar fasciopathy: protocol for a randomised clinical superiority trial (the FIX-Heel trial) | | | | 2020 | | pubmed | title/abstract | irrelevant |
| 116 | Lieberman JA | Wells Syndrome with Bullous Lesions | | | | 2017 | | pubmed | title/abstract | irrelevant |
| 117 | Canyilmaz E | Prospective Randomized Comparison of the Effectiveness of Radiation Therapy and Local Steroid Injection for the Treatment of Plantar Fasciitis | | | | 2015 | | pubmed | duplicate | duplicate |
| 118 | Laszlo KS | Idiopathic palmar fasciitis | | | | 1995 | | pubmed | title/abstract | irrelevant |
| 119 | Nougué H | Clinical and imaging factors associated with severe complications of cervical necrotizing fasciitis | | | | 2015 | | pubmed | title/abstract | irrelevant |
| 120 | Yamazaki H | Application of ultrasound in a case of eosinophilic fasciitis mimicking stiff-person syndrome | | | | 2022 | | pubmed | title/abstract | irrelevant |
| 121 | Sheehan JL | A Case of Recurrent Hepatic Abscesses | | | | 2021 | | pubmed | title/abstract | irrelevant |
| 122 | Ermutlu C | Thickness of plantar fascia is not predictive of functional outcome in plantar fasciitis treatment | | | | 2018 | | pubmed | title/abstract | irrelevant |
| 123 | Acosta-Olivo C | Plantar Fasciitis-A Comparison of Treatment with Intralesional Steroids versus Platelet-Rich Plasma (A Randomized, Blinded Study) | | | | 2017 | | pubmed | title/abstract | irrelevant |
| 124 | Chen X | Central Retinal Artery Occlusion Due to Subperiosteal Orbital Abscess Caused by Acute Sinusitis in a Child: A Case Report | | | | 2023 | | pubmed | title/abstract | irrelevant |
| 125 | Krishna K | Palmar fasciitis with polyarthritis syndrome in a patient with breast cancer | | | | 2011 | | pubmed | title/abstract | irrelevant |
| 126 | Keren S | The management of periorbital nodular fasciitis using intra-lesional triamcinolone: a case report and review of the literature | | | | 2021 | | pubmed | title/abstract | case report |
| 127 | Chen L | Reply re: "Intravenous Steroids With Antibiotics on Admission for Children With Orbital Cellulitis" | | | | 2017 | | pubmed | title/abstract | letter to editor |
| 128 | Jiménez-Pérez AE | Clinical and imaging effects of corticosteroids and platelet-rich plasma for the treatment of chronic plantar fasciitis: A comparative non randomized prospective study | | | | 2019 | | pubmed | title/abstract | irrelevant |
| 129 | Kalaci A | Treatment of plantar fasciitis using four different local injection modalities: a randomized prospective clinical trial | | | | 2009 | | pubmed | duplicate | duplicate |
| 130 | Lee HS | Risk factors affecting chronic rupture of the plantar fascia | | | | 2014 | | pubmed | title/abstract | irrelevant |
| 131 | Chawla B | MRI in retinoblastoma with orbital cellulitis | | | | 2013 | | pubmed | title/abstract | irrelevant |
| 132 | Bobrowska-Snarska D | [Eosinophilic fasciitis--diagnostic and therapeutic difficulties] | | | | 2008 | | pubmed | title/abstract | irrelevant |
| 133 | Rhatigan M | Orbital abscess following posterior subtenon injection of triamcinolone acetonide | | | | 2017 | | pubmed | duplicate | duplicate |
| 134 | Hanna BC | Corticosteroids and peritonsillar abscess formation in infectious mononucleosis | | | | 2004 | | pubmed | title/abstract | irrelevant |
| 135 | El-Jammal T | Eosinophilic Fasciitis and Common Variable Immunodeficiency: An Unusual Association and Literature Review | | | | 2019 | | pubmed | title/abstract | irrelevant |
| 136 | Bajwa J | Juvenile cellulitis (juvenile sterile granulomatous dermatitis and lymphadenitis) in a 9-week-old puppy treated with prednisolone-cyclosporine combination therapy | | | | 2022 | | pubmed | title/abstract | irrelevant |
| 137 | Kamal S | Primary Powered Endoscopic Dacryocystorhinostomy in the Setting of Acute Dacryocystitis and Lacrimal Abscess | | | | 2015 | | pubmed | title/abstract | irrelevant |
| 138 | Downard CD | Treatment of congenital pulmonary airway malformations: a systematic review from the APSA outcomes and evidence based practice committee | | | | 2017 | | pubmed | title/abstract | irrelevant |
| 139 | Erez D | Clinical experience with biologic treatment in resistant eosinophilic fasciitis: Case reports and review of the literature | | | | 2021 | | pubmed | title/abstract | irrelevant |
| 140 | Teke TA | Idiopathic Orbital Inflammation in a Child Mimicking Orbital Cellulitis | | | | 2016 | | pubmed | title/abstract | case report |
| 141 | Moore GH | Orbital Relapsing Polychondritis: A Unique Presentation, Complication, and Treatment | | | | 2016 | | pubmed | duplicate | duplicate |
| 142 | Tsai WC | Treatment of proximal plantar fasciitis with ultrasound-guided steroid injection | | | | 2000 | | pubmed | title/abstract | irrelevant |
| 143 | Kane D | The role of ultrasonography in the diagnosis and management of idiopathic plantar fasciitis | | | | 2001 | | pubmed | title/abstract | irrelevant |
| 144 | Morita T | Necrotising fasciitis after bortezomib and dexamethasone-containing regimen in an elderly patient of Waldenström macroglobulinaemia | | | | 2014 | | pubmed | title/abstract | irrelevant |
| 145 | Asaoka K | A case of eosinophilic fasciitis without skin manifestations: a case report in a patient with lupus and literature review | | | | 2021 | | pubmed | title/abstract | irrelevant |
| 146 | Hohmeister R | [Eosinophilic fasciitis] | | | | 1982 | | pubmed | title/abstract | irrelevant |
| 147 | Ramakrishnan P | Cysticercosis of the masseter | | | | 2012 | | pubmed | title/abstract | case report |
| 148 | Fanfarillo F | Necrotizing fasciitis following intra-articular steroid injection: case report and review of the literature | | | | 2012 | | pubmed | title/abstract | irrelevant |
| 149 | Sheridan L | Plantar fasciopathy treated with dynamic splinting: a randomized controlled trial | | | | 2010 | | pubmed | duplicate | duplicate |
| 150 | Poignet B | Orbital abscess following sub-Tenon's corticosteroid injection: A rare but severe complication | | | | 2024 | | pubmed | title/abstract | case report |
| 151 | Chau JK | Corticosteroids in peritonsillar abscess treatment: a blinded placebo-controlled clinical trial | | | | 2014 | | pubmed | title/abstract | irrelevant |
| 152 | Gallard C | [Wells syndrome mimicking facial cellulitis: Three cases] | | | | 2017 | | pubmed | title/abstract | irrelevant |
| 153 | Johannsen F | Endoscopic fasciotomy for plantar fasciitis provides superior results when compared to a controlled non-operative treatment protocol: a randomized controlled trial | | | | 2020 | | pubmed | title/abstract | irrelevant |
| 154 | Trenti L | Long-term evolution of acute colonic diverticulitis after successful medical treatment | | | | 2015 | | pubmed | title/abstract | irrelevant |
| 155 | Schnabel A | [Tryptophan-induced fasciitis-scleroderma-eosinophilia syndrome] | | | | 1991 | | pubmed | title/abstract | irrelevant |
| 156 | Kumar R | Paradoxical response to chemotherapy in neurotuberculosis | | | | 2006 | | pubmed | title/abstract | irrelevant |
| 157 | Kromer C | Response of eosinophilic fasciitis associated with Waldenström macroglobulinemia to rituximab | | | | 2021 | | pubmed | title/abstract | irrelevant |
| 158 | Issing P | Schnellere Schmerzlinderung mit adjuvanten Steroiden | | | | 2014 | | pubmed | title/abstract | irrelevant |
| 159 | Mason DA | Steroid therapy and dental infection. Case report | | | | 1970 | | pubmed | title/abstract | case report |
| 160 | Campbell AA | Re: "Intravenous Steroids With Antibiotics on Admission for Children With Orbital Cellulitis" | | | | 2017 | | pubmed | title/abstract | letter to editor |
| 161 | Liu M | Red-brown plaque on the leg | | | | 2018 | | pubmed | title/abstract | irrelevant |
| 162 | Bachmeyer C | Facial lesions heralding the onset and relapse of eosinophilic fasciitis | | | | 2016 | | pubmed | title/abstract | irrelevant |
| 163 | Renfrow JJ | Fungal Contamination of Methylprednisolone Causing Recurrent Lumbosacral Intradural Abscess | | | | 2017 | | pubmed | title/abstract | irrelevant |
| 164 | Fisher GB | Eosinophilic cellulitis (Wells' syndrome) | | | | 1985 | | pubmed | title/abstract | irrelevant |
| 165 | Chiu ES | Successful management of orbital cellulitis and temporary visual loss after blepharoplasty | | | | 2006 | | pubmed | duplicate | duplicate |
| 166 | Chen HM | Facial Candida albicans cellulitis occurring in a patient with oral submucous fibrosis and unknown diabetes mellitus after local corticosteroid injection treatment | | | | 2004 | | pubmed | title/abstract | case report |
| 167 | Rossiter-Thornton M | Posterior scleritis mimicking orbital cellulitis | | | | 2010 | | pubmed | duplicate | duplicate |
| 168 | Tabrizi A | The Effect of Corticosteroid Local Injection Versus Platelet-Rich Plasma for the Treatment of Plantar Fasciitis in Obese Patients: A Single-Blind, Randomized Clinical Trial | | | | 2020 | | pubmed | duplicate | duplicate |
| 169 | Ma J | Diagnosis and treatment of early eosinophilic fasciitis: a case report | | | | 2025 | | pubmed | title/abstract | irrelevant |
| 170 | Bilewicz-Stebel M | Eosinophilic Fasciitis - Clinical Features and Therapeutic Management | | | | 2020 | | pubmed | title/abstract | irrelevant |
| 171 | Huang JW | Necrotizing fasciitis caused by Serratia marcescens in two patients receiving corticosteroid therapy | | | | 1999 | | pubmed | title/abstract | irrelevant |
| 172 | Gurcay E | Shall We Inject Superficial or Deep to the Plantar Fascia? An Ultrasound Study of the Treatment of Chronic Plantar Fasciitis | | | | 2017 | | pubmed | title/abstract | irrelevant |
| 173 | Cho J | Diffuse Exfoliative Rash with Sepsis and Eosinophilia: A Case of Erythroderma? | | | | 2019 | | pubmed | title/abstract | irrelevant |
| 174 | Kim C | Incidence of plantar fascia ruptures following corticosteroid injection | | | | 2010 | | pubmed | title/abstract | irrelevant |
| 175 | Sullivan C | Eosinophilic fasciitis in siblings | | | | 2013 | | pubmed | title/abstract | irrelevant |
| 176 | Soo MRT | Infection-Associated Acquired Hemophilia and False-Positive Dengue: A Case Report | | | | 2021 | | pubmed | title/abstract | irrelevant |
| 177 | Partarrieu-Mejías F | Steroid-resistant eosinophilic fasciitis successfully treated with addition of extracorporeal photopheresis | | | | 2019 | | pubmed | title/abstract | irrelevant |
| 178 | Kamel M | High frequency ultrasonographic findings in plantar fasciitis and assessment of local steroid injection | | | | 2000 | | pubmed | title/abstract | irrelevant |
| 179 | Ģībietis V | Epidural Abscesses as a Complication of Interleukin-6 Inhibitor and Dexamethasone Treatment in a Patient with COVID-19 Pneumonia: A Case Report | | | | 2023 | | pubmed | title/abstract | irrelevant |
| 180 | Dinh H | Sweet's syndrome associated with cellulitis | | | | 2007 | | pubmed | title/abstract | irrelevant |
| 181 | Chandler TJ | Iontophoresis of 0.4% dexamethasone for plantar fasciitis | | | | 1998 | | pubmed | title/abstract | irrelevant |
| 182 | Wessling H | Cervicothoracolumbar spinal epidural abscess with tetraparesis. Good recovery after non-surgical treatment with antibiotics and dexamethasone. Case report and review of the literature | | | | 2003 | | pubmed | title/abstract | irrelevant |
| 183 | Radvan GH | Diffuse fasciitis with eosinophilia (Shulman's disease) | | | | 1979 | | pubmed | title/abstract | irrelevant |
| 184 | Shah N | Acute horseshoe abscess of the hand after corticosteroid injection to treat trigger thumb | | | | 2018 | | pubmed | title/abstract | irrelevant |
| 185 | Shetty VD | A study to compare the efficacy of corticosteroid therapy with platelet-rich plasma therapy in recalcitrant plantar fasciitis: a preliminary report | | | | 2014 | | pubmed | duplicate | duplicate |
| 186 | Yildizhan A | Effect of dexamethasone on various stages of experimental brain abscess | | | | 1989 | | pubmed | title/abstract | irrelevant |
| 187 | Buccilli TA Jr | Sterile abscess formation following a corticosteroid injection for the treatment of plantar fasciitis | | | | 2005 | | pubmed | title/abstract | irrelevant |
| 188 | Grisanti MW | Eosinophilic fasciitis in children | | | | 1989 | | pubmed | title/abstract | irrelevant |
| 189 | Inga A | Sterile granulomatous dermatitis and lymphadenitis (juvenile cellulitis) in adult dogs: a retrospective analysis of 90 cases (2004-2018) | | | | 2020 | | pubmed | title/abstract | irrelevant |
| 190 | Tang MM | Oral mucosal morphea: a new variant | | | | 2012 | | pubmed | title/abstract | irrelevant |
| 191 | Martens SM | Juvenile cellulitis in a 7-week-old golden retriever dog | | | | 2016 | | pubmed | title/abstract | irrelevant |
| 192 | Hamashige J | Necrotizing fasciitis following minor skin surgery in a patient receiving treatment with infliximab and prednisolone | | | | 2018 | | pubmed | title/abstract | irrelevant |
| 193 | Canyilmaz E | In Reply to Roos and Smith and an Erratum | | | | 2016 | | pubmed | title/abstract | irrelevant |
| 194 | Bolac CS | The impact of postoperative nausea and vomiting prophylaxis with dexamethasone on postoperative wound complications in patients undergoing laparotomy for endometrial cancer | | | | 2013 | | pubmed | title/abstract | irrelevant |
| 195 | Golitz LE | Fasciitis with eosinophilia: The Shulman syndrome | | | | 1980 | | pubmed | title/abstract | irrelevant |
| 196 | Kivlin JD | Periocular infection after strabismus surgery. The Periocular Infection Study Group | | | | 1995 | | pubmed | title/abstract | irrelevant |
| 197 | Gudeman SD | Treatment of plantar fasciitis by iontophoresis of 0.4% dexamethasone. A randomized, double-blind, placebo-controlled study | | | | 1997 | | pubmed | title/abstract | irrelevant |
| 198 | Papa R | Juvenile eosinophilic fasciitis: three case reports with review of the literature | | | | 2016 | | pubmed | title/abstract | irrelevant |
| 199 | Kaikkonen M | [Treatment of plantar fasciopathy] | | | | 2012 | | pubmed | title/abstract | irrelevant |
| 200 | Asada S | A Histologically Proven Case of Autoimmune Hepatitis with Eosinophilic Fasciitis | | | | 2019 | | pubmed | title/abstract | irrelevant |
| 201 | Zhang CC | Skin abscesses, X-linked agammaglobulinaemia and spastic paraplegias in a male patient | | | | 2022 | | pubmed | title/abstract | irrelevant |
| 202 | Frenkel A | Estimations of a degree of steroid induced leukocytosis in patients with acute infections | | | | 2018 | | pubmed | title/abstract | irrelevant |
| 203 | Kakeya H | Concurrent subcutaneous candidal abscesses and pulmonary cryptococcosis in a patient with diabetes mellitus and a history of corticosteroid therapy | | | | 2014 | | pubmed | title/abstract | irrelevant |
| 204 | Mendoza FA | Severe eosinophilic fasciitis: comparison of treatment with D-penicillamine plus corticosteroids vs. corticosteroids alone | | | | 2016 | | pubmed | title/abstract | irrelevant |
| 205 | Bhatti Z | Submental Abscess After Deoxycholic Acid Injection | | | | 2018 | | pubmed | title/abstract | irrelevant |
| 206 | Shamriz O | Eosinophilic Fasciitis: A Single Center Experience of Seven Patients | | | | 2018 | | pubmed | title/abstract | irrelevant |
| 207 | Paşaoğlu A | Treatment of experimental brain abscess. 2. Effects of combinations of hyaluronidase with antibiotics and dexamethasone | | | | 1989 | | pubmed | title/abstract | irrelevant |
| 208 | ROSS IP | Nephrotic syndrome; prednisone therapy with two deaths from cellulitis | | | | 1957 | | pubmed | title/abstract | irrelevant |
| 209 | Armesto A | Orbital cellulitis after faden operation on the medial rectus | | | | 2007 | | pubmed | duplicate | duplicate |
| 210 | Huff GR | Dexamethasone immunosuppression resulting in turkey clostridial dermatitis: a retrospective analysis of seven studies, 1998-2009 | | | | 2013 | | pubmed | title/abstract | irrelevant |
| 211 | Zhang G | Eosinophilic fasciitis following postpartum: A rare case report | | | | 2025 | | pubmed | title/abstract | irrelevant |
| 212 | Yoon J | Primary cicatricial alopecia in a single-race Asian population: A 10-year nationwide population-based study in South Korea | | | | 2018 | | pubmed | title/abstract | irrelevant |
| 213 | Shinozaki A | Efficacy of methotrexate for steroid-resistant eosinophilic fasciitis with delayed start of treatment: a case report | | | | 2023 | | pubmed | title/abstract | irrelevant |
| 214 | Uehara Y | Fasciitis-panniculitis syndrome with autoantibodies reacting to adipocyte pericellular fibers: a case report | | | | 2025 | | pubmed | title/abstract | irrelevant |
| 215 | Alexanian C | Eosinophilic fasciitis presenting as a unilateral, solitary plaque | | | | 2019 | | pubmed | title/abstract | irrelevant |
| 216 | Bobrowska-Snarska D | [Fasciitis eosinophilica: personal observations and a review of the literature] | | | | 2007 | | pubmed | title/abstract | irrelevant |
| 217 | Koh KJ | Wells' syndrome following thiomersal-containing vaccinations | | | | 2003 | | pubmed | title/abstract | irrelevant |
| 218 | Liou CH | Eosinophilic fasciitis in a military recruit: MRI evaluation with clinical correlation | | | | 2003 | | pubmed | title/abstract | irrelevant |
| 219 | Sickler SJ | Group B streptococcal cellulitis in a child with steroid-responsive nephrotic syndrome | | | | 2001 | | pubmed | title/abstract | irrelevant |
| 220 | Odhav A | Pansclerotic morphea with features of eosinophilic fasciitis: distinct entities or part of a continuum? | | | | 2014 | | pubmed | title/abstract | irrelevant |
| 221 | Sorrentino F | Role of high-resolution ultrasound in guiding treatment of idiopathic plantar fasciitis with minimally invasive techniques | | | | 2008 | | pubmed | duplicate | duplicate |
| 222 | Kuo CL | Late onset lamellar keratitis and epithelial ingrowth following orbital cellulitis | | | | 2006 | | pubmed | duplicate | duplicate |
| 223 | Chigwanda PC | A prospective study of Plantar fasciitis in Harare | | | | 1997 | | pubmed | title/abstract | irrelevant |
| 224 | Yoshii Y | Wells' Syndrome Associated with Coxsackievirus A6 Infection | | | | 2018 | | pubmed | title/abstract | irrelevant |
| 225 | Muzzi E | Bilateral orbital preseptal cellulitis after combined adenotonsillectomy and strabismus surgery--case report and pathogenetic hypothesis | | | | 2013 | | pubmed | title/abstract | irrelevant |
| 226 | Endo Y | Eosinophilic fasciitis: report of two cases and a systematic review of the literature dealing with clinical variables that predict outcome | | | | 2007 | | pubmed | title/abstract | irrelevant |
| 227 | Ortega-Loayza AG | Eosinophilic fasciitis in a female child | | | | 2008 | | pubmed | title/abstract | irrelevant |
| 228 | Park C | Combination of cyclosporin A and prednisolone for juvenile cellulitis concurrent with hindlimb paresis in 3 English cocker spaniel puppies | | | | 2010 | | pubmed | title/abstract | irrelevant |
| 229 | Olson RK | Cluster of postinjection abscesses related to corticosteroid injections and use of benzalkonium chloride | | | | 1999 | | pubmed | title/abstract | irrelevant |
| 230 | Hui JY | Over-the-counter medication and its effects | | | | 2002 | | pubmed | title/abstract | irrelevant |
| 231 | Salvi AE | Targeting the Plantar Fascia for Corticosteroid Injection | | | | 2015 | | pubmed | title/abstract | irrelevant |
| 232 | Hutchings SM | Juvenile cellulitis in a puppy | | | | 2003 | | pubmed | title/abstract | irrelevant |
| 233 | Bassett RJ | Juvenile cellulitis in an 8-month-old dog | | | | 2005 | | pubmed | title/abstract | irrelevant |
| 234 | Slocum AMY | A surgeon's nightmare: pyoderma gangrenosum with pathergy effect mimicking necrotising fasciitis | | | | 2017 | | pubmed | title/abstract | irrelevant |
| 235 | Moriguchi M | Eosinophilic fasciitis complicated with peripheral polyneuropathy | | | | 1998 | | pubmed | title/abstract | irrelevant |
| 236 | Schott CK | A pain in the neck: non-traumatic adult retropharyngeal abscess | | | | 2013 | | pubmed | title/abstract | irrelevant |
| 237 | Gülmez I | Effect of testicular torsion on the contralateral testis and prevention of this effect by prednisolone | | | | 1987 | | pubmed | title/abstract | irrelevant |
| 238 | Verenes M | Neuromuscular ultrasound findings in eosinophilic fasciitis: A case series and literature review | | | | 2018 | | pubmed | title/abstract | irrelevant |
| 239 | Alolabi B | Forearm compartment syndrome as a result of eosinophilic fasciitis: case report | | | | 2015 | | pubmed | title/abstract | irrelevant |
| 240 | Weber HO | Eosinophilic fasciitis and combined UVA1--retinoid--corticosteroid treatment: two case reports | | | | 2008 | | pubmed | title/abstract | irrelevant |
| 241 | Husain A | Nodular fasciitis presenting in an adult woman | | | | 2011 | | pubmed | title/abstract | irrelevant |
| 242 | Tsuruta Y | A case of dermato-fasciitis: amyopathic dermatomyositis associated with fasciitis | | | | 2004 | | pubmed | title/abstract | irrelevant |
| 243 | Quinn M | Ultrasound guided injection of plantar fasciitis | | | | 1998 | | pubmed | duplicate | duplicate |
| 244 | Ge R | Platelet rich plasma versus glucocorticoid for plantar fasciitis: A protocol for systematic review and meta-analysis of randomized controlled trials | | | | 2021 | | pubmed | title/abstract | irrelevant |
| 245 | Borderie VM | Endophthalmitis after Lasiodiplodia theobromae corneal abscess | | | | 1997 | | pubmed | title/abstract | irrelevant |
| 246 | Lee JH | Necrotising fasciitis by steroid-induced Sweet's syndrome: a case report | | | | 2016 | | pubmed | title/abstract | irrelevant |
| 247 | Takahashi A | Iliopsoas and intraperitoneal abscesses associated with pyoderma gangrenosum | | | | 2017 | | pubmed | title/abstract | irrelevant |
| 248 | Wyner D | A Family and Hospital's Journey and Commitment to Improving Diagnostic Safety | | | | 2021 | | pubmed | title/abstract | irrelevant |
| 249 | Nakamura T | Polyarteritis nodosa limited to calf muscles: a case report and review of the literature | | | | 2003 | | pubmed | title/abstract | irrelevant |
| 250 | Anupama B | Plantar fasciitis and impaired vision: A case report | | | | 2010 | | pubmed | title/abstract | irrelevant |
| 251 | Kayhan A | Sonographically guided corticosteroid injection for treatment of plantar fasciosis | | | | 2011 | | pubmed | title/abstract | irrelevant |
| 252 | Pradeep TG | Diffuse bilateral orbital inflammation in Churg- Strauss syndrome | | | | 2010 | | pubmed | title/abstract | irrelevant |
| 253 | Oh IK | Periocular abscess caused by Pseudallescheria boydii after a posterior subtenon injection of triamcinolone acetonide | | | | 2007 | | pubmed | title/abstract | irrelevant |
| 254 | Listernick R | A 13-Year-Old Boy with Pancytopenia and a Sacrococcygeal Abscess | | | | 2015 | | pubmed | title/abstract | irrelevant |
| 255 | Cardoso CL | Rosai-Dorfman disease with widespread oral-maxillofacial manifestations: a case report | | | | 2012 | | pubmed | title/abstract | irrelevant |
| 256 | Covey CJ | Plantar fasciitis: How best to treat? | | | | 2013 | | pubmed | title/abstract | irrelevant |
| 257 | Probst C | Cryptococcosis mimicking cutaneous cellulitis in a patient suffering from rheumatoid arthritis: a case report | | | | 2010 | | pubmed | title/abstract | irrelevant |
| 258 | Ryan M | Comparison of a physiotherapy program versus dexamethasone injections for plantar fasciopathy in prolonged standing workers: a randomized clinical trial | | | | 2014 | | pubmed | duplicate | duplicate |
| 259 | Danan J | Periorbital Necrotizing Fasciitis Following Dexamethasone Intravitreal Implant Injection | | | | 2016 | | pubmed | duplicate | duplicate |
| 260 | Andreopoulos A | Eosinophilic fasciitis accompanied by serositis | | | | 2009 | | pubmed | title/abstract | irrelevant |
| 261 | Dowden AM | Eosinophilic fasciitis masquerading as angioedema | | | | 2009 | | pubmed | title/abstract | irrelevant |
| 262 | Takahashi S | [A Case of Penile Pyoderma Gangrenosum Treated with Steroid Administration without Penectomy] | | | | 2019 | | pubmed | title/abstract | irrelevant |
| 263 | Patrone NA | Eosinophilic fasciitis in a child | | | | 1984 | | pubmed | title/abstract | irrelevant |
| 264 | Hashimoto Y | Polymerase chain reaction of Borrelia burgdorferi flagellin gene in Shulman syndrome | | | | 1996 | | pubmed | title/abstract | irrelevant |
| 265 | Naguwa SM | Eosinophilic fasciitis: a distinct clinical entity? | | | | 1983 | | pubmed | title/abstract | irrelevant |
| 266 | Marie-Cardine A | [Severe cutaneous Streptococcus pyogenes infections in the child: results of a multicenter survey] | | | | 2001 | | pubmed | title/abstract | irrelevant |
| 267 | DEANDRADE JR | SMALL DOSES OF PREDNISOLONE IN THE MANAGEMENT OF RHEUMATOID ARTHRITIS | | | | 1964 | | pubmed | title/abstract | irrelevant |
| 268 | Nakanishi K | Mimicker of necrotising fasciitis with systemic inflammatory response syndrome: recurrent necrotising Sweet's syndrome associated with chronic myelogenous leukaemia | | | | 2016 | | pubmed | title/abstract | irrelevant |
| 269 | Niskanen L | [Eosinophilic fasciitis] | | | | 1987 | | pubmed | title/abstract | irrelevant |
| 270 | Rajpara A | Recurrent paraneoplastic wells syndrome in a patient with metastatic renal cell cancer | | | | 2014 | | pubmed | title/abstract | irrelevant |
| 271 | Oshitari K | [Sweet syndrome presenting as orbital cellulitis] | | | | 2004 | | pubmed | title/abstract | case report |
| 272 | Withrow RA | Cellulitis unresponsive to antibiotics. Sweet's syndrome | | | | 2011 | | pubmed | title/abstract | irrelevant |
| 273 | Uehara F | Diagnostic imaging in patients with orbital cellulitis and inflammatory pseudotumor | | | | 2002 | | pubmed | title/abstract | irrelevant |
| 274 | Moon SH | Bullous eosinophilic cellulitis in a child treated with dapsone | | | | 2013 | | pubmed | title/abstract | irrelevant |
| 275 | Bento-Rodrigues J | Necrotizing Faciitis after shoulder mobilization and intra-articular infiltration with betametasone | | | | 2013 | | pubmed | title/abstract | irrelevant |
| 276 | Schulte RM | [Glucocorticoids--pro and contra. Possibilities for use of dexamethasone in neurology and neurosurgery (II)] | | | | 1983 | | pubmed | title/abstract | irrelevant |
| 277 | Falanga V | Increased plasma histamine level in eosinophilic fasciitis | | | | 1989 | | pubmed | title/abstract | irrelevant |
| 278 | Amdur HS | Eosinophilic fasciitis during pregnancy | | | | 1989 | | pubmed | title/abstract | irrelevant |
| 279 | Rosenfeld K | Eosinophilic fasciitis in a father and son | | | | 1994 | | pubmed | title/abstract | irrelevant |
| 280 | Schmitt NJ | Superior ophthalmic vein thrombosis in a patient with dacryocystitis-induced orbital cellulitis | | | | 2005 | | pubmed | title/abstract | irrelevant |
| 281 | Juthani V | Successful management of methicillin-resistant Staphylococcus aureus orbital cellulitis after blepharoplasty | | | | 2010 | | pubmed | duplicate | duplicate |
| 282 | Rutar T | Bilateral blindness from orbital cellulitis caused by community-acquired methicillin-resistant Staphylococcus aureus | | | | 2005 | | pubmed | duplicate | duplicate |
| 283 | Harris MS | A swollen right eye in a child | | | | 2015 | | pubmed | duplicate | duplicate |
| 284 | Ali K | Facial tuberculoid leprosy: case report | | | | 2011 | | pubmed | title/abstract | irrelevant |
| 285 | Ang LP | Orbital cellulitis following intralesional corticosteroid injection for periocular capillary haemangioma | | | | 2007 | | pubmed | title/abstract | letter to editor |
| 286 | Knox RJ | Recurrent orbital inflammation from metastatic orbital carcinoid tumor | | | | 2001 | | pubmed | title/abstract | irrelevant |
| 287 | Birkinshaw R | Necrotising fasciitis as a complication of steroid injection | | | | 1997 | | pubmed | title/abstract | irrelevant |
| 288 | Berianu F | Sarcoid Fasciitis With Magnetic Resonance Imaging Fascial Enhancement | | | | 2016 | | pubmed | title/abstract | irrelevant |
| 289 | Killen JW | Eosinophilic fasciitis with pulmonary and pleural involvement | | | | 2000 | | pubmed | title/abstract | irrelevant |
| 290 | Dasgupta B | Scintigraphic localisation of steroid injection site in plantar fasciitis | | | | 1995 | | pubmed | title/abstract | irrelevant |
| 291 | Kato T | Therapeutic efficacy of intravenous cyclophosphamide concomitant with moderate- to high-dose prednisolone in two patients with fasciitis panniculitis syndrome | | | | 2008 | | pubmed | title/abstract | irrelevant |
| 292 | Johnson JL | Pyoderma gangrenosum associated with an aseptic splenic abscess in a patient with neurofibromatosis | | | | 2015 | | pubmed | title/abstract | irrelevant |
| 293 | Blaser KU | [Eosinophilic fasciitis with aplastic anemia and Hashimoto's thyroiditis. Review of the literature and report of a typical example] | | | | 1989 | | pubmed | title/abstract | irrelevant |
| 294 | Neuber AE | Dermatitis and lymphadenitis resembling juvenile cellulitis in a four-year-old dog | | | | 2004 | | pubmed | title/abstract | irrelevant |
| 295 | Valencia IC | Eosinophilic fasciitis responsive to treatment with pulsed steroids and cyclosporine | | | | 1999 | | pubmed | title/abstract | irrelevant |
| 296 | Kan Y | Eosinophilic fasciitis in a 2-year-old child treated with a combination of methotrexate and corticosteroids | | | | 2019 | | pubmed | title/abstract | irrelevant |
| 297 | May AK | Steroids in the treatment of group A streptococcal necrotizing soft tissue infection | | | | 2011 | | pubmed | title/abstract | irrelevant |
| 298 | Weiss G | Wells' syndrome: report of a case and review of the literature | | | | 2001 | | pubmed | title/abstract | irrelevant |
| 299 | Shen WC | Wells syndrome (eosinophilic cellulitis) caused by adrenal insufficiency and relieved by cortisone supplementation | | | | 2018 | | pubmed | title/abstract | irrelevant |
| 300 | Peckruhn M | Life of lesions in eosinophilic cellulitis (Wells' syndrome)-a condition that may be missed at first sight | | | | 2015 | | pubmed | title/abstract | irrelevant |
| 301 | Rutter MM | Idiopathic hypercalcemia and eosinophilic fasciitis: a novel association | | | | 2004 | | pubmed | title/abstract | irrelevant |
| 302 | Ekiz Ö | Necrotizing fasciitis in a patient with bullous pemphigoid treating with systemic steroid | | | | 2013 | | pubmed | title/abstract | irrelevant |
| 303 | Lattmann J | [Eosinophilic fasciitis (Shulman's syndrome)] | | | | 1990 | | pubmed | title/abstract | irrelevant |
| 304 | Khanna D | Infliximab may be effective in the treatment of steroid-resistant eosinophilic fasciitis: report of three cases | | | | 2010 | | pubmed | title/abstract | irrelevant |
| 305 | Moreno M | Wells' syndrome related to tetanus vaccine | | | | 1997 | | pubmed | title/abstract | irrelevant |
| 306 | Heckmann JG | Multiple brain abscesses caused by Fusobacterium nucleatum treated conservatively | | | | 2003 | | pubmed | title/abstract | irrelevant |
| 307 | BODEY GP | USE OF GAMMA GLOBULIN INFECTION IN ACUTE-LEUKEMIA PATIENTS | | | | 1964 | | pubmed | title/abstract | irrelevant |
| 308 | Spinnato S | Nonoperative treatment of cerebellar abscesses. A case report and review of the literature | | | | 1998 | | pubmed | title/abstract | irrelevant |
| 309 | Graham BS | Nodular fasciitis: response to intralesional corticosteroids | | | | 1999 | | pubmed | title/abstract | irrelevant |
| 310 | McGarry JG | Accurate intra-articular knee joint injection in the obese? 'Fat Chance!'--A clinical lesson and recommendations for secondary referral | | | | 2011 | | pubmed | title/abstract | irrelevant |
| 311 | Singanayagam A | Systemic cytokine storm in severe eosinophilic dermatitis | | | | 2015 | | pubmed | title/abstract | irrelevant |
| 312 | Miret C | IgA nephropathy associated with eosinophilic fasciitis: report of a case | | | | 2003 | | pubmed | title/abstract | irrelevant |
| 313 | Engelman CJ | Orbital abscess following subtenon triamcinolone injection | | | | 2004 | | pubmed | title/abstract | case report |
| 314 | Bell H | Cutaneous reactive angiomatosis mimicking cellulitis in a patient with a renal transplant | | | | 2021 | | pubmed | title/abstract | irrelevant |
| 315 | O'Laughlin TJ | Rehabilitation of eosinophilic fasciitis. A case report | | | | 1994 | | pubmed | title/abstract | irrelevant |
| 316 | Sirbaugh PE | A case of orbital pseudotumor masquerading as orbital cellulitis in a patient with proptosis and fever | | | | 1997 | | pubmed | duplicate | duplicate |
| 317 | Cummings KC 3rd | Case report: epidural abscess in a parturient with pruritic urticarial papules and plaques of pregnancy (PUPPP) | | | | 2006 | | pubmed | title/abstract | irrelevant |
| 318 | Yildirim N | Otologic and leptomeningeal involvements as presenting features in seronegative Wegener granulomatosis | | | | 2008 | | pubmed | title/abstract | irrelevant |
| 319 | Farrell AM | Eosinophilic fasciitis associated with autoimmune thyroid disease and myelodysplasia treated with pulsed methylprednisolone and antihistamines | | | | 1999 | | pubmed | title/abstract | irrelevant |
| 320 | Marty R | Effects of corticosteroid (dexamethasone) administration on the brain scan | | | | 1973 | | pubmed | title/abstract | case report |
| 321 | Dziadzio L | Cytokine abnormalities in a patient with eosinophilic fasciitis | | | | 2003 | | pubmed | title/abstract | irrelevant |
| 322 | Sugiura K | Cryptococcal cellulitis in a patient with bullous pemphigoid | | | | 2013 | | pubmed | title/abstract | irrelevant |
| 323 | Meyer DR | Allergic fungal sinusitis with subperiosteal orbital abscess | | | | 2005 | | pubmed | title/abstract | irrelevant |
| 324 | Liu ZH | Successful treatment of severe kerion Celsi in an immunocompromised girl with evacuation of pus, terbinafine and short course glucocorticosteroids | | | | 2016 | | pubmed | title/abstract | irrelevant |
| 325 | Antic M | Eosinophilic fasciitis 30 years after - what do we really know? Report of 11 patients and review of the literature | | | | 2006 | | pubmed | title/abstract | irrelevant |
| 326 | Fujikawa K | Protracted febrile myalgia syndrome in a Japanese patient with fasciitis detected on MRI | | | | 2014 | | pubmed | title/abstract | irrelevant |
| 327 | Daniel RS | Case report of unilateral eosinophilic fasciitis in a Vietnamese woman | | | | 2009 | | pubmed | title/abstract | irrelevant |
| 328 | Ladoyanni E | A patient with Wells' syndrome | | | | 2010 | | pubmed | title/abstract | irrelevant |
| 329 | Moon HS | Eosinophilic cellulitis in an infant | | | | 2010 | | pubmed | title/abstract | irrelevant |
| 330 | Pagalavan L | Cerebral toxoplasmosis in systemic lupus erythematosus following intravenous methylprednisolone | | | | 2011 | | pubmed | title/abstract | irrelevant |
| 331 | Taylor WC | Eosinophilic fasciitis in a duathlete | | | | 2009 | | pubmed | title/abstract | irrelevant |
| 332 | Bach M | [Neurological symptoms in a patient on anti-TNF therapy, methotrexate and prednisolone for rheumatoid arthritis] | | | | 2020 | | pubmed | title/abstract | irrelevant |
| 333 | Freund B | Ludwig's angina: a place for steroid therapy in its management? | | | | 1992 | | pubmed | title/abstract | case report |
| 334 | Silverman ED | Eosinophilic fasciitis in a two-year-old child | | | | 1985 | | pubmed | title/abstract | irrelevant |
| 335 | Knutti O | Postoperative highcervical quadriplegia after transoral biopsy of a paravertebral tuberculous abscess with epidural extension: complete resolution after decompressive laminectomy and high-dose methylprednisolone | | | | 1997 | | pubmed | title/abstract | irrelevant |
| 336 | Sukhija J | Acute orbital abscess complicating deep posterior subtenon triamcinolone injection | | | | 2008 | | pubmed | title/abstract | case report |
| 337 | Pollard ZF | Acute rectus muscle palsy in children as a result of orbital myositis | | | | 1996 | | pubmed | title/abstract | irrelevant |
| 338 | Miura T | Subcutaneous Sweet syndrome mimicking cellulitis in a patient with myelodysplastic syndrome and subsequent secondary pulmonary alveolar proteinosis | | | | 2020 | | pubmed | title/abstract | irrelevant |
| 339 | Solomon G | Eosinophilic fasciitis responsive to cimetidine | | | | 1982 | | pubmed | title/abstract | irrelevant |
| 340 | Graham RO | Intravitreal injection of dexamethasone. Treatment of experimentally induced endophthalmitis | | | | 1974 | | pubmed | title/abstract | Animal study |
| 341 | Matsuno O | Pyomyositis associated with Bacteroides fragilis in a patient with multiple myeloma | | | | 1998 | | pubmed | title/abstract | irrelevant |
| 342 | De Jonge-Bok JM | Diffuse (eosinophilic) fasciitis. A series of six cases | | | | 1984 | | pubmed | title/abstract | irrelevant |
| 343 | Manousaridis I | Dexamethasone cyclophosphamide pulse therapy is highly efficient in autoimmune blistering and connective tissue disorders | | | | 2011 | | pubmed | title/abstract | irrelevant |
| 344 | Belzunegui J | Primary tuberculous muscle abscess in a patient with systemic lupus erythematosus | | | | 1995 | | pubmed | title/abstract | irrelevant |
| 345 | Kaklamanis P | Eosinophilic fasciitis with thrombocytopenia and Dupuytren's contracture | | | | 1990 | | pubmed | title/abstract | irrelevant |
| 346 | Godeiro-Junior C | An amyotrophic lateral sclerosis mimicker: eosinophilic fasciitis | | | | 2008 | | pubmed | title/abstract | irrelevant |
| 347 | Inaoki M | Adult-onset Stills disease with a cellulitis-like eruption | | | | 2009 | | pubmed | title/abstract | irrelevant |
| 348 | Mashima E | Eosinophilic Cellulitis Possibly Due to Mosquito Bite With High IL-5 Production | | | | 2017 | | pubmed | title/abstract | irrelevant |
| 349 | Kaul S | Spinal extradural abscess following local steroid injection | | | | 2000 | | pubmed | title/abstract | irrelevant |
| 350 | Kuba S | Vacuum-assisted biopsy and steroid therapy for granulomatous lobular mastitis: report of three cases | | | | 2009 | | pubmed | title/abstract | irrelevant |
| 351 | Loeliger AE | Eosinophilic fasciitis presenting with a reactive hepatitis | | | | 1991 | | pubmed | title/abstract | irrelevant |
| 352 | Bertken R | Chronic progressive eosinophilic fasciitis: report of a 20-year failure to attain remission | | | | 1983 | | pubmed | title/abstract | irrelevant |
| 353 | Lorente-Lavirgen AI | Pruritic nodules and plaques on the arms with blisters in a patient with chronic lymphocytic leukemia | | | | 2014 | | pubmed | title/abstract | irrelevant |
| 354 | Islam MN | Eosinophilic fasciitis: what matters in management in a developing country--a case report with two and a half-year follow-up | | | | 2012 | | pubmed | title/abstract | irrelevant |
| 355 | Kadoba K | Large vessel giant cell arteritis suggested by magnetic resonance imaging of the thigh: a potential mimicker of myositis, fasciitis and skeletal muscle vasculitis | | | | 2019 | | pubmed | title/abstract | irrelevant |
| 356 | Gaeta M | MRI findings of neutrophilic fasciitis in a patient with acute febrile neutrophilic dermatosis (Sweet's syndrome) | | | | 2011 | | pubmed | title/abstract | irrelevant |
| 357 | Wong SM | Ultrasound guided injection of plantar fasciitis | | | | 2001 | | pubmed | title/abstract | irrelevant |
| 358 | Akita S | Necrotizing fasciitis after underlying illness and steroid intake | | | | 2000 | | pubmed | title/abstract | irrelevant |
| 359 | Ngan Kee WD | Steroid therapy and extradural analgesia | | | | 1992 | | pubmed | title/abstract | irrelevant |
| 360 | Manuchehri K | A case of orbital myositis secondary to orbital cellulitis in a child | | | | 2003 | | pubmed | title/abstract | case report |
| 361 | Bizzarri F | [The observations in a case of eosinophilic fasciitis] | | | | 1992 | | pubmed | title/abstract | irrelevant |
| 362 | Hayashi Y | Cellulitis with Pseudomonas putida bacteremia in a patient with systemic lupus erythematosus: A case report | | | | 2020 | | pubmed | title/abstract | irrelevant |
| 363 | Chatterjee S | Diffuse skin thickening, myalgias and joint stiffness in a 41-year-old man | | | | 2018 | | pubmed | title/abstract | irrelevant |
| 364 | Bhushan M | Abdominal wall thickening in a middle aged man | | | | 2000 | | pubmed | title/abstract | irrelevant |
| 365 | Papadakis CE | Cervical prevertebral abscess owing to injection of corticosteroids | | | | 2005 | | pubmed | title/abstract | irrelevant |
| 366 | Zuk J | Disseminated nocardiosis mimicking exacerbation of pulmonary sarcoidosis | | | | 2013 | | pubmed | title/abstract | irrelevant |
| 367 | Green WH | Recurrent, pruritic dermal plaques and bullae. Diagnosis: eosinophilic cellulitis (Wells syndrome) | | | | 2007 | | pubmed | title/abstract | irrelevant |
| 368 | Hashimoto N | Fulminant necrotising fasciitis developing during long term corticosteroid treatment of systemic lupus erythematosus | | | | 2002 | | pubmed | title/abstract | irrelevant |
| 369 | Chu GY | Eosinophilic fasciitis following allogeneic bone marrow transplantation in a patient with acute myeloid leukaemia | | | | 2014 | | pubmed | title/abstract | irrelevant |
| 370 | Mechow N | Gigantic erythematous plaques with violaceous oedematous borders: a quiz. Wells' syndrome | | | | 2013 | | pubmed | title/abstract | irrelevant |
| 371 | Mondal S | Ultrasound is a useful adjunct in diagnosis of eosinophilic fasciitis | | | | 2015 | | pubmed | title/abstract | irrelevant |
| 372 | Janssen LJF | Image Gallery: A rare abscess-like presentation of Langerhans cell histiocytosis | | | | 2017 | | pubmed | title/abstract | irrelevant |
| 373 | Garofalo C | Very large abscesses of lower limbs by Nocardia farcinica requiring surgical management in patient with minimal change disease under chronic steroid treatment | | | | 2021 | | pubmed | title/abstract | irrelevant |
| 374 | Sood S | Chryseobacterium meningosepticum cellulitis and sepsis in an adult female with Pemphigus vulgaris | | | | 2010 | | pubmed | title/abstract | irrelevant |
| 375 | Bryant K | Hepatosplenic cat scratch disease treated with corticosteroids | | | | 2003 | | pubmed | title/abstract | irrelevant |
| 376 | Eggelmeijer F | [A fascinating disease?] | | | | 1989 | | pubmed | title/abstract | irrelevant |
| 377 | Tsang KW | Rhodococcus equi lung abscess complicating Evan's syndrome treated with corticosteroid | | | | 1998 | | pubmed | title/abstract | irrelevant |
| 378 | Harar RP | Descending necrotizing mediastinitis: report of a case following steroid neck injection | | | | 2002 | | pubmed | title/abstract | irrelevant |
| 379 | Kieran SM | Mycotic peripheral aneurysms and intracerebral abscesses secondary to infective endocarditis | | | | 2004 | | pubmed | title/abstract | irrelevant |
| 380 | Zhu L | Diffuse polymorphic eosinophilic cellulitis in a patient with metallic alloy implants: a possible association? | | | | 2011 | | pubmed | title/abstract | irrelevant |
| 381 | Izumi K | [Diffuse fasciitis with eosinophilia] | | | | 2001 | | pubmed | title/abstract | irrelevant |
| 382 | Azarbod P | Localised abscess following an injection of subtenon triamcinolone acitonide | | | | 2007 | | pubmed | title/abstract | case report |
| 383 | Venegas-Montoya E | Use of corticosteroids as an alternative to surgical treatment for liver abscesses in chronic granulomatous disease | | | | 2016 | | pubmed | title/abstract | irrelevant |
| 384 | Kusunoki T | A case of calcific retropharyngeal tendinitis suspected to be a retropharyngeal abscess upon the first medical examination | | | | 2006 | | pubmed | title/abstract | irrelevant |
| 385 | Hara M | [Shulman syndrome, diffuse eosinophilic fasciitis] | | | | 2000 | | pubmed | title/abstract | irrelevant |
| 386 | Kassiri K | Parainfectious optic neuropathy in a young patient with group A Streptococcus pyogenes orbital cellulitis | | | | 2010 | | pubmed | duplicate | duplicate |
| 387 | Yoshida Y | Dermatomyositis with tuberculous fasciitis | | | | 2004 | | pubmed | title/abstract | irrelevant |
| 388 | Delibaş A | Necrotizing fasciitis in a child: a rare complication of idiopathic nephrotic syndrome | | | | 2005 | | pubmed | title/abstract | irrelevant |
| 389 | Rahimy E | Orbital inflammation after zoledronate infusion: an emerging complication | | | | 2013 | | pubmed | title/abstract | irrelevant |
| 390 | Tenstad HB | Herpes zoster infection-induced common peroneal nerve paresis resulting in foot drop in a patient with eosinophilic fasciitis: a case report | | | | 2020 | | pubmed | title/abstract | irrelevant |
| 391 | Yam A | Necrotising fasciitis after corticosteroid injection for trigger finger: a severe complication from a 'safe' procedure | | | | 2009 | | pubmed | title/abstract | irrelevant |
| 392 | GOLINELLI G | [Treatment of some lung diseases with sigmamycin and prednisolone] | | | | 1961 | | pubmed | title/abstract | irrelevant |
| 393 | Frikha F | [Fasciitis with hypereosinophilia (Shulman syndrome) in a 34 year old woman] | | | | 2013 | | pubmed | title/abstract | irrelevant |
| 394 | Patil B | Hyaluronidase allergy after peribulbar anesthesia with orbital inflammation | | | | 2005 | | pubmed | duplicate | duplicate |
| 395 | Leecy T | Neutrophilic dermatosis of the dorsal hands: an often under recognised and mistreated entity | | | | 2013 | | pubmed | title/abstract | irrelevant |
| 396 | Biasi D | [Scleroderma induced by chemical agents. Description of a case and review of the literature] | | | | 1995 | | pubmed | title/abstract | irrelevant |
| 397 | Richardson MW | Spindle cell lesions of the head and neck mimicking rhabdomyosarcoma in children | | | | 2002 | | pubmed | title/abstract | irrelevant |
| 398 | Kloehn S | [Cushing syndrome with life-threatening infectious complications] | | | | 1997 | | pubmed | title/abstract | irrelevant |
| 399 | Paudyal BP | Eosinophilic fascitis: a rare fibrosing disorder | | | | 2012 | | pubmed | title/abstract | irrelevant |
| 400 | FRICKE E | [JOINT SUPPURATION FOLLOWING CORTISONE THERAPY] | | | | 1964 | | pubmed | title/abstract | irrelevant |
| 401 | Quhill F | Hyaluronidase allergy after peribulbar anesthesia with orbital inflammation | | | | 2004 | | pubmed | title/abstract | irrelevant |
| 402 | Markusse HM | Rheumatoid arthritis with eosinophilic fasciitis and pure red cell aplasia | | | | 1989 | | pubmed | title/abstract | irrelevant |
| 403 | Wang IJ | Juvenile dermatomyositis complicated with vasculitis and duodenal perforation | | | | 2001 | | pubmed | title/abstract | irrelevant |
| 404 | Hines DA | Eosinophilic fasciitis: an underdiagnosed syndrome | | | | 1981 | | pubmed | title/abstract | irrelevant |
| 405 | Ching DW | Childhood eosinophilic fasciitis presenting as inflammatory polyarthritis and associated with selective IgA deficiency | | | | 1991 | | pubmed | title/abstract | irrelevant |
| 406 | Vohra P | Disseminated nocardiosis complicating medical therapy in Crohn's disease | | | | 1997 | | pubmed | title/abstract | irrelevant |
| 407 | Scharfen J Jr | [Nocardia farcinica as the causative agent of a brain abscess in a patient with interstitial lung disease] | | | | 2010 | | pubmed | title/abstract | irrelevant |
| 408 | Sills EM | Diffuse fasciitis with eosinophilia in childhood | | | | 1982 | | pubmed | title/abstract | irrelevant |
| 409 | Pompecki R | [Fasciitis with eosinophilia and hypergammaglobulinaemia (author's transl)] | | | | 1982 | | pubmed | title/abstract | irrelevant |
| 410 | Michet CJ Jr | Eosinophilic fasciitis: report of 15 cases | | | | 1981 | | pubmed | title/abstract | irrelevant |
| 411 | Yamanishi Y | Complete remission of relapsing eosinophilic fasciitis associated with bronchial asthma following regular steroid inhalation | | | | 2000 | | pubmed | title/abstract | irrelevant |
| 412 | Takeuchi A | Bilateral orbital inflammation following intravesical bacille Calmette-Guérin immunotherapy for bladder cancer | | | | 2012 | | pubmed | duplicate | duplicate |
| 413 | Debrunner J | Falsely positive dexamethasone suppression test in a patient treated with phenytoin to prevent seizures due to nocardia brain abscesses | | | | 2002 | | pubmed | title/abstract | irrelevant |
| 414 | Pearl RM | Complications following silicone injections for augmentation of the contours of the face | | | | 1978 | | pubmed | title/abstract | irrelevant |
| 415 | Martin XD | Ocular complications of the Fernand-Widal triad and its therapy | | | | 2003 | | pubmed | duplicate | duplicate |
| 416 | Grigoris I | Eosinophilic fasciitis associated with L-tryptophan ingestion | | | | 1992 | | pubmed | title/abstract | irrelevant |
| 417 | Lafleur L | Cryptococcal cellulitis in a patient on prednisone monotherapy for myasthenia gravis | | | | 2004 | | pubmed | title/abstract | irrelevant |
| 418 | Matz K | [Effects of nebacetin and nebacetin dexamethasone on the growth of microorganisms in the root canal of the tooth (author's transl)] | | | | 1974 | | pubmed | title/abstract | irrelevant |
| 419 | Zimmerli PB | [Multilocular painful urticarial plaques. Eosinophilic cellulitis (Wells syndrome)] | | | | 2004 | | pubmed | title/abstract | irrelevant |
| 420 | Plastiras SC | Eosinophilic fasciitis in a patient with psoriasis: an unusual association | | | | 2006 | | pubmed | title/abstract | irrelevant |
| 421 | Hassan B | [A diagnostic trap in urologic emergencies] | | | | 2014 | | pubmed | title/abstract | irrelevant |
| 422 | Mrabet D | A case of Shulman disease in a patient with systemic lupus erythematosus | | | | 2010 | | pubmed | title/abstract | irrelevant |
| 423 | Hokken JW | [Eosinophilic fasciitis; the importance of early detection for optimal outcomes] | | | | 2008 | | pubmed | title/abstract | irrelevant |
| 424 | Lee MW | Eosinophilic cellulitis case report: treatment options | | | | 1994 | | pubmed | title/abstract | irrelevant |
| 425 | Kapsalakis Z | Analysis of the treatment of 12 consecutive cases of brain abscess | | | | 1972 | | pubmed | title/abstract | irrelevant |
| 426 | Keret S | [LIVER ABSCESSES IN A PATIENT WITH BEHCET'S DISEASE] | | | | 2025 | | pubmed | title/abstract | irrelevant |
| 427 | Ozden MG | Is it really possible to differentiate insect bite-like reaction and nodular variant of eosinophilic cellulitis in a healthy person? | | | | 2009 | | pubmed | title/abstract | irrelevant |
| 428 | Desvignes-Engelbert A | Polymyalgia revealing eosinophilic fasciitis in a young male: Contribution of magnetic resonance imaging | | | | 2010 | | pubmed | title/abstract | irrelevant |
| 429 | Agrawal A | Ulnar nerve abscess and relapse in a patient with indeterminate leprosy 1 year after completion of multidrug therapy | | | | 2005 | | pubmed | title/abstract | irrelevant |
| 430 | Davenport TE | Subcutaneous abscess in a patient referred to physical therapy following spinal epidural injection for lumbar radiculopathy | | | | 2008 | | pubmed | title/abstract | irrelevant |
| 431 | Beaucher WN | Fasciitis with eosinophilia: a case report | | | | 1985 | | pubmed | title/abstract | irrelevant |
| 432 | Farrington ML | Eosinophilic fasciitis in children frequently progresses to scleroderma-like cutaneous fibrosis | | | | 1993 | | pubmed | title/abstract | irrelevant |
| 433 | Herson S | [Long-term development of eosinophilic fasciitis. Study of 11 cases] | | | | 1984 | | pubmed | title/abstract | irrelevant |
| 434 | Niskanen L | Compartment syndrome as a late complication of eosinophilic fasciitis | | | | 1989 | | pubmed | title/abstract | irrelevant |
| 435 | Fujimoto M | Serum aldolase level is a useful indicator of disease activity in eosinophilic fasciitis | | | | 1995 | | pubmed | title/abstract | irrelevant |
| 436 | Hintner H | [Fasciitis with eosinophilia - Shulman syndrome] | | | | 1981 | | pubmed | title/abstract | irrelevant |
| 437 | Dunant Y | [Corticosteroids and orodental lesions] | | | | 1984 | | pubmed | title/abstract | unclear |
| 438 | Miehle W | [Atypical course in (eosinophilic) fasciitis] | | | | 1995 | | pubmed | title/abstract | irrelevant |
| 439 | Long WD | Experimental method for producing brain abscesses in dogs with evaluation of the effect of dexamethasone and antibiotic therapy on the pathogenesis of intracerebral abscesses | | | | 1968 | | pubmed | title/abstract | irrelevant |
| 440 | Baack BR | Atypical mycobacterium soft-tissue infection of the dorsal radial wrist: a possible complication of steroid injection for de Quervain's disease | | | | 1991 | | pubmed | title/abstract | irrelevant |
| 441 | Williams HJ | Childhood eosinophilic fasciitis--progression to linear scleroderma | | | | 1986 | | pubmed | title/abstract | irrelevant |
| 442 | Sabbagh M | Association of Shulman's syndrome and morphea: a case report | | | | 2003 | | pubmed | title/abstract | irrelevant |
| 443 | Gavriliţă L | Diffuse fasciitis with eosinophilia | | | | 1986 | | pubmed | title/abstract | irrelevant |
| 444 | Daxecker M | [Therapy-resistant tuberculous skin abscesses after methylprednisolone therapy] | | | | 2008 | | pubmed | title/abstract | irrelevant |
| 445 | Smith RL | Indurated plaques on the arms | | | | 2002 | | pubmed | title/abstract | irrelevant |
| 446 | Wong SM | Re: The role of ultrasonography in the diagnosis and management of idiopathic plantar fasciitis | | | | 2002 | | pubmed | duplicate | duplicate |
| 447 | Muschter K | [Hazards of local corticoid therapy] | | | | 1973 | | pubmed | title/abstract | irrelevant |
| 448 | Kane D | Re: The role of ultrasonography in the diagnosis and management of idiopathic plantar fasciitis | | | | 2003 | | pubmed | title/abstract | irrelevant |
| 449 | Nakagawa Y | [A case of eosinophilic fasciitis with excessive increase of muscle enzyme] | | | | 1996 | | pubmed | title/abstract | irrelevant |
| 450 | BIAGI F | INFLUENCE OF SOME STEROIDS IN THE EXPERIMENTAL PRODUCTION OF AMEBIC HEPATIC ABSCESS | | | | 1963 | | pubmed | title/abstract | irrelevant |
| 451 | de CAMP | [Experiences with 6-methylprednisolone (especially its acetate form) in a lung clinic] | | | | 1962 | | pubmed | title/abstract | irrelevant |
| 452 | Dojcinovic I | Mucormycoses: serious complication of high-dose corticosteroid therapy for traumatic optic neuropathy | | | | 2008 | | pubmed | title/abstract | case report |
| 453 | Balat A | Eosinophilic fasciitis--progression to linear scleroderma: a case report | | | | 1999 | | pubmed | title/abstract | irrelevant |
| 454 | Senff H | [L-tryptophan-induced eosinophilia-myalgia syndrome with features of diffuse fasciitis with eosinophilia] | | | | 1990 | | pubmed | title/abstract | irrelevant |
| 455 | Möller E | [Side-effects of glucocorticoids in liver therapy] | | | | 1969 | | pubmed | title/abstract | irrelevant |
| 456 | Puszczewicz M | [Diagnostic problems in eosinophilic fasciitis] | | | | 2006 | | pubmed | title/abstract | irrelevant |
| 457 | Hatton MP | Exaggerated postsurgical inflammation in a patient with insufficiently treated Addison disease | | | | 2009 | | pubmed | duplicate | duplicate |
| 458 | Fukuda H | [A case of diffuse fasciitis and its MRI findings] | | | | 1996 | | pubmed | duplicate | duplicate |
| 459 | de Winter S | [Eosinophilic cellulitis (Wells syndrome)] | | | | 2001 | | pubmed | title/abstract | irrelevant |
| 460 | Karam A | Aseptic neutrophilic abscess of the vulva | | | | 2006 | | pubmed | title/abstract | irrelevant |
| 461 | Newsome CK | Peritonsillar cellulitis. Report of a case | | | | 1967 | | pubmed | title/abstract | irrelevant |
| 462 | Govoni M | Churg-Strauss syndrome and Wells syndrome: coincidence or pathogenetic association? A new case report | | | | 2007 | | pubmed | title/abstract | irrelevant |
| 463 | Boiesen M | Eosinophilic fasciitis. Report of a case with features of other autoimmune disease | | | | 1983 | | pubmed | title/abstract | irrelevant |
| 464 | Wakhlu A | Nocardiosis in patients of chronic idiopathic thrombocytopenic purpura on steroids | | | | 2004 | | pubmed | title/abstract | irrelevant |
| 465 | Zielinski CC | [Fasciitis with eosinophilia--a case report] | | | | 1982 | | pubmed | title/abstract | irrelevant |
| 466 | Ogden IW | Fascial space infection in a patient with pemphigus | | | | 1967 | | pubmed | title/abstract | case report |
| 467 | Viraben R | Eosinophilic fasciitis (Shulman syndrome) in association with morphea, immunological disturbance and profuse achromia | | | | 1987 | | pubmed | title/abstract | irrelevant |
| 468 | Sanchez Roman J | [Eosinophilic fasciitis. Analysis of 3 new cases with atypical characteristics] | | | | 1983 | | pubmed | title/abstract | irrelevant |
| 469 | Guy C | [Fulminating necrotizing fasciitis after intramuscular injection of a corticoid. Contributing role of non-steroidal anti-inflammatory agents] | | | | 1993 | | pubmed | title/abstract | irrelevant |
| 470 | Alvi NP | Ophthalmic artery occlusion following orbital inflammation: a clinical and histopathological study | | | | 1998 | | pubmed | title/abstract | irrelevant |
| 471 | Koppenwallner C | [Fasciitis and eosinophilia--a scleroderma-like disease] | | | | 1982 | | pubmed | title/abstract | irrelevant |
| 472 | Ferlazzo B | Eosinophilic fasciitis: report of a case diagnosed 14 years after its onset | | | | 1995 | | pubmed | title/abstract | irrelevant |
| 473 | Bani-Sadr F | [Value of immunoglobulins in Schulman fasciitis] | | | | 2000 | | pubmed | title/abstract | irrelevant |
| 474 | Berger U | [Injection abscess due to Neisseria perflava] | | | | 1973 | | pubmed | title/abstract | irrelevant |
| 475 | Kikuchi K | Vitamin D-dependent rickets type II: report of three cases | | | | 1988 | | pubmed | title/abstract | irrelevant |
| 476 | Douglas SE | New lease of life. 2. Cellulitis cured by Fucidin | | | | 1969 | | pubmed | title/abstract | irrelevant |
| 477 | Aelion JA | Eosinophilic fasciitis (Shulman syndrome) with prominent synovitis | | | | 1982 | | pubmed | title/abstract | irrelevant |
| 478 | Asgeirsson H | [Nocardiosis in immunocompromised host presenting as cellulitis] | | | | 2010 | | pubmed | title/abstract | irrelevant |
| 479 | Lanzavecchia C | [Influence of corticoid preparations on dental structure] | | | | 1967 | | pubmed | title/abstract | unclear |
| 480 | Bradley JS | Dexamethasone therapy in meningitis: potentially misleading antiinflammatory effects in central nervous system infections | | | | 1994 | | pubmed | title/abstract | irrelevant |
| 481 | Haferkamp G | [Modern pathophysiological aspects regarding the treatment of brain edema (author's transl)] | | | | 1978 | | pubmed | title/abstract | irrelevant |
| 482 | Yulis Modak J | [Treatment of periapical osteitis with a new multi-drug paste] | | | | 1972 | | pubmed | title/abstract | unclear |
| 483 | Iwata A | [A case of diffuse fasciitis and its MRI findings] | | | | 1997 | | pubmed | title/abstract | irrelevant |
| 484 | Gerl A | [Indurating soft tissue swelling, eosinophilia and increased blood sedimentation rate] | | | | 1993 | | pubmed | title/abstract | irrelevant |
| 485 | Zelenin RP | [Complications of local use of hydrocortisone and their prevention] | | | | 1977 | | pubmed | title/abstract | irrelevant |
| 486 | Doménech Juan I | [Retropharyngeal phlegmon in the adult. Presentation of a case] | | | | 2003 | | pubmed | title/abstract | irrelevant |
| 487 | Shibuya E | [Effects of several steroids on an aseptic abscess made in the gastric submucosal layer in rats--histological findings] | | | | 1967 | | pubmed | title/abstract | irrelevant |
| 488 | Keitel W | [77-year-old otherwise healthy colleague, suffers from eosinophilic fasciitis (Shulmann syndrome)] | | | | 1993 | | pubmed | title/abstract | irrelevant |
| 489 | VAN CAUWENBERGE H | [Local inflammatory reactions & blood corticosteroids in rat] | | | | 1958 | | pubmed | title/abstract | irrelevant |
| 490 | Tomosugi N | [Eosinophilic fasciitis: case reports and review] | | | | 1984 | | pubmed | title/abstract | irrelevant |
| 491 | Mensing H | [Diffuse fasciitis with eosinophilia (Shulman-syndrome) (author's transl)] | | | | 1982 | | pubmed | title/abstract | irrelevant |
| 492 | Jiménez Martínez A | [Eosinophilic fasciitis. Response to treatment with cimetidine] | | | | 1987 | | pubmed | title/abstract | irrelevant |
| 493 | de CAMP G | [Critical observations on the use of corticosteroids as a differential diagnostic aid in unclear lung diseases. With special reference to miliary changes] | | | | 1962 | | pubmed | title/abstract | irrelevant |
| 494 | de CAMP G | [Corticosteroids as differential diagnostic aids in atypical diseases of the lungs] | | | | 1961 | | pubmed | title/abstract | irrelevant |
| 495 | Quilichini R | [A new case of eosinophilic fasciitis with bone marrow aplasia. Cure by high doses of corticoids] | | | | 1985 | | pubmed | title/abstract | irrelevant |
| 496 | Ambanelli U | [Eosinophilic fasciitis. Description of a clinical case with Raynaud's phenomenon] | | | | 1981 | | pubmed | title/abstract | irrelevant |
| 497 | Rietzschel I | [Eosinophilic fasciitis (Shulman syndrome)--a contribution to the differential diagnosis of sclerodermiform diseases] | | | | 1988 | | pubmed | title/abstract | irrelevant |
| 498 | Vereshchagin FF | [A case of severe suppurative complication after hydrocortisone administration] | | | | 1989 | | pubmed | title/abstract | irrelevant |
| 499 | Skov BG | [Eosinophilic fasciitis. A review and a characteristic case report] | | | | 1985 | | pubmed | title/abstract | irrelevant |
| 500 | Souaga K | [A plea for the rational use of anti-inflammatory agents in odontostomatology] | | | | 1998 | | pubmed | title/abstract | irrelevant |
| 501 | Olivos A | Entamoeba histolytica: mechanism of decrease of virulence of axenic cultures maintained for prolonged periods | | | | 2005 | | pubmed | title/abstract | irrelevant |
| 502 | Ikewaki J | Peribulbar fungal abscess and endophthalmitis following posterior subtenon injection of triamcinolone acetonide | | | | 2009 | | pubmed | title/abstract | irrelevant |
| 503 | Roman S | [Palmar fasciitis and paraneoplastic polyarthritis associated with hepatocellular carcinoma] | | | | 2001 | | pubmed | title/abstract | irrelevant |
| 504 | Willemse PH | Palmar fasciitis and arthritis in a patient with an extraovarian adenocarcinoma of the coelomic epithelium | | | | 1991 | | pubmed | title/abstract | irrelevant |
| 505 | Coldiron BM | Low-dose alternate-day prednisone for persistent Wells' syndrome | | | | 1989 | | pubmed | title/abstract | irrelevant |
| 506 | Zloczower M | [Eosinophilic cellulitis (Wells syndrome) with involvement of para-articular muscles and fascia] | | | | 1994 | | pubmed | title/abstract | irrelevant |
| 507 | Cannavò SP | Morphea-like plaque in childhood | | | | 2003 | | pubmed | title/abstract | irrelevant |
| 508 | Oldfather T | Grand rounds: Intraperitoneal infection and emergency operation in patients on long-term corticosteroid therapy | | | | 1974 | | pubmed | title/abstract | irrelevant |
| 509 | Ball EM | Steroid injection for inferior heel pain: a randomised controlled trial | | | | 2013 | | pubmed | duplicate | duplicate |
| 510 | Çatal B | Is there a relation between plantar fasciitis and total cholesterol levels? | | | | 2022 | | pubmed | title/abstract | irrelevant |
| 511 | Littlejohn GO | Eosinophilic fasciitis and aplastic anaemia | | | | 1980 | | pubmed | title/abstract | irrelevant |
| 512 | Akşahin E | The comparison of the effect of corticosteroids and platelet-rich plasma (PRP) for the treatment of plantar fasciitis | | | | 2012 | | pubmed | title/abstract | irrelevant |
| 513 | Dabrowski Z | [Gastric phlegmon during corticosteroid treatment for psoriasis] | | | | 1975 | | pubmed | title/abstract | irrelevant |
| 514 | Riumshin GI | [Complications of the local administration of hydrocortisone] | | | | 1974 | | pubmed | title/abstract | irrelevant |
| 515 | Mittal A | Psoas muscle cysticercosis presenting as acute appendicitis | | | | 2008 | | pubmed | title/abstract | irrelevant |
| 516 | Kane D | Ultrasound guided injection of recalcitrant plantar fasciitis | | | | 1998 | | pubmed | title/abstract | irrelevant |
| 517 | Suzuki G | Surgical management of eosinophilic fasciitis of the upper extremity | | | | 1997 | | pubmed | title/abstract | irrelevant |
| 518 | Hardman JC | Do corticosteroids improve outcomes in peritonsillar abscess? | | | | 2015 | | pubmed | title/abstract | Review |
| 519 | Segers AM | [Cellulite] | | | | 1985 | | pubmed | title/abstract | irrelevant |
| 520 | Chevalier K | [An abscessed granulomatous prostatitis] | | | | 2020 | | pubmed | title/abstract | irrelevant |
| 521 | Dhrami-Gavazi E | Bilateral Orbital Abscesses After Strabismus Surgery | | | | 2015 | | pubmed | duplicate | duplicate |
| 522 | Incalzi RA | Catastrophic antiphospholipid syndrome presenting with multiorgan failure and gangrenous lesions of the skin | | | | 2008 | | pubmed | title/abstract | irrelevant |
| 523 | Jacob SE | Paraneoplastic eosinophilic fasciitis: a case report | | | | 2003 | | pubmed | title/abstract | irrelevant |
| 524 | Moossavi M | Wells' syndrome: a clinical and histopathologic review of seven cases | | | | 2003 | | pubmed | title/abstract | irrelevant |
| 525 | Bardy A | Efficacy of Il-1β blockade in refractory aseptic abscesses syndrome | | | | 2014 | | pubmed | title/abstract | irrelevant |
| 526 | Chung CL | Wells syndrome: an enigmatic and therapeutically challenging disease | | | | 2006 | | pubmed | title/abstract | irrelevant |
| 527 | Glardon O | [Diagnosis and therapy of a dog with an atypical eosinophilic cellulitis] | | | | 2010 | | pubmed | title/abstract | irrelevant |
| 528 | Yoo SS | Disseminated cellulitic cryptococcosis in the setting of prednisone monotherapy for pemphigus vulgaris | | | | 2003 | | pubmed | title/abstract | irrelevant |
| 529 | Noh SH | Whole cerebrospinal axis infection after lumbar epidural injection: a case report | | | | 2015 | | pubmed | title/abstract | irrelevant |
| 530 | Khatri ML | Sweet's syndrome associated with myelodysplastic syndrome presenting as periorbital cellulitis | | | | 2007 | | pubmed | title/abstract | irrelevant |
| 531 | Frey T | Vitreous abscess following dart injury, masked by steroids and antibiotics | | | | 1968 | | pubmed | title/abstract | irrelevant |
| 532 | Cutts S | Plantar fasciitis | | | | 2012 | | pubmed | title/abstract | irrelevant |
| 533 | Caspi D | Multisystem presentation of eosinophilic fasciitis | | | | 1982 | | pubmed | title/abstract | irrelevant |
| 534 | Lee JY | Cranial fasciitis treated with intralesional corticosteroids | | | | 2004 | | pubmed | title/abstract | irrelevant |
| 535 | Flowers RS | Unexpected postoperative problems in skin grafting | | | | 1970 | | pubmed | title/abstract | irrelevant |
| 536 | Jin KW | Klebsiella Endophthalmitis as Retinal Vasculitis with Prostatic Abscess | | | | 2015 | | pubmed | title/abstract | irrelevant |
| 537 | Pillen S | Eosinophilic fasciitis in a child mimicking a myopathy | | | | 2006 | | pubmed | title/abstract | irrelevant |
| 538 | Monto RR | Platelet-rich plasma efficacy versus corticosteroid injection treatment for chronic severe plantar fasciitis | | | | 2014 | | pubmed | title/abstract | irrelevant |
| 539 | Jiménez-García N | Eosinophilic fasciitis in a pregnant woman with corticosteroid dependence and good response to infliximab | | | | 2021 | | pubmed | title/abstract | irrelevant |
| 540 | Mylona E | Post-radiation scleredema adultorum and diffuse eosinophilic fasciitis in the same patient | | | | 2011 | | pubmed | title/abstract | irrelevant |
| 541 | Kronschnabel EF | Orbital apex syndrome due to sinus infection | | | | 1974 | | pubmed | title/abstract | irrelevant |
| 542 | Gidumal R | Calcaneal osteomyelitis following steroid injection: a case report | | | | 1985 | | pubmed | title/abstract | irrelevant |
| 543 | Kuruvilla M | Treatment of hypereosinophilic syndrome and eosinophilic dermatitis with reslizumab | | | | 2018 | | pubmed | title/abstract | irrelevant |
| 544 | Mitrevski M | Sterile abscesses complicating monoclonal gammopathy of undetermined significance | | | | 2008 | | pubmed | title/abstract | irrelevant |
| 545 | Grandière Pérez L | Necrotizing cellulitis secondary to Aspergillus in a patient with metastatic breast cancer treated with systemic steroids and trastuzumab | | | | 2021 | | pubmed | title/abstract | irrelevant |
| 546 | Mahalingam S | The role of adjuvant systemic steroids in the management of periorbital cellulitis secondary to sinusitis: a systematic review and meta-analysis | | | | 2021 | | pubmed | duplicate | duplicate |
| 547 | Yucel I | Comparison of ultrasound-, palpation-, and scintigraphy-guided steroid injections in the treatment of plantar fasciitis | | | | 2009 | | pubmed | duplicate | duplicate |
| 548 | Pinal-Fernandez I | Groove sign in eosinophilic fasciitis | | | | 2014 | | pubmed | title/abstract | irrelevant |
| 549 | Mihori A | [A case of fasciitis associated with Basedow's disease and polymyositis] | | | | 1998 | | pubmed | title/abstract | irrelevant |
| 550 | Espersen GT | [NSAID (non-steroidal anti-inflammatory agent) and necrotizing fascitis] | | | | 1987 | | pubmed | title/abstract | irrelevant |
| 551 | Olczak-Kowalczyk D | The status of dental and jaw bones in children and adolescents after kidney and liver transplantation | | | | 2012 | | pubmed | title/abstract | irrelevant |
| 552 | McQuay HJ | Is steroid therapy a contraindication to extradural analgesia? | | | | 1992 | | pubmed | title/abstract | irrelevant |
| 553 | Joiner KA | The effect of corticosteroids on subcutaneous abscess formation in the mouse | | | | 1981 | | pubmed | title/abstract | irrelevant |
| 554 | Bachman KH | Recurrent cellulitis and bacteremia caused by Flavobacterium odoratum | | | | 1996 | | pubmed | title/abstract | irrelevant |
| 555 | Vasil'eva NN | [The pathological anatomy of pemphigus in connection with its treatment with steroid hormones] | | | | 1968 | | pubmed | title/abstract | irrelevant |
| 556 | De Nardo P | Left thigh phlegmon caused by Nocardia farcinica identified by 16S rRNA sequencing in a patient with leprosy: a case report | | | | 2013 | | pubmed | title/abstract | irrelevant |
| 557 | Tsai WC | Plantar fasciitis treated with local steroid injection: comparison between sonographic and palpation guidance | | | | 2006 | | pubmed | duplicate | duplicate |
| 558 | Serra Moltó A | External ophthalmomyiasis due to Dermatobia hominis. A case report | | | | 2018 | | pubmed | title/abstract | irrelevant |
| 559 | Parish LC | The enigma of acne therapy: the acne abscess | | | | 1967 | | pubmed | title/abstract | irrelevant |
| 560 | Neudert M | [Medical examination: Preparation for ENT specialisation: part 3] | | | | 2012 | | pubmed | title/abstract | irrelevant |
| 561 | Santos RP | Wells syndrome associated with lung cancer | | | | 2017 | | pubmed | title/abstract | irrelevant |
| 562 | Rodriguez-Lojo R | Recurrent episodes of periorbital edema in an elderly woman | | | | 2016 | | pubmed | title/abstract | irrelevant |
| 563 | Abe N | Disseminated Cryptococcosis with Bronchiolitis and Cellulitis | | | | 2019 | | pubmed | title/abstract | irrelevant |
| 564 | Hagiya H | Mycoplasma hominis periaortic abscess following heart-lung transplantation | | | | 2017 | | pubmed | title/abstract | irrelevant |
| 565 | Baird K | Imatinib mesylate for the treatment of steroid-refractory sclerotic-type cutaneous chronic graft-versus-host disease | | | | 2015 | | pubmed | title/abstract | irrelevant |
| 566 | Chaplain A | [Acute cervical necrotizing fasciitis of pharyngeal origin: possible role of steroidal and non-steroidal anti-inflammatory agents. Apropos of 5 cases] | | | | 1996 | | pubmed | title/abstract | irrelevant |
| 567 | Turner EJ | Long-term steroids and an extensive diverticular abscess | | | | 2012 | | pubmed | title/abstract | irrelevant |
| 568 | Chang KV | Ultrasonographic Imaging for the Diagnosis and Guided Injection of the Lateral Cord of the Plantar Fascia in a Jogger | | | | 2019 | | pubmed | title/abstract | irrelevant |
| 569 | Peplinski SL | The clinical reasoning process for the intervention of chronic plantar fasciitis | | | | 2010 | | pubmed | title/abstract | irrelevant |
| 570 | Love SM | More on danazol therapy for periareolar abscess | | | | 1986 | | pubmed | title/abstract | irrelevant |
| 571 | COLLINS CG | The use of cortisone in major gynecologic problems | | | | 1958 | | pubmed | title/abstract | irrelevant |
| 572 | Ambrocio DU | Eosinophilic fasciitis in a 57-year-old Japanese-American woman | | | | 2007 | | pubmed | title/abstract | irrelevant |
| 573 | Adil EA | Pediatric Subperiosteal Abscess Secondary to Acute Sinusitis: A Systematic Review and Meta-analysis | | | | 2020 | | pubmed | title/abstract | irrelevant |
| 574 | Pai R | Mycobacterium fortuitum skin infection as a complication of anabolic steroids: a rare case report | | | | 2013 | | pubmed | title/abstract | irrelevant |
| 575 | Wu TT | Imatinib as a potentially effective therapeutic alternative in corticosteroid-resistant eosinophilic fasciitis | | | | 2020 | | pubmed | title/abstract | irrelevant |
| 576 | Challa S | Relative sensitivity of Tc-99m WBC versus In-111 WBC in a patient with Crohn disease and steroid use | | | | 1997 | | pubmed | title/abstract | irrelevant |
| 577 | Yang WY | Platelet-rich plasma as a treatment for plantar fasciitis: A meta-analysis of randomized controlled trials | | | | 2017 | | pubmed | title/abstract | irrelevant |
| 578 | Lyons KP | Relative sensitivity of Tc-99m WBC versus In-111 WBC in a patient with Crohn's disease on steroids | | | | 1997 | | pubmed | title/abstract | irrelevant |
| 579 | Pino Rivero V | [Parotid phlegmon with involvement of both lobes. Report of a case] | | | | 2006 | | pubmed | title/abstract | irrelevant |
| 580 | Jennings HS 3rd | Acute cryptococcal cellulitis in renal transplant recipients | | | | 1981 | | pubmed | title/abstract | irrelevant |
| 581 | LALLEMAND M | [Importance of adrenal cortex hormones in the treatment of severe abscess of the lung] | | | | 1957 | | pubmed | title/abstract | irrelevant |
| 582 | Hutchison IL | New treatment for Ludwig's angina | | | | 1989 | | pubmed | fulltext | unclear |
| 583 | Kent S | The use of steroids in cervicofacial infections in UK OMFS departments: a Maxillofacial Surgery Trainees Research Collaborative (MTReC) study | | | | 2021 | | pubmed | fulltext | included |
| 584 | Ondzotto G | [Childhood laterocervical abscess fistulized in the pharynx: a case study] | | | | 2009 | | pubmed | title/abstract | irrelevant |
| 585 | Stavropoulos PG | Molluscum contagiosum and cryosurgery: triggering factors for Wells' syndrome? | | | | 2003 | | pubmed | title/abstract | irrelevant |
| 586 | Stevenson R | Cellulitis caused by Neisseria mucosa | | | | 1975 | | pubmed | title/abstract | irrelevant |
| 587 | Louie JS | Felty's syndrome | | | | 1971 | | pubmed | title/abstract | irrelevant |
| 588 | Burstin PP | Infectious mononucleosis and bilateral peritonsillar abscesses resulting in airway obstruction | | | | 1998 | | pubmed | title/abstract | irrelevant |
| 589 | Paver K | Complications from combined oral tetracycline and oral corticoid therapy in acne vulgaris | | | | 1970 | | pubmed | title/abstract | irrelevant |
| 590 | Campbell C | Two unusual uses of steroid hoones in pelvic infections | | | | 1969 | | pubmed | title/abstract | irrelevant |
| 591 | Maimon MS | Images in emergency medicine. Ludwig's angina in a 4 month old infant | | | | 2006 | | pubmed | title/abstract | irrelevant |
| 592 | Patel RM | An abscess causing a delayed optic neuropathy after decompression for thyroid eye disease | | | | 2014 | | pubmed | title/abstract | irrelevant |
| 593 | Misumi M | [Case of eosinophilic fasciitis with change of serum Th 1/Th 2 cytokine level which correlated with disease activity] | | | | 2002 | | pubmed | title/abstract | irrelevant |
| 594 | JOHNSON WS | LUDWIG'S ANGINA. CONCEPTS OF THERAPY, WITH REPORT OF A CASE | | | | 1963 | | pubmed | title/abstract | case report |
| 595 | Wronka KS | Calcaneal osteomyelitis following steroid injection for plantar fasciitis: a case report | | | | 2012 | | pubmed | title/abstract | irrelevant |
| 596 | MATTHEWS NM Jr | The use of cortisone in pelvic cellulitis | | | | 1953 | | pubmed | title/abstract | irrelevant |
| 597 | Grajower MM | Danazol therapy for periareolar abscess | | | | 1986 | | pubmed | title/abstract | irrelevant |
| 598 | BROSS W | [Application of ultracortenol in conservative therapy of chronic pulmonary abscess] | | | | 1958 | | pubmed | title/abstract | irrelevant |
| 599 | Yoo SS | Images in pathology: birefringent cryptococcus | | | | 2003 | | pubmed | title/abstract | irrelevant |
| 600 | COLLINS CG | Use of cortisone in pelvic cellulitis; preliminary report | | | | 1952 | | pubmed | title/abstract | irrelevant |
| 601 | Sanders MD | Acute presentation of thyroid ophthalmopathy | | | | 1986 | | pubmed | title/abstract | irrelevant |
| 602 | Leung KK | Red-eared zebra diagnosis: Case of relapsing polychondritis | | | | 2018 | | pubmed | title/abstract | irrelevant |
| 603 | Scheinfeld N | Dissecting cellulitis (Perifolliculitis Capitis Abscedens et Suffodiens): a comprehensive review focusing on new treatments and findings of the last decade with commentary comparing the therapies and causes of dissecting cellulitis to hidradenitis suppurativa | | | | 2014 | | pubmed | title/abstract | irrelevant |
| 604 | Hansen SW | Lung abscess in small cell carcinoma of the lung during chemotherapy and corticosteroids: an analysis of 276 consecutive patients | | | | 1986 | | pubmed | title/abstract | irrelevant |
| 605 | DINSDALE RC | Activation of dental infections by cortisone; studies in children with rheumatic fever | | | | 1958 | | pubmed | title/abstract | irrelevant |
| 606 | Kourtópoulos H | The influence of steroids on the penetration of antibiotics into brain tissue and brain abscesses. An experimental study in rats | | | | 1983 | | pubmed | title/abstract | irrelevant |
| 607 | Chen CM | Effectiveness of device-assisted ultrasound-guided steroid injection for treating plantar fasciitis | | | | 2013 | | pubmed | title/abstract | irrelevant |
| 608 | de Silva DJ | Masked orbital abscess in Wegener's granulomatosis | | | | 2007 | | pubmed | title/abstract | case series |
| 609 | Daif JL | Group a Streptococcus causing necrotizing fasciitis and toxic shock syndrome after medical termination of pregnancy | | | | 2009 | | pubmed | title/abstract | irrelevant |
| 610 | LaFave J | Upper Cervical Epidural Abscess Resulting in Respiratory Compromise After Lumbar Steroid Injection | | | | 2019 | | pubmed | title/abstract | irrelevant |
| 611 | Pollack MS | Graft stability in a heart transplant recipient whose immunosuppressive therapy was discontinued for 8 months | | | | 1988 | | pubmed | title/abstract | irrelevant |
| 612 | Roy SL | An interesting case note. Abuse of antibiotics and corticosteroids | | | | 1965 | | pubmed | title/abstract | irrelevant |
| 613 | Coppola M | Relapsing polychondritis: an unusual cause of painful auricular swelling | | | | 1992 | | pubmed | title/abstract | irrelevant |
| 614 | Say F | Comparison of platelet-rich plasma and steroid injection in the treatment of plantar fasciitis | | | | 2014 | | pubmed | title/abstract | irrelevant |
| 615 | Desmots F | Serious complication of postextubation laryngeal oedema treated by corticosteroids: septic cricoid chondronecrosis | | | | 2014 | | pubmed | title/abstract | irrelevant |
| 616 | Schwartz TR | Systemic corticosteroids for orbital complications of pediatric rhinosinusitis: A systematic review | | | | 2023 | | pubmed | title/abstract | Review |
| 617 | Leung R | Listeria brain abscess associated with steroid therapy: successful non-surgical treatment | | | | 1987 | | pubmed | title/abstract | irrelevant |
| 618 | Dineen P | Fusidate sodium in treatment of surgical infections | | | | 1968 | | pubmed | title/abstract | irrelevant |
| 619 | Duniewicz M | [Acute disseminated aspergillosis] | | | | 1968 | | pubmed | title/abstract | irrelevant |
| 620 | Salafia A | Comment: ulnar abscess--4 months after release from control with paucibacillary-multidrug therapy | | | | 1998 | | pubmed | title/abstract | irrelevant |
| 621 | STELZNER F | [Disposition to local infection due to adrenalin and hydrocortisone] | | | | 1962 | | pubmed | title/abstract | irrelevant |
| 622 | Strohecker J | Exacerbation of brain abscess during exclusive treatment with steroids, demonstrated by computerised tomography | | | | 1985 | | pubmed | title/abstract | irrelevant |
| 623 | Stelzner F | [Cortisone and infection] | | | | 1969 | | pubmed | title/abstract | irrelevant |
| 624 | Burney RE | Late appearance of intra-abdominal abscesses after total colectomy for inflammatory bowel disease | | | | 1979 | | pubmed | title/abstract | irrelevant |
| 625 | ROMANI JD | [Contribution to the study of the antiphlogistic and pro-infectious effects of glycocorticoids; spontaneous abscess of the liver caused by cortisone in the rat] | | | | 1953 | | pubmed | title/abstract | irrelevant |
| 626 | Brun-Buisson CJ | Haemolytic streptococcal gangrene and non-steroidal anti-inflammatory drugs | | | | 1985 | | pubmed | title/abstract | irrelevant |
| 627 | Sangster W | Outcomes of early ileocolectomy after percutaneous drainage for perforated ileocolic Crohn's disease | | | | 2016 | | pubmed | title/abstract | irrelevant |
| 628 | Veyssier-Belot C | [Eosinophilic fasciitis and metastatic choroïdal melanoma: a paraneoplastic syndrome?] | | | | 2008 | | pubmed | title/abstract | irrelevant |
| 629 | BROSS W | [Local administration of ultracortenol in conservative therapy of chronic lung abscesses; preliminary report] | | | | 1959 | | pubmed | title/abstract | irrelevant |
| 630 | Kim SH | Wells' syndrome related to Mycoplasma pneumoniae in a 5-year-old boy | | | | 2020 | | pubmed | title/abstract | irrelevant |
| 631 | Rischin A | Immune checkpoint inhibitor-induced lymphocytic fasciitis | | | | 2018 | | pubmed | title/abstract | irrelevant |
| 632 | Turnier L | Fatal Streptococcus viridans (S. oralis) aortic prosthetic valve endocarditis (PVE) with paravalvular abscesses related to steroids | | | | 2009 | | pubmed | title/abstract | irrelevant |
| 633 | Ollivier Y | [Palmar fasciitis after carboplatin administration for an ovarian adenocarcinoma] | | | | 2006 | | pubmed | title/abstract | irrelevant |
| 634 | Pessach I | Wells' syndrome with a clinicopathological correlation associated with mantle-cell lymphoma | | | | 2016 | | pubmed | title/abstract | irrelevant |
| 635 | Gaucher S | Sarcoidosis and Wound Healing After Cellulitis of the Lower Limb: Is Methotrexate Responsible for Skin Graft Failure? | | | | 2017 | | pubmed | title/abstract | irrelevant |
| 636 | van Ammers PM | Necrotising fasciitis after caesarean section--association with non-steroidal anti-inflammatory drugs. A report of 3 cases | | | | 1991 | | pubmed | title/abstract | irrelevant |
| 637 | Behera B | Eosinophilic fasciitis associated with Raynaud's phenomenon, esophageal dysmotility, positive antinuclear antibody and anti-neutrophil cytoplasmic antibody | | | | 2018 | | pubmed | title/abstract | irrelevant |
| 638 | Mendoza C | [Necrotizing fasciitis and toxic shock syndrome due to Streptococcus pyogenes after intramuscular injection] | | | | 2019 | | pubmed | title/abstract | irrelevant |
| 639 | Javier RM | Fatal Aspergillus fumigatus Myositis in an immunocompetent patient | | | | 2001 | | pubmed | title/abstract | irrelevant |
| 640 | George B | A Case of Pyoderma Gangrenosum of Head and Neck Mimicking Purulent Cellulitis | | | | 2021 | | pubmed | title/abstract | irrelevant |
| 641 | Elbistanlı MS | Vit D deficiency is a possible risk factor in ARS | | | | 2017 | | pubmed | title/abstract | irrelevant |
| 642 | Cheng YF | Spontaneous cellulitis in adults with idiopathic nephrotic syndrome | | | | 1998 | | pubmed | title/abstract | irrelevant |
| 643 | Busch RF | Ludwig angina: early aggressive therapy | | | | 1999 | | pubmed | title/abstract | letter to editor |
| 644 | Govindarajan R | Posterior tibial nerve block in the therapeutic management of painful calcaneal spur (plantar fasciitis): a preliminary experience | | | | 2003 | | pubmed | duplicate | duplicate |
| 645 | KLEIMANN B | [The importance of vitamins in the treatment of dental and oral diseases] | | | | 1954 | | pubmed | title/abstract | irrelevant |
| 646 | Courtney SP | The use of fusidic acid gel in pilonidal abscess treatment: cure, recurrence and failure rates | | | | 1986 | | pubmed | title/abstract | irrelevant |
| 647 | Dixon EE | Ludwig Angina Caused by MRSA: A New Syndrome | | | | 2016 | | pubmed | title/abstract | irrelevant |
| 648 | Giessel M | Primary cutaneous histoplasmosis: a new presentation | | | | 1980 | | pubmed | title/abstract | irrelevant |
| 649 | Cantalejo-Moreira M | Multiple cerebral abscesses in a woman with giant cell arteritis | | | | 2000 | | pubmed | title/abstract | irrelevant |
| 650 | Galeev MA | [Surgical complications in the local use of hydrocortisone] | | | | 1972 | | pubmed | title/abstract | irrelevant |
| 651 | Ilyas F | Successful low-dose corticosteroid treatment of aggressive pyoderma gangrenosum with irritable bowel syndrome: a case report | | | | 2022 | | pubmed | title/abstract | irrelevant |
| 652 | Zhou H | A Mendelian Randomization Study of the Connection Between Exogenous Hormones and Perianal Abscess in Pediatric Patients | | | | 2025 | | pubmed | title/abstract | irrelevant |
| 653 | Konkina NT | [The functional state of the adrenal cortex in children with chronic tonsillitis and peritonsillitis] | | | | 1968 | | pubmed | title/abstract | irrelevant |
| 654 | van der Poel NA | Impact of superior ophthalmic vein thrombosis: a case series and literature review | | | | 2019 | | pubmed | duplicate | duplicate |
| 655 | LICHTWITZ A | [Anti-inflammatory and Inflammatory hormones] | | | | 1953 | | pubmed | title/abstract | irrelevant |
| 656 | Ogden J | Plantar fasciopathy and orthotripsy: the effect of prior cortisone injection | | | | 2005 | | pubmed | title/abstract | irrelevant |
| 657 | Vijayan N | Chemical epidural abscess: case report | | | | 1971 | | pubmed | title/abstract | irrelevant |
| 658 | Brunner HI | Efficacy and Safety of Tocilizumab for Polyarticular-Course Juvenile Idiopathic Arthritis in the Open-Label Two-Year Extension of a Phase III Trial | | | | 2021 | | pubmed | title/abstract | irrelevant |
| 659 | Katsu M | The indication of the combined use of adrenocortical steroids and antibiotics in infectious diseases | | | | 1968 | | pubmed | title/abstract | irrelevant |
| 660 | Cherenko SM | [Long-term asymptomatic abscess arising as a result of injections of corticosteroid hormones in a child with Addison's disease] | | | | 1993 | | pubmed | title/abstract | irrelevant |
| 661 | LANZA B | [Massive doses of cortisone and infectious diseases in laboratory animals] | | | | 1951 | | pubmed | title/abstract | irrelevant |
| 662 | BIAGI F | [Cholesterol in the experimental production of amebic liver abscesses] | | | | 1961 | | pubmed | title/abstract | irrelevant |
| 663 | Dwyer KM | Real pain in the neck: giant cell arteritis presenting with non-necrotising fasciitis and fever | | | | 2019 | | pubmed | title/abstract | irrelevant |
| 664 | Atak T | Strategies to treat idiopathic granulomatous mastitis: retrospective analysis of 40 patients | | | | 2015 | | pubmed | title/abstract | irrelevant |
| 665 | Reiss S | Risk Factors in the Development of Oral Bisphosphonate-induced Osteonecrosis | | | | 2015 | | pubmed | title/abstract | irrelevant |
| 666 | Lunga II | [Complications of local use of hydrocortisone in surgery] | | | | 1976 | | pubmed | title/abstract | irrelevant |
| 667 | Rafiei N | Retinal fibrovascular proliferation associated with Nocardia subretinal abscess | | | | 2006 | | pubmed | title/abstract | irrelevant |
| 668 | KOVANEV VA | [Change in the formula of the blood in patients subjected to surgical intervention on organs of the thorax in combination with cortisone and hydrocortisone therapy] | | | | 1962 | | pubmed | title/abstract | irrelevant |
| 669 | Huang RC | Cervical epidural abscess after epidural steroid injection | | | | 2004 | | pubmed | title/abstract | irrelevant |
| 670 | Ritchie IC | Technique to improve the rate of healing of incised abscesses | | | | 1972 | | pubmed | title/abstract | irrelevant |
| 671 | Corti MA | Cutaneous reactive angiomatosis with combined histological pattern mimicking a cellulitis | | | | 2013 | | pubmed | title/abstract | irrelevant |
| 672 | Assimakopoulos SF | Toxoplasma gondii meningoencephalitis without cerebral MRI findings in a patient with ulcerative colitis under immunosuppressive treatment | | | | 2015 | | pubmed | title/abstract | irrelevant |
| 673 | Gabay S | Nocardia cyriacigeorgica brain abscess in a patient on low dose steroids: a case report and review of the literature | | | | 2022 | | pubmed | title/abstract | irrelevant |
| 674 | Milenkovic A | Adrenal crisis provoked by dental infection: case report and review of the literature | | | | 2010 | | pubmed | title/abstract | irrelevant |
| 675 | Stein KM | Lung abscess. Complication of high dosage steroids in bullous pemphigoid | | | | 1970 | | pubmed | title/abstract | irrelevant |
| 676 | Sepić-Bilić T | Dental inflammation and central retinitis--it is important to protect the retina from the harmful sunlight | | | | 2008 | | pubmed | title/abstract | irrelevant |
| 677 | Mohamed MA | Does local steroid injection have a prognostic value for endoscopic plantar fascia release in chronic plantar fasciopathy? | | | | 2025 | | pubmed | title/abstract | irrelevant |
| 678 | Gautschi OP | Images in clinical medicine. Methicillin-resistant Staphylococcus aureus abscess after intramuscular steroid injection | | | | 2006 | | pubmed | title/abstract | irrelevant |
| 679 | James P | The match day use of ultrasound during professional football finals matches | | | | 2010 | | pubmed | title/abstract | irrelevant |
| 680 | Hu T | The clinical characteristic and outcome of skin and soft tissue infection in immunosuppressive patients with nephrotic syndrome | | | | 2020 | | pubmed | title/abstract | irrelevant |
| 681 | Lamkin RH | An outpatient medical treatment protocol for peritonsillar abscess | | | | 2006 | | pubmed | title/abstract | irrelevant |
| 682 | Nakamura A | [Case of diabetes mellitus associated with cervical pyogenic spondylitis and meningoencephalitis secondary to retropharyngeal abscess caused by Streptococcus pneumoniae] | | | | 2008 | | pubmed | title/abstract | irrelevant |
| 683 | Pai VS | Rupture of the plantar fascia | | | | 1996 | | pubmed | title/abstract | irrelevant |
| 684 | Montero Muñoz J | [Bilateral psoas abscess and steroid myopathy] | | | | 2021 | | pubmed | title/abstract | irrelevant |
| 685 | Götz F | [Cervical epidural abscess following lumbar epidural steroid injections] | | | | 2009 | | pubmed | title/abstract | irrelevant |
| 686 | Komar MD | [Complications in local hydrocortisone therapy] | | | | 1969 | | pubmed | title/abstract | irrelevant |
| 687 | Gill NJ | Histopathology of hepatic amoebiasis in guinea-pigs infected through intracaecal and intramesenteric routes | | | | 1985 | | pubmed | title/abstract | irrelevant |
| 688 | Cohen L | The dental management of patients with medical problems | | | | 1975 | | pubmed | title/abstract | irrelevant |
| 689 | Balatsouras DG | Lingual abscess: diagnosis and treatment | | | | 2004 | | pubmed | title/abstract | irrelevant |
| 690 | Misago N | Palisaded neutrophilic granulomatous dermatitis caused by cellulitis in a patient with systemic lupus erythematosus | | | | 2011 | | pubmed | title/abstract | irrelevant |
| 691 | Franklin A | Fusidic acid gel in the treatment of abscesses | | | | 1974 | | pubmed | title/abstract | irrelevant |
| 692 | Rotstein I | Prevalence of periapical abscesses in patients with systemic lupus erythematosus | | | | 2022 | | pubmed | duplicate | duplicate |
| 693 | Rathinam SR | Sympathetic ophthalmia following postoperative bacterial endophthalmitis: a clinicopathologic study | | | | 2006 | | pubmed | title/abstract | irrelevant |
| 694 | Botelho J | Vitamin D Deficiency and Oral Health: A Comprehensive Review | | | | 2020 | | pubmed | title/abstract | irrelevant |
| 695 |  | Plantar fasciitis. Repeated corticosteroid injections are safe | | | | 1998 | | pubmed | title/abstract | irrelevant |
| 696 | Lai TW | Ultrasonography and clinical outcome comparison of extracorporeal shock wave therapy and corticosteroid injections for chronic plantar fasciitis: A randomized controlled trial | | | | 2018 | | pubmed | duplicate | duplicate |
| 697 | Latronica RJ | Septic emboli and pulmonary abscess secondary to odontogenic infection | | | | 1973 | | pubmed | duplicate | duplicate |
| 698 | Thapa SS | Common Bite-Bizarre Rash | | | | 2018 | | pubmed | title/abstract | irrelevant |
| 699 | Waldman SD | Complications of cervical epidural nerve blocks with steroids: a prospective study of 790 consecutive blocks | | | | 1989 | | pubmed | title/abstract | irrelevant |
| 700 | GUTMAN D | LUDWIG'S ANGINA: REPORT OF TWO CASES | | | | 1965 | | pubmed | title/abstract | irrelevant |
| 701 | Tamura S | [Re-evaluation of the carrageenin-induced abscess model as a screening method for anti-inflammatory agents] | | | | 1984 | | pubmed | title/abstract | irrelevant |
| 702 | Thompson JW | Spontaneous perforation of the esophagus as a manifestation of dermatomyositis | | | | 1984 | | pubmed | title/abstract | irrelevant |
| 703 | Moadel K | Psoriatic corneal abscess | | | | 1995 | | pubmed | title/abstract | irrelevant |
| 704 | Seifarth C | Therapy of complicated Crohn's disease during pregnancy--an interdisciplinary challenge | | | | 2014 | | pubmed | title/abstract | irrelevant |
| 705 | Ito T | A case of aseptic abscesses syndrome treated with corticosteroids and TNF-alpha blockade | | | | 2013 | | pubmed | title/abstract | irrelevant |
| 706 | Darley MD | Spinal epidural abscess following minimally invasive dental examination in a rheumatoid arthritis patient receiving methotrexate, glucocorticoids, and anti-tumor necrosis factor therapy | | | | 2015 | | pubmed | title/abstract | irrelevant |
| 707 | Peña-Santos G | [Idiopathic granulomatous mastitis treated with steroids and methotrexate] | | | | 2011 | | pubmed | title/abstract | irrelevant |
| 708 | Nekachalov VV | [Morphological changes in the teeth and jaws in intramuscular cortisone therapy and the possibility of the reversal of these changes (experimental research)] | | | | 1967 | | pubmed | title/abstract | irrelevant |
| 709 | MARTELLI A | [Epidural abscess following gluteal abscess occurring during cortisone therapy] | | | | 1958 | | pubmed | title/abstract | irrelevant |
| 710 | Toba T | Multiple ulcerative colitis-associated aseptic abscesses successfully treated with infliximab: a case report | | | | 2023 | | pubmed | title/abstract | irrelevant |
| 711 | Little K | What is causing this patient's multiple skin abscesses? | | | | 2018 | | pubmed | title/abstract | irrelevant |
| 712 | Pfarschner W | [Hidden symptoms in long-term cortisone therapy] | | | | 1967 | | pubmed | title/abstract | irrelevant |
| 713 | Sabel M | Enlargement of a chronic aseptic lumbar epidural abscess by intraspinal injections--a rare cause of progressive paraparesis | | | | 2000 | | pubmed | title/abstract | irrelevant |
| 714 | GOUNELLE H | [Calcium precipitation in intramuscular fixation abscess during vitamin D2 poisoning] | | | | 1951 | | pubmed | title/abstract | irrelevant |
| 715 | Stranzenbach R | [Necrotising fasciitis following intragluteal injection of mepivacaine and triamcinolone] | | | | 2013 | | pubmed | title/abstract | irrelevant |
| 716 | Saigal G | Thoracic intradural Aspergillus abscess formation following epidural steroid injection | | | | 2004 | | pubmed | title/abstract | irrelevant |
| 717 | Stein KM | Occult lung abscess complicating high-dosage corticosteroid therapy | | | | 1972 | | pubmed | title/abstract | irrelevant |
| 718 | Dohi T | Combination Therapy for a Severe Axillary Keloid with Abscesses: A Case Report | | | | 2023 | | pubmed | title/abstract | irrelevant |
| 719 | BISTROM O | The injurious effect of cortisone on destructive inflammation | | | | 1955 | | pubmed | title/abstract | irrelevant |
| 720 | Kerr C | Disseminated cryptococcal infection initially presenting as cryptococcal cellulitis in an HIV-negative patient on long-term steroids | | | | 2018 | | pubmed | title/abstract | irrelevant |
| 721 | Cannon PS | A multi-centre case series investigating the aetiology of hypertrophic pachymeningitis with orbital inflammation | | | | 2011 | | pubmed | duplicate | duplicate |
| 722 | Scaglione F | Flumethasone pivalate (Locorten) in the treatment of oral diseases | | | | 1985 | | pubmed | title/abstract | irrelevant |
| 723 | Kaswan KK | Nocardia infection in a renal transplant recipient | | | | 2011 | | pubmed | title/abstract | irrelevant |
| 724 | Naldi L | Paecilomyces marquandii cellulitis in a kidney transplant patient | | | | 2000 | | pubmed | title/abstract | irrelevant |
| 725 | Fraser DW | Disseminated mycobacterium kansasii infection presenting as cellulitis in a recipient of a renal homograft | | | | 1975 | | pubmed | title/abstract | irrelevant |
| 726 | Bellmann H | [Severe phlegmonous suppuration of a false cecal diverticulum with imminent perforation following long-term prednisone therapy--etiology of acute appendicitis symptomatology] | | | | 1969 | | pubmed | title/abstract | irrelevant |
| 727 | Valenton MJ | A corneal abscess due to the fungus Botryodiplodia theobromae | | | | 1975 | | pubmed | title/abstract | irrelevant |
| 728 | GIARETTA D | [Different inhibitory action of cortisone and desoxycorticosterone on experimentally induced granulation tissue] | | | | 1960 | | pubmed | title/abstract | irrelevant |
| 729 | Yamagishi S | Tuberculous Addison's disease complicated by a recurrent subcutaneous cold abscess during treatment for tuberculosis | | | | 1998 | | pubmed | title/abstract | irrelevant |
| 730 | Saleh S | Group A streptococcus necrotising fasciitis from a levonorgestrel-containing intrauterine system ('Mirena' coil) | | | | 2011 | | pubmed | title/abstract | irrelevant |
| 731 | Sharma S | Tuberculous Adrenal Abscess Presenting as Adrenal Insufficiency in a 4-Year-Old Boy | | | | 2019 | | pubmed | title/abstract | irrelevant |
| 732 | Wallach D | [Neutrophilic dermatoses] | | | | 2005 | | pubmed | title/abstract | irrelevant |
| 733 | MORELLINI M | [Modifications induced by androisoxazole in the nitrogen metabolism of subjects with chronic pulmonary diseases] | | | | 1961 | | pubmed | title/abstract | irrelevant |
| 734 | Cahill D | Ludwig angina | | | | 2002 | | pubmed | title/abstract | unclear |
| 735 | Krauze J | [Suppurative complication with severe course after intra-articular injection of hydrocortisone in a patient with rheumatoid arthritis and diabetes] | | | | 1968 | | pubmed | title/abstract | irrelevant |
| 736 | Sánchez-Legaza E | [Hemilingual abscess] | | | | 2006 | | pubmed | title/abstract | irrelevant |
| 737 | Kaur A | Nipple eczema in an adolescent girl presenting with persistent unilateral nipple discharge | | | | 2020 | | pubmed | title/abstract | irrelevant |
| 738 | Abraham S | Ulnar nerve abscess in a multibacillary patient during post-multidrug therapy surveillance | | | | 1997 | | pubmed | title/abstract | irrelevant |
| 739 | Babaei-Ghazani A | Comparison of Ultrasound-Guided Local Ozone (O2-O3) Injection vs Corticosteroid Injection in the Treatment of Chronic Plantar Fasciitis: A Randomized Clinical Trial | | | | 2019 | | pubmed | duplicate | duplicate |
| 740 | Johnson KF | Splenomegaly and an abnormal peripheral smear associated with the use of danazol: case report | | | | 1984 | | pubmed | title/abstract | irrelevant |
| 741 | Safa G | Cutaneous aseptic neutrophilic abscesses and Yersinia enterocolitica infection in a case subsequently diagnosed as Crohn's disease | | | | 2008 | | pubmed | title/abstract | irrelevant |
| 742 | Tapalaga G | The Impact of Prenatal Vitamin D on Enamel Defects and Tooth Erosion: A Systematic Review | | | | 2023 | | pubmed | title/abstract | irrelevant |
| 743 | Ritchie GM | Dental manifestations of pseudohypoparathyroidism | | | | 1965 | | pubmed | title/abstract | irrelevant |
| 744 | Raissi G | Ultrasound-Guided Injection of Dextrose Versus Corticosteroid in Chronic Plantar Fasciitis Management: A Randomized, Double-Blind Clinical Trial | | | | 2023 | | pubmed | duplicate | duplicate |
| 745 | Clyman BB | Selected periarticular soft tissue problems in the elderly | | | | 2003 | | pubmed | title/abstract | irrelevant |
| 746 | Beamish D | Delayed adverse responses to both methohexitone and Althesin | | | | 1980 | | pubmed | title/abstract | irrelevant |
| 747 | Burford RG | Anti-inflammatory activity of alkoxyglycerols in rats | | | | 1968 | | pubmed | title/abstract | irrelevant |
| 748 | BENITZ KF | THE CARRAGEENIN-INDUCED ABSCESS AS A NEW TEST FOR ANTI-INFLAMMATORY ACTIVITY OF STEROIDS AND NONSTEROIDS | | | | 1963 | | pubmed | title/abstract | irrelevant |
| 749 | COURT JM | Idiopathic pulmonary haemosiderosis | | | | 1962 | | pubmed | title/abstract | irrelevant |
| 750 | Vatutin NT | [Complications of steroid therapy] | | | | 1976 | | pubmed | title/abstract | irrelevant |
| 751 | Sanchez TG | Septic thrombosis of orbital vessels due to cutaneous nasal infection | | | | 1997 | | pubmed | title/abstract | irrelevant |
| 752 | Tsuji K | Protothecosis in a patient with systemic lupus erythematosus | | | | 1993 | | pubmed | title/abstract | irrelevant |
| 753 | Scott GM | Cryptogenic brain abscess | | | | 1976 | | pubmed | title/abstract | irrelevant |
| 754 | D-FINCZICZKY K | [Successful cortisone therapy in cyclic agranulocytosis complicated with lung abscess] | | | | 1956 | | pubmed | title/abstract | irrelevant |
| 755 | REIFF S | Acute suppurative cholecystitis, with rupture of gall-bladder and liver abscess formation, during administration of cortisone | | | | 1953 | | pubmed | title/abstract | irrelevant |
| 756 | Dye ES | Characterization of a bactericidal lipid developing within staphylococcal abscesses | | | | 1981 | | pubmed | title/abstract | irrelevant |
| 757 | Cleland JA | Manual physical therapy and exercise versus electrophysical agents and exercise in the management of plantar heel pain: a multicenter randomized clinical trial | | | | 2009 | | pubmed | duplicate | duplicate |
| 758 | Elias EG | Splenectomy in Felty's syndrome | | | | 1969 | | pubmed | title/abstract | irrelevant |
| 759 | Breneman E | Aspergillosis of the CNS presenting as aseptic meningitis | | | | 1992 | | pubmed | title/abstract | irrelevant |
| 760 | ROMANI JD | [Local inhibiting effect of hydrocortisone on fixation abscess in rats] | | | | 1954 | | pubmed | title/abstract | irrelevant |
| 761 | Delavari N | Resolution of innumerable cerebral Nocardia paucivorans abscesses after medical management | | | | 2016 | | pubmed | title/abstract | irrelevant |
| 762 | Ladeira SL | Role of Pasteurella granulomatis and Dermatobia hominis in the etiology of lechiguana in cattle | | | | 1996 | | pubmed | title/abstract | irrelevant |
| 763 | Uzuner N | Follicular bronchiolitis associated with lung abscess in an eight-year-old girl | | | | 2007 | | pubmed | title/abstract | irrelevant |
| 764 | Alcock E | Facet joint injection: a rare form cause of epidural abscess formation | | | | 2003 | | pubmed | title/abstract | irrelevant |
| 765 | Edupuganti S | Fusarium falciforme vertebral abscess and osteomyelitis: case report and molecular classification | | | | 2011 | | pubmed | title/abstract | irrelevant |
| 766 | Peker E | Periorbital cellulitis caused by Bacillus thuringiensis | | | | 2010 | | pubmed | title/abstract | irrelevant |
| 767 | ROMANI JD | [Comparative inhibiting action of cortisone and hydrocortisone on inflammatory granuloma and local and generalized eosinophilia during fixation abscess in the rat] | | | | 1953 | | pubmed | title/abstract | irrelevant |
| 768 | SCHEBAT L | [The association of synthetic corticosteroids and Delbet's stock vaccine bouillon in inflammation of the pelvic connective tissue] | | | | 1960 | | pubmed | title/abstract | irrelevant |
| 769 | Karimzadeh A | Autologous whole blood versus corticosteroid local injection in treatment of plantar fasciitis: A randomized, controlled multicenter clinical trial | | | | 2017 | | pubmed | duplicate | duplicate |
| 770 | Acevedo JI | Complications of plantar fascia rupture associated with corticosteroid injection | | | | 1998 | | pubmed | title/abstract | irrelevant |
| 771 | Celik D | Joint Mobilization and Stretching Exercise vs Steroid Injection in the Treatment of Plantar Fasciitis: A Randomized Controlled Study | | | | 2016 | | pubmed | duplicate | duplicate |
| 772 | Chan ST | Spinal epidural abscess following steroid injection for sciatica. Case report | | | | 1989 | | pubmed | title/abstract | irrelevant |
| 773 | Bonner MJ | Primary cellulitis due to Serratia marcescens | | | | 1983 | | pubmed | title/abstract | irrelevant |
| 774 | DESJACQUES P | [SPLENIC ABSCESS DURING THE POSTPARTUM PERIOD] | | | | 1964 | | pubmed | title/abstract | irrelevant |
| 775 | Anderson BL | Idiopathic vertebral abscess in pregnancy: case report and literature review | | | | 2007 | | pubmed | title/abstract | irrelevant |
| 776 | Zhu TH | Cryptococcal cellulitis on the shin of an immunosuppressed patient | | | | 2016 | | pubmed | title/abstract | irrelevant |
| 777 | SACINO G | [On the local treatment of cold sternal abscess with vitamin D2 in high doses] | | | | 1948 | | pubmed | duplicate | duplicate |
| 778 | Wiersbitzky S | [Effectiveness of various therapeutic regimens in the treatment of childhood pneumonias] | | | | 1982 | | pubmed | title/abstract | irrelevant |
| 779 | Kresbach H | [On the differential diagnosis between dermatitis herpetiformis Duhring and bullous pemphigoid] | | | | 1967 | | pubmed | title/abstract | irrelevant |
| 780 | Hemady RK | Interface abscess after epikeratoplasty | | | | 1990 | | pubmed | title/abstract | irrelevant |
| 781 | Noguchi E | Bullous pemphigoid complicated by necrotising fasciitis successfully treated with systemic corticosteroids and antibiotics in combination with i.v. immunoglobulin | | | | 2018 | | pubmed | title/abstract | irrelevant |
| 782 | Yukawa K | [Protein anabolic effect on the lumbar sympathetic ganglion cells in the lower pelvic abscess] | | | | 1967 | | pubmed | title/abstract | irrelevant |
| 783 | BROWN PP | Two cases of Staphylococcal psoas abscess in patients of cortisone therapy | | | | 1956 | | pubmed | title/abstract | irrelevant |
| 784 | Mansour K | Early and late assessment of internal drainage of chronic dacryocystitis | | | | 2003 | | pubmed | title/abstract | irrelevant |
| 785 | Lee SC | Multiple sterile abscesses in antiphospholipid antibody syndrome | | | | 2001 | | pubmed | title/abstract | irrelevant |
| 786 | Somali MH | Pituitary abscess presenting with cranial nerve paresis. Case report and review of literature | | | | 2001 | | pubmed | title/abstract | irrelevant |
| 787 | Kawashima I | [A case report--typhoid fever complicated with liver and gallbladder abscess, treated for long-time as fever of unknown origin] | | | | 1997 | | pubmed | title/abstract | irrelevant |
| 788 | Breton A | Prediction of clinical response to corticosteroid or platelet-rich plasma injection in plantar fasciitis with MRI: A prospective, randomized, double-blinded study | | | | 2022 | | pubmed | duplicate | duplicate |
| 789 | Lee JY | Epidural abscess formation with an atypical pathogen following epidural steroid injection: A case report | | | | 2022 | | pubmed | title/abstract | irrelevant |
| 790 | Quin JD | Thyroid abscess complicating subacute thyroiditis: a consequence of steroid therapy? | | | | 1992 | | pubmed | title/abstract | irrelevant |
| 791 | CAMPBELL C | LIGNEOUS PELVIC CELLULITIS | | | | 1965 | | pubmed | title/abstract | irrelevant |
| 792 | Unglaub F | [Necrotizing fasciitis following therapeutic injection in a shoulder joint] | | | | 2005 | | pubmed | title/abstract | irrelevant |
| 793 | Spelsberg F | [Management of staphylococcal infections using Fucidin] | | | | 1966 | | pubmed | title/abstract | irrelevant |
| 794 | O'Day DM | Deep fungal corneal abscess. Combined corticosteroid therapy | | | | 1971 | | pubmed | title/abstract | irrelevant |
| 795 | Shigemura K | Retroperitoneal abscess perforating into the thoracic cavity in an immunocompromised host | | | | 2008 | | pubmed | title/abstract | irrelevant |
| 796 | Trillo C | Primary anastomosis in the treatment of acute disease of the unprepared left colon | | | | 1998 | | pubmed | title/abstract | irrelevant |
| 797 | Alanazi T | Hepatic vasculitis mimicking liver abscesses in a patient with systemic lupus erythematosus | | | | 2009 | | pubmed | title/abstract | irrelevant |
| 798 | Winkelmann RK | Carpal tunnel syndrome in cutaneous connective tissue disease: generalized morphea, lichen sclerosus, fasciitis, discoid lupus erythematosus, and lupus panniculitis | | | | 1982 | | pubmed | title/abstract | irrelevant |
| 799 | Connolly SM | Scleroderma and L-tryptophan: a possible explanation of the eosinophilia-myalgia syndrome | | | | 1990 | | pubmed | title/abstract | irrelevant |
| 800 | SAITO H | [Effect of cortisone and ACTH on infection. 2. Survival time and pathohistological study] | | | | 1962 | | pubmed | title/abstract | irrelevant |
| 801 | Jonville-Béra AP | [Do non-steroidal anti-inflammatory agents favor the occurrence of necrotizing fasciitis?] | | | | 2001 | | pubmed | title/abstract | irrelevant |
| 802 | Craft JC | A randomized, double-blind phase 2 study comparing the efficacy and safety of an oral fusidic acid loading-dose regimen to oral linezolid for the treatment of acute bacterial skin and skin structure infections | | | | 2011 | | pubmed | title/abstract | irrelevant |
| 803 | Lawson TM | Grand round--University Hospital of Wales. Focal myositis mimicking acute psoas abscess | | | | 1997 | | pubmed | title/abstract | irrelevant |
| 804 | Feng T | Clinical significance of dynamic variation of low cholesterol and its prognostic value in patients with pyogenic liver abscesses: a retrospective study | | | | 2023 | | pubmed | title/abstract | irrelevant |
| 805 | Provotorov VM | [The clinical efficacy of treating patients with nonspecific lung diseases using low-energy laser irradiation and intrapulmonary drug administration] | | | | 1991 | | pubmed | title/abstract | irrelevant |
| 806 | Rush ML | Kimura disease: a case report of a rare illness presenting as a common complaint | | | | 2019 | | pubmed | title/abstract | irrelevant |
| 807 | Bennani-Baïti AA | Cervicofacial cellulitis: The impact of non-steroidal anti-inflammatory drugs. A study of 70 cases | | | | 2015 | | pubmed | title/abstract | irrelevant |
| 808 | Reynolds RD | Exacerbation of tinea corporis during treatment with 1% clotrimazole/0.05% betamethasone diproprionate (Lotrisone) | | | | 1991 | | pubmed | title/abstract | irrelevant |
| 809 | Black KL | Cerebral abscess: loss of computed tomographic enhancement with steroids. Case report | | | | 1984 | | pubmed | title/abstract | irrelevant |
| 810 | Crawford F | Steroid injection for heel pain: evidence of short-term effectiveness. A randomized controlled trial | | | | 1999 | | pubmed | duplicate | duplicate |
| 811 | Ng F | A rare case of Behçet disease with generalised myositis, cardiomyositis and necrotising fasciitis | | | | 2016 | | pubmed | title/abstract | irrelevant |
| 812 | Makino K | Multiple Skin Abscesses Caused by Rhizopus sp. Infection after Candida albicans Infection in an Immunocompromised Patient | | | | 2019 | | pubmed | title/abstract | irrelevant |
| 813 | Jeong JH | Multiple Brain Abscesses Caused by Nocardia asiatica in a Patient With Systemic Lupus Erythematosus: The First Case Report and Literature Review | | | | 2017 | | pubmed | title/abstract | irrelevant |
| 814 | Jordan AJ | Multiple aseptic splenic abscesses in a 15 year old patient | | | | 2014 | | pubmed | title/abstract | irrelevant |
| 815 | Molina-Infante J | Successful pelvic abscess drainage by cecal biopsies in Crohn's disease | | | | 2011 | | pubmed | title/abstract | irrelevant |
| 816 | Cooper J | Subcutaneous abscess following steroid injection of chalazia | | | | 1986 | | pubmed | title/abstract | irrelevant |
| 817 | André M | Retropharyngeal and splenic aseptic abscesses treated with prednisone and cyclophosphamide in a patient with ulcerative colitis | | | | 2003 | | pubmed | title/abstract | irrelevant |
| 818 | Vogeser M | Steroid-induced invasive aspergillosis with thyroid gland abscess and positive blood cultures | | | | 1998 | | pubmed | title/abstract | irrelevant |
| 819 | ROMANI JD | [Effect of locally administered hydrocortisone acetate on blood glycoprotein levels in fixation abscess in the rat; comparative study of alpha 1 and alpha 2 globulin] | | | | 1955 | | pubmed | title/abstract | irrelevant |
| 820 | Schlegel D | [Cortisone treatment in postoperative "parulis"] | | | | 1965 | | pubmed | title/abstract | unclear |
| 821 | Chicago Dental Society | Your diet matters to your teeth and gums | | | | 2010 | | pubmed | title/abstract | irrelevant |
| 822 | Cook NJ | Paraspinal abscess following facet joint injection | | | | 1999 | | pubmed | title/abstract | irrelevant |
| 823 | Auvert J | [Prevesical staphylococcal phlegmon complicating acute prostatitis] | | | | 1967 | | pubmed | title/abstract | irrelevant |
| 824 | Manfredini R | Lung abscess as a complication of steroid treatment in pemphigus vulgaris | | | | 2001 | | pubmed | title/abstract | irrelevant |
| 825 | Ampel NM | Cutaneous abscess caused by Legionella micdadei in an immunosuppressed patient | | | | 1985 | | pubmed | title/abstract | irrelevant |
| 826 | de Andrés J | [Epidural abscess: a complication of the use of corticoids in the treatment for back pain] | | | | 1997 | | pubmed | title/abstract | irrelevant |
| 827 | Berná-Serna JD | Sonographically guided percutaneous intralesional triamcinolone injection: a new treatment for mammillary fistulas: preliminary results | | | | 2012 | | pubmed | title/abstract | irrelevant |
| 828 | Ishida T | [A case of multiple pulmonary arteriovenous fistulae associated with splenic abscess] | | | | 1991 | | pubmed | title/abstract | irrelevant |
| 829 | Friedler S | Ruptured tubo-ovarian abscess complicating transcervical cryopreserved embryo transfer | | | | 1996 | | pubmed | title/abstract | irrelevant |
| 830 | Pohto M | [Treatment of periapical osteitis with chemotherapy and corticoid preparations] | | | | 1965 | | pubmed | title/abstract | irrelevant |
| 831 | Spadafora R | A rare case of neonatal sepsis/meningitis caused by Pasteurella multocida complicated with status epilepticus and focal cerebritis | | | | 2011 | | pubmed | title/abstract | irrelevant |
| 832 | Dissanayake HA | Embolizing pulmonary aspergillosis, mycobacterial & aspergillous splenic abscess and cytomegalovirus co-infection following steroid induced immunosuppression: a case report | | | | 2018 | | pubmed | title/abstract | irrelevant |
| 833 | Balavoine C | [Hypersensitivity pneumonitis and abscess reaction to nontuberculous mycobacteria acquired form jacuzzi aerosol] | | | | 2019 | | pubmed | title/abstract | irrelevant |
| 834 | Self SJ | Follicular occlusion triad | | | | 1970 | | pubmed | title/abstract | irrelevant |
| 835 | Chaussain-Miller C | Dental abnormalities in patients with familial hypophosphatemic vitamin D-resistant rickets: prevention by early treatment with 1-hydroxyvitamin D | | | | 2003 | | pubmed | title/abstract | irrelevant |
| 836 | Enzmann DR | The effect of short-term corticosteroid treatment on the CT appearance of experimental brain abscesses | | | | 1982 | | pubmed | title/abstract | irrelevant |
| 837 | ZEITLHOFER J | [BONE CHANGES IN ALBERS-SCHOENBERG OSTEOSCLEROSIS ASSOCIATED WITH "RICKETS" AND AMINOACIDURIA] | | | | 1963 | | pubmed | title/abstract | irrelevant |
| 838 | STARZL TE | THE REVERSAL OF REJECTION IN HUMAN RENAL HOMOGRAFTS WITH SUBSEQUENT DEVELOPMENT OF HOMOGRAFT TOLERANCE | | | | 1963 | | pubmed | title/abstract | irrelevant |
| 839 | Gujral S | Altered lipid profile in liver amoebiasis and its emendation with metronidazole treatment | | | | 1982 | | pubmed | title/abstract | irrelevant |
| 840 | Chen CM | Comparative efficacy of corticosteroid injection and non-invasive treatments for plantar fasciitis: a systematic review and meta-analysis | | | | 2018 | | pubmed | title/abstract | irrelevant |
| 841 | MONTANDON A | [Large retroesophageal abscess caused by foreign body perforation] | | | | 1953 | | pubmed | title/abstract | irrelevant |
| 842 | SACINO G | [On the local treatment of cold sternal abscess with vitamin D2 in high doses] | | | | 1949 | | pubmed | title/abstract | irrelevant |
| 843 | Sohn KC | [Rectal Ulcer Developed in Systemic Lupus Erythematosus without Ischemic Colitis] | | | | 2019 | | pubmed | title/abstract | irrelevant |
| 844 | Koudahl G | The management of the rectal stump after subtotal colectomy for ulcerative colitis | | | | 1971 | | pubmed | title/abstract | irrelevant |
| 845 | AlHarmi RA | A brain populated with space-occupying lesions: identifying the culprit | | | | 2018 | | pubmed | duplicate | duplicate |
| 846 | Enzmann DR | Staging of human brain abscess by computed tomography | | | | 1983 | | pubmed | title/abstract | irrelevant |
| 847 | Macki M | Mycotic aneurysm and fungal spinal abscess due to tainted steroid injection | | | | 2014 | | pubmed | title/abstract | irrelevant |
| 848 | Abdel-Magied AA | Immunological, histopathological, and ultrastructural evidence of steroid-induced reactivation of chronic murine toxoplasmosis | | | | 2021 | | pubmed | title/abstract | irrelevant |
| 849 | PECAK W | [Contribution to the treatment of pulmonary sarcoidosis with prednisone] | | | | 1962 | | pubmed | title/abstract | irrelevant |
| 850 | TAUBENHAUS M | Parallel inhibition of granulation tissue by cortisone, hydrocortisone, dibenamine and banthine | | | | 1953 | | pubmed | title/abstract | irrelevant |
| 851 | CLARKE PB | WEGENER'S GRANULOMATOSIS | | | | 1964 | | pubmed | title/abstract | irrelevant |
| 852 | RASMUSSEN PE | [Acute frontal sinusitis with subperiosteal abscess treated with Fucidin] | | | | 1962 | | pubmed | title/abstract | irrelevant |
| 853 | Boughrara Z | [Cutaneous infections in bullous pemphigoid patients treated with topical corticosteroids] | | | | 2010 | | pubmed | title/abstract | irrelevant |
| 854 | Frakun VB | [On neutral 17-ketosteroids in patients with acne abscesses] | | | | 1966 | | pubmed | title/abstract | irrelevant |
| 855 | Vakhidov VV | [Corticosteroid therapy in surgery of suppurative diseases] | | | | 1975 | | pubmed | title/abstract | irrelevant |
| 856 | Cooper RG | Mycobacterial infection associated with the use of an anabolic steroid | | | | 1993 | | pubmed | title/abstract | irrelevant |
| 857 | Chiba M | [A case of ulcerative colitis associated with perirectal abscess and steroid withdrawal syndrome] | | | | 1986 | | pubmed | title/abstract | irrelevant |
| 858 | ZONDEK B | Estrone clearance test in infectious hepatitis | | | | 1947 | | pubmed | title/abstract | irrelevant |
| 859 | Ahadi T | Comparison of the Effect of Ultrasound-Guided Injection of Botulinum Toxin Type A and Corticosteroid in the Treatment of Chronic Plantar Fasciitis: A Randomized Controlled Trial | | | | 2022 | | pubmed | duplicate | duplicate |
| 860 | Maeshima K | Behçet's disease complicated by multiple aseptic abscesses of the liver and spleen | | | | 2013 | | pubmed | title/abstract | irrelevant |
| 861 | Owczarczyk-Saczonek A | Aseptic Abscess Syndrome in Rheumatoid Arthritis Patient | | | | 2022 | | pubmed | title/abstract | irrelevant |
| 862 | STUTEVILLE OH | The spread of infections in the head and neck | | | | 1958 | | pubmed | title/abstract | irrelevant |
| 863 | Li S | Clinical effects of extracorporeal shock-wave therapy and ultrasound-guided local corticosteroid injections for plantar fasciitis in adults: A meta-analysis of randomized controlled trials | | | | 2018 | | pubmed | title/abstract | irrelevant |
| 864 | Stortini B | Tubo-Ovarian Abscess in a Virginal Adolescent with Labial Agglutination Due to Lichen Sclerosus | | | | 2017 | | pubmed | title/abstract | irrelevant |
| 865 | Moraitis AG | Hypercalcemia associated with mineral oil-induced sclerosing paraffinomas | | | | 2013 | | pubmed | title/abstract | irrelevant |
| 866 | Kita A | [Ulcerative colitis complicated by pyoderma gangrenosum and multiple aseptic abscesses] | | | | 2022 | | pubmed | title/abstract | irrelevant |
| 867 | Oh SH | Surgery in corticosteroid-dependent asthmatics | | | | 1974 | | pubmed | title/abstract | irrelevant |
| 868 | Pchelin VG | [The clinical effect and dynamics of hormonal homeostasis with the inclusion of plasmosorption in the combined therapy of patients with maxillofacial phlegmons against a background of diabetes mellitus] | | | | 1994 | | pubmed | title/abstract | irrelevant |
| 869 | Fox LE | Disseminated subcutaneous Mycobacterium fortuitum infection in a dog | | | | 1995 | | pubmed | title/abstract | irrelevant |
| 870 | Guo J | Nocardiosis in patients with nephrotic syndrome: a retrospective analysis of 11 cases and a literature review | | | | 2020 | | pubmed | title/abstract | irrelevant |
| 871 | Villanueva-Fernández E | Role of steroids in conservative treatment of parapharyngeal and retropharyngeal abscess in children | | | | 2022 | | pubmed | title/abstract | Neck Infections |
| 872 | Webber SK | Staphylococcal infection under a LASIK flap | | | | 1999 | | pubmed | title/abstract | irrelevant |
| 873 | Raghavendran RR | Subcutaneous calcification following injection of triamcinolone hexacetonide for plantar fasciitis | | | | 2008 | | pubmed | title/abstract | irrelevant |
| 874 | DE VICENTE ARGUELLES A | [Therapeutic action of folliculin and estrogens in stomatology: personal treatment of epulis with diethylstilbestrol] | | | | 1955 | | pubmed | title/abstract | irrelevant |
| 875 | Moulin F | [Managing children skin and soft tissue infections] | | | | 2008 | | pubmed | title/abstract | irrelevant |
| 876 | Heermann R | [Pyoderma gangraenosum. Case report and comparison with necrotizing fasciitis] | | | | 2002 | | pubmed | title/abstract | irrelevant |
| 877 | Smirnov VE | [Possibilities of liquid crystal thermography in the diagnosis of acute paraproctitis] | | | | 1990 | | pubmed | title/abstract | irrelevant |
| 878 | Norris JM | The isolation and enumeration of three feline oral Porphyromonas species from subcutaneous abscesses in cats | | | | 1999 | | pubmed | title/abstract | irrelevant |
| 879 | Brakenbury PH | Comparison of two methods of treating acute abscesses | | | | 1985 | | pubmed | title/abstract | irrelevant |
| 880 | Sakai C | Nocardia asteroides pneumonia, subcutaneous abscess and meningitis in a patient with advanced malignant lymphoma: successful treatment based on in vitro antimicrobial susceptibility | | | | 1999 | | pubmed | title/abstract | irrelevant |
| 881 | Bromage PR | Spinal extradural abscess: pursuit of vigilance | | | | 1993 | | pubmed | title/abstract | irrelevant |
| 882 | Kotowski J | Treatment of axillary abscesses with fusidic acid gel | | | | 1979 | | pubmed | title/abstract | irrelevant |
| 883 | Sonnabend E | [Treatment of apical periodontitis in gangrenous teeth] | | | | 1974 | | pubmed | title/abstract | irrelevant |
| 884 | BOTTERO A | [THE USE OF DIMETHAZINE IN SEBILE TYPES OF BRONCHOPNEUMOPATHIES] | | | | 1964 | | pubmed | title/abstract | irrelevant |
| 885 | ZINI M | [Polycorticoid hormone therapy in convalescence; effectiveness of oral therapy] | | | | 1957 | | pubmed | title/abstract | irrelevant |
| 886 | Holm KS | Eosinophilic dermatitis with edema in nine dogs, compared with eosinophilic cellulitis in humans | | | | 1999 | | pubmed | title/abstract | irrelevant |
| 887 | Ritchie IC | Letter: Packing of abscess cavities | | | | 1973 | | pubmed | title/abstract | irrelevant |
| 888 | Darius EJ | Ulnar abscess: 4 months after release from control with paucibacillary-multidrug therapy | | | | 1997 | | pubmed | title/abstract | irrelevant |
| 889 | Crofford LJ | L-tryptophan implicated in human eosinophilia-myalgia syndrome causes fasciitis and perimyositis in the Lewis rat | | | | 1990 | | pubmed | title/abstract | irrelevant |
| 890 | Arens J | Treating AIDS-associated cerebral toxoplasmosis - pyrimethamine plus sulfadiazine compared with cotrimoxazole, and outcome with adjunctive glucocorticoids | | | | 2007 | | pubmed | title/abstract | irrelevant |
| 891 | Hisano Y | [Antibiotics, tranquilizers and Chinese medicine used in dentistry] | | | | 1984 | | pubmed | title/abstract | irrelevant |
| 892 | Madden BP | Pyogenic psoas abscess: a rare complication after orthotopic heart transplantation | | | | 2002 | | pubmed | title/abstract | irrelevant |
| 893 | Sheu SJ | Risk factors for endogenous endophthalmitis secondary to klebsiella pneumoniae liver abscess: 20-year experience in Southern Taiwan | | | | 2011 | | pubmed | title/abstract | irrelevant |
| 894 | Theophilo F | Brain abscess in childhood | | | | 1985 | | pubmed | title/abstract | irrelevant |
| 895 | Weingärtner L | [Lung abscess formations in children] | | | | 1967 | | pubmed | title/abstract | irrelevant |
| 896 | Monaco WE | A Case of Relapsing Polychondritis Mimicking Hemophagocytic Lymphohistiocytosis After Propionibacterium acnes Infection | | | | 2019 | | pubmed | title/abstract | irrelevant |
| 897 | Janssen AG | Abscess of the lacrimal sac due to chronic or subacute dacryocystitis: treatment with temporary stent placement in the nasolacrimal duct | | | | 2000 | | pubmed | title/abstract | irrelevant |
| 898 | Tazawa J | [A case of splenic abscess occurring in ulcerative colitis and successfully treated by antibiotic therapy] | | | | 1988 | | pubmed | title/abstract | irrelevant |
| 899 | Hawkins DB | Acute epiglottitis in adults | | | | 1973 | | pubmed | title/abstract | irrelevant |
| 900 | Bordel-Gómez MT | [Necrotizing cellulitis as the first manifestation of disseminated cryptococcosis] | | | | 2011 | | pubmed | title/abstract | irrelevant |
| 901 | Ishiki Y | [On three clinical cases of vitamin D resistant rickets] | | | | 1967 | | pubmed | title/abstract | irrelevant |
| 902 | Hinson KR | Photo Quiz: An 85-Year-Old Man with Brain Abscess | | | | 2018 | | pubmed | title/abstract | irrelevant |
| 903 | Fukui S | RS3PE Syndrome with Iliopsoas Bursitis Distinguished from an Iliopsoas Abscess Using a CT-guided Puncture | | | | 2015 | | pubmed | title/abstract | irrelevant |
| 904 | Dijkmans BA | Brain abscess due to Streptobacillus moniliformis and Actinobacterium meyerii | | | | 1984 | | pubmed | title/abstract | irrelevant |
| 905 | DI GIACOMO G | [Curing of cold abscess with large doses of vitamin D 2] | | | | 1956 | | pubmed | title/abstract | irrelevant |
| 906 | Fernández Pérez A | [Paraglottic laryngeal abscesses] | | | | 2002 | | pubmed | title/abstract | irrelevant |
| 907 | Harvey JP | Endogenous bacterial endophthalmitis and subretinal abscess complicating diabetic ketoacidosis | | | | 2018 | | pubmed | title/abstract | irrelevant |
| 908 | Herskovitz I | Caseating granulomas manifesting as aseptic abscesses in the setting of ulcerative colitis | | | | 2018 | | pubmed | title/abstract | irrelevant |
| 909 | Lacruz F | [Cerebral abscess treated with antibiotics and corticosteroids] | | | | 1983 | | pubmed | title/abstract | irrelevant |
| 910 | Toyoda T | [A case of SLE under steroid-treatment who developed pulmonary tuberculosis, subcutaneous tuberculous abscess and disseminated calcifications in the spleen] | | | | 1986 | | pubmed | title/abstract | irrelevant |
| 911 | Cervera-Hernandez ME | Lung, Brain, and Spinal Cord Abscesses After a Near-Drowning Episode | | | | 2021 | | pubmed | title/abstract | irrelevant |
| 912 | Baynham JT | Ocular vaccinia with severe restriction of extraocular motility | | | | 2009 | | pubmed | duplicate | duplicate |
| 913 | Li Z | Ultrasound- versus palpation-guided injection of corticosteroid for plantar fasciitis: a meta-analysis | | | | 2014 | | pubmed | title/abstract | irrelevant |
| 914 | Chao AS | Abscess formation in ovarian endometriomas after failure of mifepristone-induced abortion | | | | 2016 | | pubmed | title/abstract | irrelevant |
| 915 | MARTIN FR | EXTERNAL BILIARY FISTULA, WITH PORTAL HYPERTENSION, DUE TO MULTIPLE TUBERCULOUS ABSCESSES OF THE LIVER | | | | 1965 | | pubmed | title/abstract | irrelevant |
| 916 | Shi ZG | [Bacterial translocation from the gastrointestinal tract and endogenous infection induced by immunosuppression after burn] | | | | 1990 | | pubmed | title/abstract | irrelevant |
| 917 | Felder JB | The safety of corticosteroid therapy in Crohn's disease with an abdominal mass | | | | 1991 | | pubmed | title/abstract | irrelevant |
| 918 | Ayabe R | Adalimumab as steroid-sparing treatment of inflammatory-stage thyroid eye disease | | | | 2014 | | pubmed | title/abstract | irrelevant |
| 919 | Karls SL | Effectiveness of Corticosteroid Injections in the Treatment of Plantar Fasciitis | | | | 2016 | | pubmed | title/abstract | irrelevant |
| 920 | Zhang N | Multiple brain abscesses due to Listeria monocytogenes infection in a patient with systemic lupus erythematosus: A case report and literature review | | | | 2021 | | pubmed | title/abstract | irrelevant |
| 921 | Neto AA da S | [Sensitivity of microrganisms of root canals of human teeth to corticosteroid-antibiotic-fungicide combinations] | | | | 1975 | | pubmed | title/abstract | irrelevant |
| 922 | Hanchanale P | Nocardia liver abscess post liver transplantation-A rare presentation | | | | 2017 | | pubmed | title/abstract | irrelevant |
| 923 | Akram MR | Comparison of mean pain score of oral non-steroidal anti-inflammatory agents and locally injectable steroid for the treatment of plantar fasciitis | | | | 2022 | | pubmed | title/abstract | irrelevant |
| 924 | Lermi N | What predicts the recurrence in ıdiopathic granulomatous mastitis? | | | | 2023 | | pubmed | title/abstract | irrelevant |
| 925 | Gurun E | Evaluation of the effectiveness of ultrasound-guided corticosteroid injection treatment in plantar fasciitis using shear wave elastography | | | | 2025 | | pubmed | title/abstract | irrelevant |
| 926 | GREEN MA | CRITICAL EVALUATION OF EMULSION THERAPY IN ALLERGY | | | | 1964 | | pubmed | title/abstract | irrelevant |
| 927 | BONFILS S | [Cortisone-cocarboxylase antagonism in regard to induced abscess in the rat] | | | | 1954 | | pubmed | title/abstract | irrelevant |
| 928 | Annabel M | [Treatment of 2 cases of kerions with griseofulvin and oral steroids] | | | | 2009 | | pubmed | title/abstract | irrelevant |
| 929 | Cunningham DS | Spontaneous vulvar necrotizing fasciitis in Cushing's syndrome | | | | 1994 | | pubmed | title/abstract | irrelevant |
| 930 | Ewing DE | Clostridium perfringens necrotizing fasciitis with massive hemolysis | | | | 2017 | | pubmed | title/abstract | irrelevant |
| 931 | Spry CJ | The hypereosinophilic syndrome: clinical features, laboratory findings and treatment | | | | 1982 | | pubmed | title/abstract | irrelevant |
| 932 | Edelstein RA | Cholesteryl ester storage disease: a patient with massive splenomegaly and splenic abscess | | | | 1988 | | pubmed | title/abstract | irrelevant |
| 933 | Savasta S | Gradenigo's syndrome with abscess of the petrous apex in pediatric patients: what is the best treatment? | | | | 2019 | | pubmed | title/abstract | irrelevant |
| 934 | Lund WS | A review of 50 cases of intracranial complications from otogenic infection between 1961 and 1977 | | | | 1978 | | pubmed | title/abstract | irrelevant |
| 935 | Frakun VB | [On the functional status of the adrenal glands in patients with abscessing acne] | | | | 1966 | | pubmed | title/abstract | irrelevant |
| 936 | Genc H | Long-term ultrasonographic follow-up of plantar fasciitis patients treated with steroid injection | | | | 2005 | | pubmed | duplicate | duplicate |
| 937 | MCKENZIE CG | PYOGENIC INFECTION OF LIVER SECONDARY TO INFECTION IN THE PORTAL DRAINAGE AREA | | | | 1964 | | pubmed | title/abstract | irrelevant |
| 938 | Krzymiński TJ | [A case of conservatively treated multiple brain abscesses] | | | | 1986 | | pubmed | title/abstract | irrelevant |
| 939 | Podolsky S | Fatal systemic candidiasis following treatment of Addisonian crisis in a juvenile diabetic | | | | 1970 | | pubmed | title/abstract | irrelevant |
| 940 | ALDRETE JS | GAS GANGRENE; A COMPLICATION OF ELECTIVE ABDOMINAL SURGERY | | | | 1965 | | pubmed | title/abstract | irrelevant |
| 941 | BERDJIS CC | Cortisone and irradiation. II. Pulmonary necrosis and blood vessel impairment in irradiated cortisone-treated rat lung | | | | 1960 | | pubmed | title/abstract | irrelevant |
| 942 | Kent S | Re: Evidence regarding corticosteroid use in deep cervicofacial infections of odontogenic origin | | | | 2020 | | pubmed | title/abstract | comment |
| 943 | Ukai Y | Case of muscle abscess due to disseminated nocardiosis in a patient with autoimmune hemolytic anemia, and review of the published work | | | | 2012 | | pubmed | title/abstract | irrelevant |
| 944 | Franklin A | Packing of abscess cavities | | | | 1973 | | pubmed | title/abstract | irrelevant |
| 945 | WHEATE HW | TWO UNUSUAL CASES OF NERVE ABSCESS | | | | 1964 | | pubmed | title/abstract | irrelevant |
| 946 | Lee TG | Intralesional autologous blood injection compared to corticosteroid injection for treatment of chronic plantar fasciitis. A prospective, randomized, controlled trial | | | | 2007 | | pubmed | title/abstract | irrelevant |
| 947 | Moskopp D | [Septic brain abscess following closed craniocerebral trauma with steroid therapy] | | | | 1985 | | pubmed | title/abstract | irrelevant |
| 948 | Gürçay E | Ultrasound-Guided Plantar Fascia Injections: Where Are You Putting the Gas or Steroids? | | | | 2019 | | pubmed | title/abstract | irrelevant |
| 949 | Corder A | Steroids, non-steroidal anti-inflammatory drugs, and serious septic complications of diverticular disease | | | | 1987 | | pubmed | title/abstract | irrelevant |
| 950 | Frater C | Bone scintigraphy predicts outcome of steroid injection for plantar fasciitis | | | | 2006 | | pubmed | title/abstract | irrelevant |
| 951 | Babaei-Ghazani A | Reply to the Letter to the Editor: Ultrasound-Guided Plantar Fascia Injections: (Where) Are You Putting the Gas or Steroids? | | | | 2019 | | pubmed | title/abstract | irrelevant |
| 952 | DOWNS JW | Surgical complications resulting from ACTH and cortisone medication | | | | 1955 | | pubmed | title/abstract | irrelevant |
| 953 | Tonelli G | Acute toxicity of corticoids in the mouse | | | | 1966 | | pubmed | title/abstract | irrelevant |
| 954 | Henkin RE | Selected topics in intra-abdominal imaging via nuclear medicine techniques | | | | 1979 | | pubmed | title/abstract | irrelevant |
| 955 | Palomar Asenjo V | [Peritonsillar infection. Out-patient management] | | | | 2006 | | pubmed | title/abstract | irrelevant |
| 956 | Lisbona R | Aspergillomatous abscesses of the brain and thyroid | | | | 1973 | | pubmed | title/abstract | irrelevant |
| 957 | Dispenza C | Frontal sinus osteoma complicated by palpebral abscess: case report | | | | 2004 | | pubmed | title/abstract | irrelevant |
| 958 | Gonzalez GA | A ring hypopyon in a patient with meningococcal endophthalmitis: a case report and review of the literature | | | | 1998 | | pubmed | title/abstract | irrelevant |
| 959 | Tarayre JP | Comparison of the cutaneous/systemic antiinflammatory activity ratios for desonide and hydrocortisone in various experimental models | | | | 1988 | | pubmed | title/abstract | irrelevant |
| 960 | Campbell IW | Non-fatal ketoacidosis in a 94-year-old diabetic patient | | | | 1974 | | pubmed | title/abstract | irrelevant |
| 961 | Martino A | [Favorable endobronchial treatment of blocked suppurative bronchopneumopathies] | | | | 1968 | | pubmed | title/abstract | irrelevant |
| 962 | Peart JM | Sporotrichoid fluctuant nodules | | | | 2016 | | pubmed | title/abstract | irrelevant |
| 963 | Jeannet G | [Severe infectious syndromes after tooth extractions. A propos of 3 cases] | | | | 1968 | | pubmed | title/abstract | irrelevant |
| 964 | Cuna GR | High dose medroxyprogesterone acetate (MPA) treatment in metastatic carcinoma of the breast: a dose-response evaluation | | | | 1978 | | pubmed | title/abstract | irrelevant |
| 965 | HUGONOT R | [Abscessed hemorrhagic varicelloid pneumopathy. Role of corticotherapy] | | | | 1959 | | pubmed | title/abstract | irrelevant |
| 966 | Yuan L | Optic nerve aspergillosis | | | | 2015 | | pubmed | title/abstract | irrelevant |
| 967 | Hakan T | Bacterial brain abscesses: an evaluation of 96 cases | | | | 2006 | | pubmed | title/abstract | irrelevant |
| 968 | Curone M | Fatal Aspergillus brain abscess in immunocompetent patient | | | | 2009 | | pubmed | title/abstract | irrelevant |
| 969 | Huskisson EC | Severe, unusual, and recurrent infections in rheumatoid arthritis | | | | 1972 | | pubmed | title/abstract | irrelevant |
| 970 | Alkhatib N | Platelet-Rich Plasma Versus Corticosteroids in the Treatment of Chronic Plantar Fasciitis: A Systematic Review and Meta-analysis of Prospective Comparative Studies | | | | 2020 | | pubmed | title/abstract | irrelevant |
| 971 | Zhang H | Comment on Singh et al.:A systematic review and meta-analysis of platelet-rich plasma versus corticosteroid injections for plantar fasciopathy | | | | 2018 | | pubmed | title/abstract | irrelevant |
| 972 | Nagamoto E | Case of Paecilomyces lilacinus infection occurring in necrotizing fasciitis-associated skin ulcers on the face and surrounding a tracheotomy stoma | | | | 2014 | | pubmed | title/abstract | irrelevant |
| 973 | Heisel NJ | In plantar fasciitis, corticosteroid injections and placebo do not differ for reducing pain at ≤ 12 weeks | | | | 2020 | | pubmed | title/abstract | irrelevant |
| 974 | Adrian RM | Perifolliculitis capitis: successful control with alternate-day corticosteroids | | | | 1980 | | pubmed | title/abstract | irrelevant |
| 975 | Chávez-Rimache L | Evidence regarding use of corticosteroids in deep cervicofacial infections of odontogenic origin | | | | 2020 | | pubmed | title/abstract | comment |
| 976 | Iborra-Marcos Á | Intratissue Percutaneous Electrolysis vs Corticosteroid Infiltration for the Treatment of Plantar Fasciosis | | | | 2018 | | pubmed | title/abstract | irrelevant |
| 977 | Faulk CT | Phaeohyphomycosis and Mycobacterium fortuitum abscesses in a patient receiving corticosteroids for sarcoidosis | | | | 1995 | | pubmed | title/abstract | irrelevant |
| 978 | Sonneville R | Neurologic outcomes and adjunctive steroids in HIV patients with severe cerebral toxoplasmosis | | | | 2012 | | pubmed | title/abstract | irrelevant |
| 979 | Schwartz ID | Pituitary abscess: an unusual presentation of "aseptic meningitis" | | | | 1995 | | pubmed | title/abstract | irrelevant |
| 980 | GOLDSTEIN S | A THREE-STAGE SEQUENTIAL SCREENING PROGRAM FOR THE DETECTION OF ANTI-INFLAMMATORY AGENTS USING A CARRAGEENIN-INDUCED ABSCESS | | | | 1963 | | pubmed | title/abstract | irrelevant |
| 981 | Lee HS | Predictors of unprovoked seizures in surgically treated pyogenic brain abscess: Does perioperative adjunctive use of steroids has any protective effect? | | | | 2018 | | pubmed | title/abstract | irrelevant |
| 982 | Shen K | Sweet syndrome secondary to myelodysplastic syndrome mimicking necrotizing fasciitis | | | | 2017 | | pubmed | title/abstract | irrelevant |
| 983 | el-Hennawy M | Hazards of cortisone therapy in hepatic amoebiasis | | | | 1978 | | pubmed | title/abstract | irrelevant |
| 984 | Mofredj A | Article: Paradoxical enlargement of tuberculous brain abscess during drug treatment: a case report (S K Ng, et al) | | | | 2002 | | pubmed | title/abstract | irrelevant |
| 985 | Forno LS | Allescheria boydii infection of the brain | | | | 1972 | | pubmed | title/abstract | irrelevant |
| 986 | Yampolsky C | Fungal cerebral abscess in a diabetic patient successfully treated with surgery followed by prolonged antifungal therapy | | | | 2010 | | pubmed | title/abstract | irrelevant |
| 987 | Salvatore T | Cushing syndrome and giant sterile abscess induced by self intramuscular injection of supra-therapeutic doses of triamcinolone | | | | 2013 | | pubmed | title/abstract | irrelevant |
| 988 | Guimarães F | Predictors of Relapse After Corticosteroid Injection for the Treatment of Plantar Fasciitis | | | | 2022 | | pubmed | title/abstract | irrelevant |
| 989 | Corrigan AB | Intraspinal corticosteroid injections | | | | 1982 | | pubmed | title/abstract | irrelevant |
| 990 | Hammill HA | A rat model of unilateral utero-tubo-ovarian abscess | | | | 1984 | | pubmed | title/abstract | irrelevant |
| 991 | Yamanouchi J | [Acquired hemophilia complicated with multiple muscle abscess by Nocardia] | | | | 2009 | | pubmed | title/abstract | irrelevant |
| 992 | Bossi G | Cerebral Toxocariasis as a Cause of Epilepsy: A Pediatric Case | | | | 2021 | | pubmed | title/abstract | irrelevant |
| 993 | Biagi F | [Relation between cholesterol and hepatic amebiasis in man] | | | | 1965 | | pubmed | title/abstract | irrelevant |
| 994 | ROMANI JD | [Study of the pro-inflammatory action of aldosterone (electrocortin) on the fixation abscess caused by terebenthine in the rat] | | | | 1955 | | pubmed | title/abstract | irrelevant |
| 995 | ROMANI JD | [The effect of anti-inflammatory corticoids on the fibroblasts and on the mucopolysaccharides of the fixation abscess in the rat] | | | | 1954 | | pubmed | title/abstract | irrelevant |
| 996 | Tunbridge WM | Unusual Munchausen's syndrome | | | | 1969 | | pubmed | title/abstract | irrelevant |
| 997 | Sedney CL | An unusual form of listerial CNS infection | | | | 2012 | | pubmed | title/abstract | irrelevant |
| 998 | Bloom DC | Head and neck manifestation of mycobacterium avium complex disease as a consequence of return of immunocompetency in AIDS | | | | 2001 | | pubmed | title/abstract | irrelevant |
| 999 | Vadillo M | Pyoderma gangrenosum with liver, spleen and bone involvement in a patient with chronic myelomonocytic leukaemia | | | | 1999 | | pubmed | title/abstract | irrelevant |
| 1000 | Muranjan SN | Tubercular laryngeal abscess | | | | 2001 | | pubmed | title/abstract | irrelevant |
| 1001 | Rath S | Orbital cysticercosis: clinical manifestations, diagnosis, management, and outcome | | | | 2010 | | pubmed | title/abstract | irrelevant |
| 1002 | De Córdoba JL | Spinal injections: getting hold of the wrong end of the stick | | | | 2006 | | pubmed | title/abstract | irrelevant |
| 1003 | Gorback MS | Primum non nocere | | | | 2006 | | pubmed | title/abstract | irrelevant |
| 1004 | Knight JW | Epidural abscess following epidural steroid and local anaesthetic injection | | | | 1997 | | pubmed | title/abstract | irrelevant |
| 1005 | Skoutelis A | Serious complications of tuberculous epididymitis | | | | 2000 | | pubmed | title/abstract | irrelevant |
| 1006 | Tzaribachev N | Infliximab effective in steroid-dependent juvenile eosinophilic fasciitis | | | | 2008 | | pubmed | title/abstract | irrelevant |
| 1007 | Brown RS | Vitamins and the treatment of oral and dental diseases | | | | 2010 | | pubmed | title/abstract | irrelevant |
| 1008 | Moragrega R | [Non-surgical treatment of a cerebral abscess] | | | | 1984 | | pubmed | title/abstract | irrelevant |
| 1009 | Vilalta Castán J | [Cerebral abscess treated with antibiotics and corticotherapy] | | | | 1983 | | pubmed | title/abstract | irrelevant |
| 1010 | KOHLER V | [The formation of aseptic corneal abscesses plotted as a function of monoiodoacetic acid and desoxycorticosterone glucoside] | | | | 1951 | | pubmed | title/abstract | irrelevant |
| 1011 | Valleix B | [A parotitis as primary infection of Lemierre's syndrome] | | | | 2011 | | pubmed | title/abstract | irrelevant |
| 1012 | Sabatier J | Contribution of in vivo 1H spectroscopy to the diagnosis of deep-seated brain abscess | | | | 1999 | | pubmed | title/abstract | irrelevant |
| 1013 | Srikumar T | Aspergillus Terreus Brain Abscess Complicated by Tension Pneumocephalus in a Patient with Angiosarcoma | | | | 2017 | | pubmed | title/abstract | irrelevant |
| 1014 | Dubeau F | Brain abscess due to Petriellidium boydii | | | | 1984 | | pubmed | title/abstract | irrelevant |
| 1015 | Stauffer UG | [Postoperative progressive gangrene] | | | | 1970 | | pubmed | title/abstract | irrelevant |
| 1016 | Bejanga BI | Unusual complications of myositis tropicans | | | | 1979 | | pubmed | title/abstract | irrelevant |
| 1017 | Alvarez-Cordero R | [Intensive care unit for the treatment of severe invasive amebiasis] | | | | 1974 | | pubmed | title/abstract | irrelevant |
| 1018 | MARCHAL G | [PULMONARY MYCOTIC COMPLICATIONS IN BLOOD DISEASES TREATED BY CORTISONE. (APROPOS OF 4 CASES)] | | | | 1963 | | pubmed | title/abstract | irrelevant |
| 1019 | Agrawal A | Effect of systemic corticosteroid therapy on risk for intra-abdominal or pelvic abscess in non-operated Crohn's disease | | | | 2005 | | pubmed | title/abstract | irrelevant |
| 1020 | FRANCHIMONT P | [GLOMERULOTROPHIN AND EXPERIMENTAL INFLAMMATION] | | | | 1964 | | pubmed | title/abstract | irrelevant |
| 1021 | Boom WH | Successful treatment of multiple brain abscesses with antibiotics alone | | | | 1985 | | pubmed | title/abstract | irrelevant |
| 1022 | Riordan M | Investigation and treatment of facial paralysis | | | | 2001 | | pubmed | title/abstract | irrelevant |
| 1023 | Bay A | Multiple brain abscesses in a child with autoimmune hemolytic anemia | | | | 2007 | | pubmed | title/abstract | irrelevant |
| 1024 | Ishizawa J | [Recurrent cellulitis due to Helicobacter cinaedi after chemotherapy for malignant lymphoma] | | | | 2012 | | pubmed | title/abstract | irrelevant |
| 1025 | Furr PM | The susceptibility of germ-free, oestradiol-treated, mice to Mycoplasma hominis | | | | 1989 | | pubmed | title/abstract | irrelevant |
| 1026 | Leelarasamee A | Disseminated nocardiosis after pulmonary collapse: a case report | | | | 1977 | | pubmed | title/abstract | irrelevant |
| 1027 | Thorne E | Subcutaneous Trichophyton rubrum abscesses. A case report | | | | 1971 | | pubmed | title/abstract | irrelevant |
| 1028 | Mustafin DG | [Acute lung destruction after corticosteroid therapy of bronchial asthma] | | | | 1978 | | pubmed | title/abstract | irrelevant |
| 1029 | Sayabovorn N | Cryptococcal fungemia and Mycobacterium haemophilum cellulitis in a patient receiving ruxolitinib: a case report and literature review | | | | 2021 | | pubmed | title/abstract | irrelevant |
| 1030 | Friis-Møller A | Treatment of Legionella lung abscess in a renal transplant recipient with erythromycin and fusidic acid | | | | 1985 | | pubmed | title/abstract | irrelevant |
| 1031 | McIntosh CS | Maintenance of silastic-teflon shunts for intermitten haemodialysis | | | | 1969 | | pubmed | title/abstract | irrelevant |
| 1032 | KALLIOMAEKI JL | NORANDROSTENOLONE DECANOATE AS A CARDIAC ANABOLIZER STUDIED BY MEANS OF ELECTROCARDIOGRAPHIC CHANGES | | | | 1963 | | pubmed | title/abstract | irrelevant |
| 1033 | ARDAGH JW | IDIOPATHIC RETROPERITONEAL FIBROSIS | | | | 1964 | | pubmed | title/abstract | irrelevant |
| 1034 | Kurtzman DJB | Image Gallery: Dissecting cellulitis of the scalp following anabolic steroid use | | | | 2017 | | pubmed | title/abstract | irrelevant |
| 1035 | JENSEN K | FULMINATING STAPHYLOCOCCAL INFECTIONS TREATED WITH FUCIDIN AND PENICILLIN OR SEMISYNTHETIC PENICILLIN | | | | 1964 | | pubmed | title/abstract | irrelevant |
| 1036 | Fárková H | [Idiopathic hypoparathyroidism] | | | | 1971 | | pubmed | title/abstract | irrelevant |
| 1037 | Marzano AV | Neutrophilic dermatoses and inflammatory bowel diseases | | | | 2013 | | pubmed | title/abstract | irrelevant |
| 1038 | Wirthlin MR | Chemical treatment of diseased root surfaces in vitro | | | | 1981 | | pubmed | title/abstract | irrelevant |
| 1039 | Campbell BG | False-negative single-photon emission CT in AIDS lymphoma: lack of effect of steroids | | | | 1996 | | pubmed | title/abstract | irrelevant |
| 1040 | Ohtake T | Biloma during steroid therapy for minimal change nephrotic syndrome | | | | 1993 | | pubmed | title/abstract | irrelevant |
| 1041 | Heine S | Legionellosis must be kept in mind in case of pneumonia with lung abscesses in children receiving therapeutic steroids | | | | 2011 | | pubmed | title/abstract | irrelevant |
| 1042 | Kitano T | Successful transcatheter arterial antimicrobial and steroid therapy for refractory liver abscess in chronic granulomatous disease: A case report and review of literature | | | | 2018 | | pubmed | title/abstract | irrelevant |
| 1043 | Malcomson KG | Wegener's giant cell granulomatosis treated with corticosteroids | | | | 1966 | | pubmed | title/abstract | irrelevant |
| 1044 | Estefan-Estefan A | [The cortico-steroids in endodontics] | | | | 1968 | | pubmed | title/abstract | unclear |
| 1045 | Gable AD | Suppurative inflammation with microabscess and pseudocyst formation is a characteristic histologic manifestation of cutaneous infections with rapid-growing Mycobacterium species | | | | 2008 | | pubmed | title/abstract | irrelevant |
| 1046 | Chen KJ | Prevention of Evisceration or Enucleation in Endogenous Bacterial Panophthalmitis with No Light Perception and Scleral Abscess | | | | 2017 | | pubmed | title/abstract | irrelevant |
| 1047 | Desai N | Tuberculous brain abscess in an adolescent with complex congenital cyanotic heart disease | | | | 2013 | | pubmed | title/abstract | irrelevant |
| 1048 | Aguilera V | [Sciatica secondary to a presacral abscess as the first manifestation of Crohn's disease] | | | | 2002 | | pubmed | title/abstract | irrelevant |
| 1049 | Yucel U | Full-length silicone insoles versus ultrasound-guided corticosteroid injection in the management of plantar fasciitis: a randomized clinical trial | | | | 2013 | | pubmed | title/abstract | irrelevant |
| 1050 | Guetgemann A | Unclear fever 7 weeks after renal transplantation in a 56-year-old patient | | | | 2006 | | pubmed | title/abstract | irrelevant |
| 1051 | Stuiver PC | Corticosteroids and liver amoebiasis | | | | 1978 | | pubmed | title/abstract | irrelevant |
| 1052 | Toy S | Corticosteroid, Platelet-Rich Plasma, and Ozone Injections for Sinus Tarsi Syndrome | | | | 2023 | | pubmed | duplicate | duplicate |
| 1053 | Lee EJ | Outgrowing skin involvement in malakoplakia after kidney transplantation: A case report | | | | 2022 | | pubmed | title/abstract | irrelevant |
| 1054 | MAGGI CA | [ANABOLIC DRUGS IN THE CORRECTION OF A PROTEIN-DEFICIENT NUTRITIONAL STATE. BIOPHARMACOLOGICAL AND CLINICAL CONSIDERATIONS ON THE USE OF 2-ALPHA,17-ALPHA-DIMETHYL-5-ALPHA-ANDROSTAN-17-BETA-OL-3,3'-AZINE (DIMETHAZINE)] | | | | 1964 | | pubmed | title/abstract | irrelevant |
| 1055 | Holton CP | Clinical study of daunomycin and prednisone for induction of remission in children with advanced leukemia | | | | 1969 | | pubmed | title/abstract | irrelevant |
| 1056 | Smirnov VE | [Diagnosis of postero-inferior subdiaphragmatic abscess using liquid crystal thermography] | | | | 1990 | | pubmed | title/abstract | irrelevant |
| 1057 | Gustincic D | [Treatment of root canal and periapical abscess with the drug dexamethason-neomycin] | | | | 1970 | | pubmed | title/abstract | irrelevant |
| 1058 | BARTHE JJ | [TERRAMYCIN OINTMENT WITH HYDROCORTISONE AND TERRAMYCIN NEBULIZER WITH HYDROCORTISONE AND GENERAL MEDICINE] | | | | 1964 | | pubmed | title/abstract | irrelevant |
| 1059 | HURLEY JV | The effects of amino-acetonitrile and cortisone on the healing of turpentine-induced abscesses in the rat | | | | 1958 | | pubmed | title/abstract | irrelevant |
| 1060 | Milioni C | Pharmacological study in vivo of the new topical anti-inflammatory steroid 21-thiol-9 alpha-fluoro-11 beta,17 alpha-dihydroxy-16 alpha-methyl-3,20-dione-21-acetylamino cysteine | | | | 1991 | | pubmed | title/abstract | irrelevant |
| 1061 | Goucke CR | Extradural abscess following local anaesthetic and steroid injection for chronic low back pain | | | | 1990 | | pubmed | title/abstract | irrelevant |
| 1062 | Reiss G | [Brain abscess as an endocranial complication of otologic diseases] | | | | 1992 | | pubmed | title/abstract | irrelevant |
| 1063 | Richards J | Microbiology, chemotherapy and mortality of brain abscess in Newcastle-upon-Tyne between 1979 and 1988 | | | | 1990 | | pubmed | title/abstract | irrelevant |
| 1064 | Hinson KR | Answer to July 2018 Photo Quiz | | | | 2018 | | pubmed | title/abstract | irrelevant |
| 1065 | Kirkman MA | Neurological picture. Multiple intracranial abscesses due to Streptococcus anginosus in a previously well individual | | | | 2012 | | pubmed | title/abstract | irrelevant |
| 1066 | Er-Lukowiak M | Testosterone affects type I/type II interferon response of neutrophils during hepatic amebiasis | | | | 2023 | | pubmed | title/abstract | irrelevant |
| 1067 | Slavei K | [Flesh-eating bacteria infection of an immunocompromised patient] | | | | 2001 | | pubmed | title/abstract | irrelevant |
| 1068 | Gupta D | A complex orbit | | | | 2008 | | pubmed | title/abstract | irrelevant |
| 1069 | Fasano CJ | Bilateral peritonsillar abscesses: not your usual sore throat | | | | 2005 | | pubmed | title/abstract | irrelevant |
| 1070 | Page C | Immediate tonsillectomy: indications for use as first-line surgical management of peritonsillar abscess (quinsy) and parapharyngeal abscess | | | | 2010 | | pubmed | title/abstract | irrelevant |
| 1071 | Buxhofer V | Successful treatment of invasive mould infection affecting lung and brain in an adult suffering from acute leukaemia | | | | 2001 | | pubmed | title/abstract | irrelevant |
| 1072 | Tkachenko SS | [Reasons for complications during hydrocortisone treatments of various diseases of the extremities] | | | | 1972 | | pubmed | title/abstract | irrelevant |
| 1073 | Dehority W | Brain abscess caused by Streptococcus pyogenes in a previously healthy child | | | | 2006 | | pubmed | title/abstract | irrelevant |
| 1074 | Lifshitz T | Rhodotorula rubra keratitis and melting after repeated penetrating keratoplasty | | | | 2005 | | pubmed | title/abstract | irrelevant |
| 1075 | Bhama JK | Primary anorectal lymphoma presenting as a perianal abscess in an HIV-positive male | | | | 2002 | | pubmed | title/abstract | irrelevant |
| 1076 | Blanco J | [Cerebral abscess caused by Rhodococcus equi in an immunocompetent patient] | | | | 1998 | | pubmed | title/abstract | irrelevant |
| 1077 | Roda RH | Rhodococcus equi pulmonary-central nervous system syndrome: brain abscess in a patient on high-dose steroids--a case report and review of the literature | | | | 2009 | | pubmed | title/abstract | irrelevant |
| 1078 | Carvalho P | [Cutaneous aseptic abscesses, manifestations of neutrophilic diseases] | | | | 2001 | | pubmed | title/abstract | irrelevant |
| 1079 | Sowter MC | Delayed presentation of an extradural abscess complicating thoracic extradural analgesia | | | | 1992 | | pubmed | title/abstract | irrelevant |
| 1080 | Talyshinskii AM | [Effect of hydrocortisone on changes in brain tissue in experimental brain abscess and leptomeningitis] | | | | 1986 | | pubmed | title/abstract | irrelevant |
| 1081 | Rich JD | Abscess related to anabolic-androgenic steroid injection | | | | 1999 | | pubmed | title/abstract | irrelevant |
| 1082 | Baron EJ | Actinomycotic pulmonary abscess in an immunosuppressed patient | | | | 1979 | | pubmed | title/abstract | irrelevant |
| 1083 | Salloum S | Septic cavernous sinus thrombosis in a paediatric patient with undiagnosed thrombophilia | | | | 2019 | | pubmed | title/abstract | irrelevant |
| 1084 | Madsen SM | Magnetic resonance imaging of Crohn disease: early recognition of treatment response and relapse | | | | 1997 | | pubmed | title/abstract | irrelevant |
| 1085 | Mauser HW | Conservative and surgical management of focal cerebral infection | | | | 1985 | | pubmed | title/abstract | irrelevant |
| 1086 | Neuwelt EA | Cerebrovascular permeability and delivery of gentamicin to normal brain and experimental brain abscess in rats | | | | 1984 | | pubmed | title/abstract | irrelevant |
| 1087 | BAHR D | Waterhouse-Friderichsen syndrome: report of a case with recovery | | | | 1955 | | pubmed | title/abstract | irrelevant |
| 1088 | Rosazza A | Nonsurgical treatment of interhemispheric subdural empyemas | | | | 1979 | | pubmed | title/abstract | irrelevant |
| 1089 | Meister H | [Skin manifestations in monocytic leukemia] | | | | 1971 | | pubmed | title/abstract | irrelevant |
| 1090 | Yamazaki-Nakashimada MA | Corticosteroid therapy for refractory infections in chronic granulomatous disease: case reports and review of the literature | | | | 2006 | | pubmed | title/abstract | irrelevant |
| 1091 | Dubos F | Bacterial skin infections in children hospitalized with varicella: a possible negative impact of non-steroidal anti-inflammatory drugs? | | | | 2008 | | pubmed | title/abstract | irrelevant |
| 1092 | Cooper K | Intralesional corticosteroids and diathermy ablation for the management of anogenital granulomatosis: a retrospective cohort study | | | | 2022 | | pubmed | title/abstract | irrelevant |
| 1093 | Ancona S | Dose escalation of adalimumab as a strategy to overcome anti-drug antibodies: A case report of infantile-onset inflammatory bowel disease | | | | 2023 | | pubmed | title/abstract | irrelevant |
| 1094 | Jokinen MA | Pulp capping with corticoid-chemotherapeutic plus calciumhydroxide | | | | 1970 | | pubmed | title/abstract | irrelevant |
| 1095 | Kaya A | A case of recurrent sterile abscesses following tetanus-diphtheria vaccination treated with corticosteroids | | | | 2021 | | pubmed | title/abstract | irrelevant |
| 1096 | Sharma R | Effect of platelet-rich plasma versus steroid injection in plantar fasciitis: a randomized clinical trial | | | | 2023 | | pubmed | title/abstract | irrelevant |
| 1097 | Sellman JR | Plantar fascia rupture associated with corticosteroid injection | | | | 1994 | | pubmed | title/abstract | irrelevant |
| 1098 | Bertozzi G | Immunodeficiency as a side effect of anabolic androgenic steroid abuse: a case of necrotizing myofasciitis | | | | 2019 | | pubmed | title/abstract | irrelevant |
| 1099 | Uygur E | Preliminary Report on the Role of Dry Needling Versus Corticosteroid Injection, an Effective Treatment Method for Plantar Fasciitis: A Randomized Controlled Trial | | | | 2019 | | pubmed | duplicate | duplicate |
| 1100 | Karandashov VI | [Kallikrein-kinin system and the blood cortisol level of patients with maxillofacial phlegmons] | | | | 1983 | | pubmed | title/abstract | irrelevant |
| 1101 | Oshinowo AG | Tuberculous cerebellar abscess | | | | 1998 | | pubmed | title/abstract | irrelevant |
| 1102 | Sehić M | [Experience in gangrene therapy with Ledermix paste] | | | | 1978 | | pubmed | title/abstract | irrelevant |
| 1103 | SALIBA A | Treatment of mycotic infections: hydrocortisone in the control of amphotericin-B toxicity | | | | 1962 | | pubmed | title/abstract | irrelevant |
| 1104 | Talyshinskiĭ AM | [Effect of hydrocortisone on changes in the hypothalamo-hypophyseal system in experimental brain abscess] | | | | 1987 | | pubmed | title/abstract | irrelevant |
| 1105 | LINCKE HO | [ON THE TREATMENT OF SUPPURATIVE BACTERIAL MENINGITIS] | | | | 1964 | | pubmed | title/abstract | irrelevant |
| 1106 | FAVEZ G | [INDICATIONS FOR CORTISOL IN PNEUMOLOGY] | | | | 1964 | | pubmed | title/abstract | irrelevant |
| 1107 | Loftis L | Acute infectious upper airway obstructions in children | | | | 2006 | | pubmed | title/abstract | irrelevant |
| 1108 | Morris T | Letter: Packing of abscess cavities | | | | 1973 | | pubmed | duplicate | duplicate |
| 1109 | Iwahashi C | Orbital abscess caused by Exophiala dermatitidis following posterior subtenon injection of triamcinolone acetonide: a case report and a review of literature related to Exophiala eye infections | | | | 2020 | | pubmed | title/abstract | irrelevant |
| 1110 | Burman JH | The fate of ileorectal anastomosis in Crohn's disease | | | | 1971 | | pubmed | title/abstract | irrelevant |
| 1111 | Goenka PK | Corticosteroids in the Treatment of Pediatric Retropharyngeal and Parapharyngeal Abscesses | | | | 2021 | | pubmed | title/abstract | ineligible population |
| 1112 | Hohmann E | Platelet-Rich Plasma Versus Corticosteroids for the Treatment of Plantar Fasciitis: A Systematic Review and Meta-analysis | | | | 2021 | | pubmed | title/abstract | irrelevant |
| 1113 | Saglam N | Intratendinous septic abscess of the Achilles tendon after local steroid injection | | | | 2009 | | pubmed | title/abstract | irrelevant |
| 1114 | Chen YJ | Autologous Blood-Derived Products Compared With Corticosteroids for Treatment of Plantar Fasciopathy: A Systematic Review and Meta-Analysis | | | | 2019 | | pubmed | title/abstract | irrelevant |
| 1115 | Gupta A | Epididymal abscess in renal transplant: uncommon presentation of Klebsiella septicemia | | | | 2010 | | pubmed | title/abstract | irrelevant |
| 1116 | Ahmed AR | Pemphigus vegetans. Neumann type and Hallopeau type | | | | 1984 | | pubmed | title/abstract | irrelevant |
| 1117 | Sobocinski PZ | Effect of adrenalectomy on cadmium- and turpentine-induced hepatic synthesis of metallothionein and alpha 2-macrofetoprotein in the rat | | | | 1981 | | pubmed | title/abstract | irrelevant |
| 1118 | Zuo A | Platelet-Rich Plasma Versus Corticosteroids in the Treatment of Plantar Fasciitis: A Systematic Review and Meta-analysis | | | | 2025 | | pubmed | title/abstract | irrelevant |
| 1119 | Lindholm J | Intrasellar or pituitary abscess | | | | 1973 | | pubmed | title/abstract | irrelevant |
| 1120 | Kumetz EA | Epidermolysis Bullosa Acquisita: A Case Report | | | | 2020 | | pubmed | title/abstract | irrelevant |
| 1121 | Fujikawa T | Spinal cord abscess and inflammatory bowel disease | | | | 2015 | | pubmed | title/abstract | irrelevant |
| 1122 | Garner J | Ring lesions in the brain: a harmless commensal? | | | | 2013 | | pubmed | title/abstract | irrelevant |
| 1123 | Montaruli E | Sterile hepatic abscess secondary to administration of parenteral fluids via an umbilical venous catheter in a premature baby | | | | 2011 | | pubmed | title/abstract | irrelevant |
| 1124 | Katz U | Chronic idiopathic granulomatous mastitis | | | | 2007 | | pubmed | title/abstract | irrelevant |
| 1125 | Tsukaguchi K | [A case of chronic mucocutaneous candidiasis (CMCC) with repeated pulmonary abscess and sepsis] | | | | 1987 | | pubmed | title/abstract | irrelevant |
| 1126 | Ostrov MR | Dramatic resolution of chronic urticaria | | | | 1995 | | pubmed | title/abstract | irrelevant |
| 1127 | Firsching R | [Brain abscess: surgical or conservative treatment?] | | | | 1986 | | pubmed | title/abstract | irrelevant |
| 1128 | Krivoruchko IA | [The role of endothelin-1 in the pathogenesis of septic shock in the abdominal sepsis] | | | | 2004 | | pubmed | title/abstract | irrelevant |
| 1129 | Hanami Y | Association of pyoderma gangrenosum, erythema nodosum and aseptic liver abscess without significant underlying disease | | | | 2019 | | pubmed | title/abstract | irrelevant |
| 1130 | Ingwer I | Aspergillus fumigatus epidural abscess in a renal transplant recipient | | | | 1978 | | pubmed | title/abstract | irrelevant |
| 1131 | Razon RV | Retropharyngeal calcific tendonitis: report of two cases | | | | 2009 | | pubmed | title/abstract | irrelevant |
| 1132 | Opanasenko NS | [Experience in the use of lymphatic infusion preparations in multimodal treatment of patients with giant lung abscess] | | | | 1996 | | pubmed | title/abstract | irrelevant |
| 1133 | Flores MS | Hypocholesterolemia in patients with an amebic liver abscess | | | | 2014 | | pubmed | title/abstract | irrelevant |
| 1134 | Presant CA | Disseminated extrapulmonary nocardiosis presenting as a renal abscess | | | | 1970 | | pubmed | title/abstract | irrelevant |
| 1135 | Salazar A | [Splenic abscesses caused by Mycobacterium tuberculosis in AIDS] | | | | 1994 | | pubmed | title/abstract | irrelevant |
| 1136 | Hartmann A | Intracerebral abscess caused by Nocardia otitidiscaviarum in a renal transplant patient--cured by evacuation plus antibiotic therapy | | | | 2000 | | pubmed | title/abstract | irrelevant |
| 1137 | Yamashita J | Brain abscess due to Haemophilus aphrophilus: case report | | | | 1972 | | pubmed | title/abstract | irrelevant |
| 1138 | Vandersmissen G | Cutaneous cryptococcosis in corticosteroid-treated patients without AIDS | | | | 1996 | | pubmed | title/abstract | irrelevant |
| 1139 | Chiang HL | Epidural abscess in an obstetric patient with patient-controlled epidural analgesia--a case report | | | | 2005 | | pubmed | title/abstract | irrelevant |
| 1140 | Yapici F | Which Treatment Method Is Better in the Treatment of Chronic Plantar Fasciitis: Corticosteroid Injection, Extracorporeal Shock Wave Therapy, or Radiofrequency Thermal Lesioning? | | | | 2023 | | pubmed | title/abstract | irrelevant |
| 1141 | Segura F | [Cerebral abscess treated with antibiotics and corticotherapy] | | | | 1984 | | pubmed | title/abstract | irrelevant |
| 1142 | Yamagata H | [Experimental study on the prevention of the cavity formation. (The prevention of cavity formation by staphylococci)] | | | | 1966 | | pubmed | title/abstract | irrelevant |
| 1143 | Betts PR | Oral manifestations of Letterer-Siwe disease | | | | 1972 | | pubmed | title/abstract | irrelevant |
| 1144 | Sakai H | A verrucous lesion on skin grafted after necrotizing fasciitis in a diabetic patient successfully treated with combined topical 5-FU and tacalcitol | | | | 1997 | | pubmed | title/abstract | irrelevant |
| 1145 | Yucel I | Comparison of high-dose extracorporeal shockwave therapy and intralesional corticosteroid injection in the treatment of plantar fasciitis | | | | 2010 | | pubmed | duplicate | duplicate |
| 1146 | Karagaiah P | Update on Hormonal Therapy in Hidradenitis Suppurativa | | | | 2023 | | pubmed | title/abstract | irrelevant |
| 1147 | Rotstein I | Prevalence of periapical abscesses in vitamin D deficient patients | | | | 2021 | | pubmed | title/abstract | irrelevant |
| 1148 | Lynch JC | [Intracerebral abscess: clinical treatment. Experience with a case] | | | | 1985 | | pubmed | title/abstract | irrelevant |
| 1149 | Riel H | Does a corticosteroid injection plus exercise or exercise alone add to the effect of patient advice and a heel cup for patients with plantar fasciopathy? A randomised clinical trial | | | | 2023 | | pubmed | title/abstract | irrelevant |
| 1150 | Briani C | Polyneuropathy, organomegaly, endocrinopathy, M protein, skin changes: not always a POEMS syndrome | | | | 2006 | | pubmed | title/abstract | irrelevant |
| 1151 | Yamamoto T | Risk factors for intra-abdominal sepsis after surgery in Crohn's disease | | | | 2000 | | pubmed | title/abstract | irrelevant |
| 1152 | Cockburn F | Maternal vitamin D intake and mineral metabolism in mothers and their newborn infants | | | | 1980 | | pubmed | title/abstract | irrelevant |
| 1153 | Kuusanmäki P | How to diagnose chronic rejection. A study in porcine intestinal allografts | | | | 1997 | | pubmed | title/abstract | irrelevant |
| 1154 | Gutmann R | [Rare complications of soft tissue infections in the head and neck area: deep neck phlegmona, thrombophlebitis and mediastinitis with pericardial effusion] | | | | 1994 | | pubmed | title/abstract | irrelevant |
| 1155 | Ozbek C | Use of steroids in the treatment of peritonsillar abscess | | | | 2004 | | pubmed | duplicate | duplicate |
| 1156 | Durieux S | [Complications of colonic diverticular disease during rheumatoid polyarthritis: 7 cases] | | | | 1999 | | pubmed | title/abstract | irrelevant |
| 1157 | Yun SJ | Cutaneous abscess by Trichosporon asahii developing on a steroid injection site in a healthy adult | | | | 2006 | | pubmed | title/abstract | irrelevant |
| 1158 | Charavin-Cocuzza M | [Febrile cellulitis surrounding a scar revealing a large immunoblastic B-cell lymphoma] | | | | 2008 | | pubmed | title/abstract | irrelevant |
| 1159 | Fasulo CP | Hand-Schüller-Christian disease. Medical and oral surgical problems involved | | | | 1966 | | pubmed | title/abstract | irrelevant |
| 1160 | Tsikopoulos K | Autologous whole blood or corticosteroid injections for the treatment of epicondylopathy and plantar fasciopathy? A systematic review and meta-analysis of randomized controlled trials | | | | 2016 | | pubmed | title/abstract | irrelevant |
| 1161 | Higuchi K | Comparative studies on a heat-stable cholesterol-binding protein in dental cyst fluid and serum | | | | 1981 | | pubmed | title/abstract | irrelevant |
| 1162 | Rangé H | Chewing capacity and ideal cardiovascular health in adulthood: A cross-sectional analysis of a population-based cohort study | | | | 2020 | | pubmed | title/abstract | irrelevant |
| 1163 | Kovalev MM | [The effect of acute cholecystitis on the function of the adrenal cortex] | | | | 1969 | | pubmed | title/abstract | irrelevant |
| 1164 | Seale JP | An unusual complication of corticosteroid therapy for sarcoidosis | | | | 1977 | | pubmed | title/abstract | irrelevant |
| 1165 | Whittaker GA | Predictors of response to foot orthoses and corticosteroid injection for plantar heel pain | | | | 2020 | | pubmed | duplicate | duplicate |
| 1166 | André M | [Corticosteroid-sensitive aseptic abscess associated with inflammatory bowel disease. An emerging syndrome] | | | | 2001 | | pubmed | title/abstract | irrelevant |
| 1167 | Kune GA | The challenge of severe acute pancreatitis | | | | 1968 | | pubmed | title/abstract | irrelevant |
| 1168 | Ruiz-Roca JA | Pyostomatitis vegetans. Report of two cases and review of the literature | | | | 2005 | | pubmed | title/abstract | irrelevant |
| 1169 | FERKO S | [THE ROLE OF GLYCOCORTICOIDS IN THE TREATMENT OF SEVERE INFECTIOUS OBSTETRICAL AND GYNECOLOGICAL DISEASES] | | | | 1964 | | pubmed | title/abstract | irrelevant |
| 1170 | Feldhoff C | [Peritonitis and infection in children with idiopathic nephrotic syndrome] | | | | 1988 | | pubmed | title/abstract | irrelevant |
| 1171 | Gill NJ | Progesterone-induced amoebic liver abscess in guinea-pigs--a new model | | | | 1983 | | pubmed | title/abstract | irrelevant |
| 1172 | Götz M | [Obstructive airway diseases in children] | | | | 1982 | | pubmed | title/abstract | irrelevant |
| 1173 | Bos HJ | Virulence and toxicity of axenic Entamoeba histolytica | | | | 1977 | | pubmed | title/abstract | irrelevant |
| 1174 | Durst J | [Appendicitis and pregnancy] | | | | 1970 | | pubmed | title/abstract | irrelevant |
| 1175 | Alleva FR | Effect of a single prepubertal injection of testosterone propionate on later reproductive functions of the female golden hamster | | | | 1969 | | pubmed | title/abstract | irrelevant |
| 1176 | Dashkovsky I | Unusual presentation of angiocentric T-cell lymphoma mimicking perianal abscess | | | | 2003 | | pubmed | title/abstract | irrelevant |
| 1177 | García-Pérez A | [Non-dystonic torticollis. A report of a case secondary to retropharyngeal abscess] | | | | 2000 | | pubmed | title/abstract | irrelevant |
| 1178 | Hornof R | Intraperitoneal cholelithiasis after laparoscopic cholecystectomy--behavior of 'lost' concrements and their role in abscess formation | | | | 1996 | | pubmed | title/abstract | irrelevant |
| 1179 | Kyle J | Psoas abscess in Crohn's disease | | | | 1971 | | pubmed | title/abstract | irrelevant |
| 1180 | Strahan RW | Thyroiditis. A classification and review | | | | 1971 | | pubmed | title/abstract | irrelevant |
| 1181 | Gill NJ | Antibody dependent cellular cytotoxicity in experimental intestinal & hepatic amoebiasis | | | | 1988 | | pubmed | title/abstract | irrelevant |
| 1182 | Shimokawaji T | [Pulmonary nocardiosis complicated with multiple brain abscess] | | | | 2005 | | pubmed | title/abstract | irrelevant |
| 1183 | Butler SH | Primum non nocere--first do no harm | | | | 2005 | | pubmed | title/abstract | irrelevant |
| 1184 | Hetessyné Debreczeni L | [Root canal therapy with pyrazolidine] | | | | 1966 | | pubmed | title/abstract | irrelevant |
| 1185 | Carrero JC | Parasiticidal effect of 16alpha-bromoepiandrosterone (EpiBr) in amoebiasis and cysticercosis | | | | 2010 | | pubmed | title/abstract | irrelevant |
| 1186 | Luchshev VI | [Treatment of liver insufficiency of various etiology] | | | | 1978 | | pubmed | title/abstract | irrelevant |
| 1187 | SALAMATINA VV | [ON FAT METABOLISM IN CHRONIC NONSPECIFIC LUNG DISEASES] | | | | 1963 | | pubmed | title/abstract | irrelevant |
| 1188 | CORCOS A | [Pseudo-cancer of the liver of amebic origin treated with emetine; appearance of bilateral macronodular pulmonary tuberculosis resistant to antibiotics; remarkable results of cortisone] | | | | 1956 | | pubmed | title/abstract | irrelevant |
| 1189 | Tan CZ | Erythematous Plaques on the Buttock | | | | 2016 | | pubmed | title/abstract | irrelevant |
| 1190 | Dannepond C | [Mastitis revealing Churg-Strauss syndrome] | | | | 2014 | | pubmed | title/abstract | irrelevant |
| 1191 | Biggar WD | Malakoplakia and immunosuppressive therapy. Reversal of clinical and leukocyte abnormalities after withdrawal of prednisone and azathioprine | | | | 1985 | | pubmed | title/abstract | irrelevant |
| 1192 | Castillo R | Pyogenic brain abscesses treated with antibiotics in a patient with hemophagocytic lymphohistiocytosis on HLH-94 protocol | | | | 2017 | | pubmed | title/abstract | irrelevant |
| 1193 | Terebelo S | Disseminated nocardiosis in an immunosuppressed patient with systemic lupus erythematosus and neuromyelitis optica spectrum disorder | | | | 2021 | | pubmed | title/abstract | irrelevant |
| 1194 | Bethke G | [Pyostomatitis vegetans. Numerous pale yellow dots on inflammatory red ground - A case report.] | | | | 2017 | | pubmed | title/abstract | irrelevant |
| 1195 | Rossi A | [Practical contribution to the use of a paste containing corticosteroids, antibiotics and enzymes in root canal therapy for acute apical periodontitis] | | | | 1966 | | pubmed | title/abstract | irrelevant |
| 1196 | Melegati G | The influence of local steroid injections, body weight and the length of symptoms in the treatment of painful subcalcaneal spurs with extracorporeal shock wave therapy | | | | 2002 | | pubmed | title/abstract | irrelevant |
| 1197 | Khuong MA | Staphylococcal scaled skin syndrome in an adult: possible influence of non-steroidal anti-inflammatory drugs | | | | 1993 | | pubmed | title/abstract | irrelevant |
| 1198 | Porter MD | Intralesional corticosteroid injection versus extracorporeal shock wave therapy for plantar fasciopathy | | | | 2005 | | pubmed | duplicate | duplicate |
| 1199 | Schneider T | [Recurrent fever in a patient treated with immunosuppressive therapy for Takayasu arteritis] | | | | 1997 | | pubmed | title/abstract | irrelevant |
| 1200 | Yoshida K | [Deep-seated mycosis] | | | | 2014 | | pubmed | title/abstract | irrelevant |
| 1201 | Eslamian F | Extra Corporeal Shock Wave Therapy Versus Local Corticosteroid Injection in the Treatment of Chronic Plantar Fasciitis, a Single Blinded Randomized Clinical Trial | | | | 2016 | | pubmed | duplicate | duplicate |
| 1202 | Vidal JE | Cerebral aspergillosis due to Aspergillus fumigatus in AIDS patient: first culture-proven case reported in Brazil | | | | 2005 | | pubmed | title/abstract | irrelevant |
| 1203 | Patil SD | Acute Compartment Syndrome of the Foot due to Infection After Local Hydrocortisone Injection: A Case Report | | | | 2015 | | pubmed | title/abstract | irrelevant |
| 1204 | Jacob ET | Severe pulmonary nocardiosis in a kidney allograft recipient with a low immunological response | | | | 1985 | | pubmed | title/abstract | irrelevant |
| 1205 | Al Shawabkeh MA | Nasal Type Extranodal Natural Killer/T (NK/T) Cell Lymphoma Presenting as Periorbital Cellulitis: A Case Report | | | | 2016 | | pubmed | duplicate | duplicate |
| 1206 | Wang JL | Rupioid psoriasis associated with arthropathy | | | | 1997 | | pubmed | title/abstract | irrelevant |
| 1207 | Montebugnoli L | Poor oral health is associated with coronary heart disease and elevated systemic inflammatory and haemostatic factors | | | | 2004 | | pubmed | title/abstract | irrelevant |
| 1208 | Ramonas KM | Iris abscess as an unusual presentation of endogenous endophthalmitis in a patient with bacterial endocarditis | | | | 2003 | | pubmed | title/abstract | irrelevant |
| 1209 | Kao MS | Crohn's disease of the vulva | | | | 1975 | | pubmed | title/abstract | irrelevant |
| 1210 | Quartey GR | Decadron in the treatment of cerebral abscess. An experimental study | | | | 1976 | | pubmed | title/abstract | irrelevant |
| 1211 | SANSON J | MYOCARDIAL ABSCESSES | | | | 1963 | | pubmed | title/abstract | irrelevant |
| 1212 | Handler SD | Peritonsillar abscess: a complication of corticosteroid treatment in infectious mononucleosis | | | | 1979 | | pubmed | title/abstract | irrelevant |
| 1213 | Merino-Urrutia W | Cauda equina syndrome following an uneventful spinal anesthesia in a patient undergoing drainage of the Bartholin abscess: A case report | | | | 2018 | | pubmed | title/abstract | irrelevant |
| 1214 | GERSTL B | LIPID STUDIES OF WHITE MATTER AND THALAMUS OF HUMAN BRAINS | | | | 1963 | | pubmed | title/abstract | irrelevant |
| 1215 | Polak-Daćko D | [A case of multiple abscesses of the liver developed during mechanical icterus treated with corticoids] | | | | 1969 | | pubmed | title/abstract | irrelevant |
| 1216 | Park R | Retropharyngeal calcific tendinitis: case report and review of the literature | | | | 2010 | | pubmed | title/abstract | irrelevant |
| 1217 | Paul T | [Successful treatment of a brain abscess with antibiotics and drainage puncture in an 11-year-old boy with a complex cyanotic heart defect] | | | | 1987 | | pubmed | title/abstract | irrelevant |
| 1218 | Ronnekleiv-Kelly SM | Impact of cardiac comorbidity on early outcomes after pancreatic resection | | | | 2014 | | pubmed | title/abstract | irrelevant |
| 1219 | Mulherin D | Efficacy of tibial nerve block, local steroid injection or both in the treatment of plantar heel pain syndrome | | | | 2009 | | pubmed | title/abstract | irrelevant |
| 1220 | Rontal E | Metastatic abscess as a complication of retrograde esophageal dilatation | | | | 1973 | | pubmed | title/abstract | irrelevant |
| 1221 | Reechaipichitkul W | NOCARDIOSIS REVEALED BY THYROID ABSCESS AND PNEUMONIA IN A LIVER TRANSPLANT RECIPIENT | | | | 2015 | | pubmed | title/abstract | irrelevant |
| 1222 | Ushida H | [Systemic lupus erythematosus presenting as a brainstem infarction and hemorrhage during treating retroperitoneal abscess: a case report] | | | | 2001 | | pubmed | title/abstract | irrelevant |
| 1223 | González de Echavarri Perez de Heredia C | [Soft tissue abscess, is it always as it seems?] | | | | 2014 | | pubmed | title/abstract | irrelevant |
| 1224 | Wright FW | The radiological diagnosis of "avascular" renal tumours | | | | 1975 | | pubmed | title/abstract | irrelevant |
| 1225 | Battaglia A | Comparison of Medical Therapy Alone to Medical Therapy with Surgical Treatment of Peritonsillar Abscess | | | | 2018 | | pubmed | duplicate | duplicate |
| 1226 | Xiong Y | Comparison of efficacy of shock-wave therapy versus corticosteroids in plantar fasciitis: a meta-analysis of randomized controlled trials | | | | 2019 | | pubmed | title/abstract | irrelevant |
| 1227 | Xu D | Comparison Between Extracorporeal Shock Wave Therapy and Local Corticosteroid Injection for Plantar Fasciitis | | | | 2020 | | pubmed | duplicate | duplicate |
| 1228 | Aravysky RA | Comparative histopathology of chromomycosis and cladosporiosis in the experiment | | | | 1968 | | pubmed | title/abstract | irrelevant |
| 1229 | Baghai M | Fatal sepsis in a patient with rheumatoid arthritis treated with etanercept | | | | 2001 | | pubmed | title/abstract | irrelevant |
| 1230 | Mitchell WG | Neurocysticercosis and acquired cerebral toxoplasmosis in children | | | | 1999 | | pubmed | title/abstract | irrelevant |
| 1231 | Weng M | Corticosteroid induce leukocytosis during the pain management | | | | 2014 | | pubmed | title/abstract | irrelevant |
| 1232 | Holak H | [Long-term follow-up of bilateral endogenous Klebsiella endophthalmitis] | | | | 2003 | | pubmed | title/abstract | irrelevant |
| 1233 | Dobben GD | Orbital subperiosteal hematoma, cholesterol granuloma, and infection. Evaluation with MR imaging and CT | | | | 1998 | | pubmed | title/abstract | irrelevant |
| 1234 | Corden TE | Pyomyositis during induction chemotherapy for acute lymphocytic leukemia | | | | 1996 | | pubmed | title/abstract | irrelevant |
| 1235 | Mok MY | Necrotizing fasciitis in rheumatic diseases | | | | 2006 | | pubmed | title/abstract | irrelevant |
| 1236 | Saigal K | Bacillus cereus causing intratumoral brain abscess | | | | 2016 | | pubmed | title/abstract | irrelevant |
| 1237 | Yokoiyama S | [Peroral infection of fasted and cortisone-treated mice with Corynebacterium kutscheri (author's transl)] | | | | 1974 | | pubmed | title/abstract | irrelevant |
| 1238 | Sabatiello M | The Pott's puffy tumor: an unusual complication of frontal sinusitis, methods for its detection | | | | 2010 | | pubmed | title/abstract | irrelevant |
| 1239 | Golub B | Lung abscess due to Corynebacterium equi. Report of first human infection | | | | 1967 | | pubmed | title/abstract | irrelevant |
| 1240 | Hansotia P | Chelation therapy in Wegener's granulomatosis. Treatment with EDTA | | | | 1969 | | pubmed | title/abstract | irrelevant |
| 1241 | Skedros JG | Polymicrobial anaerobic infection with a deep abscess in the supraspinous fossa following a subacromial corticosteroid injection | | | | 2018 | | pubmed | title/abstract | irrelevant |
| 1242 | Gaul C | Iatrogenic (para-) spinal abscesses and meningitis following injection therapy for low back pain | | | | 2005 | | pubmed | title/abstract | irrelevant |
| 1243 | Lurie S | Serum testosterone, DHEAS, and prolactin levels in patients with a Bartholin's abscess | | | | 1997 | | pubmed | title/abstract | irrelevant |
| 1244 | Huang K | Platelet-Rich Plasma Versus Corticosteroid Injections in the Management of Elbow Epicondylitis and Plantar Fasciitis: An Updated Systematic Review and Meta-analysis | | | | 2020 | | pubmed | title/abstract | irrelevant |
| 1245 | Shetty SH | Platelet-Rich Plasma Has Better Long-Term Results Than Corticosteroids or Placebo for Chronic Plantar Fasciitis: Randomized Control Trial | | | | 2019 | | pubmed | duplicate | duplicate |
| 1246 | García González LA | [Brain abscess secondary to rhinosinusitis. Therapeutical modalities. Exposition of one case] | | | | 2004 | | pubmed | title/abstract | irrelevant |
| 1247 | Díaz-Pedroche C | [Cerebral abscess due to Gemella haemolysans] | | | | 2005 | | pubmed | title/abstract | irrelevant |
| 1248 | Rousset JJ | [Apropos of a case of hepatic distomatosis due to Fasciola gigantica contracted in Cameroon] | | | | 1968 | | pubmed | title/abstract | irrelevant |
| 1249 | Shibata M | A case of IgG4-related hepatic inflammatory pseudotumor replaced by an abscess after steroid treatment | | | | 2016 | | pubmed | title/abstract | irrelevant |
| 1250 | Simmons DJ | Staphylococcal kidney abscesses in rats treated with corticosteroids | | | | 1977 | | pubmed | title/abstract | irrelevant |
| 1251 | Haid RW Jr | Epidural lipomatosis simulating an epidural abscess: case report and literature review | | | | 1987 | | pubmed | title/abstract | irrelevant |
| 1252 | Glotzer DJ | Surgical management of regional enteritis | | | | 1971 | | pubmed | title/abstract | irrelevant |
| 1253 | Goldstein S | A method for differentiating nonspecific irritants from anti-inflammatory agents using the carrageenin abscess test | | | | 1966 | | pubmed | title/abstract | irrelevant |
| 1254 | Dietrich U | MRI of intracranial toxoplasmosis after bone marrow transplantation | | | | 2000 | | pubmed | title/abstract | irrelevant |
| 1255 | Ricciardi B | Cerebral abscess caused by Capnocytophaga spp in an immunocompetent subject: case report | | | | 2008 | | pubmed | title/abstract | irrelevant |
| 1256 | Atkins KL | Image intensifier-guided injection of corticosteroid and local anesthetic agent for the treatment of recalcitrant plantar fasciitis | | | | 2010 | | pubmed | title/abstract | irrelevant |
| 1257 | Chen YH | Prognostic Factors and Visual Outcomes of Pyogenic Liver Abscess-Related Endogenous Klebsiella pneumoniae Endophthalmitis: A 20-year retrospective review | | | | 2019 | | pubmed | title/abstract | irrelevant |
| 1258 | Guettrot-Imbert G | F-18 FDG-PET/CT in aseptic abscesses with recurrent febrile abdominal pain | | | | 2011 | | pubmed | title/abstract | irrelevant |
| 1259 | Choma T | Epidural abscess as a delayed complication of spinal instrumentation in scoliosis surgery: a case of progressive neurologic dysfunction with complete recovery | | | | 2008 | | pubmed | title/abstract | irrelevant |
| 1260 | Bozzo L | Lipid components of human dental periapical lesions. Histochemical and histophysical observations | | | | 1972 | | pubmed | title/abstract | irrelevant |
| 1261 | SINGH KS | 5-HYDROXYTRYPTAMINE (5-HT) CONTENT OF CEREBROSPINAL FLUID IN INFECTIVE CONDITIONS OF THE CENTRAL NERVOUS SYSTEM | | | | 1964 | | pubmed | title/abstract | irrelevant |
| 1262 | FELDTHUS M | [ELABORATE FUCIDIN TEST IN A GENERAL MEDICAL WARD] | | | | 1964 | | pubmed | title/abstract | irrelevant |
| 1263 | Ramaswamy A | "Better late than never" | | | | 2011 | | pubmed | title/abstract | irrelevant |
| 1264 | Fialová S | [Pharmacotherapy of periodontitis] | | | | 1974 | | pubmed | title/abstract | irrelevant |
| 1265 | Polak A | Experimental infection of mice by Fonsecaea pedrosoi and Wangiella dermatitidis | | | | 1984 | | pubmed | title/abstract | irrelevant |
| 1266 | Cvetković T | [Treatment of root canal and periapical processes with hemotherapeutics and antibiotics. Therapeutic results with Antipulpit II] | | | | 1968 | | pubmed | title/abstract | irrelevant |
| 1267 | Yamaguchi M | [Epidural abscess associated with epidural block in a patient with immunosuppressive disease] | | | | 1999 | | pubmed | title/abstract | irrelevant |
| 1268 | Janowski R | [Pulmonary embolism as a complication of nephrotic syndrome--case report and therapeutic management] | | | | 1997 | | pubmed | title/abstract | irrelevant |
| 1269 | Waldman SD | Cervical epidural abscess after cervical epidural nerve block with steroids | | | | 1991 | | pubmed | title/abstract | irrelevant |
| 1270 | Kienbacher G | [A case of a tumorsimulating expansion caused by anabolic androgen steroids in body building] | | | | 2007 | | pubmed | title/abstract | irrelevant |
| 1271 | Rodríguez-Granger J | [Cerebral abscess in an immunosuppressed patient] | | | | 2005 | | pubmed | title/abstract | irrelevant |
| 1272 | Han ER | Inhaled corticosteroid-related tooth problems in asthmatics | | | | 2009 | | pubmed | title/abstract | irrelevant |
| 1273 | Paffetti A | Successful meropenem therapy of a brain abscess and meningitis arising from acute purulent otomastoiditis: case report | | | | 1998 | | pubmed | title/abstract | irrelevant |
| 1274 | Li J | Azathioprine combined with corticosteroids for recurrent cutaneous necrotizing eosinophilic vasculitis secondary to eosinophilic dermatitis | | | | 2023 | | pubmed | title/abstract | irrelevant |
| 1275 | Markowitz J | Long-term 6-mercaptopurine treatment in adolescents with Crohn's disease | | | | 1990 | | pubmed | title/abstract | irrelevant |
| 1276 | Segovia E | [Serum lipoproteins in amebic hepatic abscess] | | | | 1973 | | pubmed | title/abstract | irrelevant |
| 1277 | Pappas G | Pulmonary surgery in immunosuppressed patients | | | | 1970 | | pubmed | title/abstract | irrelevant |
| 1278 | Herr A | [A thirty-year old bodybuilder with septic shock and ARDS from abuse of anabolic steroids] | | | | 2002 | | pubmed | title/abstract | irrelevant |
| 1279 | Zeng Y | AIDS-Related Kaposi Sarcoma Associated with Steroid-Unresponsive Periorbital Lymphedema that Responded to Chemotherapy | | | | 2023 | | pubmed | title/abstract | irrelevant |
| 1280 | Zachariasen R | Oral manifestations of metabolic bone disease: vitamin D and osteoporosis | | | | 1990 | | pubmed | title/abstract | irrelevant |
| 1281 | Savage MW | Silent nocardia cerebral abscesses in treated dermatomyositis | | | | 1990 | | pubmed | title/abstract | irrelevant |
| 1282 | Gill NJ | Model of amoebic liver abscess in cholesterol fed guinea pigs through intracaecal infection route | | | | 1983 | | pubmed | title/abstract | irrelevant |
| 1283 | CHARPIN J | [Use of an injectable delayed corticoid in the gastric ulcer patient in respiratory pathology] | | | | 1962 | | pubmed | title/abstract | irrelevant |
| 1284 | Listernick R | A 2 1/2-year-old boy with leg pain | | | | 2009 | | pubmed | title/abstract | irrelevant |
| 1285 | Higuchi T | Deep vein thrombosis associated with factor V inhibitor followed by immune thrombocytopenia | | | | 2012 | | pubmed | title/abstract | irrelevant |
| 1286 | Patrikiou A | [Non-steroid anti-inflammatory agents (NSAIDs) in dentistry] | | | | 1986 | | pubmed | title/abstract | irrelevant |
| 1287 | Henry DD | Low viral load post-transplant lymphoproliferative disease localized within the tongue | | | | 2008 | | pubmed | title/abstract | irrelevant |
| 1288 | Basaria S | Case in point. Cerebral toxoplasmosis | | | | 2000 | | pubmed | title/abstract | irrelevant |
| 1289 | Golub R | Gallstone shrapnel contamination during laparoscopic cholecystectomy | | | | 1994 | | pubmed | title/abstract | irrelevant |
| 1290 | Morii K | Reversible splenial lesion of the corpus callosum associated with bacterial meningitis | | | | 2014 | | pubmed | title/abstract | irrelevant |
| 1291 | Okamoto K | [A case of cerebral aspergillosis associated with induction chemotherapy for acute lymphoblastic leukemia] | | | | 1996 | | pubmed | title/abstract | irrelevant |
| 1292 | Seth I | The role of corticosteroid injections in treating plantar fasciitis: A systematic review and meta-analysis | | | | 2023 | | pubmed | title/abstract | irrelevant |
| 1293 | Amin N | Amoebiasis and corticosteroids | | | | 1978 | | pubmed | title/abstract | irrelevant |
| 1294 | Pitt Ford TR | Tissue reactions to two root canal sealers containing formaldehyde | | | | 1985 | | pubmed | title/abstract | irrelevant |
| 1295 | Costa CA | Influence of inflammation on parasitism and area of experimental amoebic liver abscess: an immunohistochemical and morphometric study | | | | 2011 | | pubmed | title/abstract | irrelevant |
| 1296 | Jimenez-Galanes Marchan S | Disseminated nocardiosis: a rare infectious complication following non-heart-beating donor liver transplantation | | | | 2009 | | pubmed | title/abstract | irrelevant |
| 1297 | Pittard A | A child with difficulty swallowing | | | | 2009 | | pubmed | title/abstract | irrelevant |
| 1298 | Chivite D | Retropharyngeal abscess caused by Streptococcus agalactiae | | | | 1998 | | pubmed | title/abstract | irrelevant |
| 1299 | Fatori Popovic S | [Pregnancy and lactation period: Which antibiotic and rinsing solutions?] | | | | 2016 | | pubmed | title/abstract | irrelevant |
| 1300 | Karjigi S | Primary Neuritic Hansen's Disease presenting as Ulnar Nerve Abscess in a Human Immunodeficiency Virus Positive Patient | | | | 2015 | | pubmed | title/abstract | irrelevant |
| 1301 | Pahud BA | Preterm neonates with candidal brain microabscesses: a case series | | | | 2009 | | pubmed | title/abstract | irrelevant |
| 1302 | Moore TC | The period and nature of hazard in clinical renal transplantation. I. The hazard to patient survival | | | | 1969 | | pubmed | title/abstract | irrelevant |
| 1303 | Hansson L | Multiple renal abscesses in a patient with rapidly progressive glomerulonephritis while on immunosuppressive treatment | | | | 1971 | | pubmed | title/abstract | irrelevant |
| 1304 | Weingarten TN | Septic facet joint arthritis after a corticosteroid facet injection | | | | 2006 | | pubmed | title/abstract | irrelevant |
| 1305 | Miyara T | Rapidly expanding lung abscess caused by Legionella pneumophila in immunocompromised patients: a report of two cases | | | | 2002 | | pubmed | title/abstract | irrelevant |
| 1306 | Miret C | [Clostridium ramosum: a rare cause of brain abscess] | | | | 1998 | | pubmed | title/abstract | irrelevant |
| 1307 | Localio SA | Panel discussion on surgical management of inflammatory bowel disease | | | | 1973 | | pubmed | title/abstract | irrelevant |
| 1308 | Tay ST | Nocardia kroppenstedtii: a rare pathogen isolated from the spinal vertebral abscess of a patient on long-term immunosuppressive therapy | | | | 2021 | | pubmed | title/abstract | irrelevant |
| 1309 | Saishoji Y | Sternoclavicular Septic Arthritis Caused by Parvimonas micra and Fusobacterium nucleatum Infection with Intra-articular Corticosteroid Administration | | | | 2024 | | pubmed | title/abstract | irrelevant |
| 1310 | Doulberis M | A RARE CASE OF CROHN DISEASE COMPLICATED WITH STEROID MONOTHERAPY-RELATED RETROPHARYNGEAL ABSCESS AND INITIALLY MISINTERPRETED PYODERMA GANGRENOSUM DEVELOPMENT | | | | 2018 | | pubmed | title/abstract | irrelevant |
| 1311 | Lyons BE | Short term, high dose corticosteroids in computed tomographic staging of experimental brain abscess | | | | 1982 | | pubmed | title/abstract | irrelevant |
| 1312 | Radhakrishnan N | Conidiobolomycosis in relapsed acute lymphoblastic leukemia | | | | 2009 | | pubmed | duplicate | duplicate |
| 1313 | Brincourt J | [Liquefying effect on suppurations of an oral dose of calciferol] | | | | 1969 | | pubmed | title/abstract | irrelevant |
| 1314 | Hsiao MY | Comparative effectiveness of autologous blood-derived products, shock-wave therapy and corticosteroids for treatment of plantar fasciitis: a network meta-analysis | | | | 2015 | | pubmed | duplicate | duplicate |
| 1315 | Leiding JW | Corticosteroid therapy for liver abscess in chronic granulomatous disease | | | | 2012 | | pubmed | title/abstract | irrelevant |
| 1316 | Fox TP | Plantar fascia calcification a sequelae of corticosteroid injection in the treatment of recalcitrant plantar fasciitis | | | | 2013 | | pubmed | title/abstract | irrelevant |
| 1317 | Guy SD | Legionella pneumophila lung abscess associated with immune suppression | | | | 2011 | | pubmed | title/abstract | irrelevant |
| 1318 | Onyima C | Epidural abscess after lumbar medial branch blocks in a patient on disease-modifying anti-rheumatic drug and corticosteroid | | | | 2021 | | pubmed | title/abstract | irrelevant |
| 1319 | Hiremath M | Heparin in the long-term management of ligneous conjunctivitis: a case report and review of literature | | | | 2011 | | pubmed | duplicate | duplicate |
| 1320 | Ali NM | Nerve abscess in Hansen's disease as part of immune reconstitution inflammatory syndrome: a case report | | | | 2017 | | pubmed | title/abstract | irrelevant |
| 1321 | César A | Cutaneous Richter Syndrome mimicking a lower limb cellulitis infection - a case report and review of the literature | | | | 2016 | | pubmed | title/abstract | irrelevant |
| 1322 | Wagner Y | Pediatricians' oral health recommendations for 0- to 3-year-old children: results of a survey in Thuringia, Germany | | | | 2014 | | pubmed | title/abstract | irrelevant |
| 1323 | Cheatham BD | A dental complication involving Pseudomonas during chemotherapy for acute lymphoblastic leukemia | | | | 1994 | | pubmed | title/abstract | irrelevant |
| 1324 | Granel B | [Brain nocardiasis of good outcome occurring in a heart transplant recipient] | | | | 2003 | | pubmed | title/abstract | irrelevant |
| 1325 | Scharfetter F | [The cerebral abscess (author's transl)] | | | | 1980 | | pubmed | title/abstract | irrelevant |
| 1326 | Waxman AD | Gallium scanning in cerebral and cranial disorders | | | | 1980 | | pubmed | title/abstract | irrelevant |
| 1327 |  | Medical grand rounds from the University of Alabama Medical Center | | | | 1968 | | pubmed | title/abstract | irrelevant |
| 1328 | Buguslavskaia MI | [Specific features of the electrolyte compostion of the blood and urine and 17-ketosteroid excretion in chronic lung suppuration and bronchial asthma] | | | | 1966 | | pubmed | title/abstract | irrelevant |
| 1329 | SEMONOV VI | [CLINICAL SIGNIFICANCE OF CHANGES IN SOME INDICATORS OF PROTEIN AND LIPID METABOLISM IN PATIENTS WITH SUPPURATIVE LUNG DISEASE] | | | | 1963 | | pubmed | title/abstract | irrelevant |
| 1330 | Benbouzid MA | [Cervicofacial cellulitis revealing cutaneous lymphomas] | | | | 2007 | | pubmed | title/abstract | irrelevant |
| 1331 | Marshall JG | Factors associated with endodontic posttreatment pain | | | | 1993 | | pubmed | duplicate | duplicate |
| 1332 | Yoo D | Brain abscesses due to Pseudallescheria boydii associated with primary non-Hodgkin's lymphoma of the central nervous system: a case report and literature review | | | | 1985 | | pubmed | title/abstract | irrelevant |
| 1333 | Biagi F | [Amebiasis, a challenge to the understanding of pathological mechanism] | | | | 1967 | | pubmed | title/abstract | irrelevant |
| 1334 | Jain A | Severe hyperphosphatemia resulting from high-dose liposomal amphotericin in a child with leukemia | | | | 2003 | | pubmed | title/abstract | irrelevant |
| 1335 | Cleveland KO | Listerial brain abscess in a patient with chronic lymphocytic leukemia treated with fludarabine | | | | 1993 | | pubmed | title/abstract | irrelevant |
| 1336 | Villar Rodríguez JL | [Abscessing rhombencephalitis caused by Listeria in an iummunodepressed patient] | | | | 1990 | | pubmed | title/abstract | irrelevant |
| 1337 | Erden T | Outcome of Corticosteroid Injections, Extracorporeal Shock Wave Therapy, and Radiofrequency Thermal Lesioning for Chronic Plantar Fasciitis | | | | 2021 | | pubmed | title/abstract | irrelevant |
| 1338 | Gasch O | [Cerebral aspergillosis in an HIV-infected patient: unsuccessful outcome despite combined antifungal therapy. ] | | | | 2009 | | pubmed | title/abstract | irrelevant |
| 1339 | Cortés-Pérez I | Efficacy of extracorporeal shockwave therapy, compared to corticosteroid injections, on pain, plantar fascia thickness and foot function in patients with plantar fasciitis: A systematic review and meta-analysis | | | | 2024 | | pubmed | title/abstract | irrelevant |
| 1340 | Holtkamp M | Cerebral toxoplasmosis in a patient with common variable immunodeficiency | | | | 2004 | | pubmed | title/abstract | irrelevant |
| 1341 | Sell M | Primary cerebral toxoplasmosis: a rare case of ventriculitis and hydrocephalus in AIDS | | | | 2005 | | pubmed | title/abstract | irrelevant |
| 1342 | Hodohara K | Disseminated subcutaneous Nocardia asteroides abscesses in a patient after bone marrow transplantation | | | | 1993 | | pubmed | title/abstract | irrelevant |
| 1343 | Kongara K | Effect of non-steroidal anti-inflammatory drugs on glomerular filtration rate and urinary N-acetyl-β-D-glucosaminidase activity in cats after dental surgery | | | | 2020 | | pubmed | title/abstract | irrelevant |
| 1344 | de CAMP | [Supplementary corticosteroid treatment in lung abscesses] | | | | 1961 | | pubmed | title/abstract | irrelevant |
| 1345 | Hesarur N | Case Report: Chronic Fungal Meningitis Masquerading as Tubercular Meningitis | | | | 2020 | | pubmed | title/abstract | irrelevant |
| 1346 | Chauhan SL | Calcification of peripheral nerves in leprosy | | | | 1996 | | pubmed | title/abstract | irrelevant |
| 1347 | Woodruff AW | Advances in the treatment of tropical diseases | | | | 1968 | | pubmed | title/abstract | irrelevant |
| 1348 | Jin C | The efficacy and safety of different doses of calcitriol combined with neutral phosphate in X-linked hypophosphatemia: a prospective study | | | | 2022 | | pubmed | title/abstract | irrelevant |
| 1349 | Ebersole JL | Comparative virulence of periodontopathogens in a mouse abscess model | | | | 1995 | | pubmed | title/abstract | irrelevant |
| 1350 | Mortensen JE | Modification of bactericidal fatty acids by an enzyme of Staphylococcus aureus | | | | 1992 | | pubmed | title/abstract | irrelevant |
| 1351 | Louria DB | Symposium on infectious complications of neoplastic disease (Part I). Introduction and epidemiology | | | | 1984 | | pubmed | title/abstract | irrelevant |
| 1352 | Graybill JR | Disseminated mycobacteriosis due to Mycobacterium abcessus in two recipients of renal homografts | | | | 1974 | | pubmed | title/abstract | irrelevant |
| 1353 | Amromin GD | Massive cerebral Aspergillus abscess in a leukemic child. Case report | | | | 1971 | | pubmed | title/abstract | irrelevant |
| 1354 | Victoria-Hernández JA | Case report: multiple and atypical amoebic cerebral abscesses resistant to treatment | | | | 2020 | | pubmed | title/abstract | irrelevant |
| 1355 | Iio K | Secondary haemophagocytic lymphohistiocytosis associated with metronidazole | | | | 2019 | | pubmed | title/abstract | irrelevant |
| 1356 | Lambe DW Jr | Pathogenicity of Staphylococcus lugdunensis, Staphylococcus schleiferi, and three other coagulase-negative staphylococci in a mouse model and possible virulence factors | | | | 1990 | | pubmed | title/abstract | irrelevant |
| 1357 | Gordon AM | The nitroblue tetrazolium (NBT) test in renal transplantation | | | | 1974 | | pubmed | title/abstract | irrelevant |
| 1358 | Szreder W | Effect of artificially induced abacterial erysipelas and of chronic aseptic abscess on human and experimental neoplasms | | | | 1968 | | pubmed | title/abstract | irrelevant |
| 1359 | Lee HJ | [A case of Henoch-Schönlein purpura with psoas muscle abscess and full-blown gastrointestinal complications] | | | | 2007 | | pubmed | title/abstract | irrelevant |
| 1360 | Koch CA | Abnormal ACTH-stimulation test in a patient with AIDS: adrenal insufficiency or toxoplasmosis? | | | | 2001 | | pubmed | title/abstract | irrelevant |
| 1361 | Larmas M | Oral manifestations of familial hypophosphatemic rickets after phosphate supplement therapy: a review of the literature and report of case | | | | 1991 | | pubmed | title/abstract | irrelevant |
| 1362 | Ravitch MM | Observations on the healing of wounds of the intestines | | | | 1975 | | pubmed | title/abstract | irrelevant |
| 1363 | Zhou C | Idiopathic thrombocytopenic purpura with brain abscess caused by Nocardia farcinica diagnosed using metagenomics next-generation sequencing of the cerebrospinal fluid: a case report | | | | 2021 | | pubmed | title/abstract | irrelevant |
| 1364 | Bodey GP | Central nervous system aspergillosis following steroidal therapy for allergic bronchopulmonary aspergillosis | | | | 1993 | | pubmed | title/abstract | irrelevant |
| 1365 | Chatterjee DK | Antiamoebic activity of chonemorphine, a steroidal alkaloid, in experimental models | | | | 1987 | | pubmed | title/abstract | irrelevant |
| 1366 | Wang WY | [Analysis of risk factors for anastomotic infectious complications following bowel resection for Crohn disease] | | | | 2013 | | pubmed | title/abstract | irrelevant |
| 1367 | Guner S | Effectiveness of local tenoxicam versus corticosteroid injection for plantar fasciitis treatment | | | | 2013 | | pubmed | title/abstract | irrelevant |
| 1368 | Goldberg MH | Health hazard: the inappropriate use of corticosteroids in dentistry | | | | 1981 | | pubmed | title/abstract | irrelevant |
| 1369 | Falcone MW | Sporotrichosis and nocardiosis in a patient with Boeck's sarcoid | | | | 1969 | | pubmed | title/abstract | irrelevant |
| 1370 | Barclay MF | The anesthetic management of the "wet lung" | | | | 1967 | | pubmed | title/abstract | irrelevant |
| 1371 | Chatzoulis G | Primary omental abscess with increased concentrations of carcinoembryonic antigen | | | | 2014 | | pubmed | title/abstract | irrelevant |
| 1372 | Molnar S | Mineral metabolism and microstructural defects in primate teeth | | | | 1975 | | pubmed | title/abstract | irrelevant |
| 1373 | Trokhim IuI | [Control of intracranial pressure and prevention of hypertension following brain surgery] | | | | 1971 | | pubmed | title/abstract | irrelevant |
| 1374 | Tsur I | Meningoencephalitis and brain abscessation due to Escherichia coli in a 2 week old alpaca cria | | | | 1996 | | pubmed | title/abstract | irrelevant |
| 1375 | de CAMP | [Experiences with corticosteroid therapy in a lung clinic] | | | | 1961 | | pubmed | title/abstract | irrelevant |
| 1376 | Einwag J | [Endodontics in primary dentition] | | | | 1991 | | pubmed | title/abstract | irrelevant |
| 1377 | Hauser N | An Immunocompromised Woman with a Brain Lesion | | | | 2020 | | pubmed | title/abstract | irrelevant |
| 1378 | Al-Rawashdeh BM | Vitamin D Levels in Children with Recurrent Acute Tonsillitis in Jordan: A Case-Control Study | | | | 2022 | | pubmed | title/abstract | irrelevant |
| 1379 | CHIGIRINSKII AN | [On pulmonary lesions in prolonged corticosteroid therapy] | | | | 1961 | | pubmed | title/abstract | irrelevant |
| 1380 | Hagiya H | Severe soft tissue infection of the lower extremity caused by Haemophilus influenzae (serotype f, biotype II) in an adult patient | | | | 2012 | | pubmed | title/abstract | irrelevant |
| 1381 | Forbes N | Necrotizing fasciitis and non steroidal anti-inflammatory drugs: a case series and review of the literature | | | | 2001 | | pubmed | title/abstract | irrelevant |
| 1382 | Koka VK | Spinal epidural abscess after corticosteroid injections | | | | 2002 | | pubmed | title/abstract | irrelevant |
| 1383 | Tanimoto-Weki M | [Inoculation of E. histolytica into hamsters under the effect of immunosuppressive drugs] | | | | 1974 | | pubmed | title/abstract | irrelevant |
| 1384 | Luo Z | The role of male hormones in bacterial infections: enhancing Staphylococcus aureus virulence through testosterone-induced Agr activation | | | | 2024 | | pubmed | title/abstract | irrelevant |
| 1385 | Hoelzer BC | Paraspinal abscess complicated by endocarditis following a facet joint injection | | | | 2008 | | pubmed | title/abstract | irrelevant |
| 1386 | Ono M | Nocardia exalbida brain abscess in a patient with follicular lymphoma | | | | 2008 | | pubmed | title/abstract | irrelevant |
| 1387 | Oto J | An adult patient with Kabuki syndrome presenting with Henoch-Schönlein purpura complicated with pulmonary hemorrhage | | | | 2008 | | pubmed | title/abstract | irrelevant |
| 1388 | Davis KA | Combination therapy that targets secondary pulmonary changes after abdominal trauma | | | | 2001 | | pubmed | title/abstract | irrelevant |
| 1389 | Huber W | [Pyrimethamine-sulfadiazine resistant cerebral toxoplasmosis in AIDS] | | | | 1995 | | pubmed | title/abstract | irrelevant |
| 1390 | Gago R | Severe systemic inflammatory response syndrome immediately after spinal surgery in a patient with axial gout | | | | 2018 | | pubmed | title/abstract | irrelevant |
| 1391 | Elizondo-Rodríguez J | Comparison of Botulinum Toxin A, Corticosteroid, and Anesthetic Injection for Plantar Fasciitis | | | | 2021 | | pubmed | duplicate | duplicate |
| 1392 | Jackisch T | [Gas gangrene with ulcerative colitis under immunosuppressive therapy: report of a case] | | | | 2006 | | pubmed | title/abstract | irrelevant |
| 1393 | Silver HS | Hypopituitarism secondary to cavernous sinus thrombosis | | | | 1983 | | pubmed | title/abstract | irrelevant |
| 1394 | Castillo RF | Pyoderma gangrenosum developing over an arteriovenous fistula scar | | | | 2011 | | pubmed | title/abstract | irrelevant |
| 1395 | Megel H | The anti-inflammatory actions of tilorone hydrochloride | | | | 1975 | | pubmed | title/abstract | irrelevant |
| 1396 | Mori S | [Distribution of antibiotics in various parts of the body, with special reference to visceral organs and gonads] | | | | 1971 | | pubmed | title/abstract | irrelevant |
| 1397 | Ahmad I | Methicillin-resistant Staphylococcus aureus neck infections resulting in a delayed abscess and a tracheo-oesophageal fistula | | | | 1999 | | pubmed | title/abstract | irrelevant |
| 1398 | Miyawaki S | [Multiple liver abscesses successfully treated by intraportal administration of amphotericin B in a case of AML (M2)] | | | | 1986 | | pubmed | title/abstract | irrelevant |
| 1399 | Inaba A | [Cerebral toxoplasmosis after umbilical cord blood transplantation diagnosed by the detection of anti-toxoplasma specific IgM antibody in cerebrospinal fluid] | | | | 2014 | | pubmed | title/abstract | irrelevant |
| 1400 | Yoshikawa TT | Role of anaerobic bacteria in subdural empyema. Report of four cases and review of 327 cases from the English literature | | | | 1975 | | pubmed | title/abstract | irrelevant |
| 1401 | Schulz V | [Idiopathic cholesterol pneumonia with transition into a pulmonary cholesterol granulomatosis] | | | | 1973 | | pubmed | title/abstract | irrelevant |
| 1402 | LISIN N | [CONTRIBUTION TO THE EXPERIMENTAL STUDY OF A NEW ANTI-INFLAMMATORY AGENT, "A-230"] | | | | 1964 | | pubmed | title/abstract | irrelevant |
| 1403 | Shen A | A 12-Month-Old Boy With Bilateral Facial Swelling and Proptosis | | | | 2018 | | pubmed | title/abstract | case report |
| 1404 | Povzun SA | [Artificial illness as a result of non-medical use of anabolic androgenic steroids: A case report and a review of literature] | | | | 2016 | | pubmed | title/abstract | case report |
| 1405 | Leak D | Acute Wegener's granulomatosis | | | | 1967 | | pubmed | title/abstract | case report |
| 1406 | Koliada IS | [Lung abscesses developing during treatment with corticosteroid preparations] | | | | 1977 | | pubmed | title/abstract | case report |
| 1407 | Hutchinson D | Sigmoid diverticular abscess perforation in 2 patients with rheumatoid arthritis treated with high dose corticosteroids. A cautionary tale | | | | 2001 | | pubmed | title/abstract | case report |
| 1408 | Morton JR | Corticosteroids and malnutrition. Aspergillus lung abscess in an asthmatic child | | | | 1980 | | pubmed | title/abstract | case report |
| 1409 | Vidal JE | First case report of eosinophilic meningitis associated with cerebral toxoplasmosis in an HIV-positive patient | | | | 2020 | | pubmed | title/abstract | case report |
| 1410 | Seino Y | A case of rickets due to external biliary fistula--study on vitamin D effect upon Ca absorption and retention | | | | 1971 | | pubmed | title/abstract | case report |
| 1411 | Cello JP | Cholestasis in ulcerative colitis. Long term complications and medical therapy | | | | 1977 | | pubmed | title/abstract | case report |
| 1412 | Kent S | Systematic review of the role of corticosteroids in cervicofacial infections | | | | 2019 | | pubmed | title/abstract | review |
| 1413 | Limeback H | The effects of hypocalcemia/hypophosphatemia on porcine bone and dental hard tissues in an inherited form of type 1 pseudo-vitamin D deficiency rickets | | | | 1992 | | pubmed | title/abstract | irrelevant |
| 1414 | Esquivel López A | [Serum bile acids in hepatobiliary disease] | | | | 1982 | | pubmed | title/abstract | irrelevant |
| 1415 | Vandersteen PR | Bacterial infections of the skin | | | | 1974 | | pubmed | title/abstract | irrelevant |
| 1416 | Karjalainen S | Oral health of 3-year-old children and their parents after 29 months of child-focused antiatherosclerotic dietary intervention in a prospective randomized trial | | | | 1997 | | pubmed | title/abstract | irrelevant |
| 1417 | Hansford JR | Bacillus cereus bacteremia and multiple brain abscesses during acute lymphoblastic leukemia induction therapy | | | | 2014 | | pubmed | title/abstract | irrelevant |
| 1418 | Huang HL | Klebsiella pneumoniae bacteremia and renosplenic abscesses without intestinal symptoms as the initial manifestations of non-steroidal anti-inflammatory drug-induced colitis: a rare case report | | | | 2013 | | pubmed | title/abstract | irrelevant |
| 1419 | Collis DH | Zygomatic haematoma in an 11-month-old helps diagnose retropharyngeal abscess, with concurrent tonsillitis and subsequent infant tonsillectomy | | | | 2016 | | pubmed | title/abstract | irrelevant |
| 1420 | Hraiech S | Lung abscess following ventilator-associated pneumonia during COVID-19: a retrospective multicenter cohort study | | | | 2023 | | pubmed | title/abstract | irrelevant |
| 1421 | Lai CC | Disseminated Nocardia farcinica infection in a uraemia patient with idiopathic thrombocytopenia purpura receiving steroid therapy | | | | 2005 | | pubmed | title/abstract | irrelevant |
| 1422 | Logan HL | Pain and immunologic response to root canal treatment and subsequent health outcomes | | | | 2001 | | pubmed | title/abstract | irrelevant |
| 1423 | Alekseenko AV | [Treatment of acute lung abscesses using interstitial electrophoresis] | | | | 1983 | | pubmed | title/abstract | irrelevant |
| 1424 | Reulen HJ | Vasogenic brain oedema. New aspects in its formation, resolution and therapy | | | | 1976 | | pubmed | title/abstract | irrelevant |
| 1425 | Jakób-Dolezal K | [Treatment of pulp gangrene complicated by periapical lesions with polyantibiotic mixture and hydrocortisone. I. Introduction] | | | | 1970 | | pubmed | title/abstract | irrelevant |
| 1426 | Archampong EQ | Peritonitis from amoebic liver abscess | | | | 1972 | | pubmed | title/abstract | irrelevant |
| 1427 | Myers ML | A nutrition study of school children in a depressed urban district. II. Physical and biochemical findings | | | | 1968 | | pubmed | title/abstract | irrelevant |
| 1428 | Lozano-Masdemont B | Staphylococcus lugdunensis: An Emerging Pathogen in Skin and Soft Tissue Infections | | | | 2015 | | pubmed | title/abstract | irrelevant |
| 1429 | López-Duarte M | Cerebral toxoplasmosis after autologous peripheral blood stem cell transplantation | | | | 2003 | | pubmed | title/abstract | irrelevant |
| 1430 | Ratcliffe GE | Amoebic disease precipitated by corticosteroids prescribed for tuberculous pleural effusions | | | | 1988 | | pubmed | title/abstract | irrelevant |
| 1431 |  | Case records of the Massachusetts General Hospital. Weekly clinicopathological exercises. Case 21-1973 | | | | 1973 | | pubmed | title/abstract | irrelevant |
| 1432 | Rosa Duque JS | Candida Tropicalis renal microabscesses in a child with leukemia confirmed using nucleic acid amplification and recovery after prolonged antifungal and corticosteroid treatment | | | | 2019 | | pubmed | title/abstract | irrelevant |
| 1433 | Hobson DTG | Pregnancy complicated by recurrent brain abscess after extraction of an infected tooth | | | | 2011 | | pubmed | duplicate | duplicate |
| 1434 | Orefice G | Cerebral toxoplasmosis and AIDS. Clinical, neuroradiological and immunological findings in 15 patients | | | | 1992 | | pubmed | title/abstract | irrelevant |
| 1435 | Wang T | Nocardiosis in Kidney Disease Patients under Immunosuppressive Therapy: Case Report and Literature Review | | | | 2019 | | pubmed | title/abstract | irrelevant |
| 1436 | Seta N | A possible novel mechanism of opportunistic infection in systemic lupus erythematosus, based on a case of toxoplasmic encephalopathy | | | | 2002 | | pubmed | title/abstract | irrelevant |
| 1437 | Yoshimura K | [Non-Hodgkin's lymphoma with Toxoplasma encephalitis] | | | | 1999 | | pubmed | title/abstract | irrelevant |
| 1438 | Minicucci EM | Dental abnormalities in children after chemotherapy treatment for acute lymphoid leukemia | | | | 2003 | | pubmed | title/abstract | irrelevant |
| 1439 | Ang TW | The effectiveness of corticosteroid injection in the treatment of plantar fasciitis | | | | 2015 | | pubmed | title/abstract | irrelevant |
| 1440 | Zhao J | Extracorporeal shock wave therapy versus corticosteroid injection for chronic plantar fasciitis: A protocol of randomized controlled trial | | | | 2020 | | pubmed | title/abstract | irrelevant |
| 1441 | Howard DL | Temporal Trends in the Uptake and Continuation of the Etonogestrel Implant in a Large Private Practice Setting | | | | 2018 | | pubmed | title/abstract | irrelevant |
| 1442 | Fava LR | Acute apical periodontitis: incidence of post-operative pain using two different root canal dressings | | | | 1998 | | pubmed | duplicate | duplicate |
| 1443 | Yiannopoulou KG | Reversal of tetraparesis due to staphylococcal cervical spondylodiscitis associated with anterior epidural abscess after conservative treatment | | | | 2008 | | pubmed | title/abstract | irrelevant |
| 1444 | Currie BJ | Strongyloides stercoralis infection as a manifestation of immune restoration syndrome? | | | | 2005 | | pubmed | title/abstract | irrelevant |
| 1445 | Nayeem MA | Detergent dissection of membrane proteins of Entamoeba histolytica and its effect on lymphokine release in in vitro | | | | 1991 | | pubmed | title/abstract | irrelevant |
| 1446 | Castaño-Amores C | Cerebral toxoplasmosis associated with treatment with rituximab, azathioprine and prednisone for dermatomyositis | | | | 2021 | | pubmed | title/abstract | irrelevant |
| 1447 |  | Chemotherapy of brain abscess | | | | 1978 | | pubmed | title/abstract | irrelevant |
| 1448 | Reulen HJ | [Means, methods and measures of emergency treatment of comatose patients with acute surgical problems] | | | | 1979 | | pubmed | title/abstract | irrelevant |
| 1449 | Marx RE | Bisphosphonate-induced exposed bone (osteonecrosis/osteopetrosis) of the jaws: risk factors, recognition, prevention, and treatment | | | | 2005 | | pubmed | title/abstract | irrelevant |
| 1450 | Johannsen FE | Corticosteroid injection is the best treatment in plantar fasciitis if combined with controlled training | | | | 2019 | | pubmed | title/abstract | irrelevant |
| 1451 | Arnold MJ | Injected Corticosteroids for Plantar Heel Pain | | | | 2018 | | pubmed | title/abstract | irrelevant |
| 1452 | Prieto de Paula JM | [Persistent neutrophilic meningitis and brain abscesses in a male patient with pulmonary sarcoidosis and corticosteroid therapy. Meningitis and cerebral abscesses due to Nocardia sp. Pulmonary sarcoidosis, steroid treatment] | | | | 2000 | | pubmed | title/abstract | irrelevant |
| 1453 | Cohen-Dolev N | Differences in Outcomes Over Time With Exclusive Enteral Nutrition Compared With Steroids in Children With Mild to Moderate Crohn's Disease: Results From the GROWTH CD Study | | | | 2018 | | pubmed | title/abstract | irrelevant |
| 1454 | Mpofu S | Steroids, non-steroidal anti-inflammatory drugs, and sigmoid diverticular abscess perforation in rheumatic conditions | | | | 2004 | | pubmed | duplicate | duplicate |
| 1455 | Cho ES | Primary reticulum cell sarcoma of the brain in a renal transplantation recipient | | | | 1974 | | pubmed | title/abstract | irrelevant |
| 1456 | Tageja N | Cervical spinal epidural abscess complicated with cerebral salt wasting | | | | 2009 | | pubmed | title/abstract | irrelevant |
| 1457 | Antoniou D | Osteomyelitis of the calcaneus and talus | | | | 1974 | | pubmed | title/abstract | irrelevant |
| 1458 | Hughes KL | Recent knowledge of the strict anaerobes of the gut | | | | 1972 | | pubmed | title/abstract | irrelevant |
| 1459 | Friedrich EG Jr | Evaluation and management of diseases of the vulva: therapeutic principles and techniques | | | | 1978 | | pubmed | title/abstract | irrelevant |
| 1460 | Lotter H | Testosterone increases susceptibility to amebic liver abscess in mice and mediates inhibition of IFNγ secretion in natural killer T cells | | | | 2013 | | pubmed | title/abstract | irrelevant |
| 1461 | Ngu BB | Eosinophilic granuloma of the atlas presenting as torticollis in a child | | | | 2004 | | pubmed | title/abstract | irrelevant |
| 1462 | Holm SE | Penetration of antibiotics into brain tissue and brain abscesses. An experimental study in steroid treated rats | | | | 1985 | | pubmed | title/abstract | irrelevant |
| 1463 | Campbell K | Non-steroidal anti-inflammatory drugs and complicated diverticular disease: a case-control study | | | | 1991 | | pubmed | title/abstract | irrelevant |
| 1464 | Wang X | The role of adjuvant systemic corticosteroid in pediatric retropharyngeal and parapharyngeal abscess | | | | 2024 | | pubmed | title/abstract | ineligible population |
| 1465 | Diamantes-Kepiotes A | [Clinical study on the effect of corticosteroid-antibiotic compound in the prevention and treatment of acute apical periodontitis] | | | | 1974 | | pubmed | title/abstract | irrelevant |
| 1466 | Olson PD | Androgen exposure potentiates formation of intratubular communities and renal abscesses by Escherichia coli | | | | 2018 | | pubmed | title/abstract | irrelevant |
| 1467 | Takashima Y | Detection of the initial site of Toxoplasma gondii reactivation in brain tissue | | | | 2008 | | pubmed | title/abstract | irrelevant |
| 1468 | Bresnan MJ | Bacterial meningitis. A symposium. IV. Neurological aspects: their diagnosis and treatment | | | | 1973 | | pubmed | title/abstract | irrelevant |
| 1469 | Nayeem MA | Immunogenicity of detergent membrane proteins of Entamoeba histolytica | | | | 1991 | | pubmed | title/abstract | irrelevant |
| 1470 | van den Broek RW | A new surgical complication related to corticosteroids in a patient with Henoch-Schönlein purpura | | | | 1995 | | pubmed | title/abstract | irrelevant |
| 1471 | Muehlbauer MA | Reticulocellsarcoma of the retroperitoneum, gastric perforation and steroids | | | | 1968 | | pubmed | title/abstract | irrelevant |
| 1472 | Lee WJ | Laparoscopic splenectomy for chronic idiopathic thrombocytopenic purpura | | | | 1997 | | pubmed | title/abstract | irrelevant |
| 1473 | Al Salman JM | Pituitary abscess | | | | 2017 | | pubmed | title/abstract | irrelevant |
| 1474 | Piroulas C | Non-steroids anti-inflammatory drugs and risk of peritonsillar abscess in pharyngitis: a French longitudinal study in primary care† | | | | 2019 | | pubmed | title/abstract | irrelevant |
| 1475 | Jensen KB | IgM turnover in man | | | | 1972 | | pubmed | title/abstract | irrelevant |
| 1476 | Cohen AS | Renal transplantation in two cases of amyloidosis | | | | 1971 | | pubmed | title/abstract | irrelevant |
| 1477 | Lucha PA Jr | The strictured anastomosis: successful treatment by corticosteroid injections--report of three cases and review of the literature | | | | 2005 | | pubmed | title/abstract | irrelevant |
| 1478 | Zouridaki E | Dermatological complications after bariatric surgery: report of two cases and review of the literature | | | | 2014 | | pubmed | title/abstract | irrelevant |
| 1479 | Gallardo D | Neurologic complications after allogeneic bone marrow transplantation | | | | 1996 | | pubmed | title/abstract | irrelevant |
| 1480 | Amir-Jahed AK | Thoracobilia: a surgical complication of hepatic echinococcosis and amebiasis | | | | 1972 | | pubmed | title/abstract | irrelevant |
| 1481 | Drobacheff C | [Buccal ulcers disclosing Wegener's disease. Therapeutic value of cyclosporin] | | | | 1990 | | pubmed | title/abstract | irrelevant |
| 1482 | Leckie WJ | Albumin turnover in pleural effusions | | | | 1965 | | pubmed | title/abstract | irrelevant |
| 1483 | Motohiro T | [Intractable bacterial infections in the nervous system] | | | | 1994 | | pubmed | title/abstract | irrelevant |
| 1484 | Loesche WJ | Assessing the relationship between dental disease and coronary heart disease in elderly U.S. veterans | | | | 1998 | | pubmed | title/abstract | irrelevant |
| 1485 | Busch MH | Spondylitis as a Rare Manifestation of Granulomatosis With Polyangiitis | | | | 2021 | | pubmed | title/abstract | irrelevant |
| 1486 | Palladini G | Daratumumab plus CyBorD for patients with newly diagnosed AL amyloidosis: safety run-in results of ANDROMEDA | | | | 2020 | | pubmed | title/abstract | irrelevant |
| 1487 | Moragrega B | [Disseminated tuberculosis with splenic abscesses during haemodialysis] | | | | 2010 | | pubmed | title/abstract | irrelevant |
| 1488 | Sousa EL | Quantification of endotoxins in infected root canals and acute apical abscess exudates: monitoring the effectiveness of root canal procedures in the reduction of endotoxins | | | | 2014 | | pubmed | duplicate | duplicate |
| 1489 | Lyu Y | Prognostic risk factors for pyogenic liver abscess caused by Klebsiella pneumoniae | | | | 2024 | | pubmed | title/abstract | irrelevant |
| 1490 | Kalantri SA | Thyroid abscess in case of Pre B acute lymphoblastic leukaemia: a rare presentation | | | | 2016 | | pubmed | title/abstract | irrelevant |
| 1491 | Mochizuki T | Spontaneous multiple insufficiency fractures after pelvic abscess and sepsis in a rheumatoid arthritis patient treated with high-load corticosteroid therapy: a case report | | | | 2007 | | pubmed | title/abstract | irrelevant |
| 1492 | Garas G | Prospective audit on the outpatient management of patients with a peritonsillar abscess: closing the loop: how we do it | | | | 2011 | | pubmed | title/abstract | irrelevant |
| 1493 | Escobar A | Multiple brain abscesses from isolated cerebral mucormycosis | | | | 1990 | | pubmed | title/abstract | irrelevant |
| 1494 | Ostrovskiĭ SE | [Infusion of drugs into the pulmonary artery in chronic suppurative diseases of the lungs] | | | | 1971 | | pubmed | title/abstract | irrelevant |
| 1495 | Wang L | Multiple hepatocellular adenomas associated with long-term administration of androgenic steroids for aplastic anemia: A case report and literature review | | | | 2020 | | pubmed | title/abstract | irrelevant |
| 1496 | Simsek I | Behçet's disease with a cecal perforation | | | | 2000 | | pubmed | title/abstract | irrelevant |
| 1497 | Lecuit M | [Intracerebral tuberculoma in HIV infection. Epidemiology and contribution of magnetic resonance imaging] | | | | 1994 | | pubmed | title/abstract | irrelevant |
| 1498 | Zenone T | Brain abscesses caused by Abiotrophia defectiva: complication of immunosuppressive therapy in a patient with connective-tissue disease | | | | 2004 | | pubmed | title/abstract | irrelevant |
| 1499 | Guyonnet JC | Relationship between the inhibitory activity on 'RCS' and prostaglandins synthesis and the anti-inflammatory activity of ketoprofen and several other non-steroidal anti-inflammatory agents | | | | 1976 | | pubmed | title/abstract | irrelevant |
| 1500 | Gibelin A | Lung abscess complicating pneumococcal pneumonia: a causal role of non-steroidal anti-inflammatory drugs? | | | | 2013 | | pubmed | title/abstract | irrelevant |
| 1501 | Bansal D | Altered lipid parameters in patients infected with Entamoeba histolytica, Entamoeba dispar and Giardia lamblia | | | | 2005 | | pubmed | title/abstract | irrelevant |
| 1502 | Tura S | Splenectomy in early chronic myeloid leukaemia: preliminary report on 37 cases | | | | 1974 | | pubmed | title/abstract | irrelevant |
| 1503 | Eiben RM | Acute brain swelling (toxic encephalopathy) | | | | 1967 | | pubmed | title/abstract | irrelevant |
| 1504 | Soriano Arandes A | [Subdural empyema: a complication of sinusitis. Report of 3 cases] | | | | 1998 | | pubmed | title/abstract | irrelevant |
| 1505 | Quintanar-Quintanar ME | Immunosuppressive treatment inhibits the development of amebic liver abscesses in hamsters | | | | 2004 | | pubmed | title/abstract | irrelevant |
| 1506 | Díaz-Llopis IV | Randomized controlled study of the efficacy of the injection of botulinum toxin type A versus corticosteroids in chronic plantar fasciitis: results at one and six months | | | | 2012 | | pubmed | duplicate | duplicate |
| 1507 | Croles FN | Splenic gas as a result of a non-Hodgkin's lymphoma in a patient with common variable immunodeficiency | | | | 2011 | | pubmed | title/abstract | irrelevant |
| 1508 | Seshadri N | Primary hepatic (extranodal) lymphoma: utility of [(18)F]fluorodeoxyglucose-PET/CT | | | | 2010 | | pubmed | title/abstract | irrelevant |
| 1509 | Kalinoski T | Case Report: A Case of Severe Cryptococcal Immune Reconstitution Inflammatory Syndrome Presenting with Brain and Intradural Abscesses in an HIV Patient | | | | 2020 | | pubmed | title/abstract | irrelevant |
| 1510 | Pircio AW | Pharmacology of a new non-steroidal anti-inflammatory agent 5-cyclohexylindan-1-carboxylic acid | | | | 1972 | | pubmed | title/abstract | irrelevant |
| 1511 | Eales L | Symptomatic porphyria in a case of Felty's syndrome. I. Clinical and routine biochemcial studies | | | | 1972 | | pubmed | title/abstract | irrelevant |
| 1512 | Kim SK | Bradyzoite-specific surface antigen SRS9 plays a role in maintaining Toxoplasma gondii persistence in the brain and in host control of parasite replication in the intestine | | | | 2007 | | pubmed | title/abstract | irrelevant |
| 1513 | Serrano-Luna J | Effect of phosphatidylcholine-cholesterol liposomes on Entamoeba histolytica virulence | | | | 2010 | | pubmed | title/abstract | irrelevant |
| 1514 | Whittaker GA | Effectiveness of Foot Orthoses Versus Corticosteroid Injection for Plantar Heel Pain: The SOOTHE Randomized Clinical Trial | | | | 2019 | | pubmed | title/abstract | irrelevant |
| 1515 | Snow DM | Lateral plantar nerve injury following steroid injection for plantar fasciitis | | | | 2005 | | pubmed | title/abstract | irrelevant |
| 1516 | Jung A | Intracerebral mass lesion diagnosed as cryptococcoma in a patient with sarcoidosis, a rare opportunistic manifestation induced by immunosuppression with corticosteroids | | | | 2012 | | pubmed | title/abstract | irrelevant |
| 1517 | Joss RA | Lung abscesses following corticosteroid therapy for central nervous system metastases | | | | 1981 | | pubmed | title/abstract | irrelevant |
| 1518 | Finkelstein FO | Risk factor analysis in renal transplantation: guidelines for the management of the transplant recipient | | | | 1974 | | pubmed | title/abstract | irrelevant |
| 1519 | Sánchez-Moreno P | Typhoid fever causing haemophagocytic lymphohistiocytosis in a non-endemic country - first case report and review of the current literature | | | | 2019 | | pubmed | title/abstract | irrelevant |
| 1520 | Moriya K | [Amebic liver abscesses developing during R-CHOP chemotherapy in a patient with mantle cell lymphoma] | | | | 2019 | | pubmed | title/abstract | irrelevant |
| 1521 | Voth D | Perioperative prevention of infection in neurosurgery | | | | 1985 | | pubmed | title/abstract | irrelevant |
| 1522 | Rascu A | Osteonecrosis in systemic lupus erythematosus, steroid-induced or a lupus-dependent manifestation? | | | | 1996 | | pubmed | title/abstract | irrelevant |
| 1523 | Manrique J | [Multiple complications after renal transplantation] | | | | 2004 | | pubmed | title/abstract | irrelevant |
| 1524 | Ferrante M | Corticosteroids but not infliximab increase short-term postoperative infectious complications in patients with ulcerative colitis | | | | 2009 | | pubmed | title/abstract | irrelevant |
| 1525 | Lueza JM | [Long-term corticosteroid therapy. Their impact on the oral environment] | | | | 1974 | | pubmed | title/abstract | irrelevant |
| 1526 | Rivero Fernández M | [Filgrastim in refractory Crohn's disease with an intra-abdominal abscess] | | | | 2007 | | pubmed | title/abstract | irrelevant |
| 1527 | Prévot S | [Pathology of the gallbladder and extra-hepatic bile ducts. Cases 2 and 3. Chronic cholecystitis] | | | | 2014 | | pubmed | title/abstract | irrelevant |
| 1528 | Kersten MJ | Unexpected neurologic complications following a novel lymphoma treatment 'expected' to give rise to neurologic toxicity | | | | 2019 | | pubmed | title/abstract | irrelevant |
| 1529 | Kumar H | Management of 2 teeth diagnosed with dens invaginatus with regenerative endodontics and apexification in the same patient: a case report and review | | | | 2014 | | pubmed | title/abstract | irrelevant |
| 1530 | Nieder C | Brain metastases in renal cell cancer: diagnostic and therapeutic aspects | | | | 2004 | | pubmed | title/abstract | irrelevant |
| 1531 | Morishita Y | Antithymocyte globulin for a patient with systemic lupus erythematosus complicated by severe pancytopenia | | | | 1997 | | pubmed | title/abstract | irrelevant |
| 1532 | Yasuda N | A case of Evans' syndrome complicated with multiple nocardial abscesses: a long-term survivor under corticosteroid therapy | | | | 2001 | | pubmed | title/abstract | irrelevant |
| 1533 | Kato T | [Toxoplasmic encephalitis and cytomegaloviral retinitis in a non-AIDS patient with chronic renal failure undergoing corticosteroid therapy] | | | | 2009 | | pubmed | title/abstract | irrelevant |
| 1534 | Christensen BJ | Is Postoperative Steroid Use Associated With Improved Outcomes in Severe Odontogenic Infections? | | | | 2025 | | pubmed | fulltext | included |
| 1535 | Cordero Ruiz P | Efficacy of adalimumab in patients with Crohn's disease and failure to infliximab therapy: a clinical series | | | | 2011 | | pubmed | title/abstract | irrelevant |
| 1536 | Van Slyck EJ | Pseudo-Chediak-Higashi anomaly in acute leukemia. A significant morphologic corollary? | | | | 1974 | | pubmed | title/abstract | irrelevant |
| 1537 | Blanco-Vidal MJ | [Left-sided hemiparesis in a diabetic patient: chronic granulomatous encephalitis due to Acanthamoeba] | | | | 2013 | | pubmed | title/abstract | irrelevant |
| 1538 | Urbina O | [The significance of antibiotic-hypersensitivity syndrome] | | | | 2011 | | pubmed | title/abstract | irrelevant |
| 1539 | Engle MA | Recent advances in the diagnosis and treatment of congenital heart disease | | | | 1977 | | pubmed | title/abstract | irrelevant |
| 1540 | Kurul S | Schilder's disease: case study with serial neuroimaging | | | | 2003 | | pubmed | title/abstract | irrelevant |
| 1541 | Tajima K | Multiple inflammatory pseudotumors of the liver associated with acute myeloblastic leukemia | | | | 1998 | | pubmed | title/abstract | irrelevant |
| 1542 | Arruda MA | Cluster headache in children and adolescents: ten years of follow-up in three pediatric cases | | | | 2011 | | pubmed | title/abstract | irrelevant |
| 1543 | Mathis RK | Liver disease in infants. Part II: hepatic disease states | | | | 1977 | | pubmed | title/abstract | irrelevant |
| 1544 | Del Forno A | Non-Hodgkin's lymphoma of the maxillary sinus in a patient with acquired immunodeficiency syndrome | | | | 1998 | | pubmed | title/abstract | irrelevant |
| 1545 | Kfir A | The diagnosis and conservative treatment of a complex type 3 dens invaginatus using cone beam computed tomography (CBCT) and 3D plastic models | | | | 2013 | | pubmed | title/abstract | irrelevant |
| 1546 | Desar IM | A Sézary cryptogram. Disseminated cryptococcal infection | | | | 2011 | | pubmed | title/abstract | irrelevant |
| 1547 | Boyce AM | Generalized Arterial Calcification of Infancy: New Insights, Controversies, and Approach to Management | | | | 2020 | | pubmed | title/abstract | irrelevant |
| 1548 | Rechner I | Systemic capillary leak syndrome after granulocyte colony-stimulating factor (G-CSF) | | | | 2003 | | pubmed | title/abstract | irrelevant |
| 1549 | Bersohn I | Liver-function tests in primary cancer of the liver in the Bantu | | | | 1969 | | pubmed | title/abstract | irrelevant |
| 1550 | Goubault P | Low-Phospholipid Associated Cholelithiasis (LPAC) syndrome: A synthetic review | | | | 2019 | | pubmed | title/abstract | irrelevant |
| 1551 | Salonen JH | Successful management of cerebral and pulmonary mucormycosis with liposomal amphotericin B in a 28-year-old woman with acute lymphoblastic leukemia | | | | 2006 | | pubmed | title/abstract | irrelevant |
| 1552 | Lalayanni C | Rituximab is effective for selected patients with chronic steroid-refractory immune thrombocytopenic purpura | | | | 2004 | | pubmed | title/abstract | irrelevant |
| 1553 | Wong GB | Primary extranodal B-cell non-Hodgkin lymphoma mimicking an endodontic lesion: report of 2 cases | | | | 2013 | | pubmed | title/abstract | irrelevant |
| 1554 | Basille D | Non-steroidal Anti-inflammatory Drugs may Worsen the Course of Community-Acquired Pneumonia: A Cohort Study | | | | 2017 | | pubmed | title/abstract | irrelevant |
| 1555 | Conterno G | [Antigens and antibodies of Streptococcus pyogenes in the laboratory and in clinical medicine. Clinico-experimental research on ASO, ASK, ASH and anti-M antibodies] | | | | 1967 | | pubmed | title/abstract | irrelevant |
| 1556 | Tayebi Khosroshahi H | Atypical clinical course of antineutrophil cytoplasmic autoantibodies-associated vasculitis | | | | 2013 | | pubmed | title/abstract | irrelevant |
| 1557 | Touahri T | Toxoplasmic encephalitis in a non-HIV patient with follicular lymphoma | | | | 2002 | | pubmed | title/abstract | irrelevant |
| 1558 | Daskivich TJ | Failure of gonadotropin-releasing hormone agonists with and without sterile abscess formation at depot sites: insight into mechanisms? | | | | 2006 | | pubmed | title/abstract | irrelevant |
| 1559 | Spinner CD | [Steroids in infection medicine] | | | | 2021 | | pubmed | title/abstract | irrelevant |
| 1560 | Legrand F | Adjuvant corticosteroid therapy for chronic disseminated candidiasis | | | | 2008 | | pubmed | title/abstract | irrelevant |
| 1561 | Flaherty GN | Deaths amongst asthmatics in Tasmania, 1964-1966 | | | | 1970 | | pubmed | title/abstract | irrelevant |
| 1562 | Di Carlo P | [Postnatal follow-up of infants born to mothers with certain Toxoplasma gondii infection: evaluation of prenatal management] | | | | 2005 | | pubmed | title/abstract | irrelevant |
| 1563 | J., Li, Jin | Orbital natural killer/T-cell lymphoma: a comprehensive case series and literature review | | | | 2025 | | Scopus | title/abstract | irrelevant |
| 1564 | A., Kais, A | Determinants of inpatient treatment in children with orbital cellulitis | | | | 2025 | | Scopus | title/abstract | irrelevant |
| 1565 | G., Chung, | Endogenous endophthalmitis due to Klebsiella pneumoniae liver abscess: a retrospective study of clinical course, treatment pattern, and prognosis | | | | 2025 | | Scopus | title/abstract | irrelevant |
| 1566 | B.J., Chris | Is Postoperative Steroid Use Associated With Improved Outcomes in Severe Odontogenic Infections? | | | | 2025 | | Scopus | duplicate | duplicate |
| 1567 | J.S., Lam, | Outcomes of surgical management in orbital cellulitis due to mucormycosis in patients recovered from COVID-19 | | | | 2025 | | Scopus | title/abstract | irrelevant |
| 1568 | M.A., O'Rou | Orbital Cellulitis Secondary to Dacryocystitis: A Case Series and Literature Review | | | | 2025 | | Scopus | title/abstract | irrelevant |
| 1569 | Y., Yang, Y | Granulomatous cheilitis after treatment of venous malformations of the upper lip: a case report and literature review | | | | 2025 | | Scopus | title/abstract | irrelevant |
| 1570 | C.S., Ho, C | Posterior scleritis mimicking orbital cellulitis in an early adolescent patient | | | | 2025 | | Scopus | title/abstract | irrelevant |
| 1571 | D.C., Mari, | Sinusitis al Dente | | | | 2025 | | Scopus | title/abstract | case report |
| 1572 | Z.E., McPhe | Evaluation of moderate periorbital cellulitis and home-based therapy in children (EPOCH study, Part 2): A prospective single centre cohort study | | | | 2025 | | Scopus | title/abstract | irrelevant |
| 1573 | T.W.X., Ang | Differentiation of bacterial orbital cellulitis and diffuse non-specific orbital inflammation on magnetic resonance imaging | | | | 2025 | | Scopus | title/abstract | irrelevant |
| 1574 | S., Riesche | Susceptibility to mycobacterial infection in VEXAS syndrome | | | | 2025 | | Scopus | title/abstract | irrelevant |
| 1575 | M.S., Alam, | Vision loss following orbital cellulitis complicated by acute dacryocystitis: Report of a rare case with review of literature | | | | 2025 | | Scopus | title/abstract | irrelevant |
| 1576 | P., Sriniva | Hansen’s disease mimicking orbital cellulitis | | | | 2025 | | Scopus | title/abstract | irrelevant |
| 1577 | F.M., Butle | Acute Rhinosinusitis: Rapid Evidence Review | | | | 2025 | | Scopus | title/abstract | irrelevant |
| 1578 | J.M., Ambat | A case of extraocular muscle pyomyositis in an elderly patient with diabetes mellitus | | | | 2025 | | Scopus | title/abstract | irrelevant |
| 1579 | C.Y., Lewis | Valsalva-associated orbital compartment syndrome in the setting of frontoethmoidal mucocele and orbital cellulitis | | | | 2025 | | Scopus | title/abstract | irrelevant |
| 1580 | F.A., Mukit | Penetrating colored pencil injury with Clostridium bifermentans pre-septal cellulitis: case report, literature review, and treatment algorithm | | | | 2025 | | Scopus | title/abstract | irrelevant |
| 1581 | A., Niknaha | Comparison of management and characteristics of orbital and preseptal cellulitis in adult patients with and without diabetes mellitus: a retrospective cohort study | | | | 2025 | | Scopus | title/abstract | irrelevant |
| 1582 | V.M., Chump | Role of Corticosteroids as Adjunctive Therapy in Patients With Odontogenic Cervicofacial Infections: Systematic Review | | | | 2025 | | Scopus | title/abstract | review |
| 1583 | H., Aya, Ha | A Case of Orbital Inflammation as the Initial Manifestation of Eosinophilic Granulomatosis with Polyangiitis Following Dupilumab administration, Successfully Treated with Mepolizumab | | | | 2025 | | Scopus | title/abstract | irrelevant |
| 1584 | D.D., Vasov | Orbital Cellulitis Following Stink Bug Toxin Exposure: A Unique Case Report | | | | 2025 | | Scopus | title/abstract | irrelevant |
| 1585 | P., Rujkora | Orbital Cellulitis Associated with Scleral Buckle Infection Caused by Macrolide-Resistant Mycobacterium abscessus Complex: A Case Report and Literature Review | | | | 2025 | | Scopus | title/abstract | irrelevant |
| 1586 | B.M., Wong, | Atypical pediatric orbital cellulitis with cavernous sinus thrombosis and petrous apicitis: a case report | | | | 2025 | | Scopus | title/abstract | irrelevant |
| 1587 | A., Abu-Sha | FROM ROUTINE TO RUIN: AN ASTONISHING COMPUTED TOMOGRAPHY SCAN REVEALS CATASTROPHIC LUDWIG’S ANGINA AND NECROTIZING MEDIASTINITIS AFTER A SIMPLE DENTAL PROCEDURE | | | | 2025 | | Scopus | title/abstract | irrelevant |
| 1588 | S.M., Rafiz | Toxic Orbital Cellulitis and Optic Neuropathy from High-Pressure Diesel Fuel Injury: A Case Report and Literature Review | | | | 2025 | | Scopus | title/abstract | irrelevant |
| 1589 | R., Timoumi | Necrotising cellulitis occurring after cosmetic blepharoplasty: A case report | | | | 2024 | | Scopus | title/abstract | irrelevant |
| 1590 | A.R., Daman | Use of a handheld ultrasound device for detecting orbital inflammation | | | | 2024 | | Scopus | title/abstract | irrelevant |
| 1591 | L.Y., Alsug | Subperiosteal abscess volume; an objective indication for surgical management in pediatrics | | | | 2024 | | Scopus | title/abstract | irrelevant |
| 1592 | S., Bloise, | Behind Suspected Orbital Cellulitis: Luc's Abscess | | | | 2024 | | Scopus | title/abstract | case report |
| 1593 | S., Allonen | Streptococcus intermedius causing primary bacterial ventriculitis in a patient with severe periodontitis - a case report | | | | 2024 | | Scopus | title/abstract | irrelevant |
| 1594 | M., Morrow, | Extensive Invasive Sinusitis Secondary to Streptococcus Intermedius Infection | | | | 2024 | | Scopus | title/abstract | irrelevant |
| 1595 | D., Tetik, | Orbital pseudocellulitis following systemic chemotherapy in a case of congenital retinoblastoma; Pseudocellulite orbitaire suite à une chimiothérapie systémique dans un cas de rétinoblastome congénital | | | | 2024 | | Scopus | title/abstract | irrelevant |
| 1596 | S., Agwan, | A vexing case of a 73-year-old man with fevers, orbital cellulitis, and asymptomatic interstitial lung disease | | | | 2024 | | Scopus | title/abstract | irrelevant |
| 1597 | A., Bhari, | Nasal natural killer/T-cell lymphoma mimicking orbital cellulitis: A diagnostic dilemma | | | | 2024 | | Scopus | title/abstract | irrelevant |
| 1598 | S.V., Kumar | Ocular dirofilariasis masquerading as orbital cellulitis: A case report and review of the literature | | | | 2024 | | Scopus | title/abstract | irrelevant |
| 1599 | S.K., Deka, | A STUDY OF THE PRESCRIBING PATTERN AND OUTCOME OF ANTIMICROBIALS IN INFECTIOUS EYE DISEASES IN A TERTIARY CARE HOSPITAL | | | | 2024 | | Scopus | title/abstract | irrelevant |
| 1600 | A., Chen, A | Imaging Features of Invasive Fungal Rhinosinusitis: A Systematic Review | | | | 2024 | | Scopus | title/abstract | irrelevant |
| 1601 | M.O., Boama | PERFORATED PEPTIC ULCER DISEASE IN CHRONIC NSAID USE: CASE OF A PATIENT WITH LONG-STANDING DENTAL INFECTION | | | | 2024 | | Scopus | title/abstract | irrelevant |
| 1602 | R.A., Pîrvu | Odontogenic Orbital Cellulitis at the Crossroads of Surgeries: Multidisciplinary Management and Review | | | | 2024 | | Scopus | title/abstract | case report |
| 1603 | P., Balakri | Management of avulsion-induced external root resorption of permanent maxillary left central and lateral incisors - a one-year follow-up case report | | | | 2024 | | Scopus | title/abstract | irrelevant |
| 1604 | A., Ambasth | Causes of Vision Loss Associated with Dengue Fever in Bihar, India – A Case Series | | | | 2024 | | Scopus | title/abstract | irrelevant |
| 1605 | S., Jawad, | Late-onset Pseudomonas aeruginosa orbital cellulitis following glaucoma drainage device implantation | | | | 2024 | | Scopus | title/abstract | irrelevant |
| 1606 | G.M., Desir | "Demodicosis" Mimicking PreSeptal Cellulitis: Severe Periocular and Facial Inflammation Caused by "Normal" Skin Flora | | | | 2024 | | Scopus | title/abstract | irrelevant |
| 1607 | A., Jannath | Maxillary mucormycosis masquerading as an endodontic-periodontal lesion | | | | 2024 | | Scopus | title/abstract | irrelevant |
| 1608 | C.L., Shiel | 2024 | Scopus | No | excluded | | title/abstract | |  |  |
| 1609 | J.E., Lu, J | Epidemiology of Orbital Inflammatory Disease: An AAO IRIS Registry Study | | | | 2024 | | Scopus | title/abstract | irrelevant |
| 1610 | H., Ali Hus | Rhino orbital cerebral mucormycosis: A life‑threatening complication of coronavirus diseases 2019 in an uncontrolled diabetic patient | | | | 2024 | | Scopus | title/abstract | irrelevant |
| 1611 | K.M., Clark | Combined upper lid skin crease and endoscopic approach to frontal sinus mucocoeles | | | | 2024 | | Scopus | title/abstract | irrelevant |
| 1612 | A., Kais, A | Role of Acute Rhinosinusitis in Periorbital Infections in Children | | | | 2024 | | Scopus | title/abstract | irrelevant |
| 1613 | M., Baba, M | A rare case of orbital inflammation complicated by hemophagocytic lymphohistiocytosis | | | | 2024 | | Scopus | title/abstract | irrelevant |
| 1614 | R.H., Malai | Orbital abscess with white eye in patients treated with oral antibiotics for orbital cellulitis | | | | 2024 | | Scopus | title/abstract | irrelevant |
| 1615 | N.E.H., Che | A rare type of primary CD19-negative diffuse large B-cell lymphoma presenting as an infraorbital mass in the maxillary sinus | | | | 2023 | | Scopus | title/abstract | irrelevant |
| 1616 | T., Xu-Yuan | A rare ocular complication of septicemia: a case series report and literature review | | | | 2023 | | Scopus | title/abstract | irrelevant |
| 1617 | A., Gómez M | Persistent socket pain in a dog after the enucleation of the eye and its clinical management | | | | 2023 | | Scopus | title/abstract | irrelevant |
| 1618 | J.B., Gueri | Infectious and Inflammatory Processes of the Orbits in Children | | | | 2023 | | Scopus | title/abstract | irrelevant |
| 1619 | X., Liu, Xi | Case Report: Vision Loss in a Child Caused by Streptococcus constellatus | | | | 2023 | | Scopus | title/abstract | irrelevant |
| 1620 | I.S., Dharm | Post Covid-19 Mucormycosis (Black fungus): A Cases Report | | | | 2023 | | Scopus | title/abstract | irrelevant |
| 1621 | O., Peleg, | Risk Factors for Postsurgical Infections in Facial Feminization Surgery | | | | 2023 | | Scopus | title/abstract | irrelevant |
| 1622 | R., Fu, Rox | Artificial Intelligence Automation of Proptosis Measurement: An Indicator for Pediatric Orbital Abscess Surgery | | | | 2023 | | Scopus | title/abstract | irrelevant |
| 1623 | A., Al-Jana | Bilateral, sequential orbital inflammation secondary to relapsing polychondritis | | | | 2023 | | Scopus | title/abstract | irrelevant |
| 1624 | G., Balchev | Rare Case of Bilateral Orbital Cellulitis in an Adult, Caused by Enterococcus faecalis | | | | 2023 | | Scopus | title/abstract | irrelevant |
| 1625 | A., Rangics | Management of Odontogenic Sinusitis: Results with Single-Step FESS and Dentoalveolar Surgery | | | | 2023 | | Scopus | title/abstract | irrelevant |
| 1626 | M.M., Carlà | Orbital cellulitis and massive chemosis as first sign of a cilio-choroidal malignant melanoma without extraocular extension: A case report | | | | 2023 | | Scopus | title/abstract | irrelevant |
| 1627 | D., Mire, D | Does Preoperative Surgical Delay Lead to Increased Postsurgical Length of Stay or Reoperation in Patients With Severe Odontogenic Infections? | | | | 2023 | | Scopus | title/abstract | irrelevant |
| 1628 | L., Wang, L | Ocular Phenotypes in Patients With Hemophagocytic Lymphohistiocytosis: A Retrospective Analysis in a Single Center Over 7 Years | | | | 2023 | | Scopus | title/abstract | irrelevant |
| 1629 | X., Chen, X | Central Retinal Artery Occlusion Due to Subperiosteal Orbital Abscess Caused by Acute Sinusitis in a Child: A Case Report | | | | 2023 | | Scopus | duplicate | duplicate |
| 1630 | C.J., Mears | Concurrent Pansinusitis and Orbital Cellulitis Complicated by Extensive Head and Neck Venous Thrombosis in an Unvaccinated Adolescent Patient with COVID-19: A Case Report | | | | 2023 | | Scopus | title/abstract | irrelevant |
| 1631 | A., Kais, A | Seasonal variations, acute rhinosinusitis and orbital infections in children | | | | 2023 | | Scopus | title/abstract | irrelevant |
| 1632 | N., Anton, | The Implications of SARS-CoV-2 Infection in a Series of Neuro-Ophthalmological Manifestations—Case Series and Literature Review | | | | 2023 | | Scopus | title/abstract | irrelevant |
| 1633 | H., Mahran, | Trends and Outcome of Aggressive Fascial Space Infections | | | | 2023 | | Scopus | title/abstract | irrelevant |
| 1634 | S.A., Alram | COVID-related mucormycosis mimicking dental infection | | | | 2023 | | Scopus | title/abstract | irrelevant |
| 1635 | J., Pelleti | High risk and low prevalence diseases: Orbital cellulitis | | | | 2023 | | Scopus | title/abstract | review |
| 1636 | A.J.K., Chu | Osteoradionecrosis of the lamina papyracea leading to recurrent orbital infections—A case study | | | | 2023 | | Scopus | title/abstract | irrelevant |
| 1637 | J., Vranckx | Atypic presentation of a Langerhans cell histiocytosis as an orbital mass; Présentation atypique d'une histiocytose de Langerhans sous forme d'une masse orbitaire | | | | 2023 | | Scopus | title/abstract | irrelevant |
| 1638 | E.L.V., Ngu | Variation in the Management of Hospitalized Children With Orbital Cellulitis Over 10 Years | | | | 2023 | | Scopus | fulltext | unclear |
| 1639 | V., Agarwal | Evaluation of risk factors, clinico-radiographic presentations of COVID-associated mucormycosis in the maxillofacial region reporting to a tertiary care dental facility | | | | 2023 | | Scopus | title/abstract | irrelevant |
| 1640 | M., Tbini, | A superior ophthalmic vein thrombosis following an acute pansinusitis: A case report | | | | 2023 | | Scopus | title/abstract | irrelevant |
| 1641 | S., Dolan, | Necrotising fasciitis of the head and neck: A case series from the West of Scotland | | | | 2023 | | Scopus | title/abstract | irrelevant |
| 1642 | O., Yalcinb | Different Cases, Different Manifestations of Post-COVID-19 Retinal Artery Occlusion: A Case Series | | | | 2023 | | Scopus | title/abstract | irrelevant |
| 1643 | M.A.P., Lus | Difficulty in management of acute invasive fungal rhinosinusitis in Indonesia during the COVID-19 pandemic: A case report | | | | 2023 | | Scopus | title/abstract | irrelevant |
| 1644 | Imen Mehri Turki | Clinical characteristics and management of odontogenic necrotizing fasciitis: a retrospective study | | | | 2023 | | Scopus | fulltext | unclear |
| 1645 | Y., Yanagaw | A Fatal Rhizopus Species Infection after Facial Injury in a Patient with Myelodysplastic Syndrome and Diabetes Mellitus | | | | 2023 | | Scopus | title/abstract | irrelevant |
| 1646 | M., Jari, M | Episcleritis; and posterior uveitis misdiagnosed as orbital cellulitis in a child patient with Behçet’s disease | | | | 2023 | | Scopus | title/abstract | irrelevant |
| 1647 | B., Gurnani | Successful management of delayed hyaluronidase hypersensitivity after subtenon's anesthesia during the COVID-19 pandemic: A rare case report | | | | 2023 | | Scopus | title/abstract | irrelevant |
| 1648 | J.E., Lu, J | The Role of Steroids for Pediatric Orbital Cellulitis–Review of the Controversy | | | | 2023 | | Scopus | title/abstract | review |
| 1649 | G., Davis, | Rapid Loss and Recovery of Vision Following Steroid Treatment in Orbital Myositis: A Case Report | | | | 2023 | | Scopus | title/abstract | irrelevant |
| 1650 | C.J., Hwang | A Case of the Blues - Colored Pencil Orbitopathy in an 18-Month-Old Boy | | | | 2023 | | Scopus | title/abstract | irrelevant |
| 1651 | U., Tungsat | Bilateral Orbital Inflammation Associated with COVID-19 Infection: A Case Report and Brief Review of the Literature | | | | 2023 | | Scopus | title/abstract | irrelevant |
| 1652 | R., Fu, Rox | Progression of Subperiosteal Orbital Abscess after Clinical Resolution on Intravenous Antibiotics and Steroids | | | | 2023 | | Scopus | title/abstract | case report |
| 1653 | E., Li | Orbital Subperiosteal Abscess: The Role of Nonsurgical Management Among Adolescents and Adults | | | | 2023 | | Scopus | fulltext | ineligible intervention |
| 1654 | A.S., Lawre | Orbital cellulitis and cavernous sinus thrombosis with contralateral sinus disease in a COVID-19 positive adolescent patient | | | | 2023 | | Scopus | title/abstract | irrelevant |
| 1655 | M.A., Eldes | A Proposed Diagnostic Algorithm for Fungal Orbital Infections after 20 Years of Experience in a Tertiary Eye Care Center– Egypt | | | | 2023 | | Scopus | title/abstract | irrelevant |
| 1656 | S., Prasad, | Rhino-orbital mucormycosis in a patient with no susceptibility following P.vivax malaria infection—a case report | | | | 2022 | | Scopus | title/abstract | irrelevant |
| 1657 | E., Gray, E | Did the March 2020 lockdown cause an increase in patients presenting to the emergency department with odontogenic pain and infection? A single centre, retrospective analysis | | | | 2022 | | Scopus | title/abstract | irrelevant |
| 1658 | R.P., Exley | Airway management, intensive care requirement, and corticosteroid use in cervicofacial infection. A Maxillofacial Trainee Research Collaborative (MTReC) study | | | | 2022 | | Scopus | fulltext | ineligible outcome |
| 1659 | N., Wang, N | Long-term efficacy of enucleation combined with primary orbital implantation in children with retinoblastoma histopathological invasion of optic nerve | | | | 2022 | | Scopus | title/abstract | irrelevant |
| 1660 | E., Shih, E | Antibiotic Choices for Pediatric Periorbital Cellulitis—A 20-Year Retrospective Study from Taiwan | | | | 2022 | | Scopus | title/abstract | irrelevant |
| 1661 | S., Madurei | Ribavirin for severe acute hepatitis E virus infection | | | | 2022 | | Scopus | title/abstract | irrelevant |
| 1662 | M., Mekni, | Orbital cellulitis: diagnostic, therapeutic and prognostic approach in a reference center in Tunis, Tunisia (a retrospective study of 109 cases); Les cellulites orbitaires: approche diagnostique, thérapeutique et pronostique dans un centre de référence à Tunis, Tunisie (une étude rétrospective sur 109 cas) | | | | 2022 | | Scopus | title/abstract | irrelevant |
| 1663 | G., Cantare | Misdirection of a nasopharyngeal SARS-CoV-2 swab: An unexpected complication | | | | 2022 | | Scopus | title/abstract | irrelevant |
| 1664 | P.E.V.N., B | A Rare Case of Orbital Cellulitis with Tolosa-Hunt Syndrome Caused by Methicillin-Resistant Staphylococcus aureus (MRSA): a Case Report | | | | 2022 | | Scopus | title/abstract | irrelevant |
| 1665 | B., Koirala | Orbital apex syndrome secondary to myocysticercosis: A case report from Nepal | | | | 2022 | | Scopus | title/abstract | irrelevant |
| 1666 | S., Sasmant | Odontogenic brain abscess due to Anaerococcus prevotii infections: A case report and review article | | | | 2022 | | Scopus | title/abstract | irrelevant |
| 1667 | S., Guo, Sa | Misdiagnosis of Orbital Foreign Body as Glaucoma Drainage Device | | | | 2022 | | Scopus | title/abstract | irrelevant |
| 1668 | H., Lee, Ho | The first Aotearoa New Zealand case of NUDT15-variant-related thiopurine-induced myelotoxicity | | | | 2022 | | Scopus | title/abstract | irrelevant |
| 1669 | B.I., Anosi | Epidemiology and Management of Orbital Cellulitis in Children | | | | 2022 | | Scopus | fulltext | unclear |
| 1670 | C.D., Sisk, | Monocular Vision Loss Subsequent to Complicated Orbital Cellulitis | | | | 2022 | | Scopus | title/abstract | irrelevant |
| 1671 | S.M., Coall | Clinical, advanced imaging data and outcome of inflammatory and neoplastic orbital disease in 81 dogs and 16 cats in Australia (2010–2019) | | | | 2022 | | Scopus | title/abstract | irrelevant |
| 1672 | A., Hári-Ko | Orbital Cellulitis following COVID-19 Vaccination | | | | 2022 | | Scopus | title/abstract | case report |
| 1673 | B., Kim, Bo | Role of systemic corticosteroids in orbital cellulitis: a meta-analysis and literature review | | | | 2022 | | Scopus | title/abstract | review |
| 1674 | D., Bueno-S | Acute Dyspnea and Hemoptysis in an 84-Year-Old Man With Multiple Comorbidities | | | | 2022 | | Scopus | title/abstract | irrelevant |
| 1675 | W., Lahmini | Management of periorbital cellulitis at the Pediatric Emergency Department: A ten years study; Prise en charge de la cellulite périorbitaire aux Urgences Pédiatriques : étude de dix ans | | | | 2022 | | Scopus | fulltext | ineligible outcome |
| 1676 | B., Yu, Bo; | Immediate Endoscopic Dacryocystorhinostomy in Patients With New Onset Acute Dacryocystitis | | | | 2022 | | Scopus | title/abstract | irrelevant |
| 1677 | D., Sarkar, | Case of leprosy mimicking preseptal cellulitis: A diagnostic dilemma | | | | 2022 | | Scopus | title/abstract | irrelevant |
| 1678 | W.A., Naqvi | Acute Rhino-orbital-cerebral Mucormycosis in a Patient with COVID – 19 | | | | 2022 | | Scopus | title/abstract | irrelevant |
| 1679 | S.W., Hamed | Orbital Infections | | | | 2022 | | Scopus | title/abstract | unclear |
| 1680 | D., Tadros, | Orbital Complications of Acute Invasive Fungal Rhinosinusitis: A New Challenge in the COVID-19 Convalescent Patients | | | | 2022 | | Scopus | title/abstract | irrelevant |
| 1681 | Y., Rawate, | Analysis of ophthalmic manifestations of invasive rhino-orbito-cerebral mucormycosis in COVID-19 patients in a medical college | | | | 2022 | | Scopus | title/abstract | irrelevant |
| 1682 | H.L., Letso | A case of severe endogenous endophthalmitis with orbital cellulitis post COVID-19 | | | | 2022 | | Scopus | title/abstract | irrelevant |
| 1683 | P.J., Gill, | Association Between Corticosteroids and Outcomes in Children Hospitalized With Orbital Cellulitis | | | | 2022 | | Scopus | fulltext | included |
| 1684 | M., Mardani | Dexamethasone Induced Sino-Orbital Mucormycosis In a Patient Infected With COVID-19 | | | | 2022 | | Scopus | title/abstract | irrelevant |
| 1685 | M.Z., Salta | Orbital Complications of Acute Sinusitis in Pediatric Patients: Management of Chandler III Patients | | | | 2022 | | Scopus | title/abstract | irrelevant |
| 1686 | A., Kovacov | Eye problems in people with diabetes: more than just diabetic retinopathy | | | | 2022 | | Scopus | title/abstract | irrelevant |
| 1687 | A., Ghanoun | Occult Colon Adenocarcinoma and Multiple Myeloma Associated with Clostridium septicum Panophthalmitis with Orbital and Chiasmal Extension: A Case Report | | | | 2022 | | Scopus | title/abstract | irrelevant |
| 1688 | J.A., Barne | Parvimonas micra Necrotizing Panophthalmitis Involving the Sclera, Cornea, Uvea, Retina, and Orbit | | | | 2022 | | Scopus | title/abstract | irrelevant |
| 1689 | I., Rotstei | Prevalence of periapical abscesses in patients with systemic lupus erythematosus | | | | 2022 | | Scopus | title/abstract | irrelevant |
| 1690 | V.A., Chand | A Rare Fungal Orbital Infection in an Immunocompetent Young Male Caused by Lichtheimia corymbifera (Absidia corymbifera) | | | | 2022 | | Scopus | title/abstract | irrelevant |
| 1691 | M.K., Rao, | Fungal abscess after intra-orbital steroid injection: a case report | | | | 2022 | | Scopus | duplicate | duplicate |
| 1692 | R.L., Zamor | Severe complications from infectious mononucleosis after prolonged steroid therapy | | | | 2021 | | Scopus | title/abstract | irrelevant |
| 1693 | S., Nayak, | Sight-threatening intraocular infection in patients with COVID-19 in India | | | | 2021 | | Scopus | title/abstract | irrelevant |
| 1694 | M.F.S.O., A | Combined endophthalmitis and orbital cellulitis in patients with corona virus disease (COVID-19) | | | | 2021 | | Scopus | title/abstract | irrelevant |
| 1695 | E.H., Theng | Periorbital inflammation associated with craniofacial fibrous dysplasia: Report of three cases and review of the literature | | | | 2021 | | Scopus | title/abstract | irrelevant |
| 1696 | T.M., Roush | A case series of post COVID-19 mucormycosis—a neurological prospective | | | | 2021 | | Scopus | title/abstract | irrelevant |
| 1697 | M.A., Leszc | Corticosteroids for acute orbital cellulitis | | | | 2021 | | Scopus | duplicate | duplicate |
| 1698 | F.P., Hudso | Acute chagas disease manifesting as orbital cellulitis, texas, usa | | | | 2021 | | Scopus | title/abstract | case report |
| 1699 | J., Miranda | Preseptal Versus Orbital Cellulitis in Children: An Observational Study | | | | 2021 | | Scopus | title/abstract | irrelevant |
| 1700 | N., Ben Abd | Pseudomonas aeruginosa orbital cellulitis complicated by ophthalmic artery occlusion in an immunocompetent patient: A case report | | | | 2021 | | Scopus | title/abstract | irrelevant |
| 1701 | C., Savard, | Extraction of aberrant tusk and mandibular osteomyelitis treatment in a pot-bellied pig (Sus scrofa domesticus) | | | | 2021 | | Scopus | title/abstract | irrelevant |
| 1702 | P., Sinha, | Bilateral endogenous endophthalmitis complicated by scleral perforation: an unusual presentation | | | | 2021 | | Scopus | title/abstract | irrelevant |
| 1703 | K.A., Derwi | A PEDIATRIC CASE OF ORBITAL CELLULITIS WITH PANSINUSITIS AND SUBPERIOSTEAL ABSCESS | | | | 2021 | | Scopus | title/abstract | case report |
| 1704 | S.J.W., Ken | The use of steroids in cervicofacial infections in UK OMFS departments: a Maxillofacial Surgery Trainees Research Collaborative (MTReC) study | | | | 2021 | | Scopus | duplicate | duplicate |
| 1705 | A., Mehta, | Aseptic orbital cellulitis: A master masquerade of intraocular malignancy | | | | 2021 | | Scopus | title/abstract | case report |
| 1706 | D.S., AlQah | A child with refractory orbital cellulitis after water pipe smoking | | | | 2021 | | Scopus | title/abstract | case report |
| 1707 | H., Park, H | Lacrimal Gland Ductal Cyst Infection Presenting as Acute Orbital Cellulitis with Abscess | | | | 2021 | | Scopus | title/abstract | case report |
| 1708 | N., Bayram, | Susceptibility of severe COVID-19 patients to rhino-orbital mucormycosis fungal infection in different clinical manifestations | | | | 2021 | | Scopus | title/abstract | irrelevant |
| 1709 | S., Mahalin | The role of adjuvant systemic steroids in the management of periorbital cellulitis secondary to sinusitis: a systematic review and meta-analysis | | | | 2021 | | Scopus | title/abstract | review |
| 1710 | C.B., Krueg | Changes in the management of severe orbital infections over seventeen years | | | | 2021 | | Scopus | fulltext | unclear |
| 1711 | S.A., Ravan | Rise of the phoenix: Mucormycosis in COVID-19 times | | | | 2021 | | Scopus | title/abstract | irrelevant |
| 1712 | S., Lalwani | Oculomotor synkinesis: An uncommon sequela of paediatric cavernous sinus thrombosis | | | | 2021 | | Scopus | title/abstract | irrelevant |
| 1713 | A., van Oor | Case report of severe acute eosinophilic pneumonia induced by amoxicillin; Une forme sévère de pneumopathie aiguë à éosinophiles liée à l'amoxicilline | | | | 2021 | | Scopus | title/abstract | irrelevant |
| 1714 | A., Kahana, | Orbital inflammatory disorders: New knowledge, future challenges | | | | 2021 | | Scopus | title/abstract | review |
| 1715 | E.A., Korne | Corticosteroids for periorbital and orbital cellulitis | | | | 2021 | | Scopus | duplicate | duplicate |
| 1716 | T., Jiamjun | Rare manifestation of ocular immune reconstitution inflammatory syndrome from mycobacterium scrofulaceum infection in a patient with AIDS | | | | 2021 | | Scopus | title/abstract | irrelevant |
| 1717 | M.R., Jaisa | Tension orbit secondary to a carious primary molar—A case report | | | | 2021 | | Scopus | title/abstract | irrelevant |
| 1718 | G.P., Bauma | The Effects of Dexamethasone on the Time to Pain Resolution in Dental Periapical Abscess | | | | 2021 | | Scopus | duplicate | duplicate |
| 1719 | N., Pham Da | Severe odontogenic infections drastically dropped during the COVID19-confinement: because hospitals became sanctuaries or because of the massive interruption in the consumption of NSAIDs? | | | | 2021 | | Scopus | title/abstract | irrelevant |
| 1720 | R.E., Johns | Odontogenic Infections: Disease Burden During COVID-19 at a Single Institution | | | | 2021 | | Scopus | title/abstract | irrelevant |
| 1721 | K., Chauhan | Silicone oil-associated orbital cellulitis with lipogranulomatous inflammation in the setting of HIV: A management challenge and clinicopathological correlation | | | | 2021 | | Scopus | title/abstract | irrelevant |
| 1722 | B., Mathews | Ocular Syphilis Causing Panophthalmitis | | | | 2021 | | Scopus | title/abstract | case report |
| 1723 | Y., Cao, Yu | Diagnosis and treatment of 11 cases of subperiosteal orbital abscess caused by acute sinusitis; 11 例急性鼻窦炎并发眶骨膜下脓肿的诊治分析 | | | | 2021 | | Scopus | title/abstract | unclear |
| 1724 | K.K., Ramin | Post COVID-19 Rhino-Orbital-Cerebral Mucormycosis: The Guitar Pick Sign; COVID-19 Sonrası Rino-Orbito-Serebral Mukormikoz: Gitar Penası İşareti | | | | 2021 | | Scopus | title/abstract | irrelevant |
| 1725 | Y.Z., Demir | Two case of Rhino-Orbito-Cerebral Mucormicosis developed after COVID-19 infection; COVID-19 enfeksiyonu sonrası gelişen ıki Rino-Orbito-Serebral Mukormikoz olgusu | | | | 2021 | | Scopus | title/abstract | irrelevant |
| 1726 | S., Bouhout | A rare presentation of Klebsiella pneumoniae endogenous panophthalmitis with optic neuritis and orbital cellulitis from a urinary tract infection | | | | 2021 | | Scopus | title/abstract | irrelevant |
| 1727 | G.C., Chasc | Adult gonococcal conjunctivitis: Prevalence, clinical features and complications | | | | 2021 | | Scopus | title/abstract | irrelevant |
| 1728 | M.I., Alkha | Superior oblique myositis mimics a subperiosteal abscess in a patient with sinusitis; Miosite oblíqua superior mimetiza abscesso subperiosteal em paciente com sinusite | | | | 2021 | | Scopus | title/abstract | irrelevant |
| 1729 | D., Yıldız, | Unilateral pseudotumor orbita in a 13-year-old boy; On üç yaşında bir çocukta unilateral psödotümör orbita | | | | 2021 | | Scopus | title/abstract | irrelevant |
| 1730 | Y., Irawati | Orbital compressed air and diesel explosion injury resembling orbital cellulitis: An unusual case | | | | 2021 | | Scopus | title/abstract | irrelevant |
| 1731 | C., Wern-Yi | Bilateral cavernous sinus and left dural sigmoid sinus thrombosis associated with extreme exertion: a case report; Trombose bilateral do seio cavernoso e do seio sigmoide esquerdo associada a exercício extremo: relato de caso | | | | 2021 | | Scopus | title/abstract | irrelevant |
| 1732 | C., Delbet- | Does anti-inflammatory drugs modify the severe odontogenic infection prognosis? A 10-year’s experience | | | | 2021 | | Scopus | fulltext | included |
| 1733 | A.J., Chen, | A case of mistaken identity: Saksenaea vasiformis of the orbit | | | | 2021 | | Scopus | title/abstract | irrelevant |
| 1734 | Brian J Christensen | Risk Factors for Reoperation in Patients Hospitalized for Odontogenic Infections | | | | 2025 | | Scopus | fulltext | irrelevant |
| 1735 | P., Chandra | Multifocal extraocular muscle pyomyositis: A case report and review of literature | | | | 2021 | | Scopus | title/abstract | irrelevant |
| 1736 | O., Nanegru | Posterior Scleritis Mimicking Orbital Cellulitis: A Report of the Three Cases | | | | 2020 | | Scopus | title/abstract | irrelevant |
| 1737 | E.A.W.F., H | Idiopathic TTP in the Middle East: Epidemiology and clinical outcomes in infection associated episodes | | | | 2020 | | Scopus | title/abstract | irrelevant |
| 1738 | M.R., Khali | Invasive Fungal Keratitis as an Uncommon Form of Mucormycosis Leading to Endophthalmitis: Report of Two Cases and Literature Review | | | | 2020 | | Scopus | title/abstract | irrelevant |
| 1739 | L., Fang, L | Auricular suppurative perichondritis secondary to exclusive endoscopic ear surgery for tympanoplasty: A case report and literature review | | | | 2020 | | Scopus | title/abstract | irrelevant |
| 1740 | K.B.H., Vo, | Two cases of epidemic keratoconjunctivitis-associated dacryocystitis | | | | 2020 | | Scopus | title/abstract | irrelevant |
| 1741 | I., Staten, | Periapical abscess progressing to parotitis and descending necrotizing mediastinitis with thoracic abscess in a patient on etanercept: A case report | | | | 2020 | | Scopus | title/abstract | irrelevant |
| 1742 | J., Buffaul | Is the Xen® Gel Stent really minimally invasive? | | | | 2020 | | Scopus | title/abstract | irrelevant |
| 1743 | J., McCarth | Anaesthesia for maxillofacial surgery | | | | 2020 | | Scopus | duplicate | duplicate |
| 1744 | W., Song, W | A case report of exudative retinal detachment derived from orbital cellulitis in mainland China | | | | 2020 | | Scopus | title/abstract | irrelevant |
| 1745 | A., Tami, A | Ludwig's angina and steroid use: A narrative review | | | | 2020 | | Scopus | title/abstract | review |
| 1746 | T., Miura, | A case of chronic sinusitis with an orbital subperiosteal abscess requiring surgical drainage; 慢性副鼻腔炎急性増悪後に眼窩骨膜下膿瘍をきたし外科的ドレナージを必要とした 1 例 | | | | 2020 | | Scopus | title/abstract | irrelevant |
| 1747 | M.E., Corre | Fulminating Orbital Cellulitis after Uncomplicated Cataract Surgery in an Immunocompromised Patient with Secondary Endophthalmitis | | | | 2020 | | Scopus | title/abstract | irrelevant |
| 1748 | R., Moghadd | Presumed Primary Bacterial Rhinosinusitis-Associated Optic Neuritis in a Cat | | | | 2020 | | Scopus | title/abstract | irrelevant |
| 1749 | R.A., Denu, | MRSA septicemia with septic arthritis and prostatic, intraretinal, periapical, and lung abscesses | | | | 2020 | | Scopus | title/abstract | irrelevant |
| 1750 | K.A., Sadle | Very unusual case of a primary sinonasal germ cell tumour | | | | 2020 | | Scopus | title/abstract | irrelevant |
| 1751 | V., Chandra | Concomitant parasagittal meningioma and adjacent intracranial abscess of occult etiology | | | | 2020 | | Scopus | title/abstract | irrelevant |
| 1752 | L.M., Hassm | Herpetic Panophthalmitis: A Diagnostic Dilemma | | | | 2020 | | Scopus | title/abstract | irrelevant |
| 1753 | M.M., Al-Mo | Sporadic form of isolated orbital Burkitt lymphoma presenting with fulminant proptosis and dystopia | | | | 2020 | | Scopus | title/abstract | irrelevant |
| 1754 | P.K., Kavia | Exigency of ocular complications of systemic lupus erythematosus | | | | 2020 | | Scopus | title/abstract | irrelevant |
| 1755 | J.N., Jaban | Management of a large intraorbital wooden foreign body: Case report | | | | 2020 | | Scopus | title/abstract | irrelevant |
| 1756 | Y., Yamana, | A case of idiopathic orbital inflammation with posterior scleritis simulating orbital cellulitis | | | | 2020 | | Scopus | title/abstract | case report |
| 1757 | J., Geusens | Subperiosteal orbital abscess from odontogenic origin: A case report | | | | 2020 | | Scopus | title/abstract | case report |
| 1758 | A.G., Nair, | Simultaneous bilateral orbital cellulitis with meningitis caused by methicillin-resistant staphylococcus aureus in an immunocompetent infant | | | | 2020 | | Scopus | title/abstract | case report |
| 1759 | F.V., Santo | Deep neck space infection and Lemierre's syndrome caused by Streptococcus anginosus: A case report | | | | 2020 | | Scopus | title/abstract | case report |
| 1760 | K., Jung, K | Multiple brain abscesses treated by extraction of the maxillary molars with chronic apical lesion to remove the source of infection | | | | 2019 | | Scopus | title/abstract | irrelevant |
| 1761 | M.S., de Al | Treatment of an Acute Apical Abscess in a Patient With Autoimmune Hepatitis Taking Alendronate: A Case Report | | | | 2019 | | Scopus | title/abstract | irrelevant |
| 1762 | D.J., Danek | Corneal perforation with uveal prolapse: An initial presentation of orbital metastatic breast cancer | | | | 2019 | | Scopus | title/abstract | irrelevant |
| 1763 | M.P., Breaz | Endogenous Serratia marcescens panophthalmitis: A case series | | | | 2019 | | Scopus | title/abstract | irrelevant |
| 1764 | D., Ghosh, | Antibiotic use in dental practice: A review | | | | 2019 | | Scopus | title/abstract | irrelevant |
| 1765 | G., de Frei | Perinephric Abscess and Bacteremia Due to Lactobacillus Species in a Diabetic Adult: An Uncommon Presentation | | | | 2019 | | Scopus | title/abstract | irrelevant |
| 1766 | D., Somsen, | Rapid onset of orbital cellulitis after uncomplicated strabismus surgery | | | | 2019 | | Scopus | title/abstract | irrelevant |
| 1767 | R.R., Alana | Scleral buckle induce orbital cellulitis and scleritis – A case report and literature review | | | | 2019 | | Scopus | title/abstract | case report |
| 1768 | M., Singh, | Long-term ophthalmic outcomes in pediatric orbital cellulitis: A prospective, multidisciplinary study from a tertiary-care referral institute | | | | 2019 | | Scopus | title/abstract | case report |
| 1769 | K.J., Willi | Paediatric orbital and periorbital infections | | | | 2019 | | Scopus | title/abstract | review |
| 1770 | M.T., Ally, | Mucormycosis: More Than Meets the Eye! | | | | 2019 | | Scopus | title/abstract | irrelevant |
| 1771 | B., Mirzash | The role of poor oral health in surgical site infection following elective spinal surgery | | | | 2019 | | Scopus | title/abstract | irrelevant |
| 1772 | L., Zhu, Li | Clinical analysis and antidiastole of eye disorders which manifest as acute orbital inflammation; 眼眶急性炎症的病例分析及鉴别诊断 | | | | 2019 | | Scopus | title/abstract | unclear |
| 1773 | V.H., Vasan | Choroidal Melanoma with Optic Nerve Infiltration Presenting as Orbital Pseudocellulitis | | | | 2019 | | Scopus | title/abstract | irrelevant |
| 1774 | N.A., van d | Impact of superior ophthalmic vein thrombosis: a case series and literature review | | | | 2019 | | Scopus | title/abstract | irrelevant |
| 1775 | J.C., Santo | Pediatric preseptal and orbital cellulitis: A 10-year experience | | | | 2019 | | Scopus | fulltext | ineligible outcome |
| 1776 | B., Alqasee | Orbital cellulitis following preseptal cellulitis | | | | 2019 | | Scopus | title/abstract | case report |
| 1777 | D., McKenna | Pediatric intraorbital abscess: Early recognition and management | | | | 2019 | | Scopus | title/abstract | case report |
| 1778 | E., Ortiz-L | An Update on the Treatment and Management of Cellulitis; Actualización en el abordaje y manejo de celulitis | | | | 2019 | | Scopus | title/abstract | irrelevant |
| 1779 | A., Raman, | Histopathologic Features of Chronic Sinusitis Precipitated by Odontogenic Infection | | | | 2019 | | Scopus | title/abstract | irrelevant |
| 1780 | A., Das, An | Simultaneous diagnosis of unilateral retinoblastoma and contralateral optic pathway glioma in a child with neurofibromatosis type 1 | | | | 2019 | | Scopus | title/abstract | irrelevant |
| 1781 | R.G., Spini | Recurrent orbital complication by rhinosinusitis. A case report; Complicación orbitaria recurrente por rinosinusitis. Reporte de un caso | | | | 2019 | | Scopus | title/abstract | case report |
| 1782 | S.P., Amir, | Orbital cellulitis clinically mimicking rhabdomyosarcoma | | | | 2019 | | Scopus | title/abstract | case report |
| 1783 | M.R., Ing, | Extraocular and intraocular infections following strabismus surgery: A review | | | | 2019 | | Scopus | title/abstract | review |
| 1784 | H., Sotoude | Superior ophthalmic vein thrombosis: What radiologist and clinician must know? | | | | 2019 | | Scopus | title/abstract | irrelevant |
| 1785 | M., Huh, Mi | A case of acute dacryocystitis diagnosed after surgical treatment of orbital cellulitis and orbital abscess | | | | 2019 | | Scopus | title/abstract | irrelevant |
| 1786 | G.J., Shaug | Absorbable Implant Foreign Body Reaction Masquerading as Orbital Cellulitis | | | | 2019 | | Scopus | title/abstract | irrelevant |
| 1787 | J.W., Goldf | A Case of Pseudomonas Orbital Cellulitis Following Glaucoma Device Implantation | | | | 2019 | | Scopus | title/abstract | irrelevant |
| 1788 | D.H., AlHar | Infected conjunctival pyogenic granuloma at strabismus surgery site mimicking conjunctival abscess | | | | 2019 | | Scopus | title/abstract | irrelevant |
| 1789 | P., Singh, | Choroidal melanoma masquerading as orbital cellulitis | | | | 2018 | | Scopus | title/abstract | irrelevant |
| 1790 | J.M., Kim, | Orbital cellulitis with choroidal detachment following strabismus surgery in an adult | | | | 2018 | | Scopus | title/abstract | irrelevant |
| 1791 | A., Brameli | Systemic corticosteroids may be beneficial for managing severe or refractory orbital cellulitis in children | | | | 2018 | | Scopus | duplicate | duplicate |
| 1792 | J., Juri Ma | Superior Ophthalmic Vein Thrombosis with Complete Loss of Vision as a Complication of Autoimmune and Infective Conditions | | | | 2018 | | Scopus | title/abstract | irrelevant |
| 1793 | W.V., Lin, | Scleritis, keratitis, and orbital cellulitis: isolated ocular manifestation of systemic lupus erythematosus | | | | 2018 | | Scopus | title/abstract | irrelevant |
| 1794 | M., Albert- | A case report of orbital Langerhans cell histiocytosis presenting as a orbital cellulitis; Caso clínico de histiocitosis de células de Langerhans presentándose como una celulitis orbitaria | | | | 2018 | | Scopus | title/abstract | case report |
| 1795 | C.E., Foste | Molecular Characterization of Staphylococcus aureus Isolates from Children with Periorbital or Orbital Cellulitis | | | | 2018 | | Scopus | fulltext | included |
| 1796 | D.A., Kline | Successful Management of Descending Necrotizing Mediastinitis with Minimally Invasive Mediastinal Interventions | | | | 2018 | | Scopus | title/abstract | irrelevant |
| 1797 | A., Shen, A | A 12-month-old boywith bilateral facial swelling and proptosis | | | | 2018 | | Scopus | title/abstract | case report |
| 1798 | D.S., Churg | Multi-drug resistant Mycobacterium chelonae scleral buckle infection | | | | 2018 | | Scopus | title/abstract | irrelevant |
| 1799 | N., Tananuv | Ocular Basidiobolomycosis: A Case Report | | | | 2018 | | Scopus | title/abstract | irrelevant |
| 1800 | L., Chen, L | Intravenous Steroids With Antibiotics on Admission for Children With Orbital Cellulitis | | | | 2018 | | Scopus | fulltext | included |
| 1801 | A., Lecler, | Infraorbital Nerve Involvement on Magnetic Resonance Imaging in Igg4-Related Ophthalmic Disease: A Highly Suggestive Sign | | | | 2018 | | Scopus | title/abstract | irrelevant |
| 1802 | N.D., Raich | Hyaluronidase allergy mimicking orbital cellulitis | | | | 2018 | | Scopus | title/abstract | irrelevant |
| 1803 | M.M., Amir, | Caroticocavernous Fistula: Successful Reversal by Endovascular Treatment | | | | 2018 | | Scopus | title/abstract | irrelevant |
| 1804 | F., Pakdel, | Spontaneous rupture of lacrimal gland pleomorphic adenoma: Pivotal role in masquerading orbital cellulitis | | | | 2018 | | Scopus | title/abstract | irrelevant |
| 1805 | J.L., Markh | Variation in care and clinical outcomes in children hospitalized with orbital cellulitis | | | | 2018 | | Scopus | fulltext | ineligible intervention |
| 1806 | G.E.L., Amr | Extra-nodal NK/T-cell lymphoma, nasal type presenting as orbital cellulitis; Un lymphome T/NK extra-ganglionnaire de type nasal se présentant comme une cellulite orbitaire | | | | 2018 | | Scopus | title/abstract | irrelevant |
| 1807 | A.K., Alsal | Acute dacryocystitis complicated by orbital cellulitis and loss of vision: A case report and review of the literature | | | | 2018 | | Scopus | title/abstract | irrelevant |
| 1808 | E., Wates, | A severe deep neck odontogenic infection not prioritised by the emergency department triage system and National Early Warning Score | | | | 2018 | | Scopus | title/abstract | case report |
| 1809 | R.A.R., AlH | A brain populated with space-occupying lesions: Identifying the culprit | | | | 2018 | | Scopus | title/abstract | irrelevant |
| 1810 | S., Galeb, | Orbital Infections: Current Standards and Future Directions | | | | 2018 | | Scopus | title/abstract | chapter |
| 1811 | L., Palazzo | Overview of the Diagnosis, Evaluation, and Novel Treatment Strategies for Ophthalmic Emergencies in the Hospitalized Geriatric Patient | | | | 2018 | | Scopus | title/abstract | irrelevant |
| 1812 | H., Nalci B | Necrotic intraocular retinoblastoma associated with orbital cellulitis | | | | 2018 | | Scopus | title/abstract | irrelevant |
| 1813 | M.K., Srini | Management of Extraocular Infections | | | | 2017 | | Scopus | title/abstract | irrelevant |
| 1814 | S.A., LaPon | When an orbital infection isn’t infectious at all: A review of orbital inflammatory syndrome | | | | 2017 | | Scopus | title/abstract | irrelevant |
| 1815 | C., McDonal | Management of cervicofacial infections: a survey of current practice in maxillofacial units in the UK | | | | 2017 | | Scopus | fulltext | ineligible intervention |
| 1816 | B.A., Wineg | Imaging of Pediatric Paranasal Sinus and Orbital Infections | | | | 2017 | | Scopus | title/abstract | review |
| 1817 | A.A., Campb | Re: “Intravenous Steroids With Antibiotics on Admission for Children With Orbital Cellulitis” | | | | 2017 | | Scopus | title/abstract | letter to editor |
| 1818 | L., Chen, L | Reply re: “Intravenous Steroids With Antibiotics on Admission for Children With Orbital Cellulitis” | | | | 2017 | | Scopus | title/abstract | letter to editor |
| 1819 | L., Kersan, | Anaesthesia for maxillofacial surgery | | | | 2017 | | Scopus | title/abstract | irrelevant |
| 1820 | M., Rhatiga | Orbital abscess following posterior subtenon injection of triamcinolone acetonide | | | | 2017 | | Scopus | title/abstract | case report |
| 1821 | V., Sciarre | Management of orbital cellulitis and subperiosteal orbital abscess in pediatric patients: A ten-year review | | | | 2017 | | Scopus | fulltext | ineligible intervention |
| 1822 | S.M., Scofi | Metastatic Colon Cancer to the Sphenoid Wing and Ethmoid Sinus Mimicking Orbital Cellulitis: A Rare Presentation | | | | 2017 | | Scopus | title/abstract | irrelevant |
| 1823 | G.A., Duart | Case series: Two cases of eyeball tattoos with short-term complications | | | | 2017 | | Scopus | title/abstract | irrelevant |
| 1824 | N., Pushker | Intra-ocular medulloepithelioma as a masquerade for PHPV and Panophthalmitis: a Diagnostic Dilemma | | | | 2017 | | Scopus | title/abstract | irrelevant |
| 1825 | J., Kim, Jo | Intraocular foreign body entering the anterior chamber through the mouth: A case report | | | | 2017 | | Scopus | title/abstract | irrelevant |
| 1826 | F., Al.Mulh | Case report: Orbital cellulitis in old patient with history of ocular intervention | | | | 2017 | | Scopus | title/abstract | case report |
| 1827 | D., Kim, Do | Ascending Facial Necrotizing Fasciitis in a Patient Taking a Bisphosphonate | | | | 2017 | | Scopus | title/abstract | irrelevant |
| 1828 | J., Temnogo | The best of the best: a review of select oculoplastic case series published in 2015 | | | | 2017 | | Scopus | title/abstract | irrelevant |
| 1829 | R., Verma, | Orbital perivenous abscess complicating the diagnosis and management of orbital cellulitis | | | | 2017 | | Scopus | title/abstract | case report |
| 1830 | Y., Hsu, Yu | Orbital cellulitis presenting as giant cell arteritis: A case report | | | | 2017 | | Scopus | title/abstract | case report |
| 1831 | S., Aulakh, | Orbital cellulitis with endogenous panophthalmitis caused by methicillin-sensitive staphylococcus aureus in pregnancy | | | | 2017 | | Scopus | title/abstract | irrelevant |
| 1832 | B., Reiss, | Antibiotic prophylaxis in orbital fractures | | | | 2017 | | Scopus | title/abstract | irrelevant |
| 1833 | A.M., Kolom | Bilateral cavernous sinus thrombosis in a patient with tacrolimus-associated posttransplant thrombotic microangiopathy | | | | 2017 | | Scopus | title/abstract | irrelevant |
| 1834 | R., Raina, | Infection associated acute interstitial nephritis; a case report | | | | 2017 | | Scopus | title/abstract | irrelevant |
| 1835 | A.C., Weber | 2017 | Scopus | No | excluded | | title/abstract | |  |  |
| 1836 | M.A., Al Sh | Nasal type extranodal natural killer/T (NK/T) cell lymphoma presenting as periorbital cellulitis: A case report | | | | 2016 | | Scopus | title/abstract | irrelevant |
| 1837 | A.D., Havle | Olopatadine hydrochloride and fluticasone propionate in topical treatment of allergic rhinitis: A single blind randomised study | | | | 2016 | | Scopus | title/abstract | irrelevant |
| 1838 | S.S.M., Fun | Ophthalmomyiasis interna masquerading as orbital cellulitis | | | | 2016 | | Scopus | title/abstract | irrelevant |
| 1839 | A., Galindo | Recurrent orbital inflammation mimicking orbital cellulitis associated with orbitopalpebral venous lymphatic malformation | | | | 2016 | | Scopus | title/abstract | case report |
| 1840 | D., Yin, De | Analysis of acute sinusitis or nasal furuncle derived orbitalcellulitis in children: review of 18 cases | | | | 2016 | | Scopus | fulltext | unclear |
| 1841 | Y., Wan, Yu | Treatment of orbital complications following acute rhinosinusitis in children | | | | 2016 | | Scopus | fulltext | ineligible population |
| 1842 | M.S., Benni | Acute Rhinosinusitis: Prescription Patterns in a Real-World Setting | | | | 2016 | | Scopus | title/abstract | irrelevant |
| 1843 | M., Mikhail | Orbital cellulitis and multiple abscess formation after strabismus surgery | | | | 2016 | | Scopus | title/abstract | case report |
| 1844 | G.H., Moore | Orbital relapsing polychondritis: A unique presentation, complication, and treatment | | | | 2016 | | Scopus | title/abstract | case report |
| 1845 | E.T., Cunni | Drugs, Inflammation, and the Eye | | | | 2016 | | Scopus | title/abstract | irrelevant |
| 1846 | E., Papavas | Ipilimumab-induced Ocular and Orbital Inflammation - A Case Series and Review of the Literature | | | | 2016 | | Scopus | title/abstract | irrelevant |
| 1847 | D.R., Lefeb | A Case Series and Review of Bisphosphonate-associated Orbital Inflammation | | | | 2016 | | Scopus | title/abstract | irrelevant |
| 1848 | B.W., Davie | Concurrent Endophthalmitis and Orbital Cellulitis from Metastatic Klebsiella pneumonia Liver Abscess | | | | 2016 | | Scopus | title/abstract | irrelevant |
| 1849 | A.H., Laula | Intracranial abscesses over the last four decades; Changes in aetiology, diagnostics, treatment and outcome | | | | 2016 | | Scopus | title/abstract | irrelevant |
| 1850 | J., Danan, | Periorbital necrotizing fasciitis following dexamethasone intravitreal implant injection | | | | 2016 | | Scopus | title/abstract | case report |
| 1851 | N., Alsaif, | External Ophthalmomyiasis Due to Dermatobia hominis Masquerading As Orbital Cellulitis | | | | 2016 | | Scopus | title/abstract | irrelevant |
| 1852 | A., Pirbhai | Bisphosphonate-Induced Orbital Inflammation: A Case Series and Review | | | | 2015 | | Scopus | title/abstract | irrelevant |
| 1853 | A.K., Joshi | A rare case of idiopathic orbital inflammatory syndrome | | | | 2015 | | Scopus | title/abstract | case report |
| 1854 | A.C., Fang, | Pediatric acute bacterial sinusitis diagnostic and treatment dilemmas | | | | 2015 | | Scopus | title/abstract | irrelevant |
| 1855 | R., Kahloun | Orbital infections: Review of 28 cases; Les infections orbitaires: A propos de 28 cas | | | | 2015 | | Scopus | title/abstract | unclear |
| 1856 | C.A., Press | Cavernous Sinus Thrombosis in Children: Imaging Characteristics and Clinical Outcomes | | | | 2015 | | Scopus | title/abstract | irrelevant |
| 1857 | B.W., Davie | C-Reactive Protein As a Marker for Initiating Steroid Treatment in Children with Orbital Cellulitis | | | | 2015 | | Scopus | fulltext | included |
| 1858 | A., Sayani, | Clinical Recognition and Management of an Atypical Dacryoadenitis | | | | 2015 | | Scopus | title/abstract | case report |
| 1859 | N., Furuya, | Relapsing polychondritis with different types of ocular inflammations | | | | 2015 | | Scopus | title/abstract | irrelevant |
| 1860 | F., Pakdel, | Masquerading Orbital Abscess Following Attempted Hydrogel Scleral Buckle Removal: Diagnostic Value of Orbital Magnetic Resonance Spectroscopy | | | | 2015 | | Scopus | title/abstract | irrelevant |
| 1861 | B., Lee, Br | Intracranial Infectious Aneurysm in Orbital Cellulitis | | | | 2015 | | Scopus | title/abstract | irrelevant |
| 1862 | R.T., Carli | Differential diagnosis of the swollen red eyelid | | | | 2015 | | Scopus | title/abstract | irrelevant |
| 1863 | M.S., Atfeh | Orbital infections: Five-year case series, literature review and guideline development | | | | 2015 | | Scopus | title/abstract | irrelevant |
| 1864 | Q., Vu, Qui | A rare case of odontogenic parainfectious intraorbital optic neuritis with the absence of clinical manifestation of orbital cellulitis | | | | 2015 | | Scopus | title/abstract | case report |
| 1865 | B.P., Erick | Orbital cellulitis and subperiosteal abscess: A 5-year outcomes analysis | | | | 2015 | | Scopus | title/abstract | irrelevant |
| 1866 | R., Rasteni | Odontogenic maxillofacial infections: A ten-year retrospective analysis | | | | 2015 | | Scopus | title/abstract | irrelevant |
| 1867 | M.S., Harri | A swollen right eye in a child | | | | 2015 | | Scopus | title/abstract | case report |
| 1868 | P., Taravat | Neuro-Sweet disease causing orbital inflammation | | | | 2015 | | Scopus | title/abstract | case report |
| 1869 | H., Yun, Hy | An unusual cause of acute maxillary sinusitis in a 9-year-old child: Odontogenic origin of infected dentigerous cyst with supernumerary teeth | | | | 2015 | | Scopus | title/abstract | case report |
| 1870 | E., Dhrami- | Bilateral orbital abscesses after strabismus surgery | | | | 2015 | | Scopus | title/abstract | case report |
| 1871 | S.P., Kurup | Characterization of dacryops infections | | | | 2015 | | Scopus | title/abstract | irrelevant |
| 1872 | K., Doumbia | Cervico-facial cellulitis during pregnancy: about a series of 10 cases in Mali; Cellulite cervico-faciale au cours de la grossesse. À propos d’une série de 10 cas au Mali | | | | 2014 | | Scopus | title/abstract | irrelevant |
| 1873 | H., Chahed, | Management of ocular and orbital complications in acute sinusitis; Prise en charge des complications oculo-orbitaires des sinusites aiguës | | | | 2014 | | Scopus | title/abstract | irrelevant |
| 1874 | M.J., Wan, | Complications of strabismus surgery: Incidence and risk factors | | | | 2014 | | Scopus | title/abstract | irrelevant |
| 1875 | J.A.D.J., B | Carotid-cavernous fistula; Fístula carótido-cavernosa | | | | 2014 | | Scopus | title/abstract | irrelevant |
| 1876 | A.G., Angel | In vein: Blinding bilateral ophthalmic thrombosis | | | | 2014 | | Scopus | title/abstract | irrelevant |
| 1877 | W.S., Lim, | Isolated extraocular muscle abscess presenting 40 years after squint surgery | | | | 2014 | | Scopus | title/abstract | case report |
| 1878 | S., Park, S | Orbital inflammation secondary to a delayed hypersensitivity reaction to sub-Tenon's hyaluronidase | | | | 2014 | | Scopus | title/abstract | case report |
| 1879 | E.L.R., Sou | Quantification of endotoxins in infected root canals and acute apical abscess exudates: Monitoring the effectiveness of root canal procedures in the reduction of endotoxins | | | | 2014 | | Scopus | title/abstract | irrelevant |
| 1880 | L., Arrico, | Acute monolateral proptosis and orbital myositis in a patient with discoid lupus erythematosus: A case report | | | | 2014 | | Scopus | title/abstract | case report |
| 1881 | R.I., Ayabe | Adalimumab as steroid-sparing treatment of inflammatory-stage thyroid eye disease | | | | 2014 | | Scopus | title/abstract | irrelevant |
| 1882 | T., Marchin | An aggressive primary orbital natural killer/T-cell lymphoma case: Poor response to chemotherapy | | | | 2014 | | Scopus | title/abstract | irrelevant |
| 1883 | O.R., Sadeq | The effect of ibuprofen on hepatic glutamic pyruvic transaminase (SGPT), glutamic oxaloacetic transaminase (SGOT) and Alkaline phosphatase (ALP) in dental patients | | | | 2014 | | Scopus | title/abstract | irrelevant |
| 1884 | G.V., Sowmy | Facial pain followed by unilateral facial nerve palsy: A case report with literature review | | | | 2014 | | Scopus | title/abstract | case report |
| 1885 | A., Kurashi | A case of IgG4-related disease of the orbital foramen and paranasal sinuses | | | | 2014 | | Scopus | title/abstract | case report |
| 1886 | A.F., DeAng | Review article: Maxillofacial emergencies: Oral pain and odontogenic infections | | | | 2014 | | Scopus | title/abstract | review |
| 1887 | A.G., Nair, | Intraocular malignant melanoma of the choroid presenting as orbital cellulitis | | | | 2014 | | Scopus | title/abstract | irrelevant |
| 1888 | A., Jose, A | Odontogenic infection and pachymeningitis of the cavernous sinus | | | | 2014 | | Scopus | title/abstract | short communication |
| 1889 | J.A., Huntb | Salmon patch conjunctiva associated with odontogenic infection | | | | 2014 | | Scopus | title/abstract | case report |
| 1890 | S., Strul, | Orbital cellulitis and intraconal abscess formation after strabismus surgery in an adult patient | | | | 2014 | | Scopus | title/abstract | case report |
| 1891 | N., Sahoo, | Necrotizing fasciitis of the cervico-facial region due to odontogenic infection | | | | 2014 | | Scopus | title/abstract | case report |
| 1892 | R., Wolf, R | Periorbital (eyelid) dermatides | | | | 2014 | | Scopus | title/abstract | irrelevant |
| 1893 | N., Kikuchi | Dental infection as a triggering factor in palmoplantar pustulosis | | | | 2013 | | Scopus | title/abstract | irrelevant |
| 1894 | E., Thomas, | Wells syndrome in a diabetic patient | | | | 2013 | | Scopus | title/abstract | irrelevant |
| 1895 | A.M., Fay, | Prophylactic postoperative antibiotics for enucleation and evisceration | | | | 2013 | | Scopus | title/abstract | irrelevant |
| 1896 | E., Muzzi, | Bilateral orbital preseptal cellulitis after combined adenotonsillectomy and strabismus surgery-Case report and pathogenetic hypothesis | | | | 2013 | | Scopus | title/abstract | irrelevant |
| 1897 | J.A., Walin | Retinoblastoma presenting with orbital cellulitis | | | | 2013 | | Scopus | title/abstract | irrelevant |
| 1898 | M., Lecoufl | Orbital myositis associated with ipilimumab; Myosite orbitaire associée à un traitement par ipilimumab | | | | 2013 | | Scopus | title/abstract | irrelevant |
| 1899 | F.L., Shahi | Cogan's syndrome associated with orbital inflammation | | | | 2013 | | Scopus | title/abstract | case report |
| 1900 | S., Kim, Su | Orbital inflammation developing from epidemic keratoconjunctivitis in an adult | | | | 2013 | | Scopus | title/abstract | case report |
| 1901 | S., Kamal, | Cysticercosis causing orbital cellulitis and panophthalmitis in dengue fever | | | | 2013 | | Scopus | title/abstract | case report |
| 1902 | K., Lee, Ky | Blindness resulting from orbital cellulitis following rhinoplasty | | | | 2013 | | Scopus | title/abstract | irrelevant |
| 1903 | Y.C., Chin, | Postoperative orbital swelling - Causes, diagnosis and management | | | | 2013 | | Scopus | title/abstract | irrelevant |
| 1904 | C., Park, C | A case of odontogenic orbital cellulitis causing blindness by severe tension orbit | | | | 2013 | | Scopus | title/abstract | case report |
| 1905 | Z., Bednaří | Orbital cellulitis in childhood; Orbitocelulitida v dětském věku | | | | 2013 | | Scopus | title/abstract | irrelevant |
| 1906 | T., Yamamot | Pustulotic arthro-osteitis associated with palmoplantar pustulosis | | | | 2013 | | Scopus | title/abstract | irrelevant |
| 1907 | M., Dey, Mo | Superior ophthalmic vein thrombosis as an initial manifestation of antiphospholipid syndrome. | | | | 2013 | | Scopus | title/abstract | irrelevant |
| 1908 | R.R., Pine, | CD56 negative extranodal NK/T-cell lymphoma of the orbit mimicking orbital cellulitis. | | | | 2013 | | Scopus | title/abstract | irrelevant |
| 1909 | N., Pushker | Role of oral corticosteroids in orbital cellulitis | | | | 2013 | | Scopus | duplicate | duplicate |
| 1910 | B.V., Chawl | MRI in retinoblastoma with orbital cellulitis | | | | 2013 | | Scopus | duplicate | duplicate |
| 1911 | M.P., Rabin | Diesel fuel injury to the orbit | | | | 2013 | | Scopus | title/abstract | irrelevant |
| 1912 | L., Gilardi | Odontogenic infections in the Internal Medicine Service | | | | 2012 | | Scopus | title/abstract | letter to editor |
| 1913 | A., Waris, | A rare case of Intraorbital abscess engulfing the optic nerve in an anemic child | | | | 2012 | | Scopus | title/abstract | irrelevant |
| 1914 | O., Abdoura | Bilateral intraorbital abscesses with intracranial complications in a young Cameroonian girl: A case report | | | | 2012 | | Scopus | title/abstract | case report |
| 1915 | P.J., Ryan, | Severe dental infections in the emergency department | | | | 2012 | | Scopus | title/abstract | review |
| 1916 | S.E., Choon | Conidiobolomycosis in a young malaysian woman showing chronic localized fibrosing leukocytoclastic vasculitis: A case report and meta-analysis focusing on clinicopathologic and therapeutic correlations with outcome | | | | 2012 | | Scopus | title/abstract | irrelevant |
| 1917 | R., Shinder | Re: "Retinoblastoma associated orbital cellulitis" | | | | 2012 | | Scopus | title/abstract | irrelevant |
| 1918 | A., Takeuch | Bilateral orbital inflammation following intravesical bacille Calmette-Guérin immunotherapy for bladder cancer | | | | 2012 | | Scopus | title/abstract | irrelevant |
| 1919 | B., Eshragh | A case report of orbital pseudotumor with presentation like orbital cellulitis | | | | 2012 | | Scopus | title/abstract | case report |
| 1920 | H., Moss, H | An unusual response of dental sepsis to antibiotics: Parallels with the Jarisch-Herxheimer reaction | | | | 2012 | | Scopus | title/abstract | irrelevant |
| 1921 | C., Ramu, C | Indications of antibiotic prophylaxis in dental practice-Review | | | | 2012 | | Scopus | title/abstract | irrelevant |
| 1922 | M., Kosaka, | Cutaneous small vessel vasculitis accompanied by pustulosis palmaris et plantaris | | | | 2012 | | Scopus | title/abstract | irrelevant |
| 1923 | M., Hiremat | Heparin in the long-term management of ligneous conjunctivitis: A case report and review of literature | | | | 2011 | | Scopus | title/abstract | irrelevant |
| 1924 | R., Sánchez | Severe odontogenic infections: Epidemiological, microbiological and therapeutic factors | | | | 2011 | | Scopus | title/abstract | irrelevant |
| 1925 | D.T.G., Hob | Pregnancy complicated by recurrent brain abscess after extraction of an infected tooth | | | | 2011 | | Scopus | title/abstract | irrelevant |
| 1926 | J.C.P., Roo | Necrotizing group A streptococcal periorbital infection following adalimumab therapy for rheumatoid arthritis | | | | 2011 | | Scopus | title/abstract | irrelevant |
| 1927 | V., Thunstr | Primary eye care | | | | 2011 | | Scopus | title/abstract | irrelevant |
| 1928 | P.S., Canno | A multi-centre case series investigating the aetiology of hypertrophic pachymeningitis with orbital inflammation | | | | 2011 | | Scopus | title/abstract | irrelevant |
| 1929 | R., Battikh | Cerebral actinomycosis pseudotumor: A case report; Actinomycose cérébrale d'aspect pseudotumoral : à propos d'un cas | | | | 2011 | | Scopus | title/abstract | irrelevant |
| 1930 | K., Igawa, | Possible association of Henoch-Schönlein purpura in adults with odontogenic focal infection | | | | 2011 | | Scopus | title/abstract | irrelevant |
| 1931 | D.E.C., Bar | An evidence based review of Periorbital Cellulitis | | | | 2011 | | Scopus | title/abstract | letter to editor |
| 1932 | V.L., Vaska | Community-associated methicillin-resistant staphylococcus aureus causing orbital cellulitis in Australian children | | | | 2011 | | Scopus | title/abstract | irrelevant |
| 1933 | J., Fozard, | Periorbital cellulits - A mistaken diagnosis! | | | | 2011 | | Scopus | title/abstract | irrelevant |
| 1934 | I.G., Georg | Intraocular safari: Ophthalmomyiasis interna | | | | 2011 | | Scopus | title/abstract | irrelevant |
| 1935 | S., Ortiz-P | Two cases of drug-induced orbital inflammatory disease | | | | 2011 | | Scopus | title/abstract | irrelevant |
| 1936 | S., Lee, Se | Management of preseptal and orbital cellulitis | | | | 2011 | | Scopus | title/abstract | review |
| 1937 | V.V., Jutha | Successful management of methicillin-resistant Staphylococcus aureus orbital cellulitis after blepharoplasty | | | | 2010 | | Scopus | title/abstract | irrelevant |
| 1938 | T.C., Zeril | Orbital pseudotumor after an upper respiratory infection: A comprehensive review | | | | 2010 | | Scopus | title/abstract | irrelevant |
| 1939 | A., Mallis, | Rhinocerebral mucormycosis: An update | | | | 2010 | | Scopus | title/abstract | irrelevant |
| 1940 | N., Masuda, | A case of posterior scleritis presenting various clinical findings | | | | 2010 | | Scopus | title/abstract | irrelevant |
| 1941 | M., Rossite | Posterior scleritis mimicking orbital cellulitis | | | | 2010 | | Scopus | title/abstract | irrelevant |
| 1942 | B.I., Barah | Forget me not | | | | 2010 | | Scopus | title/abstract | irrelevant |
| 1943 | J.N., Mbeke | Bisphosphonate-induced orbital inflammation | | | | 2010 | | Scopus | title/abstract | irrelevant |
| 1944 | J.C., Hwang | Acute orbitocranial inflammation following radioimmunotherapy for non-hodgkin lymphoma | | | | 2010 | | Scopus | title/abstract | irrelevant |
| 1945 | D., Kim, Do | Idiopathic orbital myositis mimicking orbital cellulitis | | | | 2010 | | Scopus | duplicate | duplicate |
| 1946 | E.E., Adder | Zygomycosis originating from an odontogenic infection in a pediatric oncology patient | | | | 2010 | | Scopus | title/abstract | case report |
| 1947 | A., Akhadda | Orbital abscess associated with sinusitis from odontogenic origin | | | | 2010 | | Scopus | title/abstract | case report |
| 1948 | M.A., Al-Mu | Unilateral facial nerve paralysis following an infected lower third molar | | | | 2010 | | Scopus | title/abstract | irrelevant |
| 1949 | K.R., Paude | Prevalence of pharmacotherapy in the department of paediatric dentistry | | | | 2010 | | Scopus | title/abstract | irrelevant |
| 1950 | K., Kassiri | Parainfectious optic neuropathy in a young patient with group A Streptococcus pyogenes orbital cellulitis | | | | 2010 | | Scopus | title/abstract | irrelevant |
| 1951 | T.G., Prade | Diffuse bilateral orbital inflammation in churg- strauss syndrome | | | | 2010 | | Scopus | title/abstract | irrelevant |
| 1952 | C.J., Magri | Rhinocerebral mucormycosis complicated by diabetes insipidus: A case report and literature review | | | | 2010 | | Scopus | title/abstract | irrelevant |
| 1953 | N., Radhakr | Conidiobolomycosis in relapsed acute lymphoblastic leukemia | | | | 2009 | | Scopus | title/abstract | irrelevant |
| 1954 | Y., Park, Y | Acute severe periocular swelling caused by periapical abscess in a dog | | | | 2009 | | Scopus | title/abstract | irrelevant |
| 1955 | J.T.L., Bay | Ocular vaccinia with severe restriction of extraocular motility | | | | 2009 | | Scopus | title/abstract | irrelevant |
| 1956 | R.D., Mahes | Acute dacryocystitis causing orbital cellulitis and abscess | | | | 2009 | | Scopus | title/abstract | irrelevant |
| 1957 | N., Pushker | Orbital abscess with unusual features | | | | 2009 | | Scopus | title/abstract | irrelevant |
| 1958 | E., la Orde | Review on periorbital and orbital cellulitis. A fifteen year's experience; Revisión de celulitis periorbitaria y orbitaria. Experiencia de quince años | | | | 2009 | | Scopus | title/abstract | irrelevant |
| 1959 | K., Hokazon | Herpes zoster ophthalmicus and orbital apex syndrome: Case report and literature review; Síndrome do ápice orbitário causada por herpes zóster oftálmico: Relato de caso e revisão da literatura | | | | 2009 | | Scopus | title/abstract | irrelevant |
| 1960 | I.G., Brook | Microbiology and antimicrobial treatment of orbital and intracranial complications of sinusitis in children and their management | | | | 2009 | | Scopus | title/abstract | review |
| 1961 | H.W., Lin, | Ludwig's angina following frenuloplasty in an adolescent | | | | 2009 | | Scopus | title/abstract | case report |
| 1962 | R.M.S., Cos | Orbital myositis: Diagnosis and management | | | | 2009 | | Scopus | title/abstract | irrelevant |
| 1963 | T., Tanaka, | Dental infection associated with nummular eczema as an overlooked focal infection | | | | 2009 | | Scopus | title/abstract | irrelevant |
| 1964 | U., Sethura | The red eye: Evaluation and management | | | | 2009 | | Scopus | title/abstract | irrelevant |
| 1965 | R.C., Kemps | Langerhans cell histiocytosis mimicking preseptal cellulitis | | | | 2009 | | Scopus | title/abstract | irrelevant |
| 1966 | M., Masud-U | Bony complications of chronic sinusitis | | | | 2009 | | Scopus | title/abstract | irrelevant |
| 1967 | R., Balasub | Burkitt lymphoma of the oral cavity: an atypical presentation | | | | 2009 | | Scopus | title/abstract | irrelevant |
| 1968 | S.M., Golds | Community-acquired methicillinresistant staphylococcus aureus periorbital cellulitis: A problem here to stay | | | | 2009 | | Scopus | title/abstract | irrelevant |
| 1969 | M.P., Hatto | Exaggerated postsurgical inflammation in a patient with insufficiently treated addison disease | | | | 2009 | | Scopus | title/abstract | irrelevant |
| 1970 | T.A.A., Vyz | Rhino-cerebral zygomycosis resistant to antimycotic treatment: A case report | | | | 2009 | | Scopus | title/abstract | irrelevant |
| 1971 | N.T., Annan | Outpatient management of severe gonococcal ophthalmia without genital infection | | | | 2008 | | Scopus | title/abstract | irrelevant |
| 1972 | N.M., Koede | Postoperative povidone-iodine prophylaxis in strabismus surgery | | | | 2008 | | Scopus | title/abstract | irrelevant |
| 1973 | V.A., Epste | Invasive Fungal Sinusitis and Complications of Rhinosinusitis | | | | 2008 | | Scopus | title/abstract | irrelevant |
| 1974 | S., Colnagh | ICHD-II diagnostic criteria for Tolosa-Hunt syndrome in idiopathic inflammatory syndromes of the orbit and/or the cavernous sinus | | | | 2008 | | Scopus | title/abstract | irrelevant |
| 1975 | D.D., Shome | Community-acquired methicillin-resistant Staphylococcus aureus (CAMRSA) - A rare cause of fulminant orbital cellulitis | | | | 2008 | | Scopus | title/abstract | irrelevant |
| 1976 | B.T., Özkan | Paresthesia of the mental nerve stem from periapical infection of mandibular canine tooth: a case report | | | | 2008 | | Scopus | title/abstract | irrelevant |
| 1977 | I., Athanas | A case of herpes zoster ophthalmicus associated with multiple ocular and extraocular manifestations | | | | 2008 | | Scopus | title/abstract | irrelevant |
| 1978 | A., Papier, | Differential diagnosis of the swollen red eyelid | | | | 2007 | | Scopus | title/abstract | irrelevant |
| 1979 | A., Armesto | Orbital cellulitis after Faden operation on the medial rectus | | | | 2007 | | Scopus | title/abstract | irrelevant |
| 1980 | K.B., Mille | The use of biologics and other immunosuppressants in the treatment of common inflammatory diseases in neuro-ophthalmology | | | | 2007 | | Scopus | title/abstract | irrelevant |
| 1981 | H.V., Danes | Orbital inflammatory disease | | | | 2007 | | Scopus | title/abstract | review |
| 1982 | A.M.A., Qad | Sudden visual loss in a pregnant woman | | | | 2007 | | Scopus | title/abstract | irrelevant |
| 1983 | R.W., Evans | Eye pain without visual symptoms | | | | 2007 | | Scopus | title/abstract | irrelevant |
| 1984 | J.R., Bilyk | Periocular infection | | | | 2007 | | Scopus | title/abstract | irrelevant |
| 1985 | K., Yoshifu | A case of subperiosteal orbital abscess | | | | 2007 | | Scopus | title/abstract | case report |
| 1986 | S.R., Durki | Successful treatment of a large choroidal abscess in an immunocompetent child | | | | 2007 | | Scopus | title/abstract | irrelevant |
| 1987 | R.M., Vagef | Atypical presentations of pleomorphic adenoma of the lacrimal gland | | | | 2007 | | Scopus | title/abstract | irrelevant |
| 1988 | S., Jindal, | Crucial distinction [8] | | | | 2007 | | Scopus | title/abstract | irrelevant |
| 1989 | A., Al-Rika | A case of herpes zoster presenting as orbital cellulitis. | | | | 2007 | | Scopus | title/abstract | case report |
| 1990 | A., Acocell | Acute thyroiditis of odontogenic origin. | | | | 2007 | | Scopus | title/abstract | irrelevant |
| 1991 | M.K., Brijl | Epidural empyema secondary to orbital cellulitis: A case report | | | | 2007 | | Scopus | title/abstract | irrelevant |
| 1992 | G., Güven, | Rosai-Dorfman disease of the parotid and submandibular glands: Salivary gland scintigraphy and oral findings in two siblings | | | | 2007 | | Scopus | title/abstract | irrelevant |
| 1993 | L.P.K., Ang | Orbital cellulitis following intralesional corticosteroid injection for periocular capillary haemangioma [3] | | | | 2007 | | Scopus | title/abstract | correspondence |
| 1994 | P., Farhi, | Orbital cellulitis associated with combined retinal and choroidal detachments [14] | | | | 2007 | | Scopus | title/abstract | irrelevant |
| 1995 | A.T., Komne | Ocular manifestations of natural canine monocytic ehrlichiosis (Ehrlichia canis): A retrospective study of 90 cases | | | | 2007 | | Scopus | title/abstract | irrelevant |
| 1996 | I.A., Chaud | Orbital cellulitis following implantation of aqueous drainage devices | | | | 2007 | | Scopus | title/abstract | irrelevant |
| 1997 | A.R., Sande | Orbital cysticercosis: Diagnosis and treatment controversies | | | | 2007 | | Scopus | title/abstract | irrelevant |
| 1998 | H., Kishiha | Review of unilateral exophthalmos at Kitasato University Hospital | | | | 2006 | | Scopus | title/abstract | irrelevant |
| 1999 | P.H., Blomq | Methicillin-resistant Staphylococcus aureus infections of the eye and orbit (an American ophthalmological society thesis) | | | | 2006 | | Scopus | title/abstract | irrelevant |
| 2000 | S.Y.W., Lia | Orbital cellulitis as a postoperative complication of sub-Tenon anaesthesia in cataract surgery | | | | 2006 | | Scopus | title/abstract | irrelevant |
| 2001 | M.W.S., Ho, | Use of interventional radiology in the management of mediastinitis of odontogenic origin | | | | 2006 | | Scopus | title/abstract | irrelevant |
| 2002 | É., Hoyama, | Blinding orbital cellulitis: A complication of strabismus surgery | | | | 2006 | | Scopus | title/abstract | irrelevant |
| 2003 | F.R., Ghosh | Intraorbital heroin injection resulting in orbital cellulitis and superior ophthalmic vein thrombosis | | | | 2006 | | Scopus | title/abstract | irrelevant |
| 2004 | T., Kobayas | Orbital cellulites showing external opthalmoplegia and blepharoptosis | | | | 2006 | | Scopus | title/abstract | irrelevant |
| 2005 | R.J., Gaspa | Bedside ultrasound of the soft tissue of the face: A case of early Ludwig's angina | | | | 2006 | | Scopus | title/abstract | irrelevant |
| 2006 | A., Norris, | Infections and foreign bodies in the ear, nose and throat | | | | 2006 | | Scopus | title/abstract | irrelevant |
| 2007 | E.S., Chiu, | Successful management of orbital cellulitis and temporary visual loss after blepharoplasty | | | | 2006 | | Scopus | title/abstract | case report |
| 2008 | M.D., Ricci | Unusual Vascular Anatomy in Interrupted Aortic Arch With Ventricular Septal Defect | | | | 2006 | | Scopus | title/abstract | irrelevant |
| 2009 | S.F., Capal | Jaw osteonecrosis associated with use of bisphosphonates and chemotherapy: Paradoxical complication of treatment of bone lesions in multiple myeloma patients | | | | 2006 | | Scopus | title/abstract | irrelevant |
| 2010 | R., Vashish | Anesthesia in a child with fibrodysplasia ossificans progressiva | | | | 2006 | | Scopus | title/abstract | irrelevant |
| 2011 | H., Jyonouc | SAPHO osteomyelitis and sarcoid dermatitis in a patient with DiGeorge syndrome | | | | 2006 | | Scopus | title/abstract | irrelevant |
| 2012 | M.T., Yen, | Current techniques for the management of orbital cellulitis | | | | 2006 | | Scopus | title/abstract | irrelevant |
| 2013 | I., Obuchow | A new approach towards giant cell arteritis; Współczesne poglady na temat olbrzymiokomórkowego zapalenia tetnic | | | | 2006 | | Scopus | title/abstract | irrelevant |
| 2014 | F.O.J., Luk | Late leakage of filtering bleb in a patient with orbital pseudotumor | | | | 2006 | | Scopus | title/abstract | irrelevant |
| 2015 | J., Anderso | Orbital pseudotumour presenting as orbital cellulitis | | | | 2006 | | Scopus | title/abstract | irrelevant |
| 2016 | C.E., Kloek | Role of inflammation in orbital cellulitis | | | | 2006 | | Scopus | title/abstract | irrelevant |
| 2017 | C., Huang, | Maxillary sinusitis and periapical abscess following periodontal therapy: A case report using three-dimensional evaluation | | | | 2006 | | Scopus | title/abstract | case report |
| 2018 | C., Kuo, Ch | Late onset lamellar keratitis and epithelial ingrowth following orbital cellulitis | | | | 2006 | | Scopus | title/abstract | irrelevant |
| 2019 | H.I., Ursu, | Late onset graves thyrotoxicosis in a patient with 18 years history of euthyroid graves ophthalmopathy | | | | 2005 | | Scopus | title/abstract | irrelevant |
| 2020 | M., Palamar | Orbital cellulitis after strabismus surgery | | | | 2005 | | Scopus | title/abstract | irrelevant |
| 2021 | T., Rutar, | Bilateral blindness from orbital cellulitis caused by community-acquired methicillin-resistant Staphylococcus aureus | | | | 2005 | | Scopus | title/abstract | irrelevant |
| 2022 | N.J., Schmi | Superior ophthalmic vein thrombosis in a patient with dacryocystitis- induced orbital cellulitis | | | | 2005 | | Scopus | title/abstract | irrelevant |
| 2023 | M.T., Yen, | Effect of corticosteroids in the acute management of pediatric orbital cellulitis with subperiosteal abscess | | | | 2005 | | Scopus | duplicate | duplicate |
| 2024 | J.B., Holds | Commentary on effect of corticosteroids in the acute management of pediatric orbital cellulitis with subperiosteal abscess | | | | 2005 | | Scopus | title/abstract | irrelevant |
| 2025 | A., Salam, | Orbital cellulitis or lymphoma? A diagnostic challenge | | | | 2005 | | Scopus | title/abstract | irrelevant |
| 2026 | D., Kobayas | A case of subperiosteal orbital abscess due to acute sinusitis | | | | 2005 | | Scopus | title/abstract | case report |
| 2027 | G., Dorosze | Septic thrombosis of the cavernous sinus complicated by intracerebral hemorrhage; Septyczne zakrzepowe zapalenie zatoki jamistej powikłane krwotokiem śródmózgowym. | | | | 2005 | | Scopus | title/abstract | irrelevant |
| 2028 | A., Rubinst | Posterior scleritis mimicking orbital cellulitis [11] | | | | 2005 | | Scopus | title/abstract | irrelevant |
| 2029 | A.M., Wane, | Senegalese experience of orbital cellulitis; Une expérience sénégalaise des cellulites orbitaires | | | | 2005 | | Scopus | title/abstract | irrelevant |
| 2030 | G.T., Clark | Orofacial pain and sensory disorders in the elderly | | | | 2005 | | Scopus | title/abstract | irrelevant |
| 2031 | P.V., Carro | Endodontics: Part 3 - Treatment of endodontic emergencies | | | | 2004 | | Scopus | title/abstract | irrelevant |
| 2032 | B., Vaisman | Dental treatment for children with chronic idiopathic thrombocytopaenic purpura: A report of two cases | | | | 2004 | | Scopus | title/abstract | irrelevant |
| 2033 | Z.H., Baqai | How serious are oral infections? | | | | 2004 | | Scopus | title/abstract | case report |
| 2034 | M., Agarwal | Retinoblastoma presenting as orbital cellulitis: Report of four with a review of the literature | | | | 2004 | | Scopus | title/abstract | irrelevant |
| 2035 | K., Oshitar | Sweet syndrome presenting as orbital cellulitis | | | | 2004 | | Scopus | title/abstract | case report |
| 2036 | C.M., Kumar | Orbital swelling following peribulbar and sub-Tenon's anaesthesia | | | | 2004 | | Scopus | title/abstract | irrelevant |
| 2037 | H., Withero | Management of oral and maxillofacial infection | | | | 2004 | | Scopus | title/abstract | irrelevant |
| 2038 | L.J., MacCh | Orbital cellulitis, panophthalmitis, and ecthyma gangrenosum in an immunocompromised host with Pseudomonas septicemia | | | | 2004 | | Scopus | title/abstract | case report |
| 2039 | W., Tavee, | An Unusual Presentation of a Cutaneous Odontogenic Sinus [5] | | | | 2003 | | Scopus | title/abstract | case report |
| 2040 | T., Satoh, | Chronic Nodular Prurigo Associated with Nummular Eczema: Possible Involvement of Odontogenic Infection [2] | | | | 2003 | | Scopus | title/abstract | letter to editor |
| 2041 | R.E., Marx, | Pamidronate (Aredia) and zoledronate (Zometa) induced avascular necrosis of the jaws: A growing epidemic [1] | | | | 2003 | | Scopus | title/abstract | irrelevant |
| 2042 | R.J., Campb | The acute red eye in the elderly | | | | 2003 | | Scopus | title/abstract | irrelevant |
| 2043 | X.D., Marti | Ocular complications of the Fernand-Widal triad and its therapy | | | | 2003 | | Scopus | title/abstract | irrelevant |
| 2044 | S., Kastenb | Brain and Spinal Cord Abscess | | | | 2003 | | Scopus | title/abstract | irrelevant |
| 2045 | K.P., Cocke | Orbital inflammation | | | | 2003 | | Scopus | title/abstract | irrelevant |
| 2046 | A.C., Goldm | Complications of sphenoid sinusitis | | | | 2003 | | Scopus | title/abstract | irrelevant |
| 2047 | I., Ejedepa | Apparent orbital cellulitis with acidosis and hyperglycemia | | | | 2003 | | Scopus | title/abstract | irrelevant |
| 2048 | S., Jain, S | Acute adduction deficit in a 7-week-old infant | | | | 2002 | | Scopus | title/abstract | irrelevant |
| 2049 | D.A., Jacob | Diagnosis and management of orbital pseudotumor | | | | 2002 | | Scopus | title/abstract | irrelevant |
| 2050 | T., Satoh, | Chronic pigmented purpura associated with odontogenic infection | | | | 2002 | | Scopus | title/abstract | irrelevant |
| 2051 | A.H., Dahlm | Orbital cellulitis following sub-Tenon’s anaesthesia | | | | 2002 | | Scopus | title/abstract | irrelevant |
| 2052 | L.U., Senne | Deep neck infections: Prospective study of 57 patients | | | | 2002 | | Scopus | title/abstract | irrelevant |
| 2053 | T., Alaçam, | Interappointment emergencies in teeth with necrotic pulps | | | | 2002 | | Scopus | duplicate | duplicate |
| 2054 | S., Sarma, | Brain-stem abscess successfully treated by microsurgical drainage: A case report | | | | 2001 | | Scopus | title/abstract | irrelevant |
| 2055 | A., Neumann | Orbital myositis. A rare differential diagnosis of sinugenic orbital complications; Die okuläre myositis. Eine seltene differenzialdiagnose der sinugenen orbitalen komplikation | | | | 2001 | | Scopus | title/abstract | irrelevant |
| 2056 | P.L., Lip, | A postoperative complication far worse than endophthalmitis: The coexistence of orbital cellulitis [10] | | | | 2001 | | Scopus | title/abstract | letter to editor |
| 2057 | M.A., Corso | Are dental infections a cause of brain abscess? Case report and review of the literature | | | | 2001 | | Scopus | title/abstract | irrelevant |
| 2058 | K., Fujii, | A case of orbital cellulitis as an early manifestation of acute retinal necrosis | | | | 2001 | | Scopus | title/abstract | irrelevant |
| 2059 | B., Redmill | Orbital cellulitis following corneal gluing under sub-Tenon's local anaesthesia [10] | | | | 2001 | | Scopus | title/abstract | irrelevant |
| 2060 | K.W., Morga | Sweet’s syndrome in acute myelogenous leukemia presenting as periorbital cellulitis with an infiltrate of leukemic cells | | | | 2001 | | Scopus | title/abstract | irrelevant |
| 2061 | T., Murakaw | Paropsia in a patient with sinusitis | | | | 2001 | | Scopus | title/abstract | irrelevant |
| 2062 | Z.F., Polla | Results and complications in 66 cases using a silicone tendon expander on overacting superior obliques with A-pattern anisotropias | | | | 2000 | | Scopus | title/abstract | irrelevant |
| 2063 | E.R., Menke | The effectiveness of prophylactic etodolac on postendodontic pain | | | | 2000 | | Scopus | title/abstract | irrelevant |
| 2064 | M.T., Chow, | Relapsing polychondritis | | | | 2000 | | Scopus | title/abstract | irrelevant |
| 2065 | M., Saeed, | Surgical treatment of anophthalmic socket - An experience with 42 intra orbital implants | | | | 2000 | | Scopus | title/abstract | irrelevant |
| 2066 | T., Oki, Te | Unilateral upper eyelid swelling as an initial sign in polyarteritis nodosa | | | | 2000 | | Scopus | title/abstract | irrelevant |
| 2067 | V., Rusu, V | Recurrent unilateral inflammatory exophthalmos; Exoftalmie unilaterala inflamatorie recurenta. | | | | 1999 | | Scopus | title/abstract | irrelevant |
| 2068 | S., Li, Suh | Unilateral Blastomyces dermatitidis endophthalmitis and orbital cellulitis: A case report and literature review | | | | 1998 | | Scopus | title/abstract | irrelevant |
| 2069 | L.R.G., Fav | Acute apical periodontitis: Incidence of post-operative pain using two different root canal dressings | | | | 1998 | | Scopus | duplicate | duplicate |
| 2070 | S.V.C., de | Alveolar osteitis (dry socket) in a dog: A case report | | | | 1998 | | Scopus | title/abstract | irrelevant |
| 2071 | P.B., Mulla | Retinoblastoma associated orbital cellulitis | | | | 1998 | | Scopus | title/abstract | irrelevant |
| 2072 | Z.A., Karci | Orbital cellulitis with retinoblastoma: Review of 14 cases | | | | 1997 | | Scopus | title/abstract | irrelevant |
| 2073 | P.E., Sirba | A case of orbital pseudotumor masquerading as orbital cellulitis in a patient with proptosis and fever | | | | 1997 | | Scopus | title/abstract | irrelevant |
| 2074 | S.L., Duda, | Retinoblastoma associated orbital cellulitis | | | | 1997 | | Scopus | duplicate | duplicate |
| 2075 | G., Popiela | Bilateral isolated posterior scleritis; Obustronne, izolowane zapalenie tylnej cześci twardówki. | | | | 1997 | | Scopus | title/abstract | irrelevant |
| 2076 | K., Nomiyam | Massive corticosteroid was effective for orbital cellulitis in a child | | | | 1997 | | Scopus | title/abstract | case report |
| 2077 | Z.F., Polla | Acute rectus muscle palsy in children as a result of orbital myositis | | | | 1996 | | Scopus | duplicate | duplicate |
| 2078 | F.J., Weins | Common eye disorders: Six patients to treat, pitfalls to avoid | | | | 1996 | | Scopus | title/abstract | irrelevant |
| 2079 | L.D., Ormer | Absidial rhino-orbital mucormycosis complicating the management of ocular trauma | | | | 1995 | | Scopus | title/abstract | irrelevant |
| 2080 | J.C., Merle | Ondontogenic cervical necrotizing fasciitis; CELLULITIS CERVICO FACIALES ODONTOGENIQUES | | | | 1995 | | Scopus | title/abstract | irrelevant |
| 2081 | I., Gonzále | Update of the treatment of orbital cellulitis in children. Management of subperiosteal abscess; ACTUALIZACION DEL TRATAMIENTO DE LAS CELULITIS ORBITARIAS EN LOS NINOS. MANEJO DEL ABSCESO SUBPERIOSTICO | | | | 1995 | | Scopus | title/abstract | irrelevant |
| 2082 | A., Shayega | Streptococcal gangrene of the eyelids and orbit | | | | 1995 | | Scopus | title/abstract | irrelevant |
| 2083 | L.A.M., van | Orbital cellulitis due to sinusitis; ORBITALE CELLULITIS ALS GEVOLG VAN NEUSBIJHOLTEONTSTEKING | | | | 1995 | | Scopus | title/abstract | irrelevant |
| 2084 | J.J., Shell | Adverse reaction to ibuprofen overdose. | | | | 1994 | | Scopus | title/abstract | irrelevant |
| 2085 | G.O., Akped | Localized extracranial infections in children with acute bacterial meningitis | | | | 1994 | | Scopus | title/abstract | irrelevant |
| 2086 | J.K., Forts | Bilateral ethmoid sinusitis with unilateral proptosis as an initial manifestation of metastatic prostate carcinoma. | | | | 1994 | | Scopus | title/abstract | irrelevant |
| 2087 | D.S., Barde | Neutrophilic Eccrine Hidradenitis Simulating Orbital Cellulitis | | | | 1994 | | Scopus | title/abstract | irrelevant |
| 2088 | V.Y.M., Won | Medical management of orbital infection | | | | 1994 | | Scopus | title/abstract | irrelevant |
| 2089 | P.R., Pavan | Exogenous Endophthalmitis Initially Treated without Systemic Antibiotics | | | | 1994 | | Scopus | title/abstract | irrelevant |
| 2090 | S.G., Pauke | A Rewarding Pursuit of Certainty | | | | 1993 | | Scopus | title/abstract | irrelevant |
| 2091 | J.G., Marsh | Factors associated with endodontic posttreatment pain | | | | 1993 | | Scopus | duplicate | duplicate |
| 2092 | M.P., Jacob | Bipolaris-lnduced orbital cellulitis | | | | 1992 | | Scopus | title/abstract | editorial comment |
| 2093 | W.J., Richt | Medical and surgical management of sinusitis in adults | | | | 1991 | | Scopus | title/abstract | irrelevant |
| 2094 | J.A., Shiel | Retinoblastoma manifesting as orbital cellulitis | | | | 1991 | | Scopus | title/abstract | irrelevant |
| 2095 | T.W., McGui | Fungal Endophthalmitis: An Experimental Study with a Review of 17 Human Ocular Cases | | | | 1991 | | Scopus | title/abstract | irrelevant |
| 2096 | Ghazali Mat | Ludwig's angina - A case report | | | | 1989 | | Scopus | title/abstract | case report |
| 2097 | J.A., Mauri | Pseudotumor and lymphoid tumor: Distinct clinicopathologic entities | | | | 1989 | | Scopus | title/abstract | irrelevant |
| 2098 | A., Orten, | Orbital cellulitis | | | | 1989 | | Scopus | title/abstract | irrelevant |
| 2099 | K., Kotzama | Orbital cellulitis due to mucormycosis - A case report | | | | 1988 | | Scopus | title/abstract | case report |
| 2100 | R.B., Patch | Ophthalmic complications with disseminated intravascular coagulation | | | | 1988 | | Scopus | title/abstract | irrelevant |
| 2101 | M.J., DiNub | Septic Thrombosis of the Cavernous Sinuses | | | | 1988 | | Scopus | title/abstract | irrelevant |
| 2102 | M.D., Sande | Acute presentation of thyroid ophthalmology | | | | 1986 | | Scopus | title/abstract | irrelevant |
| 2103 | C.F., Bladi | Clinico-statistical study of an anti-inflammatory agent in dentistry; Contributo clinico-statistico su un antinfiammatorio in stomatologia. | | | | 1986 | | Scopus | title/abstract | irrelevant |
| 2104 | J.T., Harve | Sheep botfly: Ophthalmomyiasis externa | | | | 1986 | | Scopus | title/abstract | irrelevant |
| 2105 | H.M., Malik | Recurrent sectorial corneal endotheliitis associated with dental abscess | | | | 1985 | | Scopus | title/abstract | irrelevant |
| 2106 | J.W., Dunne | OPTIC NERVE INVOLVEMENT IN GRAVES‘ OPHTHALMOPATHY: A CASE REPORT AND REVIEW | | | | 1985 | | Scopus | title/abstract | irrelevant |
| 2107 | T.J.K., Leo | GRAVES' DISEASE PRESENTING WITH BILATERAL ACUTE PAINFUL PROPTOSIS, PTOSIS, OPHTHALMOPLEGIA, AND VISUAL LOSS | | | | 1984 | | Scopus | title/abstract | irrelevant |
| 2108 | C.L., Bulle | Ocular Complications of Wegener's Granulomatosis | | | | 1983 | | Scopus | title/abstract | irrelevant |
| 2109 | J.A., Reyna | Head and Neck Infection After Renal Transplantation | | | | 1982 | | Scopus | title/abstract | irrelevant |
| 2110 | T., Hosoda, | Clinical Experience with Ceftizoxime in the Pediatric Field | | | | 1982 | | Scopus | title/abstract | irrelevant |
| 2111 | T., Harada, | An ophthalmoscopic appearance in the course of orbital apex syndrome (Japanese) | | | | 1976 | | Scopus | title/abstract | irrelevant |
| 2112 | S., Neto Aa | 1975 | Scopus | No | excluded | | title/abstract | |  |  |
| 2113 | T.E., Eilde | Fatal postextraction cerebral mucormycosis in an unknown diabetic | | | | 1974 | | Scopus | title/abstract | irrelevant |
| 2114 | E., Sonnabe | Treatment of apical periodontitis in gangrenous teeth; Therapie der apikalen Parodontitis gangränöser Zähne | | | | 1974 | | Scopus | title/abstract | irrelevant |
| 2115 | A., Diamant | 1974 | Scopus | No | excluded | | title/abstract | |  |  |
| 2116 | R.J., Latro | Septic emboli and pulmonary abscess secondary to odontogenic infection | | | | 1973 | | Scopus | title/abstract | irrelevant |
| 2117 | B.S., Choha | Epidemic keratoconjunctivitis with corneal involvement (clinical observation of 375 cases) | | | | 1973 | | Scopus | title/abstract | irrelevant |
| 2118 | J., Yulis M | 1972 | Scopus | No | excluded | | title/abstract | |  |  |
| 2119 | D., Gustinc | 1970 | Scopus | No | excluded | | title/abstract | |  |  |
| 2120 | M.A., Jokin | Pulp capping with corticoid-chemotherapeutic plus calciumhydroxide. | | | | 1970 | | Scopus | title/abstract | irrelevant |
| 2121 | M.A., Jokin | Current approaches in therapy in pulpal and periapical diseases; Pulpan- ja juurenhoidon mykyisistä suuntaukista. | | | | 1969 | | Scopus | title/abstract | irrelevant |
| 2122 | A., Baratie | Present progress in biologic therapy of septic-degenerative conditions of the dental pulp and of periapical affections; Progrès actuels dans la thérapeutique biologique des états septico-dégénératifs de la pulpe dentaire et des affections périapicales. | | | | 1968 | | Scopus | title/abstract | irrelevant |
| 2123 | D., Schlege | Experimental studies of the effects of endocrine disorders on the dental and skeletal system in the rat; Experimentelle Untersuchungen über die Auswirkung endokriner Störungen auf das Zahn- und Skelettsystem bei Ratten. | | | | 1968 | | Scopus | title/abstract | irrelevant |
| 2124 | A., Estefan | 1968 | Scopus | No | excluded | | title/abstract | |  |  |
| 2125 | K., Konishi | Clinical results in the use of Macrobin in parodontology | | | | 1967 | | Scopus | title/abstract | irrelevant |
| 2126 | A., Rossi, | 1966 | Scopus | No | excluded | | title/abstract | |  |  |
| 2127 | M., Pohto, | Treatment of periapical osteitis with chemotherapy and corticoid preparations; Periapikaalisen ostiitin hoidosta kemoterapeutti-kortikoidivalmisteella. | | | | 1965 | | Scopus | title/abstract | irrelevant |
| 2128 | NCT01997658, | Preoperative Glucocorticoid Use in Major Hepatectomy | | | | 2013 | | CENTRAL | title/abstract | trial registry |
| 2129 | NCT05951504, | The Prospective Evaluation of Peri-Operative Glucocorticoid Use in the Management of Cervicofacial Infections of Odontogenic Origin | | | | 2023 | | CENTRAL | title/abstract | trial registry |
| 2130 | NCT01957631, | Comparing Steroid Injections and Platelet Rich Plasma Injections in the Treatment of Plantar Fasciitis | | | | 2013 | | CENTRAL | title/abstract | trial registry |
| 2131 | NCT01994759, | Optimal Treatment of Plantar Fasciitis: physical Training, Glucocorticoid Injections or a Combination Thereof | | | | 2013 | | CENTRAL | title/abstract | trial registry |
| 2132 | NCT05914350, | Comparison Between Ultrasound Guided Ozone, Platelet-Rich Plasma or Steroid Injection in the Treatment of Sacroiliitis; a Randomized Double Blinded Controlled Study | | | | 2023 | | CENTRAL | title/abstract | trial registry |
| 2133 | NCT02448316, | Plantar Fasciitis, Operation or Conservative Treatment | | | | 2015 | | CENTRAL | title/abstract | trial registry |
| 2134 | NCT04493463, | Effects of Methylprednisolone Plus Ropivacaine Infiltration Before Wound Closure on Laminoplasty or Laminectomy | | | | 2020 | | CENTRAL | title/abstract | trial registry |
| 2135 | NCT02334475, | Steroid Versus Platelet Rich Plasma Injection for Chronic Low Back Pain | | | | 2014 | | CENTRAL | title/abstract | trial registry |
| 2136 | Owlia, MB; Salimzadeh, A; Alishiri, G; Haghighi, A | Comparison of two doses of corticosteroid in epidural steroid injection for lumbar radicular pain | | | | 2007 | | CENTRAL | title/abstract | irrelevant |
| 2137 | Pushker, N; Tejwani, LK; Bajaj, MS; Khurana, S; Velpandian, T; Chandra, M | Role of oral corticosteroids in orbital cellulitis | | | | 2013 | | CENTRAL | fulltext | included |
| 2138 | Rastegar, S; Baradaran Mahdavi, S; Hoseinzadeh, B; Badiei, S | Comparison of dry needling and steroid injection in the treatment of plantar fasciitis: a single-blind randomized clinical trial | | | | 2018 | | CENTRAL | title/abstract | irrelevant |
| 2139 | NCT03938896, | PRP IN Planter Fascitis | | | | 2019 | | CENTRAL | title/abstract | trial registry |
| 2140 | Ahmed, GS; Shaikh, AH; Tofique, M | Local steroid injection for treatment of planter fasciitis. Comparison between methylprednisolone and dexamethasone | | | | 2013 | | CENTRAL | title/abstract | irrelevant |
| 2141 | Acosta-Olivo, C; Elizondo-Rodriguez, J; Lopez-Cavazos, R; Vilchez-Cavazos, F; Simental-Mendia, M; Mendoza-Lemus, O | Plantar Fasciitis-A Comparison of Treatment with Intralesional Steroids versus Platelet-Rich Plasma A Randomized, Blinded Study | | | | 2017 | | CENTRAL | title/abstract | irrelevant |
| 2142 | Canyilmaz, E; Canyilmaz, F; Aynaci, O; Colak, F; Serdar, L; Uslu, GH; Aynaci, O; Yoney, A | Prospective Randomized Comparison of the Effectiveness of Radiation Therapy and Local Steroid Injection for the Treatment of Plantar Fasciitis | | | | 2015 | | CENTRAL | title/abstract | irrelevant |
| 2143 | Randhawa, FA; Butt, NF; Talat, SO; Sabir, SH; Qamar, MA | Effectiveness of intralesional steroid injections with dilatation in corrosive oesophageal strictures - A Randomized Control Trial | | | | 2018 | | CENTRAL | title/abstract | irrelevant |
| 2144 | KoÃ§er, G; Yuce, E; Tuzuner Oncul, A; Dereci, O; Koskan, O | Effect of the route of administration of methylprednisolone on oedema and trismus in impacted lower third molar surgery | | | | 2014 | | CENTRAL | fulltext | ineligible population |
| 2145 | NCT05339542, | Platelet Rich Plasma in Plantar Fasciitis | | | | 2022 | | CENTRAL | title/abstract | trial registry |
| 2146 | CTRI/2018/02/011764, | comparison between 3 steroid injections for treating heel pain | | | | 2018 | | CENTRAL | title/abstract | trial registry |
| 2147 | Yu, C; Huang, C; Mao, H; Yu, J | Comparative study of glucocorticoids versus NSAIDS for treatment of partial splenic embolization syndrome | | | | 2013 | | CENTRAL | title/abstract | irrelevant |
| 2148 | Mulherin, D; Price, M | Efficacy of tibial nerve block, local steroid injection or both in the treatment of plantar heel pain syndrome | | | | 2009 | | CENTRAL | title/abstract | irrelevant |
| 2149 | NCT06788158, | Clinical Effects of Incobotulinum Toxin vs Corticosteroid in Plantar Fascitis | | | | 2025 | | CENTRAL | title/abstract | trial registry |
| 2150 | Mardani-Kivi, M; Karimi Mobarakeh, M; Hassanzadeh, Z; Mirbolook, A; Asadi, K; Ettehad, H; Hashemi-Motlagh, K; Saheb-Ekhtiari, K; Fallah-Alipour, K | Treatment Outcomes of Corticosteroid Injection and Extracorporeal Shock Wave Therapy as Two Primary Therapeutic Methods for Acute Plantar Fasciitis: a Prospective Randomized Clinical Trial | | | | 2015 | | CENTRAL | title/abstract | irrelevant |
| 2151 | Elizondo-Rodriguez, J; Araujo-Lopez, Y; Moreno-Gonzalez, JA; Cardenas-Estrada, E; Mendoza-Lemus, O; Acosta-Olivo, C | A comparison of botulinum toxin a and intralesional steroids for the treatment of plantar fasciitis: a randomized, double-blinded study | | | | 2013 | | CENTRAL | duplicate | duplicate |
| 2152 | Hanselman, AE; Tidwell, JE; Santrock, RD | Cryopreserved human amniotic membrane injection for plantar fasciitis: a randomized, controlled, double-blind pilot study | | | | 2015 | | CENTRAL | title/abstract | irrelevant |
| 2153 | NCT04709484, | Comparison of the Effectiveness of USG and Palpation Guidance Steroid Injection in Patients With Plantar Fasciitis | | | | 2021 | | CENTRAL | title/abstract | trial registry |
| 2154 | Ball, EM; McKeeman, HM; Patterson, C; Burns, J; Yau, WH; Moore, OA; Benson, C; Foo, J; Wright, GD; Taggart, AJ | Steroid injection for inferior heel pain: a randomised controlled trial | | | | 2013 | | CENTRAL | title/abstract | irrelevant |
| 2155 | Celik, D; KuÅŸ, G; SÄ±rma, SÃ– | Joint Mobilization and Stretching Exercise vs Steroid Injection in the Treatment of Plantar Fasciitis: a Randomized Controlled Study | | | | 2016 | | CENTRAL | title/abstract | irrelevant |
| 2156 |  | STEROID INJECTIONS VERSUS AUTOLOGOUS BLOOD INJECTIONS: TREATMENT ANALYSIS IN PLANTAR FASCIITIS PATIENTS: a RANDOMIZED CONTROLLED TRIAL | | | | 2022 | | CENTRAL | title/abstract | irrelevant |
| 2157 | NCT06919458, | Short Course Steroids in Alcohol Associated Hepatitis | | | | 2025 | | CENTRAL | title/abstract | trial registry |
| 2158 | NCT05647291, | Is ESWT Better in Plantar Fasciitis Treatment? | | | | 2022 | | CENTRAL | title/abstract | trial registry |
| 2159 | Bunkar, ML; | Add-on prednisolone in the management of cervical lymph node tuberculosis | | | | 2016 | | CENTRAL | title/abstract | irrelevant |
| 2160 | Jain, SK; Suprashant, | Comparison of Plantar Fasciitis Injected With Platelet-Rich Plasma vs Corticosteroids | | | | 2018 | | CENTRAL | title/abstract | irrelevant |
| 2161 | Sharma, D | Comparative study of treatment outcome of plantar fasciitis with local steroid injection by ultrasound versus palpation technique | | | | 2018 | | CENTRAL | title/abstract | irrelevant |
| 2162 | Geetha, T; Premalatha, K; Jeyakhar, J | The Efficacy of Botulinum Toxin-A versus Methyl Prednisolone Acetate Injection in Reducing Pain and Improving Functional Outcome in Plantar Fasciitis | | | | 2023 | | CENTRAL | title/abstract | irrelevant |
| 2163 | Zamani, B; | Comparing the effect of low-power laser therapy with methylprednisolone injection in unilateral plantar fasciitis | | | | 2014 | | CENTRAL | duplicate | duplicate |
| 2164 | NCT04985396, | Platelet Rich Plasma Injection Compared With Steroid Injection for the Treatment of Plantar Fasciitis | | | | 2021 | | CENTRAL | title/abstract | trial registry |
| 2165 | Shetty, VD; | A study to compare the efficacy of corticosteroid therapy with platelet-rich plasma therapy in recalcitrant plantar fasciitis: a preliminary report | | | | 2014 | | CENTRAL | title/abstract | irrelevant |
| 2166 | Mahindra, P; | Chronic Plantar Fasciitis: effect of Platelet-Rich Plasma, Corticosteroid, and Placebo | | | | 2016 | | CENTRAL | title/abstract | irrelevant |
| 2167 | Ozbek, C; Aygenc | Use of steroids in the treatment of peritonsillar abscess | | | | 2004 | | CENTRAL | title/abstract | irrelevant |
| 2168 | Duhaut, P; Berruyer, M; Pinede | Anticardiolipin antibodies and giant cell arteritis: a prospective, multicenter case-control study. Groupe de Recherche sur l'ArtÃ©rite Ã  Cellules GÃ©antes | | | | 1998 | | CENTRAL | title/abstract | irrelevant |
| 2169 | Johannsen, F; | Endoscopic fasciotomy for plantar fasciitis provides superior results when compared to a controlled non-operative treatment protocol: a randomized controlled trial | | | | 2020 | | CENTRAL | title/abstract | irrelevant |
| 2170 | Tabrizi, A; | The Effect of Corticosteroid Local Injection Versus Platelet-Rich Plasma for the Treatment of Plantar Fasciitis in Obese Patients: a Single-Blind, Randomized Clinical Trial | | | | 2020 | | CENTRAL | title/abstract | irrelevant |
| 2171 | Welberry Smith, | Alemtuzumab induction in renal transplantation permits safe steroid avoidance with tacrolimus monotherapy: a randomized controlled trial | | | | 2013 | | CENTRAL | title/abstract | irrelevant |
| 2172 | Deng, D; Zhang, P; Guo, Y; Lim, TO | A randomised double-blind, placebo-controlled trial of allogeneic umbilical cord-derived mesenchymal stem cell for lupus nephritis | | | | 2017 | | CENTRAL | title/abstract | irrelevant |
| 2173 | Eslamian, F; Shakouri, SK; Jahanjoo, F; Hajialiloo, M; Notghi, F | Extra Corporeal Shock Wave Therapy Versus Local Corticosteroid Injection in the Treatment of Chronic Plantar Fasciitis, a Single Blinded Randomized Clinical Trial | | | | 2016 | | CENTRAL | title/abstract | irrelevant |
| 2174 | Aslam, S; Kamal, A; | Role of steroids in reduction of morbidity following mandibular wisdom tooth surgery | | | | 2012 | | CENTRAL | fulltext | included |
| 2175 | Haws, BE; Khechen | Impact of local steroid application on dysphagia following an anterior cervical discectomy and fusion: results of a prospective, randomized single-blind trial | | | | 2018 | | CENTRAL | title/abstract | irrelevant |
| 2176 | Selimovic, E; | Prevention of trismus with different pharmacological therapies after surgical extraction of impacted mandibular third molar | | | | 2017 | | CENTRAL | fulltext | irrelevant |
| 2177 | Sheridan, L; | Plantar fasciopathy treated with dynamic splinting: a randomized controlled trial | | | | 2010 | | CENTRAL | title/abstract | irrelevant |
| 2178 | Jain, K; Murphy, PN; Clough, TM | Platelet rich plasma versus corticosteroid injection for plantar fasciitis: a comparative study | | | | 2015 | | CENTRAL | title/abstract | irrelevant |
| 2179 | NCT01297686, | Exercise Versus Corticosteroid Randomized Clinical Trial for Plantar Fasciitis | | | | 2011 | | CENTRAL | title/abstract | trial registry |
| 2180 | NCT02600286, | Ulipristal Acetate In Disease Charcot-Marie-Tooth Type of 1A | | | | 2015 | | CENTRAL | title/abstract | trial registry |
| 2181 | AlcÃ¢ntara, CE | Pre-emptive effect of dexamethasone and methylprednisolone on pain, swelling, and trismus after third molar surgery: a split-mouth randomized triple-blind clinical trial | | | | 2014 | | CENTRAL | fulltext | ineligible population |
| 2182 | Babaei-Ghazani, A; Alyan, S | Ultrasound Guided Ozone vs. Steroid Injection for Plantar Fasciitis | | | | 2018 | | CENTRAL | title/abstract | irrelevant |
| 2183 | Dev, K; Meena, | A Randomized Control Study of Comparison of Standard Care versusUltrasonography Guided Single Dose of MethylprednisoloneAcetate Injection for Planar Fasciopathy | | | | 2022 | | CENTRAL | title/abstract | irrelevant |
| 2184 | KarakiliÃ§, GD; Aras, M; BÃ¼yÃ¼k, F; Bakirci, ES | PROLOTHERAPY VERSUS PHONOPHORESIS AND CORTICOSTEROID INJECTIONS FOR THE TREATMENT OF PLANTAR FASCIITIS: a RANDOMÄ°ZED, DOUBLE-BLIND CLINICAL TRIAL | | | | 2023 | | CENTRAL | title/abstract | irrelevant |
| 2185 | Jimenez-Perez, AE; Gonzalez-Arabio, D; Diaz, AS; Maderuelo, JA; Ramos-Pascua, LR | Clinical and imaging effects of corticosteroids and platelet-rich plasma for the treatment of chronic plantar fasciitis: a comparative non randomized prospective study | | | | 2019 | | CENTRAL | duplicate | duplicate |
| 2186 | NCT00220675, | Erythropoietin Spinal Cord Compression Randomized Trial | | | | 2005 | | CENTRAL | title/abstract | trial registry |
| 2187 | NCT03904966, | The Effect of Kinesio-tape and Shock Wave Therapy on Plantar Fasciitis | | | | 2019 | | CENTRAL | title/abstract | trial registry |
| 2188 | Babaei-Ghazani, A; Karimi, N; Forogh, B; Madani, SP; Ebadi, S; Fadavi, HR; Sobhani-Eraghi, A; Emami Razavi, SZ; Raeissadat, SA; Eftekharsadat, B | Comparison of Ultrasound-Guided Local Ozone (O2-O3) Injection vs Corticosteroid Injection in the Treatment of Chronic Plantar Fasciitis: a Randomized Clinical Trial | | | | 2019 | | CENTRAL | title/abstract | irrelevant |
| 2189 | NCT01835743, | Study of Low Level Laser Therapy to Treat Chronic Heel Pain Arising From Plantar Fasciitis | | | | 2013 | | CENTRAL | title/abstract | trial registry |
| 2190 | NCT03231150, | Plantar Fasciitis Randomized Clinical Control Trial | | | | 2017 | | CENTRAL | title/abstract | trial registry |
| 2191 | NCT04461197, | Efficacy of the Treatment of Plantar Orthoses With Extracorporeal Shock Wave Therapy in Plantar Fasciitis | | | | 2020 | | CENTRAL | title/abstract | trial registry |
| 2192 | NCT05984121, | Comparison of the Effectiveness of Local Ozone Injection and Dextrose Prolotherapy Injection in Chronic Plantar Fasciitis | | | | 2023 | | CENTRAL | title/abstract | trial registry |
| 2193 | NCT00189592, | Plantar Fasciosis Treatment Using Coblation | | | | 2005 | | CENTRAL | title/abstract | trial registry |
| 2194 | Rauf, MA | The benefits of steroids therapy in surgical extraction of Mandibular third molar | | | | 2015 | | CENTRAL | fulltext | ineligible population |
| 2195 | NCT06310122, | Effect of Extracorporeal Shockwave Therapy on Gait Parameters in Patients With Planter Fascitis | | | | 2024 | | CENTRAL | title/abstract | trial registry |
| 2196 | NCT05475899, | Comparison of Instrument Assisted Soft Tissue Mobilization and Kinesiology Taping on Plantar Fasciitis | | | | 2022 | | CENTRAL | title/abstract | trial registry |
| 2197 | NCT04993105, | Effects of Graston Assisted Soft Tissue Mobilization in Patients With Chronic Plantar Fasciitis | | | | 2021 | | CENTRAL | title/abstract | trial registry |
| 2198 | NCT06400433, | Comparing Efficacies of Median Nerve Hydrodissection With Dexamethasone and Dextrose in Carpal Tunnel Syndrome | | | | 2024 | | CENTRAL | title/abstract | trial registry |
| 2199 | NCT01882894, | Efficacy of a Custom Temporary Foot Orthosis for Plantar Fasciitis Treatment | | | | 2013 | | CENTRAL | title/abstract | trial registry |
| 2200 | NCT02196155, | Botulinum Toxin A Versus Steroids for the Treatment of Chronic Plantar Fasciitis | | | | 2014 | | CENTRAL | title/abstract | trial registry |
| 2201 | NCT02982226, | ReNuâ„¢ vs. Corticosteroids for the Treatment of Plantar Fasciitis | | | | 2016 | | CENTRAL | title/abstract | trial registry |
| 2202 | NCT04185259, | Acupuncture vs Sham Acupuncture or Waitlist Control for Patients With Chronic Planter Fasciitis | | | | 2019 | | CENTRAL | title/abstract | trial registry |
| 2203 | NCT01127672, | Treatment of Plantar Fasciitis With Platelet Rich Plasma | | | | 2010 | | CENTRAL | title/abstract | trial registry |
| 2204 | NCT00758641, | Platelet Rich Plasma to Treat Plantar Fasciitis | | | | 2008 | | CENTRAL | title/abstract | trial registry |
| 2205 | NCT06671223, | Effectiveness of Infiltrations in the Treatment of Plantar Fasciopathy | | | | 2024 | | CENTRAL | title/abstract | trial registry |
| 2206 | NCT04029389, | Ultrasound-Guided Tibial Nerve Block vs. Local Corticosteroid Injection in Recalcitrant Plantar Fasciitis Treatment | | | | 2019 | | CENTRAL | title/abstract | trial registry |
| 2207 | NCT04967703, | Physiotherapy Protocols in Treating Plantar Fasciitis | | | | 2021 | | CENTRAL | title/abstract | trial registry |
| 2208 | NCT04323319, | Comparions the Effect of Different Treatment Modalities on Chronic Plantar Fasiitis | | | | 2019 | | CENTRAL | title/abstract | trial registry |
| 2209 | NCT06284993, | Acupuncture of Different Treatment Frequency in Chronic Plantar Fasciitis | | | | 2024 | | CENTRAL | title/abstract | trial registry |
| 2210 | NCT06917937, | Is Exercise Program Added to Corticosteroid Injection Effective on Pain and Performance in Plantar Fasciitis? | | | | 2025 | | CENTRAL | title/abstract | trial registry |
| 2211 | NCT06737445, | Corticosteroid Injection, Extracorporeal Shock Wave Therapy, and Radiofrequency Ablation for Chronic Plantar Fasciitis | | | | 2024 | | CENTRAL | title/abstract | trial registry |
| 2212 | IRCT2017082029132N4, | Treatment of plantar fasciitis | | | | 2017 | | CENTRAL | title/abstract | trial registry |
| 2213 | Sharma, G; Gupta, A; Choudhary, AS | Role of corticosteroid (Methyl Prednisolone Injection) Versus Platelet Rich Plasma (PRP) in Treatment of Plantar Fasciitis | | | | 2024 | | CENTRAL | title/abstract | irrelevant |
| 2214 | Jiwon, Lee; Jin-Wha, Chung | A Prospective Study Comparing Steroid Injection and Needle Fenestration for the Treatment of Chronic Plantar Fasciitis | | | | 2021 | | CENTRAL | title/abstract | irrelevant |
| 2215 | Bharati, J; Gupta, | Comparison of two steroid regimens in induction therapy of proliferative lupus nephritis: a randomised controlled trial | | | | 2018 | | CENTRAL | title/abstract | irrelevant |
| 2216 | NCT04014244, | Dextrose, Corticosteroids and Surgical Release in Carpal Tunnel Syndrome | | | | 2019 | | CENTRAL | title/abstract | trial registry |
| 2217 | NCT04949373, | High Intensity Laser Therapy in Carpal Tunnel Syndrome | | | | 2021 | | CENTRAL | title/abstract | trial registry |
| 2218 | NCT04917406, | Effect of Iontophoresis vs. Ultrasound in Plantar Fasciitis". Plantar Fasciitis" | | | | 2021 | | CENTRAL | title/abstract | trial registry |
| 2219 | Crawford, F; Atkins, D; Young, P; Edwards, J | Steroid injection for heel pain: evidence of short-term effectiveness. A randomized controlled trial | | | | 1999 | | CENTRAL | title/abstract | irrelevant |
| 2220 | Chien-Min, Chen; | Effectiveness of Device-Assisted Ultrasound-Guided Steroid Injection for Treating Plantar Fasciitis | | | | 2013 | | CENTRAL | title/abstract | irrelevant |
| 2221 | Liaqat, Umar | Plantar fasciitis: intralesional steroid injections versus intralesional autologous blood injections | | | | 2018 | | CENTRAL | title/abstract | irrelevant |
| 2222 | CTRI/2020/08/027298, | Comparison of Results of Platelet Rich Plasma (PRP) Versus Steroid injection for heel pain | | | | 2020 | | CENTRAL | title/abstract | trial registry |
| 2223 | Ball, EMA | Steroid injection in Plantar Fasciitis: a placebo-controlled trial | | | | 2012 | | CENTRAL | title/abstract | irrelevant |
| 2224 | NCT06240507, | Posterior Tibial Nerve PRF vs Intralesional RFT for Painful Calcaneal Spur and Plantar Fasciitis | | | | 2024 | | CENTRAL | title/abstract | trial registry |
| 2225 | NCT03040557, | Flexible Footwear and Insole in Heel Pain | | | | 2016 | | CENTRAL | title/abstract | trial registry |
| 2226 | Tsai, WC; Hsu | Plantar fasciitis treated with local steroid injection: comparison between sonographic and palpation guidance | | | | 2006 | | CENTRAL | title/abstract | irrelevant |
| 2227 | Guner, S; Onder, H; Guner, SI; Ceylan, MF; GÃ¶kalp, MA; Keskin, S | Effectiveness of local tenoxicam versus corticosteroid injection for plantar fasciitis treatment | | | | 2013 | | CENTRAL | title/abstract | irrelevant |
| 2228 | CTRI/2024/01/061283, | A STUDY TO COMPARE THE EFFECT OF STEROID INJECTION WITH SHOCK WAVE THERAPY IN THE TREATMENT OF HEEL PAIN IN SMS MEDICAL COLLEGE, JAIPUR | | | | 2024 | | CENTRAL | title/abstract | trial registry |
| 2229 | NCT00893048, | The Use of Oral Steroids in the Treatment of Cellulitis | | | | 2009 | | CENTRAL | title/abstract | trial registry |
| 2230 | Monto, RR | Platelet-rich plasma efficacy versus corticosteroid injection treatment for chronic severe plantar fasciitis | | | | 2014 | | CENTRAL | title/abstract | irrelevant |
| 2231 | NCT05868577, | Infracalcaneal Peppering Injection Technique for Chronic Plantar Fasciitis | | | | 2023 | | CENTRAL | title/abstract | trial registry |
| 2232 | Roos, DE; Smith, JG | Randomized comparison radiation therapy and steroids for plantar fasciitis: in regard to canyilmaz ET al | | | | 2016 | | CENTRAL | title/abstract | irrelevant |
| 2233 | Amiot, A; Serrero, | One-year effectiveness and safety of vedolizumab therapy for inflammatory bowel disease: a prospective multicentre cohort study | | | | 2017 | | CENTRAL | title/abstract | irrelevant |
| 2234 | Demling, RH; DeSanti, L | Effect of the anabolic steroid oxandrolone on the rate of catabolism in acute necrotizing fasciitis | | | | 2003 | | CENTRAL | title/abstract | irrelevant |
| 2235 | Pedreira, AA; Wanderley, | Thermographic and clinical evaluation of 808-nm laser photobiomodulation effects after third molar extraction | | | | 2016 | | CENTRAL | title/abstract | irrelevant |
| 2236 | McMillan, AM; | Ultrasound guided corticosteroid injection for plantar fasciitis: randomised controlled trial | | | | 2012 | | CENTRAL | duplicate | duplicate |
| 2237 | Genovese, MC; | Peficitinib, a JAK Inhibitor, in Combination With Limited Conventional Synthetic Disease-Modifying Antirheumatic Drugs in the Treatment of Moderate-to-Severe Rheumatoid Arthritis | | | | 2017 | | CENTRAL | title/abstract | irrelevant |
| 2238 | IRCT201108157323N2, | Efficacy of dextrose 25% injection vs. steroid injection in the treatment of heel spur | | | | 2011 | | CENTRAL | title/abstract | trial registry |
| 2239 | Shuming, Li; Tong, Shen; Yongshan, Liang; Ying, Zhang; Bo, Bai | Miniscalpel-Needle versus Steroid Injection for Plantar Fasciitis: a Randomized Controlled Trial with a 12-Month Follow-Up | | | | 2014 | | CENTRAL | duplicate | duplicate |
| 2240 | Talimkhani, I; Jamalpour, MR; Babaei, H; Faradmal, J | Comparison of Intra-Socket Bupivacaine Administration Versus Oral Mefenamic Acid Capsule for Postoperative Pain Management Following Removal of Impacted Mandibular Third Molars | | | | 2019 | | CENTRAL | title/abstract | irrelevant |
| 2241 | Warraich, R; | Evaluation of postoperative discomfort following third molar surgery using submucosal dexamethasone - a randomized observer blind prospective study | | | | 2013 | | CENTRAL | fulltext | ineligible population |
| 2242 | Moshrif, A; Elwan, M | The Effect of Addition of Buffered Dextrose 5% Solution on Pain Occurring During Local Steroid Injection for Treatment of Plantar Fasciitis: a Randomized Controlled Trial | | | | 2019 | | CENTRAL | title/abstract | irrelevant |
| 2243 | CTRI/2021/04/032691, | Appropriate duration of steroids for brain worm infection | | | | 2021 | | CENTRAL | title/abstract | irrelevant |
| 2244 | Pappalardo, S; Puzzo, | The efficacy of four ways of administrating dexamethasone during surgical extraction of partially impacted lower third molars | | | | 2007 | | CENTRAL | fulltext | ineligible population |
| 2245 | Mubarak, HA; Al-Adily, SS | Randomized case-controlled clinical trial of the effect of preemptive etoricoxib, prednisolone and a control group on of postoperative sequelae after surgical removal of impacted mandibular third molars | | | | 2021 | | CENTRAL | fulltext | ineligible population |
| 2246 | NCT03005522, | The Effects of Dexamethasone on the Time to Pain Resolution in Dental Periapical Abscess | | | | 2016 | | CENTRAL | title/abstract | trial registry |
| 2247 | Sharma, R; Chaudhary, | Effect of platelet-rich plasma versus steroid injection in plantar fasciitis: a randomized clinical trial | | | | 2023 | | CENTRAL | title/abstract | irrelevant |
| 2248 | Koch, HJ; Szecsey, A | A randomized controlled trial of prednisone in Alzheimer's disease | | | | 2000 | | CENTRAL | title/abstract | irrelevant |
| 2249 | Chau, JK; Seikaly, HR | Corticosteroids in peritonsillar abscess treatment: a blinded placebo-controlled clinical trial | | | | 2014 | | CENTRAL | duplicate | duplicate |
| 2250 | ISRCTN57762240, | Ultrasound-guided versus palpation-guided steroid injection for plantar fascitis | | | | 2004 | | CENTRAL | title/abstract | trial registry |
| 2251 | Priyanga, R; Balamurugan, R; Rajan, PS | Comparison of dexamethasone administration through sublingual and intramuscular routes for evaluation of pain, swelling, and trismus after impacted mandibular third molar surgery-a prospective randomized controlled study | | | | 2022 | | CENTRAL | fulltext | ineligible population |
| 2252 | Ball, EMA; McKeeman, | Steroid injection for plantar fasciitisa placebo-controlled trial | | | | 2011 | | CENTRAL | title/abstract | irrelevant |
| 2253 | Jillani, SRUH; Mobushir, F | Comparison of intralesional steroid and extracorporal shockwave therapy for relief of pain in plantar fasciitis | | | | 2020 | | CENTRAL | title/abstract | irrelevant |
| 2254 | Srivastava, V; Vishwas, ; Rathi, R; Ln, M; Bl, K | PLANTAR FASCIITIS TREATMENT WITH PLATELET-RICH PLASMA INJECTION VERSUS STEROID INJECTION | | | | 2022 | | CENTRAL | title/abstract | irrelevant |
| 2255 | Spiera, RF; Mitnick, | A prospective, double-blind, randomized, placebo controlled trial of methotrexate in the treatment of giant cell arteritis (GCA) | | | | 2001 | | CENTRAL | title/abstract | irrelevant |
| 2256 | Bracco, P; Debernardi, C; | Efficacy of rofecoxib and nimesulide in controlling postextraction pain in oral surgery: a randomised comparative study | | | | 2004 | | CENTRAL | title/abstract | irrelevant |
| 2257 | Nozari, L; Klaudat, B; Longoni, C; Cavagni, J; Corsetti, A | Pre-operative use of injectable dexamethesone in impacted third molar surgery | | | | 2019 | | CENTRAL | fulltext | ineligible population |
| 2258 | Haq, A; Rehman, Iu; Ahmed, I; Ahmad, T | Comparison of autologous blood and steroid injection in patients with plantar fasciitis | | | | 2021 | | CENTRAL | title/abstract | irrelevant |
| 2259 | Black, AJ | 1996 | CENTRAL | No | excluded | | title/abstract | |  |  |
| 2260 | Yucel, I; Yazici, B; Degirmenci, E; Erdogmus, B; Dogan, S | Comparison of ultrasound-, palpation-, and scintigraphy-guided steroid injections in the treatment of plantar fasciitis | | | | 2009 | | CENTRAL | duplicate | duplicate |
| 2261 | Moshrif, A; Elwan, M; Daifallah, U | Deep friction massage versus local steroid injection for treatment of plantar fasciitis: a randomized controlled trial | | | | 2020 | | CENTRAL | title/abstract | irrelevant |
| 2262 | Genc, H; Saracoglu, M; Nacir, B; Erdem, HR; Kacar, M | Long-term ultrasonographic follow-up of plantar fasciitis patients treated with steroid injection | | | | 2005 | | CENTRAL | title/abstract | irrelevant |
| 2263 | Chen, CM; Chen, JS; Tsai, WC; Hsu, HC; Chen, KH; Lin, CH | Effectiveness of device-assisted ultrasound-guided steroid injection for treating plantar fasciitis | | | | 2013 | | CENTRAL | title/abstract | irrelevant |
| 2264 | Abdihakin, M; Wafula, K; Hasan, s; MacLeod, J | A randomised controlled trial of steroid injection in the management of plantar fasciitis | | | | 2012 | | CENTRAL | title/abstract | irrelevant |
| 2265 | PACTR201902720180705, | Effect of buffered dextrose on pain occurring during local steroid injection for plantar fasciitis | | | | 2019 | | CENTRAL | title/abstract | irrelevant |
| 2266 | Saber, N; Diab, H; Nassar, W; Razaak, HA | Ultrasound guided local steroid injection versus extracorporeal shockwave therapy in the treatment of plantar fasciitis | | | | 2012 | | CENTRAL | title/abstract | irrelevant |
| 2267 | ChiCTR1800014861, | Effect evaluation of musculoskeletal ultrasound interventional steroid injection combined with acupotomy therapy for plantar fasciitis | | | | 2018 | | CENTRAL | title/abstract | irrelevant |
| 2268 | CTRI/2025/05/087247, | Which Treatment Works Best for Chronic Heel Pain? Comparing PRP with Nerve Block, PRP Alone, and Steroid Injections | | | | 2025 | | CENTRAL | title/abstract | irrelevant |
| 2269 | Prakash, S; Kumar, M | Management of Chronic Plantar Fasciitis: a Comparative Study | | | | 2023 | | CENTRAL | title/abstract | irrelevant |
| 2270 | CTRI/2024/04/066454, | An interventional study to compare the effect of steroid injection versus dextrose prolotherapy in management of medial heel pain | | | | 2024 | | CENTRAL | title/abstract | irrelevant |
| 2271 | Elizondo-Rodriguez, J; | A comparison of botulinum toxin a and intralesional steroids for the treatment of plantar fasciitis: a randomized, double-blinded study | | | | 2013 | | CENTRAL | title/abstract | irrelevant |
| 2272 | IRCT2014030616865N1, | A comparison of shock wave versus corticosteroid for pain reduction in plantar fasciitis: randomized clinical trial | | | | 2014 | | CENTRAL | title/abstract | irrelevant |
| 2273 | Mukherjee, M; Kamra | Comparative evaluation of PRP vs Corticosteroid in management of plantar fasciitis | | | | 2023 | | CENTRAL | title/abstract | irrelevant |
| 2274 | ACTRN12622000808741, | Efficacy and safety of corticosteroids for orbital cellulitis: a randomised controlled trial | | | | 2022 | | CENTRAL | title/abstract | trial registry |
| 2275 | Hafez, AE; Ismail, FM; | Musculoskeletal Ultrasound Changes in Chronic Plantar Fascia after Treatment with Platelet Rich Plasma Compared to Steroid | | | | 2023 | | CENTRAL | title/abstract | irrelevant |
| 2276 | Nabeel Nazar, M; Ramadorai, AK | Efficacy of post-operative oral tramadol and diclofenac sodium on pain relief following mandibular third molar surgery: a double blind, randomized controlled trial | | | | 2014 | | CENTRAL | title/abstract | irrelevant |
| 2277 | CTRI/2017/12/010975, | A comparison of shock wave treatment versus steroid injection in the treatment of heel pain | | | | 2017 | | CENTRAL | title/abstract | trial registry |
| 2278 | Mpofu, S; Mpofu, CM | Steroids, non-steroidal anti-inflammatory drugs, and sigmoid diverticular abscess perforation in rheumatic conditions | | | | 2004 | | CENTRAL | title/abstract | irrelevant |
| 2279 | Kalaci, A; Cakici, H; | Treatment of plantar fasciitis using four different local injection modalities: a randomized prospective clinical trial | | | | 2009 | | CENTRAL | title/abstract | irrelevant |
| 2280 | Ahadi, T; Nik, SS; Forogh, B; Madani, SP; Raissi, GR | Comparison of the Effect of Ultrasound-Guided Injection of Botulinum Toxin Type A and Corticosteroid in the Treatment of Chronic Plantar Fasciitis: a Randomized Controlled Trial | | | | 2022 | | CENTRAL | title/abstract | irrelevant |
| 2281 | Schreiber, S; | A randomized, placebo-controlled trial of certolizumab pegol (CDP870) for treatment of Crohn's disease | | | | 2005 | | CENTRAL | title/abstract | irrelevant |
| 2282 | Eswara Reddy, G; | A PROSPECTIVE STUDY BETWEEN LOCAL STEROID INJECTION AND PLATELET RICH PLASMA IN THE TREATMENT OF PLANTAR FASCIITIS | | | | 2024 | | CENTRAL | title/abstract | irrelevant |
| 2283 | Fleischmann, RM; Tesser, J; Schiff, MH; Schechtman, J; Burmester, GR; Bennett, R; Modafferi, D; Zhou, L; Bell, D; Appleton, B | Safety of extended treatment with anakinra in patients with rheumatoid arthritis | | | | 2006 | | CENTRAL | title/abstract | irrelevant |
| 2284 | Heshmati, AA; Ilka, S | Effect of dose of the corticosteroid injected locally on inflammatory diseases | | | | 2019 | | CENTRAL | title/abstract | irrelevant |
| 2285 | Li, S; Shen, T; Liang, Y; Zhang, Y; Bai, B | Miniscalpel-needle versus steroid injection for plantar fasciitis: a randomized controlled trial with a 12-month follow-up | | | | 2014 | | CENTRAL | title/abstract | irrelevant |
| 2286 | Crawford, F; Atkins, D; Young, P; Edwards, J | Steroid injection for the treatment of plantar fasciitis: evidence of short term effectiveness. A randomised controlled trial | | | | 1999 | | CENTRAL | title/abstract | irrelevant |
| 2287 | Biswas, A; Pal, A; Amilta, A | A comparative study of efficacy of oral nonsteroidal antiinflammatory agents and locally injectable steroid for the treatment of plantar fasciitis | | | | 2011 | | CENTRAL | title/abstract | irrelevant |
| 2288 | ISRCTN79628180, | A randomised placebo controlled trial to compare ultrasound guided with palpation guided steroid injection in plantar fasciitis | | | | 2011 | | CENTRAL | title/abstract | irrelevant |
| 2289 | Moshrif, AA; Elwan, M | The effect of addition of buffered dextrose solution on pain occurring during local steroid injection for plantar fasciitis | | | | 2019 | | CENTRAL | title/abstract | irrelevant |
| 2290 | Kumar, S; Kumar, A | A Clinical Comparative Evaluation of the Effectiveness of Dry Needling and Steroid Injection in Treating Plantar Fasciitis | | | | 2024 | | CENTRAL | title/abstract | irrelevant |
| 2291 | Tiwari, M; Bhargava, R | Platelet rich plasma therapy: a comparative effective therapy with promising results in plantar fasciitis | | | | 2013 | | CENTRAL | title/abstract | irrelevant |
| 2292 | IRCT20220614055168N1, | Corticosteroid in plantar fasciitis "Effect of ozone in the treatment of plantar fasciitis" | | | | 2022 | | CENTRAL | title/abstract | trial registry |
| 2293 | NCT05367271, | The Efficacy of Botulinum Toxin vs. Corticosteroid for the Treatment of Refractory Plantar Fasciitis | | | | 2022 | | CENTRAL | title/abstract | trial registry |
| 2294 | Mishra, BN; Poudel, RR; Banskota, B; Shrestha, BK; Banskota, AK | Effectiveness of extra-corporeal shock wave therapy (ESWT) vs methylprednisolone injections in plantar fasciitis | | | | 2019 | | CENTRAL | title/abstract | irrelevant |
| 2295 | Zamani, B; Hadizadeh-Moghdam, M; Moravveji, SA | Comparing the effect of low-power laser therapy with methylprednisolone injection in unilateral plantar fasciitis | | | | 2014 | | CENTRAL | title/abstract | irrelevant |
| 2296 | Karagounis, P; Tsironi, M; Prionas, G; Tsiganos, G; Baltopoulos, P | Treatment of plantar fasciitis in recreational athletes: two different therapeutic protocols | | | | 2011 | | CENTRAL | title/abstract | irrelevant |
| 2297 | Al-Bluwi, MT; Sadat-Ali, M; Al-Habdan, IM; Azam, MQ | Efficacy of EZStep in the management of plantar fasciitis: a prospective, randomized study | | | | 2011 | | CENTRAL | title/abstract | irrelevant |
| 2298 | Lee, DO; Yoo, JH; Cho, HI; Cho, S; Cho, HR | Comparing effectiveness of polydeoxyribonucleotide injection and corticosteroid injection in plantar fasciitis treatment: a prospective randomized clinical study | | | | 2020 | | CENTRAL | title/abstract | irrelevant |
| 2299 | Gurcay, E; Kara, M; Karaahmet, OZ; Ata, AM; Onat, ÅžÅž; Ã–zÃ§akar, L | Shall We Inject Superficial or Deep to the Plantar Fascia? An Ultrasound Study of the Treatment of Chronic Plantar Fasciitis | | | | 2017 | | CENTRAL | duplicate | duplicate |
| 2300 | Hocaoglu, S; Vurdem, UE; Cebicci, MA; Sutbeyaz, ST; Guldeste, Z; Yunsuroglu, SG | Comparative Effectiveness of Radial Extracorporeal Shockwave Therapy and Ultrasound-Guided Local Corticosteroid Injection Treatment for Plantar Fasciitis | | | | 2017 | | CENTRAL | title/abstract | irrelevant |
| 2301 | Sconfienza, LM; Ferrero, G; Orlandi, D; Fabbro, E; Martini, C; Silvestri, E | One-year outcome of ultrasound-guided percutaneous treatment of plantar fasciitis: a randomized controlled trial | | | | 2011 | | CENTRAL | title/abstract | irrelevant |
| 2302 | Hadi, ZW; Al-Adili, SS | The effect of submucous dexamethasone injection on the post-operative sequelae after impacted third molar surgery | | | | 2020 | | CENTRAL | fulltext | ineligible population |
| 2303 | Sorrentino, F; Iovane, A; Vetro, A; Vaccari, A; Mantia, R; Midiri, M | Role of high-resolution ultrasound in guiding treatment of idiopathic plantar fasciitis with minimally invasive techniques | | | | 2008 | | CENTRAL | title/abstract | irrelevant |
| 2304 | Svensson, B; Boonen, A; Albertsson, K; van der Heijde, D; Keller, C; HafstrÃ¶m, I | Low-dose prednisolone in addition to the initial disease-modifying antirheumatic drug in patients with early active rheumatoid arthritis reduces joint destruction and increases the remission rate: a two-year randomized trial | | | | 2005 | | CENTRAL | title/abstract | irrelevant |
| 2305 | Riel, H; Vicenzino, B; Olesen, JL; Jensen, MB; Ehlers, LH; Rathleff, MS | Corticosteroid injection plus exercise versus exercise, beyond advice and a heel cup for patients with plantar fasciopathy: protocol for a randomised clinical superiority trial (the FIX-Heel trial) | | | | 2020 | | CENTRAL | title/abstract | irrelevant |
| 2306 | He, Y; Mu, K; Liu, R; Zhang, J; Xiang, N | Comparison of two different regimens of intravenous methylprednisolone for patients with moderate to severe and active Graves' ophthalmopathy: a prospective, randomized controlled trial | | | | 2017 | | CENTRAL | title/abstract | irrelevant |
| 2307 | Lucas, CE; Ledgerwood, AM | The cardiopulmonary response to massive doses of steroids in patients with septic shock | | | | 1984 | | CENTRAL | title/abstract | irrelevant |
| 2308 | Lesko, SM | The safety of ibuprofen suspension in children | | | | 2003 | | CENTRAL | title/abstract | irrelevant |
| 2309 | Davis, JS; Mackrow, C; Binks, P; Fletcher, W; Dettwiller, P; Marshall, C; Day, J; Pratt, W; Tong, SY | A double-blind randomized controlled trial of ibuprofen compared to placebo for uncomplicated cellulitis of the upper or lower limb | | | | 2017 | | CENTRAL | title/abstract | irrelevant |
| 2310 | Chhabra, D; Alvarado, A; Dalal, P; Leventhal, J; Wang, C; Sustento-Reodica, N; Najafian, N; Skaro, A; Levitsky, J; Mas, V; Gallon, L | Impact of calcineurin-inhibitor conversion to mTOR inhibitor on renal allograft function in a prednisone-free regimen | | | | 2013 | | CENTRAL | title/abstract | irrelevant |
| 2311 | IRCT2017022124572N5, | Effects of ozone and corticostroid on plantar fasciitis treatment | | | | 2017 | | CENTRAL | title/abstract | irrelevant |
| 2312 | IRCT20160508027797N7, | The effect of autologous blood on plantar fasciitis | | | | 2020 | | CENTRAL | title/abstract | irrelevant |
| 2313 | Hammer, DS; Rupp, S; Kreutz, A; Pape, D; Kohn, D; Seil, R | Extracorporeal shockwave therapy (ESWT) in patients with chronic proximal plantar fasciitis | | | | 2002 | | CENTRAL | title/abstract | irrelevant |
| 2314 | Ahern, ES; Cubitt, A; Ballard, E; Teng, MWL; Dougall, WC; Smyth, MJ; Hughes, BGM | Activator of NFkB ligand (RANKL) inhibition in nonsmall cell lung cancer (NSCLC) (POPCORN) | | | | 2019 | | CENTRAL | title/abstract | irrelevant |
| 2315 | Lipton, RB; Goldstein, J; Baggish, JS; Yataco, AR; Sorrentino, JV; Quiring, JN | Aspirin is efficacious for the treatment of acute migraine | | | | 2005 | | CENTRAL | title/abstract | irrelevant |
| 2316 | IRCT2015041321744N1, | Effects of dextrose and corticostroid on plantar fasciitis treatment | | | | 2015 | | CENTRAL | title/abstract | irrelevant |
| 2317 | Gandhi, SD; Wahlmeier, ST; Louie, P; Sauber, R; Tooley, TR; Baker, KC; Park, DK | Effect of local retropharyngeal steroids on fusion rate after anterior cervical discectomy and fusion | | | | 2020 | | CENTRAL | title/abstract | irrelevant |
| 2318 | Chavez-Mendoza, | Calcineurin Inhibitors With Reduced-Dose Steroids as First-Line Therapy for Focal Segmental Glomerulosclerosis | | | | 2019 | | CENTRAL | title/abstract | irrelevant |
| 2319 | PACTR202004672785790, | Deep friction massage for treatment of plantar fasciitis | | | | 2020 | | CENTRAL | title/abstract | irrelevant |
| 2320 | Spahr, L; Rubbia-Brandt, | Combination of steroids with infliximab or placebo in severe alcoholic hepatitis: a randomized controlled pilot study | | | | 2002 | | CENTRAL | title/abstract | irrelevant |
| 2321 | Ahern, E; Cubitt, A; Ives | Popcorn: pharmacodynamics of preoperative PD1 checkpoint blockade and rankl inhibition in non-small cell lung cancer (NSCLC): a phase 1b/2 investigator-sponsored trial in progress | | | | 2020 | | CENTRAL | title/abstract | irrelevant |
| 2322 | Soraganvi, P; Raju, R | IS PLATELET RICH PLASMA INJECTION IS MORE EFFECTIVE THAN STEROID INJECTION IN REDUCING PLANTAR FASCIA THICKNESS AND SYMPTOMS IN THE TREATMENT OF CHRONIC PLANTAR FASCIITIS? | | | | 2021 | | CENTRAL | title/abstract | irrelevant |
| 2323 | Soraganvi, P; | Is platelet-rich plasma injection more effective than steroid injection in the treatment of chronic plantar fasciitis in achieving long-term relief? | | | | 2019 | | CENTRAL | title/abstract | irrelevant |
| 2324 | Naik, HB; Raza, SMG; Sirsikar, A; Maravi, LS | A COMPARATIVE PROSPECTIVE STUDY OF FUNCTIONAL OUTCOME IN PLANTAR FASCIITIS TREATED WITH LOCAL STEROID INJECTION VERSUS LOCAL PLATELET RICH PLASMA INJECTION | | | | 2025 | | CENTRAL | title/abstract | irrelevant |
| 2325 | Sinha, N; Arun, ; Malhotra, N; Kumar, A | Comparative evaluation of the efficacy of plateletrich plasma injection with a combination of local anaesthetic and steroid in plantar fasciitis: a prospective study | | | | 2024 | | CENTRAL | title/abstract | irrelevant |
| 2326 | IRCT20210305050582N1, | Prolotherapy and corticosteroid injection in plantar fasciitis | | | | 2021 | | CENTRAL | title/abstract | irrelevant |
| 2327 | ISRCTN36539116, | A double-blind, controlled study of corticosteroid injection in plantar fasciitis | | | | 2002 | | CENTRAL | duplicate | duplicate |
| 2328 | Khurana, A; Dhankhar, V; Goel, N; Gupta, R; Goyal, A | Comparison of midterm results of Platelet Rich Plasma (PRP) versus Steroid for plantar fasciitis: a randomized control trial of 118 patients | | | | 2021 | | CENTRAL | title/abstract | irrelevant |
| 2329 | IRCT20180101038180N1, | Comparative efficacy of corticosteroid injections versus laser therapy in patients with plantar fasciitis attending | | | | 2018 | | CENTRAL | title/abstract | irrelevant |
| 2330 | Maneerit, J; Sriworakun, C; Budhraja, N; Nagavajara, P | Trigger thumb: results of a prospective randomised study of percutaneous release with steroid injection versus steroid injection alone | | | | 2003 | | CENTRAL | title/abstract | irrelevant |
| 2331 | Dionne, RA; Haynes, D; Brahim, JS; Rowan, JS; Guivarc'h, PH | Analgesic effect of sustained-release flurbiprofen administered at the site of tissue injury in the oral surgery model | | | | 2004 | | CENTRAL | title/abstract | irrelevant |
| 2332 | Ong, KS; Seymour, RA; Chen, FG; Ho, VC | Preoperative ketorolac has a preemptive effect for postoperative third molar surgical pain | | | | 2004 | | CENTRAL | title/abstract | irrelevant |
| 2333 | Graziani, F; Corsi, L; Fornai, M; Antonioli, L; Tonelli, M; Cei, S; Colucci, R; Blandizzi, C; Gabriele, M; Del Tacca, M | Clinical evaluation of piroxicam-FDDF and azithromycin in the prevention of complications associated with impacted lower third molar extraction | | | | 2005 | | CENTRAL | title/abstract | irrelevant |
| 2334 | ACTRN12619000888167, | Does a single dose of steroid improve the time to resolution of cellulitis (skin infection) in adults when given with standard antibiotic treatment | | | | 2019 | | CENTRAL | title/abstract | irrelevant |
| 2335 | NCT02087527, | Use of Corticosteroids in Children With Cellulitis | | | | 2014 | | CENTRAL | title/abstract | trial registry |
| 2336 | Conway, R; O'Neill, L; O'Flynn, E; McCarthy, GM; Murphy, C; Veale, DJ; Fearon, U; Molloy, ES | Ustekinumab for the treatment of refractory giant cell arteritis | | | | 2015 | | CENTRAL | title/abstract | irrelevant |
| 2337 | Litkowski, LJ; Christensen, SE; Adamson, DN; Van Dyke, T; Han, SH; Newman, KB | Analgesic efficacy and tolerability of oxycodone 5 mg/ibuprofen 400 mg compared with those of oxycodone 5 mg/acetaminophen 325 mg and hydrocodone 7.5 mg/acetaminophen 500 mg in patients with moderate to severe postoperative pain: a randomized, double-blind, placebo-controlled, single-dose, parallel-group study in a dental pain model | | | | 2005 | | CENTRAL | title/abstract | irrelevant |
| 2338 | Sawan, ZH; El-Tohamy, SA; Elhossieny, KM; Basha, OHA-H; Hafez, AS | Analgesic efficacy and functional outcome in refractory cases of plantar fasciitis treated with platelet-rich plasma: randomized comparative study with corticosteroids injection | | | | 2023 | | CENTRAL | title/abstract | irrelevant |
| 2339 | Afzal, T; Nazir, F; Saif-Ur-Rehman, ; Anis, Y | Comparison of local injection of corticosteroid and platelet rich plasma in patients with plantar fasciitis | | | | 2019 | | CENTRAL | title/abstract | irrelevant |
| 2340 | IRCT138811093220N1, | The effect of laser on planter fasciitis | | | | 2011 | | CENTRAL | title/abstract | trial registry |
| 2341 | IRCT2014011316201N1, | platelet rich plasma effect on plantar fasciitis | | | | 2014 | | CENTRAL | title/abstract | trial registry |
| 2342 | Kamala, GR; Hanumantharaya, GH | Comparative Study of Corticosteroids v/s Platelet Rich Plasma for the Treatment of Plantar Fasciitis in a Teaching Hospital | | | | 2024 | | CENTRAL | title/abstract | irrelevant |
| 2343 | Donley, BG; Moore, T; Sferra, J; Gozdanovic, J; Smith, R | The efficacy of oral nonsteroidal anti-inflammatory medication (NSAID) in the treatment of plantar fasciitis: a randomized, prospective, placebo-controlled study | | | | 2007 | | CENTRAL | title/abstract | irrelevant |
| 2344 | Nelson, DA; Landau, WM | Intraspinal steroids: history, efficacy, accidentality, and controversy with review of united states food and drug administration reports | | | | 2001 | | CENTRAL | title/abstract | irrelevant |
| 2345 | IRCT2015041821830N1, | Platelet rich plasma for treatment of plantar fasciitis | | | | 2015 | | CENTRAL | title/abstract | trial registry |
| 2346 | Bahrami, MH; Raeissadat, SA; Barchinejad, M; Elyaspour, D; Rahimi-Dehgolan, S | Local ozone (O2 â€“o3) versus corticosteroid injection efficacy in plantar fasciitis treatment: a double-blinded rct | | | | 2019 | | CENTRAL | title/abstract | irrelevant |
| 2347 | Rehncy, JS; Suraj, ; Bakshi, AS; Seth, A; Singh Chawla, HK; Gera, K | Comparative Study of Local Injection of Platelet-Rich Plasma versus Local Corticosteroids Injections in Treatment of Plantar Fasciitis | | | | 2024 | | CENTRAL | title/abstract | irrelevant |
| 2348 | Paruchuri, ST; Paruchuri, PK; Paruchuri, VK; Paruchuri, L | Role of Per Cutaneous Plantar Fascia Partial Tenotomy with Prp Injection In Refractory Plantar Fasciitis Pain | | | | 2017 | | CENTRAL | title/abstract | irrelevant |
[truncated: 108,558 more chars]
